# Pd(II)‐Mediated C−H Activation for Cysteine Bioconjugation

**DOI:** 10.1002/chem.202104385

**Published:** 2022-01-12

**Authors:** James A. R. Tilden, Anneke T. Lubben, Shaun B. Reeksting, Gabriele Kociok‐Köhn, Christopher G. Frost

**Affiliations:** ^1^ Department of Chemistry University of Bath Claverton Down BA2 7AY Bath United Kingdom; ^2^ Materials and Chemical Characterization (MC2) University of Bath Claverton Down BA2 7AY Bath United Kingdom

**Keywords:** bioconjugation, C−H activation, cross-coupling, cysteine, palladium

## Abstract

Selective bioconjugation remains a significant challenge for the synthetic chemist due to the stringent reaction conditions required by biomolecules coupled with their high degree of functionality. The current trailblazer of transition‐metal mediated bioconjugation chemistry involves the use of Pd(II) complexes prepared via an oxidative addition process. Herein, the preparation of Pd(II) complexes for cysteine bioconjugation via a facile C−H activation process is reported. These complexes show bioconjugation efficiency competitive with what is seen in the current literature, with a user‐friendly synthesis, common Pd(II) sources, and a more cost‐effective ligand. Furthermore, these complexes need not be isolated, and still achieve high conversion efficiency and selectivity of a model peptide. These complexes also demonstrate the ability to selectively arylate a single surface cysteine residue on a model protein substrate, further demonstrating their utility.

## Graphical abstract

**Out of the box**: A highly efficient bioconjugation strategy is reported utilising Pd(II) complexes prepared via C−H activation, which enable selective *S‐*arylation of cysteine. These C−H activation complexes were prepared using common Pd(II) sources and without the need for a glovebox. A wide substrate scope is enabled by in situ generation of these complexes, which produce excellent conversions of a model tripeptide and are able to arylate surface cysteine on an intact protein.

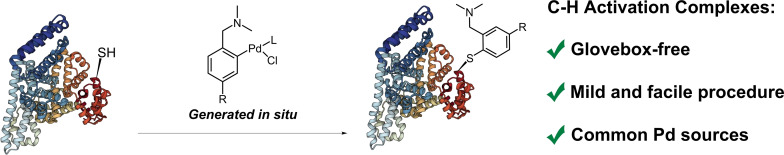

The ability to tag biomolecules, more commonly known as bioconjugation, is of great interest to synthetic chemists, biochemists, and biologists alike. This process consists of forming a covalent or characterisable non‐covalent interaction between a specific biomolecule, and another molecule. Traditionally many bioconjugation techniques, such as the Cu(I)‐catalysed alkyne/azide cycloaddition (CuAAC) reaction,[^1^, ^2^] involve the labelling of a peptide or protein that has had an alkyne or azide, two functional groups seldom found in nature, incorporated into its structure. This reaction represented the birth of ‘click’ chemistry and enabled vast substrate scopes on biomolecules. Whilst the utility of this technique cannot be overstated, many chemists have been developing techniques to selectively functionalise natural amino acids by targeting uniquely nucleophilic residues such as cysteine. Much of this work has focused on using organic electrophiles: α‐halocarbonyls,^[3]^ maleimides,[^4^, ^5^] and other Michael acceptors.^[6]^ These techniques often demonstrate favourable kinetics, however large equivalents of bioconjugation reagent are frequently required, causing selectivity issues.^[7]^

Recent advancements in transition metal‐mediated bioconjugation processes have proven their utility in the field owing to their excellent tolerance of a variety of functional groups, high selectivity, and favourable kinetics. Gold,[^8^, ^9^, ^10^] palladium,^[11]^ and most recently platinum^[12]^ complexes have all been successfully employed within the sphere of bioconjugation. The first use of gold‐based organometallic complexes in bioconjugation utilised Au(III)‐Ar complexes prepared via a cyclometallation process.^[8]^ However, the most recent examples of Au(III)‐Ar complexes for bioconjugation are prepared via an oxidative addition process,^[9]^ and these complexes have been successfully applied toward the formation of 3D peptide nanoassemblies.^[10]^

One of the first examples of Pd complexes used in bioconjugation was the use of *π*‐allylpalladium complexes, reactive towards *O*‐arylation of tyrosine residues.^[13]^ However, most examples of organometallic reagents may be found utilising Pd(II) oxidative addition complexes (OACs). Successful tuning of reactivity towards cysteine,^[11]^ lysine,^[14]^ and *p*‐aminophenylalanine^[15]^ under mild, biocompatible reaction conditions has been achieved since 2015.[^14^, ^16^] These highly selective and functional group‐tolerant complexes have proven themselves to be the cutting‐edge technology in the field.

Further application has seen the successful macrocyclisation of peptides,^[17]^ crosslinking of biomolecules,^[18]^ the ability to form protein cross‐coupling products,^[19]^ and most recently oligonucleotide bioconjugation.^[20]^

Pd(II) OACs have been further utilised in pharmaceutical diversification,[^21^, ^22^] and whilst their utility cannot be overstated, their adoption has been limited by unstable precursors.^[23]^ Whilst the OACs themselves are often air and moisture stable, their formation requires the use of (1,5‐cyclooctadiene)Pd(II)(CH_2_TMS)_2,_ ^[24]^ or Pd(0)(1,5‐cyclooctadiene) ligated with a phosphine ligand.^[25]^ Both of these precursors require manipulation in a glovebox.

In this work, a C−H activation approach towards the preparation of Pd(II) complexes was identified as being complementary to OACs for cysteine bioconjugation (Figure 1). Although palladacycles have been utilised in the bioconjugation of alkyne‐encoded biomolecules,^[26]^ they have not yet been applied to natural amino acids.

**Figure 1:**
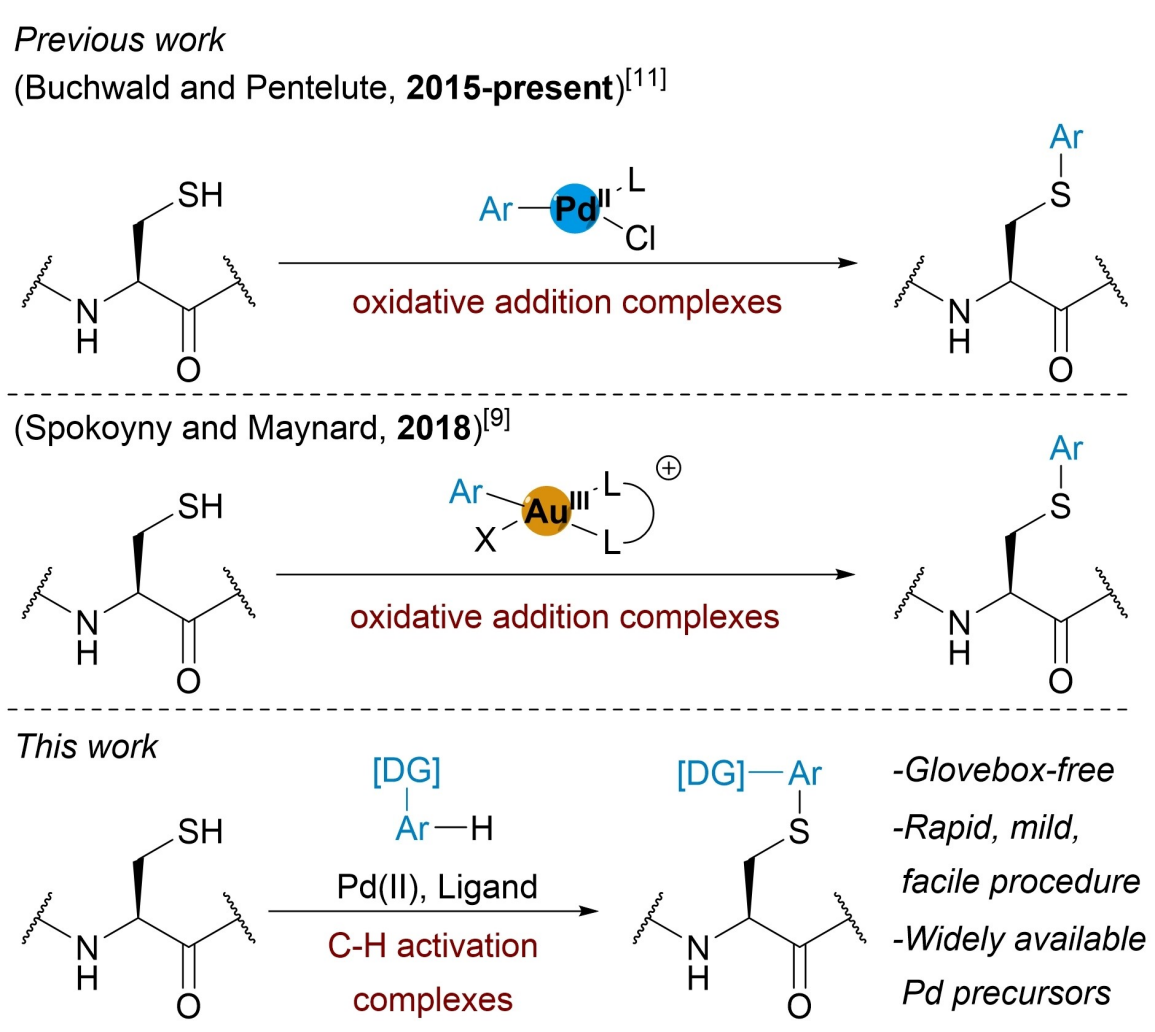
Previous work using Pd^II^ and Au^III^ oxidative addition complexes, and this work describing an Pd^II^‐mediated in situ process for cysteine *S*‐arylation.

The synthesis of palladacycles is relatively facile and may be completed without the need for a glovebox or Schlenk chemistry. Thus, Pd(II)‐Ar complexes prepared via cyclopalladation offer practical advantages to previously utilised oxidative addition complexes in the bioconjugation field. The formation of palladacycles typically produces dimeric compounds with bridging ligands. These dimeric complexes may then be cleaved using strongly σ‐donating phosphine ligands to produce a monomeric Pd(II) complex.^[27]^ This preparation produces an aryl Pd(II) species which, when the right phosphine ligand is selected, could be applied in bioconjugation chemistry.

Buchwald and Pentelute have shown that the bulky biarylphosphine ligand RuPhos produces quantitative conversions in their bioconjugation protocol.^[11]^ Xantphos has also proven to be a suitable ligand for Pd(II) catalysed C−S bond formation^[28]^ and cysteine bioconjugation in aqueous media.^[29]^ Thus, (dmba)Pd(II)Cl(Xantphos) **3** and (dmba)Pd(II)Cl(RuPhos) **4** were prepared by the reaction of [Pd(dmba)(μ‐Cl)]_2_ **2** and two equivalents of phosphine ligand in CH_2_Cl_2_ (Scheme 1). These reactions both proceeded at room temperature under air and gave a quantitative yield of Pd(II)‐Ar species. Acetanilide‐derived complexes **7** and **8** were prepared in an analogous manner. The structures of **3** and **4** were confirmed via single‐crystal X‐ray diffraction (Figure 2). Deposition Numbers 2098651 (for **3**), 2098650 (for **4**) contain the supplementary crystallographic data for this paper. These data are provided free of charge by the joint Cambridge Crystallographic Data Centre and Fachinformationszentrum Karlsruhe Access Structures service.

**Scheme 1:**
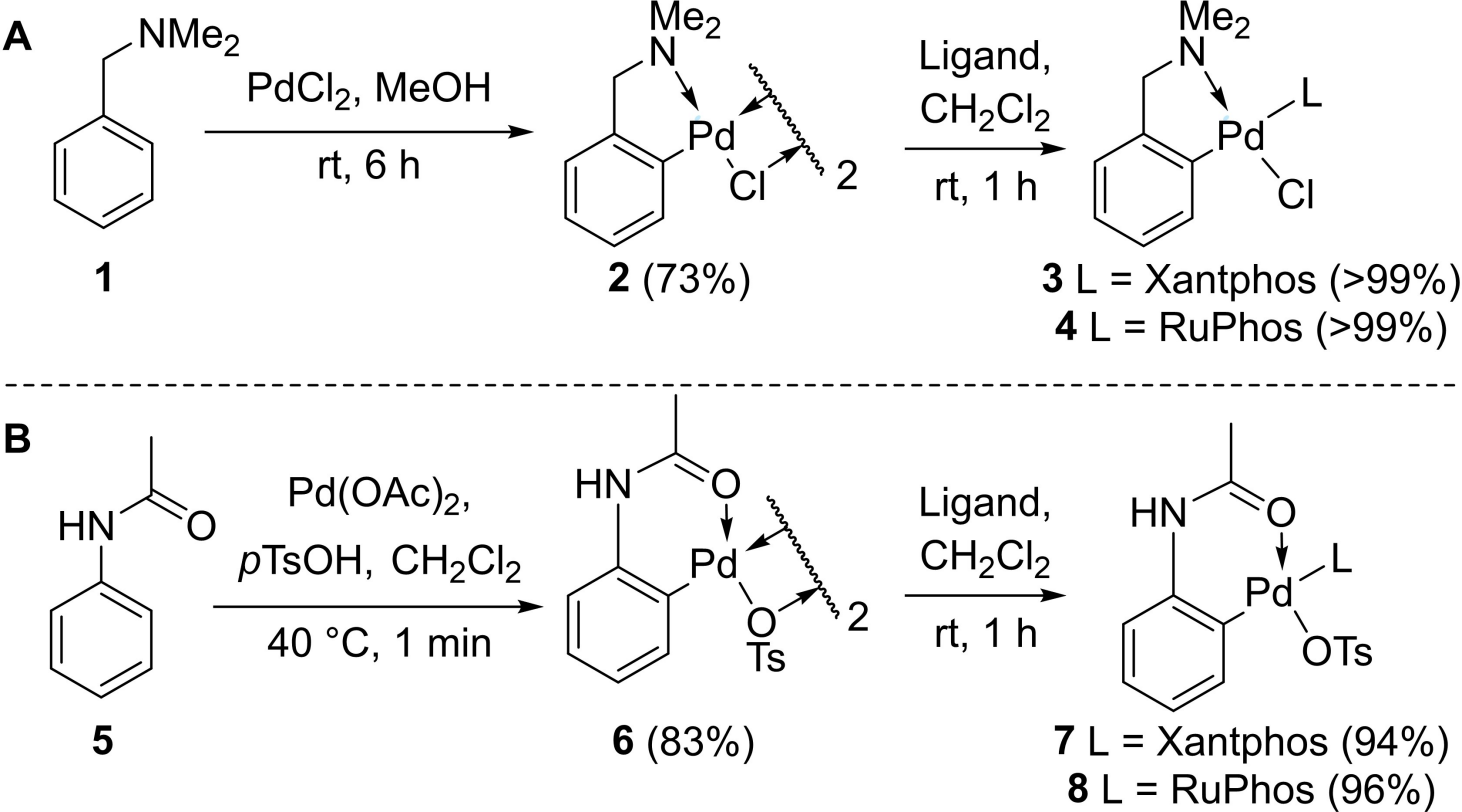
**A**: Preparation of (dmba)Pd(II)Cl(Xantphos) **3** and (dmba)Pd(II)Cl(RuPhos) **4** from *N,N*‐dimethylbenzylamine (dmba) **2** and PdCl_2_. **B**: Preparation of (acetanilide)Pd(II)OTs(RuPhos) **7** and (acetanilide)Pd(II)OTs(Xantphos) **8** from acetanilide **5**.

**Figure 2:**
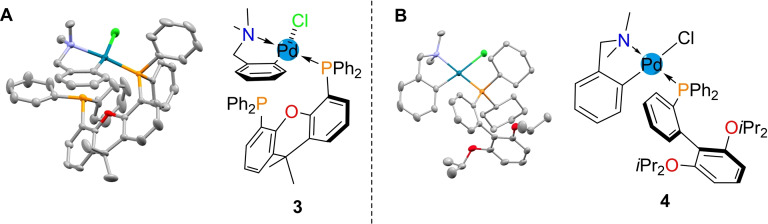
**A** X‐ray single‐crystal structure of (dmba)Pd(II)Cl(Xantphos) **3** alongside ChemDraw view. Solvent (CHCl_3_) and Hydrogen atoms omitted for clarity, and thermal ellipsoids drawn at 50 % probability. **B** X‐ray single‐crystal structure of (dmba)Pd(II)Cl(RuPhos) **4** alongside ChemDraw view. Solvent (MeOH) and Hydrogen atoms omitted for clarity, and thermal ellipsoids drawn at 50 % probability.

Optimisation of cysteine arylation was performed using glutathione (GSH) as a model peptide substrate owing to its availability and previous use in transition metal‐mediated bioconjugation reactions (Scheme 2).^[9]^ 94 % conversion to the corresponding *S‐*Arylated GSH conjugate was observed after GSH was treated with **4** at rt in a mixture of H_2_O/organic solvent (95 : 5) using 10 mM phosphate buffer at pH 7.5 in 5 min after which, 3‐mercaptopropionic acid (3‐MPA) was added as a palladium scavenger. ≥95 % conversion was observed for **3**, **7**, and **8**. The cysteine selectivity of the Xantphos complex **3** was demonstrated by investigating reactivity with *N‐*acetylcysteine (NAC), and H‐lys‐OMe. **3** showed successful arylation of NAC, but no reaction with H‐Lys‐OMe, as assessed by LC‐MS. Selectivity of **3** towards cysteine was also confirmed with MS/MS analysis, with only the cysteine‐containing fragment of glutathione being arylated.

**Scheme 2:**
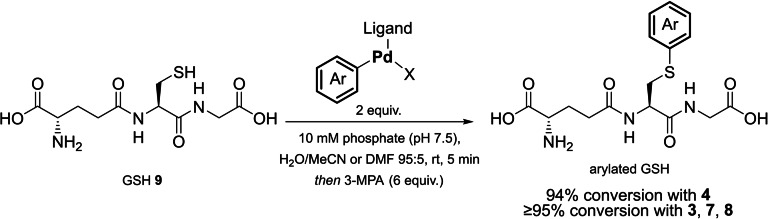
Initial assessment of the bioconjugation efficiency of Pd(II) complexes prepared via a C−H activation pathway using GSH as a model substrate.

As expected, all complexes were prepared as bench stable, crystalline solids. Commercial solvents and reagents were used without further purification, and the excellent benchtop stability of these complexes was demonstrated by **3** and **4**, which showed no degradation after 8 months, as assessed by ^1^H and ^31^P NMR. More importantly, these complexes retained excellent bioconjugation efficiency after 8 months.

A selection of *para‐* functionalised dmba analogues were prepared to explore the scope of this process. *Para‐* functionalisation retained symmetry in the molecules and prevented any possible regioselectivity issues in the cyclopalladation reaction. Compounds bearing biologically relevant functionality including an electrochemical tag (**12**) and a triethylene glycol (TEG) chain (**13**), as well as an electron donating group (**11**) and biaryl species (**10**) were prepared. These compounds were either prepared using PdCl_2_ or Na_2_PdCl_4_ as the palladium source to produce the dimeric Pd(II) complex, which was then treated with RuPhos to produce the corresponding monomeric Pd(II) complex. All reactions proceeded under air and produced bench stable, crystalline solids. Notably, when dmba was functionalised with an alkyne the cyclopalladation process did not proceed, owing to reactivity between cyclopalladated species and alkynes, which has previously been utilised by Cheng et al.^[26]^ for functionalising alkyne‐encoded proteins. Conversion to arylated GSH was respectable for all *para‐* substituted dmba analogues, ranging from 63 % to 93 % (Figure 3) after 5 min, determined by LC‐MS analysis.

**Figure 3:**
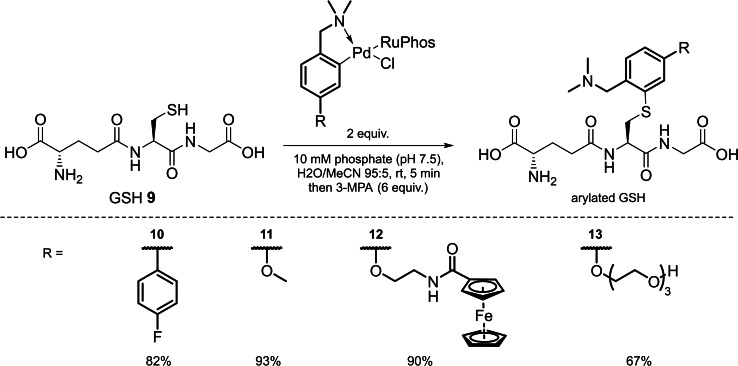
Glutathione functionalisation with isolated Pd(II) complexes (**10**–**13**) to produce respective arylated glutathione products. Conversion of glutathione for each complex shown.

To enable broader application of this bioconjugation process, an in situ method was investigated (Figure 4). Combining Na_2_PdCl_4_ and dmba **1** (4 equiv. to GSH) in MeOH for 1 h, followed by dilution with MeCN or DMF and addition of 1 equivalent of RuPhos or Xantphos produced a stock solution of **3** or **4** after another h. This solution could then be applied to the previously optimised bioconjugation conditions (2 equiv. Pd(II) complex, 10 mM phosphate buffer, 95 : 5 H_2_O/solvent, pH 7.5), resulting in 78 % and 80 % conversion to arylated GSH for both RuPhos and Xantphos, respectively. This reaction was further optimised by using fewer eqiuv. of dmba **1** (2 equiv. to GSH), with the addition of NaOAc (2 equiv. to GSH) to both aid cyclopalladation^[30]^ and act as a base. As a result, conversions of 93 % and 98 % were achieved for RuPhos and Xantphos, respectively. Notably, the phosphine ligand must be added after the formation of the palladacycle, as the cyclopalladation reaction does not proceed in the presence of phosphine ligands.^[31]^

**Figure 4:**
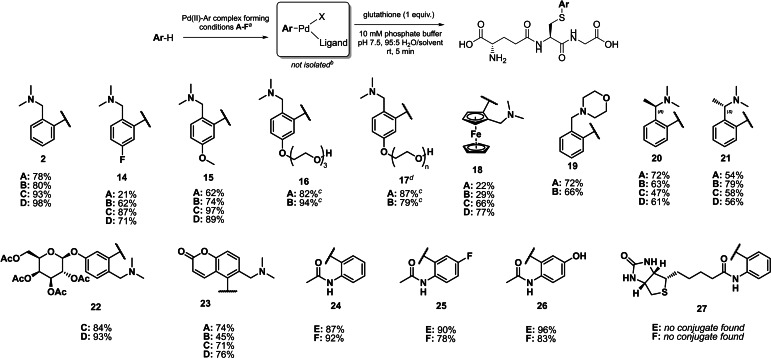
Assessment of in situ method for cysteine S‐arylation on glutathione using a range of compounds (**2**, **14**–**27**) with the ability to undergo cyclopalladation. All conversions displayed calculated using remaining concentration of free GSH after reactions by LC‐MS. ^[a]^ Pd(II)‐Ar complex forming conditions: **A**: Ar−H (4 equiv.), Na_2_PdCl_4_ (2 equiv.), MeOH, rt, 1 h then RuPhos (2 equiv.), MeCN, rt, 1 h; **B**: Ar−H (4 equiv.), Na_2_PdCl_4_ (2 equiv.), MeOH, rt, 1 h then Xantphos (2 equiv.), DMF, rt, 1 h; **C**: Ar−H (2 equiv.), Na_2_PdCl_4_ (2 equiv.), NaOAc (2 equiv.) MeOH, rt, 1 h then RuPhos (2 equiv.), MeCN, rt, 1 h; **D**: Ar−H (2 equiv.), Na_2_PdCl_4_ (2 equiv.), NaOAc (2 equiv.) MeOH, rt, 1 h then Xantphos (2 equiv.), DMF, rt, 1 h; **E**: Ar−H (2 equiv.), Pd(OAc)_2_ (2 equiv.), pTsOH (2 equiv.), dioxane, rt, 1 h then RuPhos (2 equiv.), MeCN, rt, 1 h; **F**: Ar−H (2 equiv.), Pd(OAc)_2_ (2 equiv.), pTsOH (2 equiv.), dioxane, rt, 1 h then Xantphos (2 equiv.), DMF, rt, 1 h. Equivalents all relative to GSH. ^[b]^ For Pd(II) complex forming conditions **A**–**D**, X=Cl. For **E** and **F**, X=OTs. ^[c]^ Cyclopalladation step of Pd(II) source with **16** and **17** required 16 h reaction time. ^[d]^ n=8.2–9.1 (synthesised from PEG‐400).

The ease and cost‐effectiveness of this in situ procedure allowed a substrate scope of a wide range of dimethylaminomethyl‐functionalised aromatic compounds. The entire protocol takes little over 2 h and requires only a balance and micropipettes. Modest to excellent conversions were observed for all substrates, which notably include direct conjugation to a ferrocene (**18**), polyethylene glycol (PEG) functionalised moieties (**16**, **17**), a protected galactose (**22**), and fluorescent tag (**23**). For **2**, the addition of NaOAc in the cyclopalladation step of the reaction produced a modest increase in conversions, which was also demonstrated with **18**.

The scope of this process is not limited to functionalised dmba analogues, as acetanilide and derivatives are effective substrates. The Pd(II) source has to be changed to Pd(OAc)_2_, and the addition of *p*‐toluenesulfonic acid is necessary for cyclopalladation, as demonstrated by Cheng et al.^[26]^ No GSH labelling was observed when Na_2_PdCl_4_ in MeOH was used for cyclopalladation of acetanilide derivatives. Bioconjugation with acetanilide was compatible with electron withdrawing (**25**) and donating (**26**) groups. Unfortunately, biotinylated derivative **27** showed no bioconjugation, likely owing to the very low solubility of **27**.

Following the success of both isolated and in situ generated Pd(II) complexes prepared via C−H functionalisation, direct arylation of surface cysteine on proteins was the next step. Thus, the selective functionalisation of cysteine on intact proteins was assessed using bovine serum albumin (BSA) **28** due to its single surface‐exposed cysteine (Cys‐34). BSA was treated with 10 equiv. of in situ generated Pd(II) complex in PBS:organic solvent (9 : 1) at pH 7.3, 37 °C for 1 h. Following trypsin digest, the selective modification of Cys‐34 with triethylene glycol (**29**), and acylated β‐d‐galactopyranose (**30**) derivatives were confirmed by LC‐MS/MS analysis (Scheme 3). Estimated conversions to singly arylated BSA were between 92 % and 99 %.

**Scheme 3:**
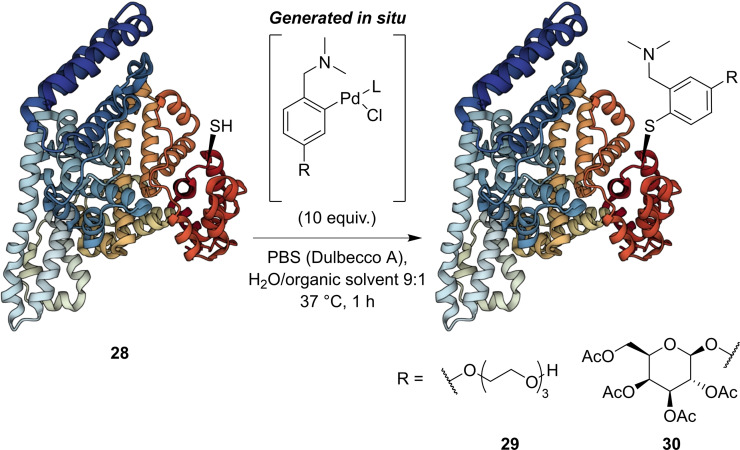
Arylation of BSA using in situ generated Pd(II) complexes. Structure of BSA^[32]^ **28** from PDB (ID: 3 V03). BSA representation generated with Mol*.^[33]^

In this work, a range of cyclopalladated Pd(II) complexes were synthesised based on *N,N*‐dimethylbenzylamine and acetanilide cores. The synthetic route towards these cyclometallated Pd(II) complex is facile, rapid, mild, and does not require the use of a glovebox. Both RuPhos and Xantphos have been demonstrated to be effective bioconjugation ligands when ligated to Pd(II) complexes, producing conversions in excess of 90 % to arylated cysteine. This offers a possible alternative to the currently used biarylphosphine ligands. In addition, a method for generating Pd(II) complexes in situ for bioconjugation has been optimised and has demonstrated the ability to selectively tag cysteine residues with a wide variety of functionalities. Pleasingly, both *N,N*‐dimethylbenzylamine and acetanilide derivatives showed excellent conversions in this procedure. This has resulted in a facile methodology for bioconjugation using Pd(II) complexes which may be adopted without the use of specialist air‐free techniques.

## Conflict of interest

The authors declare no conflict of interest.

## Supporting information

As a service to our authors and readers, this journal provides supporting information supplied by the authors. Such materials are peer reviewed and may be re‐organized for online delivery, but are not copy‐edited or typeset. Technical support issues arising from supporting information (other than missing files) should be addressed to the authors.

Supporting Information

## Data Availability

The data that support the findings of this study are available in the supplementary material of this article.
