# Supplementary material for: Pd(II)‐Mediated C−H Activation for Cysteine Bioconjugation
Source: Chemistry. 2022 Jan 12;28(11):e202104385. doi: 10.1002/chem.202104385 (PMC9305290; doi:10.1002/chem.202104385)
Supplement: Supplementary file 1 — Supporting Information [file CHEM-28-0-s001.pdf]

Chemistry–A European Journal

Supporting Information

Pd(II)-Mediated C–H Activation for Cysteine Bioconjugation

James A. R. Tilden, Anneke T. Lubben, Shaun B. Reeksting, Gabriele Kociok-Köhn, and Christopher G. Frost*

Contents

General Experimental 2
Synthetic Experimental 4
Synthesis of 2, 3, and 4 4
Synthesis of 6, 7 and 8 7
Synthesis of 10 10
Synthesis of 11 13
Synthesis of 12 15
Synthesis of 13 21
Synthesis of 14 25
Synthesis of 17 26
Synthesis of 18 29
Synthesis of 19 30
Synthesis of 20 and 21 31
Synthesis of 22 33
Synthesis of 23 35
Synthesis of 24 36
Synthesis of 27 37
LC-MS Analysis 38
General procedure for glutathione labelling 39
Glutathione calibration curve 40
(dmba)Pd(II)Cl(Xantphos) Residue Selectivity 41
Glutathione arylation using isolated Pd(II) complexes 42
Results of Pd(II)-Ar complex forming conditions A and B 46
Results of Pd(II)-Ar complex forming conditions C and D 55
Results of Pd(II)-Ar complex forming conditions E and F 58
Labelling of BSA using *in situ* generated Pd(II) complexes 61
Stability of cyclopalladated complexes 66
NMR spectra 70
Crystallographic Data 115
References 117

General Experimental

NMR spectra were recorded on an Agilent Technologies 500 MHz spectrometer (^{1}H NMR at 500 MHz, ^{11}B NMR at 160 MHz, ^{13}C NMR at 126 MHz, ^{19}F NMR at 470 MHz and ^{31}P NMR at 202.5 MHz), a Bruker 300 MHz spectrometer (^{1}H NMR at 300 MHz and ^{13}C NMR at 75.5 MHz) or a Bruker 400 MHz spectrometer (^{1}H NMR at 400 MHz and ^{13}C NMR at 101 MHz). Proton chemical shifts are reported in parts per million downfield from tetramethylsilane and are referenced to residual protium in the solvent. Carbon chemical shifts are reported in parts per million downfield from tetramethylsilane and are referenced to the carbon resonances of the solvent peak. Fluorine chemical shifts are reported in parts per million downfield from trichlorofluoromethane. Phosphorous chemical shifts are reported in parts per million downfield from 85% H_3PO_4. NMR data are represented as follows: chemical shift, integration, multiplicity (s = singlet, d = doublet, dd = doublet of doublets, ddd = doublet of doublet of doublets, t = triplet, q = quartet, m = multiplet), coupling constants (Hz). All spectra were recorded at 298 K, unless otherwise stated. Limited NMR data for select

High resolution mass spectra were obtained using an Agilent QTOF 6545 with Jetstream ESI spray source coupled to an Agilent 1260 Infinity II Quat pump HPLC with 1260 autosampler, column oven compartment and variable wavelength detector (VWD). The MS was operated in either positive or negative ionization mode with the gas temperature at 250°C, the drying gas at 12 L/min and the nebulizer gas at 45 psi (3.10 bar). The sheath gas temperature and flow were set to 350°C and 12 L/min, respectively. The MS was calibrated using reference calibrant introduced from the independent ESI reference sprayer. The VCap, Fragmentor and Skimmer was set to 3500, 125 and 45 respectively. Data processing was in Qual B 07.00 with a Find by formula matching tolerance of 5 ppm.

Single crystal X-ray diffraction data was collected at 150 K using either a n Agilent Xcalibur or Agilent SuperNova Dual diffractometer with either Mo-Kα (λ = 0.71073 Å) or Cu-Kα (λ = 1.5418 Å) radiation. The data collected by the diffractometer was processed using the proprietary Agilent software. Structures were solved by fill-matrix least squares refinement using either the WinGX-170 suite of programs or the programme suite X-SEED. All structural data was obtained and refined by Dr Gabriele Kociok-Köhn.

Electrochemical analysis was performed by applying a 30 µL sample to screen-printed electrochemical cell equipped with carbon working and counter electrodes and a silver (pseudo

Ag/AgCl) reference electrode. The potential across the cell was powered by a Metrohm Autolab PGSTAT30 potentiostat controlled by a laptop running General Purpose Electrochemical System (GPES) software in differential pulse mode (modulation = 0.04 s, interval = 0.1 s, initial voltage = −500 mV, end voltage = 500 mV, step potential = 3 mV, modulation amplitude 49.95 mV). Post-scan, a baseline correction (moving average: peak width = 0.03) was performed.

Analytical thin layer chromatography (TLC) was performed using aluminium-backed plates coated with Alugram® SIL G/UV254 purchased from Macherey-Nagel and visualised by UV light (254 nm), vanillin, ninhydrin or potassium permangantae staining.

Silica gel column chromatography was carried out using 60 Å, 200-400 mesh particle size silica gel purchased from Sigma-Aldrich.

IR spectra were recorded on a Perkin-Elmer 1600 FT IR spectrophotometer, with absorbencies quoted as ν in cm^{-1}. Strength of the peaks is defined as strong (s), medium (m), or weak (w). Broad (br) peaks are reported as such.

Melting points were obtained on an OptiMelt MPA100 automated melting point system.

Chemicals and solvents were purchased from Sigma-Aldrich, Merck, Lancaster Synthesis Ltd., Fisher Scientific Ltd., Strem Chemicals UK, Fluorochem, or VWR International and used without further purification unless otherwise stated. Anhydrous acetonitrile (MeCN), anhydrous dichloromethane, anhydrous tetrahydrofuran (THF) and anhydrous toluene were dried and degassed by passing through anhydrous alumina columns using an Innovative Technology Inc. PS-400-7 solvent purification system and stored under an atmosphere of argon prior to use. Anhydrous ethyl acetate (EtOAc), anhydrous *N*,*N*-dimethylfomamide (DMF), and anhydrous 1,4-dioxane were purchased from Sigma-Aldrich or Fisher Scientific and used as received.

Synthetic Experimental

Synthesis of 2, 3, and 4

Di-μ-chloro-bis(*N*,*N*-dimethylbenzylamine-2-*C*,*N*)dipalladium(II)

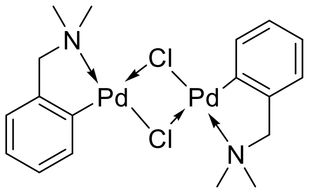

N,*N*-dimethylbenzylamine (7.35 g, 55 mmol, 2.0 equiv.) and $PdCl_2$ (4.82 g, 27 mmol, 1.0 equiv.) were combined in MeOH (270 mL) and stirred at rt for 6 h. The resulting solid was isolated *via* gravity filtration and purified by recrystallisation from benzene-*n*-hexane to yield the title compound (5.44 g, 73 %) as a green solid: m.p. 174 °C [lit.[1] 184-186 °C from benzene/hexane]; ^{1}H NMR (400 MHz, Chloroform-*d*) δ 7.24 – 7.15 (m, 1H), 7.05 – 6.95 (m, 1H), 6.94 – 6.85 (m, 2H), 3.96 (s, 2H), 2.89 (s, 3H), 2.87 (s, 3H). The spectral data are in agreement with reported literature values. [1]

Pd complex 3

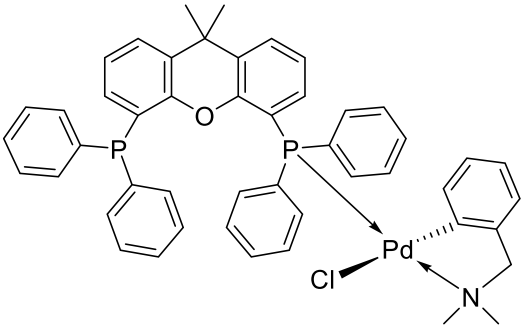

[Pd(dmba)(μ-Cl)]$_2$ (28 mg, 50 μmol, 1.0 equiv.) and XantPhos (58 mg, 100 μmol, 2.0 equiv.) were combined in CH_2Cl_2 (2 mL). After 1 h, the mixture was concentrated *in vacuo*. MTBE (200 μL) and pentane (800 μL) were added, and the mixture was left to stand for 15 min. After which, the solvent was pipetted off, and the resulting solid was washed with pentane (2 × 1 mL) to yield the **title compound** (86 mg, >99 %) as a colourless solid: m.p. decomposes at 190 °C; ^{1}H NMR (400 MHz, Chloroform-*d*, 323 K) δ 7.46 – 7.39 (m, 10H), 7.24 – 7.19 (m, 4H), 7.11 (m, 8H), 6.96 (t, *J* = 7.7 Hz, 1H), 6.90 – 6.85 (m, 2H), 6.80 (qd, i*J* = 4.5, 1.6 Hz, 2H), 6.72 (ddd, *J* = 7.3, 6.1, 2.3 Hz, 1H), 6.30 – 6.23 (m, 2H), 3.84 (s, 2H), 2.64 (s, 6H), 1.51 – 1.46 (m, 6H); ^{13}C NMR (101 MHz, $CDCl_3$) δ 152.9, 152.8, 151.1, 148.0, 137.1, 134.6, 134.5, 133.8, 130.4, 129.1, 128.0, 127.9, 124.5, 123.2, 123.1, 122.5, 72.5, 50.2, 34.4; ^{31}P NMR (162 MHz, $CDCl_3$, 323 K) δ 4.21; (ESI$^+$) of [M-Cl]$^+$ detected: 814.1931, expected for $C_{48}H_{44}NClOP_2Pd$: 814.1949; v̄max (thin film)/cm^{-1} 1434 (w), 1405 (s), 1239 (s), 739 (s), 695 (s).

Crystallisation *via* liquid-liquid diffusion of hexane layered onto a solution of the **title compound** in $CHCl_3$ (10 mg/mL) yielded crystals suitable for X-ray diffraction study.

Pd complex 4

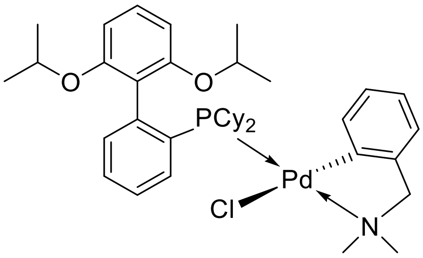

[Pd(dmba)(μ-Cl)]$_2$ (28 mg, 50 μmol, 1.0 equiv.) and RuPhos (47 mg, 100 μmol, 2.0 equiv.) were combined in CH_2Cl_2 (2 mL). After 1 h, the mixture was concentrated *in vacuo*. MTBE (200 μL) and pentane (800 μL) were added, and the mixture was left to stand for 15 min. After which, the solvent was pipetted off, and the resulting solid was washed with pentane (2 × 1 mL) to yield the **title compound** (74 mg, 99%) as a colourless solid: m.p. decomposes at 202 °C; ^{1}H NMR (400 MHz, $CDCl_3$) δ 7.67 (ddd, *J* = 14.8, 8.0, 1.4 Hz, 1H), 7.29 (d, *J* = 8.5 Hz, 1H), 7.20 (tt, *J* = 7.5, 1.8 Hz, 1H), 6.92 (ddd, *J* = 7.9, 3.1, 1.4 Hz, 1H), 6.90 – 6.82 (m, 2H), 6.75 (td, *J* = 7.3, 1.1 Hz, 1H), 6.67 – 6.54 (m, 3H), 6.46 (td, *J* = 7.5, 1.6 Hz, 1H), 4.48 (s, 2H, H-1'), 2.73 (s, 6H, H-2'), 1.71 (s, 2H), 1.26 (s, 5H), 1.11 (s, 8H); ^{13}C NMR (101 MHz, $CDCl_3$) δ 153.1, 147.5, 140.6, 140.2, 140.1, 140.0, 139.8, 133.2, 133.1, 128.9, 128.6, 128.2, 127.8, 124.8, 124.7, 124.5, 124.4, 123.0, 123.0, 122.8, 121.3, 107.0, 73.0, 72.9, 27.7, 27.6, 26.9, 26.8, 26.2, 22.7, 22.4; ^{31}P NMR (162 MHz, $CDCl_3$) δ 70.64; (ESI$^+$) *m/z* of $[M+H]^+$ detected: 742.2766, expected for $C_{39}H_{55}ClNO_2PPd$: 742.2779; v̄max (thin film)/cm^{-1} 2924 (C-H, m), 1581 (m), 1456 (s), 1243 (s), 1112 (s), 1057 (s), 727 (s).

Crystallisation *via* vapour diffusion of pentane into a solution of the **title compound** in MeOH (10 mg/mL) yielded crystals suitable for X-ray diffraction study.

Synthesis of 6, 7 and 8

Di-μ-tosyl-bis(acetanilide-2-*C*,*O*)dipalladium(II) 6

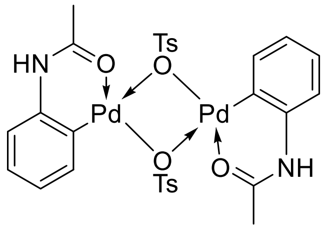

Acetanilide (27 mg, 0.20 mmol, 1.0 equiv.), $Pd(OAc)_2$ (38 mg, 0.20 mmol, 1.0 equiv.) and *p*-toluenesolfonic acid hydrate (45 mg, 0.20 mmol, 1.0 equiv.) were combined in CH_2Cl_2 (2 mL) and heated for 1 min. The resulting yellow solid was isolated *via* gravity filtration, washed with CH_2Cl_2 (3 × 2 mL) to give the **title compound** (74 mg, 83%) as a yellow solid: ^{1}H NMR (400 MHz, Methanol-d_4) δ 11.49 (s, 1H), 7.71 (d, J = 8.2 Hz, 2H), 7.25 – 7.20 (m, 1H), 7.14 – 7.08 (m, 1H), 6.94 – 6.86 (m, 3H), 2.36 (s, 3H), 2.30 (s, 3H); ^{13}C NMR (101 MHz, MeOD) δ 168.1, 141.7, 133.1, 132.2, 129.8, 127.2, 127.0, 124.3, 117.4, 114.4, 21.3, 20.7. The spectral data are in agreement with reported literature values.[2]

Pd complex 7

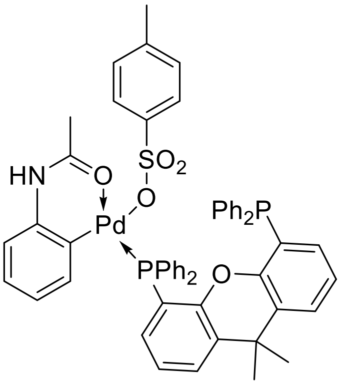

Di-µ-tosyl-bis(acetanilide-2-*C,O*)dipalladium(II) (22 mg, 25 µmol, 1.0 equiv.) and RuPhos (23 mg, 50 µmol, 1.0 equiv.) were combined in CH_2Cl_2 (2 mL) and stirred for 1 h. The reaction mixture was filtered through a bed of Celite® and concentrated *in vacuo* to yield the **title compound** (43 mg, 96%) as a yellow solid: m.p. decomposes at 120 °C; ^{1}H NMR (400 MHz, Chloroform-*d*) δ 10.52 (broad s), 7.57 (dd, *J* = 7.8, 1.5 Hz, 3H), 7.34 (d, *J* = 7.8 Hz, 2H), 7.18 – 7.07 (m, 14H), 7.03 (t, *J* = 7.5 Hz, 8H), 6.98 – 6.93 (m, 2H), 6.84 (d, *J* = 7.9 Hz, 2H), 6.74 – 6.69 (m, 1H), 6.57 – 6.52 (m, 1H), 6.26 (t, *J* = 7.5 Hz, 1H), 2.13 (s, 3H), 1.69 (s, 6H), 1.62 (s, 3H); ^{31}P NMR (162 MHz, $CDCl_3$) δ 15.2 (broad); ^{13}C NMR (101 MHz, $CDCl_3$) δ 170.0, 154.1, 154.1, 154.0, 144.2, 138.6, 133.6, 133.1, 130.6, 130.1, 128.6, 128.6, 128.3, 126.2, 125.5, 123.7, 119.8, 119.1, 35.6, 30.9, 22.8, 21.4; (ESI$^+$) *m/z* of $[M\text{-}OTs]^+$ detected: 818.1590, expected for $C_{54}H_{47}NO_5P_2PdS$: 818.1569; ν̄max (thin film)/cm^{-1} 3371 (br, w), 2978 (m), 2876 (w), 1680 (w), 1410 (s), 1368 (m), 1291 (w), 1199 (m), 1153 (m), 1117 (m), 949 (s), 734 (m), 688 (s).

Pd complex 8

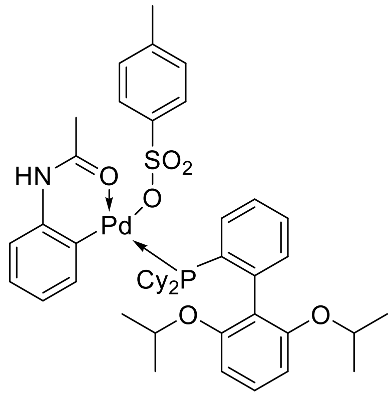

Di-µ-tosyl-bis(acetanilide-2-*C,O*)dipalladium(II) (22 mg, 25 µmol, 1.0 equiv.) and RuPhos (23 mg, 50 µmol, 1.0 equiv.) were combined in CH_2Cl_2 (2 mL) and stirred for 1 h. The reaction mixture was filtered through a bed of Celite® and concentrated *in vacuo* to yield the **title compound** (43 mg, 96%) as a yellow solid: m.p. decomposes at 110 °C; ^{1}H NMR (400 MHz, Chloroform-*d*) δ 12.05 (s, 1H), 7.88 (d, *J* = 8.0 Hz, 2H), 7.66 (t, *J* = 8.5 Hz, 1H), 7.58 (t, *J* = 7.5 Hz, 1H), 7.47 (tt, *J* = 7.5, 1.4 Hz, 1H), 7.13 (d, *J* = 7.9 Hz, 2H), 7.05 – 7.00 (m, 1H), 6.91 – 6.87 (m, 1H), 6.84 – 6.80 (m, 2H), 6.64 (d, *J* = 8.5 Hz, 2H), 4.59 (hept, *J* = 6.0 Hz, 2H), 2.43 (s, 3H), 2.32 (s, 3H), 2.25 (q, *J* = 11.5 Hz, 2H), 1.84 – 1.60 (m, 14H), 1.28 (d, *J* = 6.0 Hz, 6H), 1.03 (d, *J* = 6.0 Hz, 6H), 0.88 (t, *J* = 7.1 Hz, 6H); ^{31}P NMR (162 MHz, $CDCl_3$) δ 42.6; ^{13}C NMR (101 MHz, $CDCl_3$) δ 171.8, 171.7, 160.4, 143.9, 143.6, 143.5, 139.0, 136.7, 134.8, 132.8, 132.3, 128.6, 128.5, 126.9, 126.3, 126.2, 121.0, 109.4, 109.3, 106.7, 106.5, 106.3, 106.2, 71.9, 71.8, 33.1, 33.0, 32.8, 32.7, 29.0, 28.3, 26.9, 25.9, 25.8, 22.6, 22.3, 22.2, 22.1, 22.0, 21.8, 21.6, 21.5, 21.4, 21.3; (ESI$^+$) *m/z* of [M-OTs]$^+$ detected: 706.2664, expected for $C_{45}H_{58}NO_6PPdS$: 706.2641; ν̄max (thin film)/cm^{-1} 2974 (w), 2928 (m), 2857 (w), 1587 (m), 1454 (s), 1388 (m), 1256 (m), 1221 (m), 1175 (s), 1104 (s), 1028 (s), 1012 (s), 753 (m), 728 (s), 682 (s).

Synthesis of 10

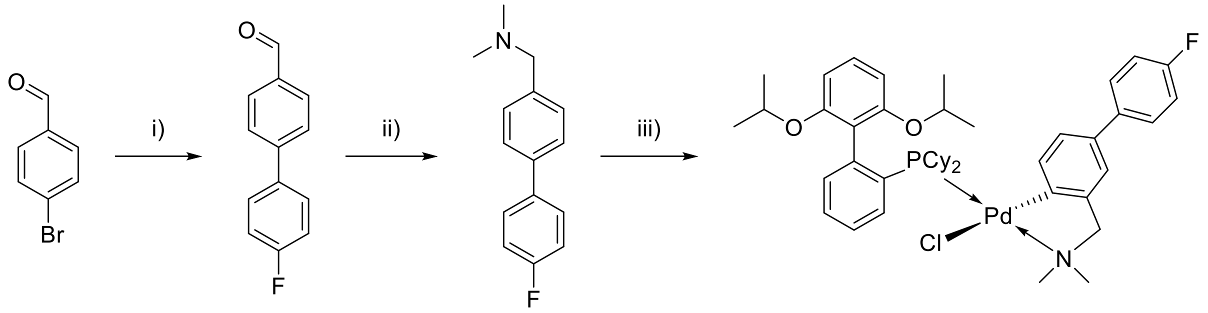

Scheme 1: Reagents and conditions: i) 4-fluoroboronic acid (1.2 equiv.), $Pd(PPh_3)_4$ (4.0 mol%), aqueous Na_2CO_3 (2.0 M), toluene/EtOH, Ar, reflux, 16 h; ii) $NaBH(OAc)_3$ (1.5 equiv.), $HNMe_2$ (1.5 equiv.), AcOH (cat.), DCE, rt, 2.5 hr; iii) $PdCl_2$ (1.0 equiv.), MeOH, rt, 1 h. ***then*** *RuPhos (1.0 equiv.), CH_2Cl_2, reflux, 3 h.*

4-(4-fluorophenyl)benzaldehyde

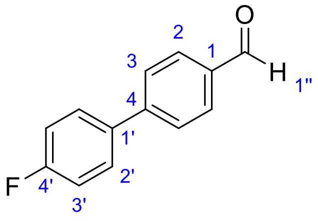

To a solution of 4-bromobenzaldehyde (460 mg, 2.5 mmol, 1.0 equiv.) in toluene (17.5 mL) was added aqueous Na_2CO_3 (2.0 M, 8.0 mL), and a solution of 4-fluorophenyl boronic acid (420 mg, 3.0 mmol, 1.2 equiv.) in EtOH (9.6 mL). The reaction mixture was purged with Ar, and $Pd(PPh_3)_4$ (120mg, 0.10 mmol, 4.0 mol%) was added, and the reaction mixture was heated to reflux overnight. After allowing the mixture to cool, EtOAc (10 mL) and H_2O (10 mL) were added, and the organics were separated. The aqueous components were extracted with EtOAc (2 × 10 mL), and the combined organics were filtered through Celite® and concentrated *in vacuo*. The crude product was purified using flash column chromatography (neat CH_2Cl_2) to yield the **title compound** (384 mg, 77%) as a colourless solid: R_f 0.55 (petroleum ether 40 – 60/CH_2Cl_2 50:50); m.p. 78 – 79 °C from CH_2Cl_2 [lit.[2] 72 – 74 °C, lit.[3] 79 – 79 °C]; ^{1}H NMR (400 MHz, Chloroform-*d*) δ 10.06 (s, 1H, H-1’’), 7.95 (d, J = 8.3 Hz, 2H, H-2), 7.71 (d,

J = 8.3 Hz, 2H, H-3), 7.67 – 7.55 (m, 2H, H-3'), 7.23 – 7.06 (m, 2H, H-2'); ^{19}F NMR (376 MHz, Chloroform-*d*) δ -113.58. The spectral data are in agreement with reported literature values.[3]

***N,N*-dimethyl-4-(4-fluorophenyl)benzylamine**

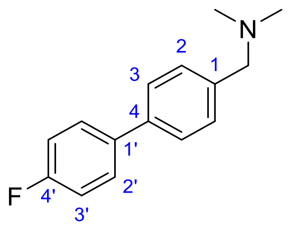

4-(4-fluorophenyl)benzaldehyde (150 mg, 0.75 mmol, 1.0 equiv.), dimethylamine (2.0 M in THF, 0.58 mL, 1.16 mmol, 1.6 equiv.), sodium triacetoxyborohydride (240 mg, 1.1 mmol, 1.5 equiv.) and glacial acetic acid (2 drops) were combined in DCE (5 mL). The resulting mixture was stirred at rt for 2.5 hr, after which aqueous sat. $NaHCO_3$ (20 mL) was added. The mixture was separated, and the aqueous components were extracted with CH_2Cl_2 (3 × 10 mL). The combined organics were washed with brine (20 mL), dried (Na_2SO_4) and concentrated *in vacuo*. The resulting crude oil was subjected to flash column chromatography (gradient up to 2% MeOH in CH_2Cl_2) to yield the **title compound** (162 mg, 95%) as a light brown oil: R_f 0.44 (CH_2Cl_2/MeOH 98:2); ^{1}H NMR (400 MHz, Chloroform-*d*) δ 7.55 (dd, J = 8.8, 5.3 Hz, 2H, H-3'), 7.50 (d, J = 8.3 Hz, 2H, H-2), 7.37 (d, J = 8.3 Hz, 2H, H-3), 7.12 (t, J = 8.8 Hz, 2H, H-2'), 3.46 (s, 2H, CH_2), 2.27 (s, 6H, CH_3); ^{19}F NMR (376 MHz, Chloroform-*d*) δ -116.00; ^{13}C NMR (101 MHz, Chloroform-*d*) δ 162.55 (d, J = 246.2 Hz, C-4'), 139.27, 137.68, 137.17 (d, J = 3.3 Hz, C-1'), 129.78, 128.71 (d, J = 8.0 Hz, C-2'), 127.03, 115.73 (d, J = 21.4 Hz, C-3'), 64.04; 45.43; (ESI$^+$) *m/z* of $[M+H]^+$ detected: 230.1344, expected for $C_{15}H_{16}FN$: 230.1340; ṽmax (thin film)/cm^{-1} 2928 (C-H, w), 1498 (s), 1224 (s), 1021 (m), 839 (s), 802 (s).

10

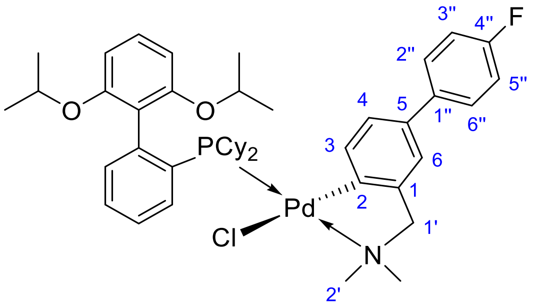

N,N-dimethyl-4-(4-fluorophenyl)benzylamine (81 mg, 0.69 mmol, 1.0 equiv.) and $PdCl_2$ (159 mg, 0.69 mmol, 1.0 equiv.) were combined in MeOH and stirred for 16 h. The reaction mixture was concentrated and dissolved in CH_2Cl_2, filtered through a column of silica, and concentrated *in vacuo*. The crude intermediate (43 mg) was then combined with RuPhos (59 mg, 0.12 mmol, 2.0 equiv. to intermediate) in CH_2Cl_2 (5 mL) and refluxed for 3 h. The solvent was removed *in vacuo*, MTBE (500 µL) and pentane (2 mL) were added, and the resulting suspension was placed in a – 20 °C freezer for 15 min. After removal from the freezer, the supernatant was pipetted off, and the solid was washed with pentane (2 × 2 mL) to give the **title compound** (43 mg, 7%); m.p. decomposes at 200 °C; ^{1}H NMR (500 MHz, Chloroform-*d*, 318 K) δ 7.81 (dd, *J* = 13.6, 8.0 Hz, 1H), 7.42 (t, *J* = 7.6 Hz, 1H), 7.33 – 7.22 (m, 1H), 7.17 (d, *J* = 7.7 Hz, 1H), 7.11 (t, *J* = 7.7 Hz, 1H), 6.95 (t, *J* = 6.7 Hz, 2H), 6.83 (t, *J* = 8.5 Hz, 2H), 6.76 (d, *J* = 5.2 Hz, 1H), 6.66 (t, *J* = 6.8 Hz, 2H), 6.56 (d, *J* = 8.4 Hz, 2H), 4.57 – 4.25 (m, 2H), 2.76 (s, 6H), 2.56 (s, 2H), 1.70 (s, 3H), 1.55 (s, 5H), 1.37 – 0.59 (m, 22H); ^{31}P NMR (202 MHz, $CDCl_3$) δ 71.41; ^{19}F NMR (376 MHz, $CDCl_3$) δ -117.91; (ESI$^+$) *m/z* [M-Cl]$^+$ detected: 796.3248, expected for $C_{45}H_{58}FClNO_2PPd$: 796.3240; ṽmax (thin film)/cm^{-1} 2922 (C-H, m), 1509 (m), 1456 (s), 1244 (m), 1111 (s), 1056 (s), 807 (s), 744 (m).

Synthesis of 11

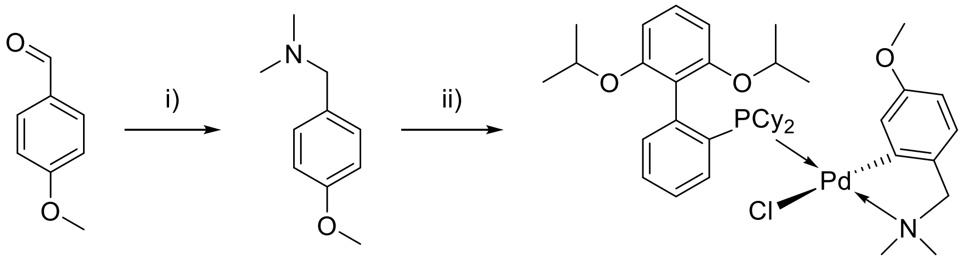

Scheme 2: Reagents and conditions: i) NaBH(OAc)$_3$ (1.5 equiv.), HNMe$_2$ (1.5 equiv.), CH$_2$Cl$_2$, rt, 16 h; ii) PdCl$_2$ (1.0 equiv.), MeOH, rt, 1 h. ***then*** *RuPhos (1.0 equiv.), CH$_2$Cl$_2$, 72 h.*

N,N-dimethyl-4-methoxylbenzylamine 3c

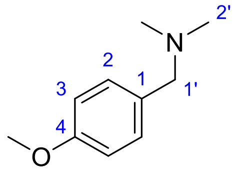

4-Methoxybenzaldehyde (110 µL, 1.0 mmol, 1.0 equiv.), dimethylamine (2.0 M in THF, 0.75 mL, 1.5 mmol, 1.5 equiv.), and sodium triacetoxyborohydride (320 mg, 1.5 mmol, 1.5 equiv.), combined in CH_2Cl_2 (20 mL) and stirred for 16 h. After which, 40 mL sat. aq. $NaHCO_3$ was added, and the aqueous component was separated and extracted with CH_2Cl_2 (3 × 20 mL). The combined organics were then washed with brine (40 mL), dried (Na_2SO_4) and concentrated *in vacuo*. The crude residue was subjected to flash column chromatography (gradient up to 2:98 MeOH/CH_2Cl_2 with 1% added NEt_3) to yield the **title compound** (118 mg, 71%) as a colourless liquid: R_f 0.21 (1:2:97 NEt_3/MeOH/CH_2Cl_2); ^{1}H NMR (400 MHz, Chloroform-*d*) δ 7.21 (d, *J* = 8.6 Hz, 2H, H-3), 6.86 (d, *J* = 8.6 Hz, 2H, H-2), 3.80 (s, 3H, OCH_3), 3.36 (s, 2H, H-1'), 2.22 (s, 6H, H-2'); ^{13}C NMR (101 MHz, $CDCl_3$) δ 158.8, 131.1, 130.4, 113.7, 63.8, 55.4, 45.3.. The spectral data are in agreement with reported literature values.[4]

11

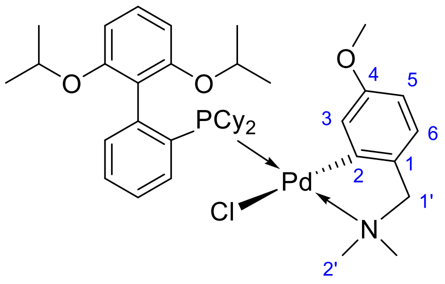

N,*N*-Dimethyl-4-methoxylbenzylamine (20 mg, 0.12 mmol, 1.0 equiv.) and $PdCl_2$ (21 mg, 0.12 mmol, 1.0 equiv.) were combined in MeOH (500 µL) and stirred for 24 h. After which, the solid formed was isolated *via* gravity filtration, washed with MeOH (5 mL) and H_2O (5 mL) and dried under high vacuum to yield a light green solid (13 mg).

This intermediate (13 mg, 0.021 mmol, 1.0 equiv.) was combined with RuPhos (20 mg, 0.042 mmol, 2.0 equiv.) in CH_2Cl_2 (1 mL) and stirred for 72 h. After which, the reaction mixture was concentrated and washed with pentane (3 × 1 mL) to yield the **title compound** (11 mg) as a colourless solid: ^{31}P NMR (162 MHz, $CDCl_3$, 328 K) δ 66.87; (ESI$^+$) *m/z* of $[M]^+$ detected: 767.2855, expected for $C_{40}H_{57}ClNO_3PPd$: 767.2815.

Synthesis of 12

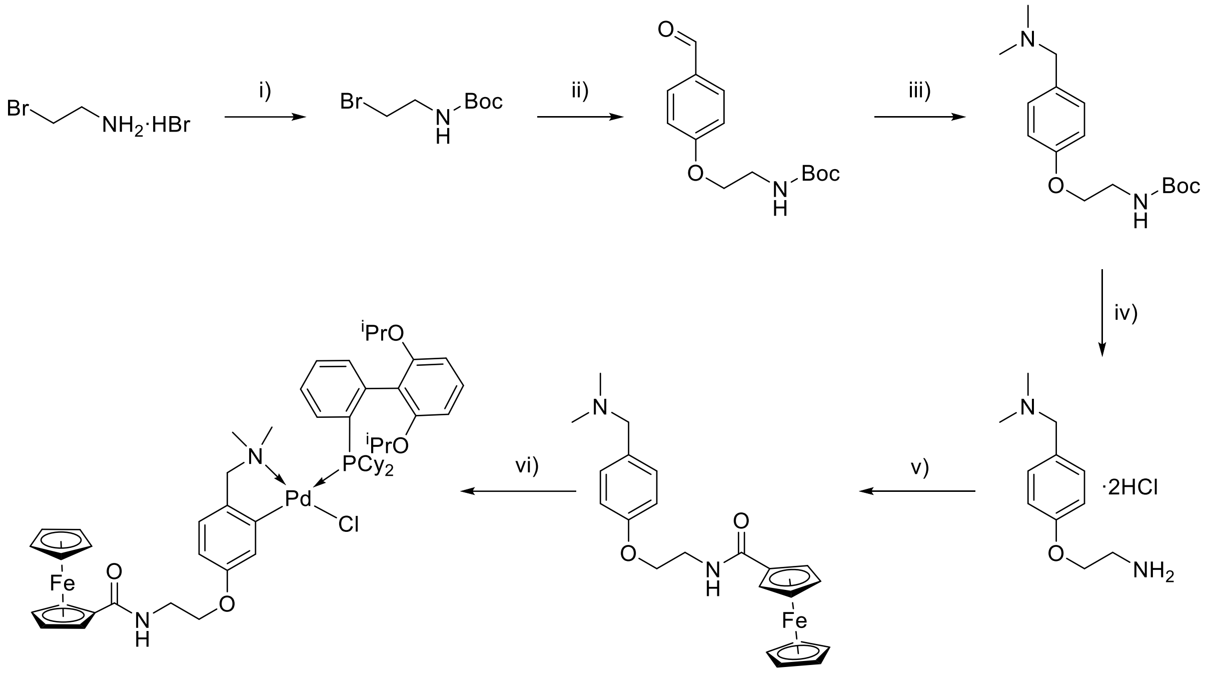

Scheme 3: Reagents and conditions: i) Boc_2O (1.1 equiv.), DIPEA (2.0 equiv.), THF, 0 °C – rt, 16 h; ii) 4-hydroxybenzaldehyde (1.0 equiv.), K_2CO_3 (2.0 equiv.), NaI (1.0 equiv.), DMF, Ar, 80 °C, 90 min; iii) $NaBH(OAc)_3$ (1.5 equiv.), $HNMe_2$ (1.5 equiv.), HOAc (cat.), CH_2Cl_2, rt, 16 h; iv) HCl in dioxane (4.0 M), 0 °C – rt, N_2, 30 min; v) EDC.HCl (1.1 equiv.), HOBt hydrate (1.1 equiv.), Fc-COOH (1.0 equiv.), NEt_3 (2.0 equiv.), CH_2Cl_2, rt, 3 h; vi) $PdCl_2$ (1.0 equiv.), NaOAc (1.0 equiv.), NaCl (2.0 equiv.), MeOH, rt, 24 h, ***then*** *RuPhos (1 equiv.), CH_2Cl_2, rt, 1 h.*

2-(Boc-amino)ethyl bromide

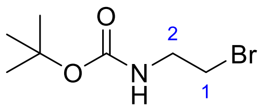

2-(Boc-amino)ethyl bromide was prepared according to a literature procedure with minor modifications.[6] 2-Bromoethylamine hydrobromide (1.02 g, 5.0 mmol, 1.0 equiv.) and di-tert-butyl decarbonate (1.20 g, 5.5 mmol, 1.1 equiv.) were combined in THF (15 mL) and cooled to 0 °C. *N,N*-Diisopropylethylamine (1.8 mL, 10 mmol, 2.0 equiv.) was added dropwise, the reaction mixture was warmed to rt, and stirred for 16 h. The reaction mixture was concentrated *in vacuo*. The residue was dissolved in EtOAc (20 mL), washed with K_2CO_3 (10%, aq., 20 mL) and brine (20 mL), and dried with Na_2SO_4 before being concentrated *in vacuo*. The crude

product was subjected to flash column chromatography (gradient up to 10% Et_2O in petroleum ether) to yield the **title compound** (916 mg, 82%) as a colourless oil: R_f 0.16 (Et_2O/petroleum ether 5:95), ^{1}H NMR (400 MHz, Chloroform-*d*) δ 4.94 (s, 1H, NH), 3.59 – 3.50 (m, 2H, C-2), 3.45 (t, J = 5.8 Hz, 2H, C-1), 1.45 (s, 9H, CH_3). The spectral data are in agreement with reported literature values.[5]

***Tert*-butyl(2-(4-formylphenoxy)ethyl)carbamate**

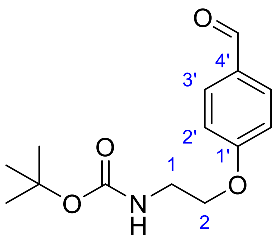

2-(Boc-amino)ethyl bromide (880 g, 3.9 mmol, 1.5 equiv.), 4-hydroxybenzaldehyde (320 mg, 2.6 mmol, 1.0 equiv.,), NaI (390 mg, 2.6 mmol, 1.0 equiv.), and K_2CO_3 (730 mg, 5.2 mmol, 2.0 equiv.) were combined in DMF (10 mL) under Ar and heated to 80 °C. After 90 min, 4-hydroxybenzaldehyde (80 mg, 0.65 mmol, 0.25 equiv.) and K_2CO_3 (180 mg, 1.3 mmol, 0.5 equiv.) were added, and the mixture stirred for another 2 h. The reaction mixture was cooled, diluted with H_2O (30 mL) and extracted with Et_2O (3 × 25 mL). The combined organics were washed with aqueous LiCl (0.5 M, 2 × 25 mL), and brine (50 mL) before being dried with Na_2SO_4 and concentrated *in vacuo* to yield the **title compound** (542 mg, 63%) as an off-white solid: m.p. (62 – 64 °C); ^{1}H NMR (400 MHz, Chloroform-*d*) δ 9.89 (s, 1H), 7.84 (d, J = 8.8 Hz, 2H), 7.00 (d, J = 8.7 Hz, 2H), 4.97 (s, 1H), 4.11 (t, J = 5.1 Hz, 2H), 3.65 – 3.53 (m, 2H), 1.45 (s, 9H). The spectral data are in agreement with reported literature values.[6]

***N*,*N*-dimethyl-4-((*N'*-boc)2-aminoethoxy)benzylamine**

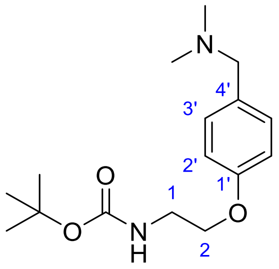

Tert-butyl(2-(4-formylphenoxy)ethyl)carbamate (620 mg, 2.3 mmol, 1.0 equiv.), dimethylamine (2.0 M in THF, 1.75 mL, 3.5 mmol, 1.5 equiv.), sodium triacetoxyborohydride (740 mg, 3.5 mmol, 1.5 equiv.), and glacial acetic acid (5 drops) were combined in CH_2Cl_2 (20 mL) under an atmosphere of N_2. After 18 h, the reaction mixture was concentrated *in vacuo*, and sat. aq. $NaHCO_3$ (50 mL) was added. The aqueous suspension was extracted with CH_2Cl_2 (3 × 40 mL), and the combined organics were dried (Na_2SO_4), and concentrated *in vacuo*. The crude product was subjected to flash column chromatography (gradient up to 1:99 NEt_3/toluene) to yield the **title compound** (394 mg, 58 %) as an off-white solid: m.p. (105 – 107 °C); ^{1}H NMR (500 MHz, Chloroform-*d*) δ 7.21 (d, *J* = 8.6 Hz, 2H, H-2'), 6.84 (d, *J* = 8.6 Hz, 2H, H-3'), 4.99 (s, br, 1H, NH), 4.01 (t, *J* = 5.1 Hz, 2H, H-2), 3.55 – 3.50 (m, 2H, H-1), 3.35 (s, H, 2H, CH_2), 2.22 (s, 6H, $N(CH_3)_2$), 1.45 (s, 9H, $C(CH_3)_3$); (ESI^+) *m/z* of $[M+H]^+$ detected: 295.2021, expected for $C_{16}H_{26}N_2O_3$: 295.2016; ^{13}C NMR (101 MHz, $CDCl_3$) δ 157.83, 131.54, 130.47, 114.31, 67.33, 63.83, 45.35, 40.30, 28.55; v̄max (thin film)/cm^{-1} 3203 (N-H, s), 2945 (N-H, s), 2819 (C-H, s), 2775 (C-H, s), 1705 (C=O, s), 1613 (C-H bend, w), 1546 (m), 1514 (s), 1451 (m), 1368 (m), 1240 (s), 1173 (s), 1154 (s), 1110 (m).

N,N-dimethyl-4-(2-aminoethoxy)benzylamine dihydrochloride

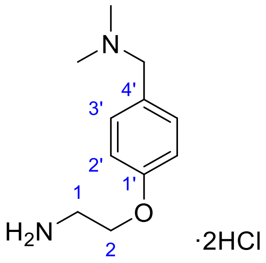

N,*N*-dimethyl-4-((*N'*-boc)2-aminoethoxy)benzylamine (150 mg, 0.50 mmol, 1.0 equiv.) was added to a solution of HCl/dioxane (4.0 M, 10 mL) that had been cooled to 0 °C under N_2. The reaction mixture was allowed to warm to rt and stirred for 30 min before being concentrated *in vacuo* to yield the **title compound** (130 mg, >99%) as an off-white solid; m.p. (decomposes at 198 °C); R_f 0.32 (5:95:1 MeOH/CH_2Cl_2/NEt_3); ^{1}H NMR (400 MHz, Methanol-d_4) δ 7.50 (d, J = 8.7 Hz, 2H), 7.13 (d, J = 8.7 Hz, 2H), 4.33 – 4.23 (m, 4H), 3.40 (t, J = 5.0 Hz, 2H), 2.83 (s, 6H); ^{13}C NMR (101 MHz, MeOD) δ 160.80, 133.74, 124.00, 116.38, 65.46, 61.58, 42.67, 40.25; (ESI$^+$) *m/z* of $[M+H]^+$ detected: 195.1494, expected for $C_{11}H_{18}N_2O$: 195.1492; ν̄max (thin film)/cm^{-1} 2953 (N-H, br, s), 2827 (C-H, s), 2771 (C-H, s), 1709 (s), 1613 (w), 1550 (w), 1514 (s), 1459 (w), 1368 (w), 1280 (m), 1237 (s), 1169 (m), 1150 (m).

N,N-dimethyl-4-(N'-(ferrocene amide)-2-aminoethoxy)benzylamine

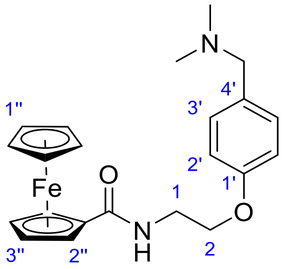

N,*N*-dimethyl-4-(2-aminoethoxy)benzylamine dihydrochloride (53 mg, 0.20 mmol, 1.0 equiv.) and NEt_3 (58 µL, 0.40 mmol, 2.0 equiv.) were combined in CH_2Cl_2 (5 mL). After stirring for 5 min, ferrocene carboxylic acid (46 mg, 0.20 mmol, 1.0 equiv.), HOBt hydrate (34 mg,

0.22 mmol, 1.1 equiv.) and EDC hydrochloride (42 mg, 0.22 mmol, 1.1 equiv.) were added, and the resulting solution was stirred for 3 h. The reaction mixture was diluted with H_2O (30 mL) and extracted with CH_2Cl_2 (2 × 30 mL). The combined organics were washed with brine (50 mL), dried (Na_2SO_4) and concentrated *in vacuo*. The crude residue was subjected to flash column chromatography (1:99 NEt_3/CH_2Cl_2) to yield the **title compound** (53 mg, 65%) as an orange solid: m.p. (100 – 102 °C); ^{1}H NMR (400 MHz, Chloroform-*d*) δ 7.15 (d, *J* = 8.6 Hz, 2H, H-3'), 6.83 (d, *J* = 8.6 Hz, 2H, H-2'), 6.08 (s, 1H, NH), 4.60 (t, *J* = 2.0 Hz, 2H, H-1), 4.27 (t, *J* = 2.0 Hz, 2H, H-2), 4.09 (s, 4H, H-1''), 4.06 (t, *J* = 5.0 Hz, 2H, H-3''), 3.75 – 3.68 (m, 2H, H-2''), 3.28 (s, 2H, NCH_2), 2.14 (s, 6H, NCH_3); ^{13}C NMR (101 MHz, $CDCl_3$) δ 170.66, 157.81, 131.76, 130.58, 114.33, 76.03, 70.59, 69.89, 68.30, 67.13, 63.82, 45.36, 39.25; (ESI$^+$) *m/z* of $[M+H]^+$ detected: 405.1462, expected for $C_{22}H_{26}FeN_2O_2$: 405.1463; $\bar{v}$max (thin film)/cm^{-1} 3290 (N-H, br, s), 2934 (C-H, br, m), 2815 (C-H, s), 2763 (C-H, s), 1625 (C=O, s), 1538 (s), 1506 (s), 1451 (m), 1296 (m), 1237 (s), 1177 (m), 1106 (m), 1023 (m), 809 (s).

12

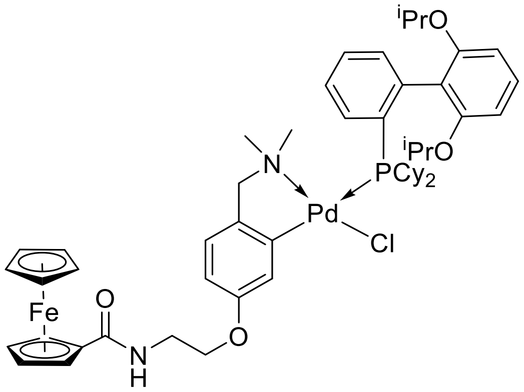

N,N-dimethyl-4-(*N'*-(ferrocene amide)-2-aminoethoxy)benzylamine (20 mg, 50 µmol, 1.0 equiv.), $PdCl_2$ (9 mg, 50 µmol, 1.0 equiv.), NaCl (6 mg, 100 µmol, 2.0 equiv.), and NaOAc (4 mg, 50 µmol, 1.0 equiv.) were combined in MeOH (500 µL) and stirred for 24 h. The resulting solid formed was isolated *via* gravity filtration, washed with H_2O (3 mL) and MeOH (2 × 3 mL), and dried under high vacuum to an orange solid (16 mg).

This intermediate (16 mg, 15 µmol, 1.0 equiv.) was combined with RuPhos (14 mg, 30 µmol, 2.0 equiv.) in CH_2Cl_2 (2 mL) and stirred for 1 h. After removal of the solvent, the resulting solid was dissolved in MTBE/Pentane (1:4, 2 mL) and placed in the freezer for 30 min. The supernatant was pipetted off and the solid was washed with pentane (2 × 2mL) to yield the **title compound** (23 mg, 60% yield over two steps) as an orange solid: ^{31}P NMR (162 MHz, $CDCl_3$) δ 66.87; (ESI$^+$) *m/z* of $[M-Cl]^+$ detected: 973.3322, expected for $C_{52}H_{68}ClFeN_2O_4PPd$: 973.3330.

Synthesis of 13

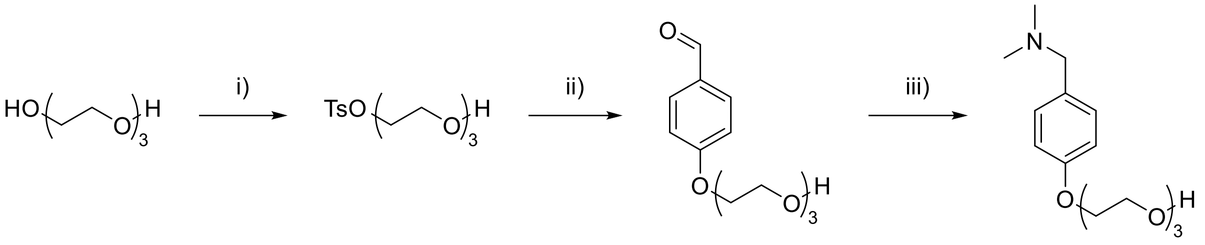

Scheme 4: Reagents and conditions: i) TsCl (0.25 equiv.), DMAP (0.5 mol%), NEt_3 (0.25 equiv.), CH_2Cl_2, 0 °C – rt, 20 h; ii) 4-hydroxybenzaldehyde (1.3 equiv.), K_2CO_3 (2.0 equiv.), DMF, 100 °C, 2.5 h; iii) $NaBH(OAc)_3$ (1.5 equiv.), $HNMe_2$ (1.5 equiv.), CH_2Cl_2, rt, 72 h.

2-(2-(2-Hydroxyethoxy)ethoxy)ethyl 4-methylbenzenesulfonate

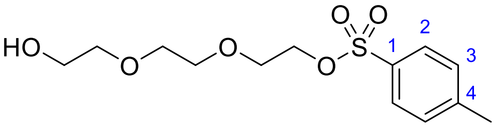

4-Toluenesulfonyl chloride (950 mg, 5.0 mmol, 1.0 equiv.) was added to a solution of triethylene glycol (2.8 mL, 21 mmol, 4.1 equiv.), 4-dimethylaminopyridine (12 mg, 0.10 mmol, 0.020 equiv.) and NEt_3 (0.80 mL, 5.5 mmol, 1.1 equiv.) in CH_2Cl_2 (25 mL) that had been cooled to 0 °C. After 20 h, the reaction mixture was diluted with CH_2Cl_2 (40 mL), washed with aqueous HCl (1 M, 40 mL), H_2O (40 mL) and brine (40 mL). The organic component was dried (Na_2SO_4) and concentrated *in vacuo*. The crude residue was then subjected to flash column chromatography (4:1 EtOAc/Petrol ether 40 – 60) to yield the **title compound** (950 mg, 56%) as a clear oil: R_f 0.24 (4:1 EtOAc/Petrol ether 40 – 60); ^{1}H NMR (500 MHz, Chloroform-*d*) δ 7.80 (d, *J* = 8.3 Hz, 2H), 7.35 – 7.33 (m, 2H), 4.17 (t, *J* = 4.7 Hz, 2H), 3.74 – 3.68 (m, 4H), 3.61 (s, 4H), 3.59 – 3.56 (m, 2H), 2.45 (s, 3H). The spectral data are in agreement with reported literature values.[7]

4-(2-(2-(2-Hydroxyethoxy)ethoxy)ethoxy)benzaldehyde

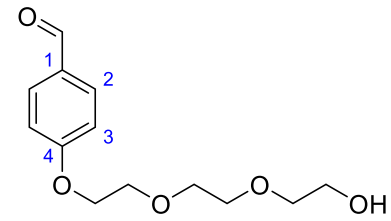

2-(2-(2-Hydroxyethoxy)ethoxy)ethyl 4-methylbenzenesulfonate (940 mg, 3.1 mmol, 1.0 equiv.) was added to a solution of 4-hydroxybenzaldehyde (490 mg, 4.0 mmol, 1.3 equiv.) and K_2CO_3 (860 mg, 6.2 mmol, 2.0 equiv.) in anhydrous *N*,*N*-dimethylformamide (10 mL). The resulting solution was heated to 100 °C for 2.5 h before being diluted with aqueous HCl (1 M, 50 mL), and extracted with CH_2Cl_2 (3 × 50 mL). The combined organics were washed with aqueous LiCl (0.5 M, 3 × 50 mL), dried (Na_2SO_4) and concentrated *in vacuo*. The resulting crude residue was subjected to flash column chromatography (gradient from 4:1 EtOAc/Petrol ether 40 – 60 to 100% EtOAc) to yield the **title compound** (580 mg, 73%) as a colourless oil: ^{1}H NMR (400 MHz, Chloroform-*d*) δ 9.89 (s, 1H), 7.83 (d, *J* = 8.7 Hz, 2H), 7.03 (d, *J* = 8.7 Hz, 2H), 4.24 – 4.20 (m, 2H), 3.92 – 3.88 (m, 2H), 3.77 – 3.69 (m, 6H), 3.65 – 3.60 (m, 2H). The spectral data are in agreement with reported literature values.[7]

***N*,*N*-dimethyl-4-(2-(2-(2-Hydroxyethoxy)ethoxy)ethoxy)benzylamine 3d**

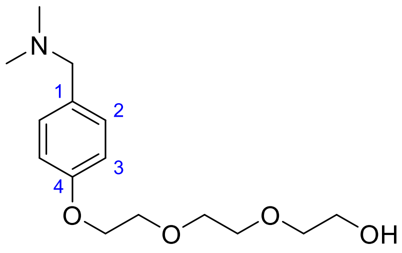

4-(2-(2-(2-Hydroxyethoxy)ethoxy)ethoxy)benzaldehyde (510 mg, 2.0 mmol, 1.0 equiv.), dimethylamine (2.0 M in THF, 1.5 mL, 3.0 mmol, 1.5 equiv.) and $NaBH(OAc)_3$ (640 mg,

3.0 mmol, 1.5 equiv.) were combined in CH_2Cl_2 (20 mL) and stirred for 72 h at rt. After which, the reaction mixture was concentrated *in vacuo*, sat. aq. $NaHCO_3$ (60 mL) was added, and the aqueous mixture was extracted with $CH_2Cl_2/^iPrOH$ (2:1, 60 mL). The organic component was washed with H_2O (60 mL), brine (60 mL), dried (Na_2SO_4), and concentrated *in vacuo*. The crude material was then purified *via* Strata® SCX (55 µm, 70 Å) 5g cartridge (eluted with CH_2Cl_2/MeOH 90:10 then 2.0 M NH_3 in EtOH) to yield the **title compound** (461 mg, 85%) as a pale yellow oil: ^1H NMR (400 MHz, Chloroform-*d*) δ 7.21 (d, *J* = 8.6 Hz, 2H), 6.87 (d, *J* = 8.6 Hz, 1H), 4.15 – 4.11 (m, 2H), 3.88 – 3.85 (m, 2H), 3.76 – 3.68 (m, 6H), 3.63 – 3.60 (m, 2H), 3.38 (s, 2H), 2.23 (s, 5H); ^{13}C NMR (101 MHz, $CDCl_3$) δ 158.07, 130.61, 130.52, 114.50, 72.63, 70.92, 70.50, 69.87, 67.48, 63.61, 61.82, 45.08; $\bar{\nu}$max (thin film)/cm^{-1} 3330 (O-H, br, w), 2930 (C-H, s), 2862 (C-H, s), 2819 (C-H, s), 2767 (C-H, s), 1613 (m), 1514 (s), 1451 (m), 1372 (w), 1237 (s), 1181 (w), 1122 (s), 1058 (C-O, s), 928 (w), 852 (m), 809 (m).

13

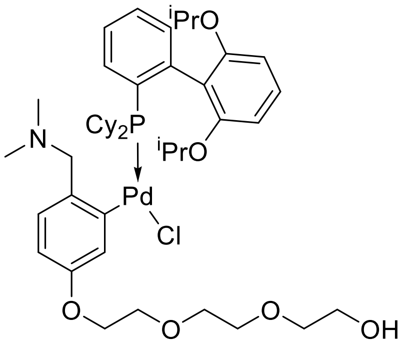

N,N-dimethyl-4-(2-(2-(2-Hydroxyethoxy)ethoxy)ethoxy)benzylamine **3d** (21 mg, 74 µmol, 1.0 equiv.), NEt_3 (11 µL, 74 µmol, 1.0 equiv.) and Na_2PdCl_4 (22 mg, 74 µmol, 1.0 equiv.) were combined in MeOH (500 µL) and stirred for 1 h before being concentrated. The residue was suspended in CH_2Cl_2 (1 mL) and filtered. RuPhos (35 mg, 74 µmol, 1.0 equiv.) was added to the filtrate and the mixture was stirred for 30 min before being concentrated. MTBE/pentane (1:4, 2 mL) was added to the residue, and the suspension was placed in the freezer for 2 h. The

supernatant was pipetted off, the mixture suspended in Et_2O and filtered to remove any NEt_3HCl. The Et_2O was then removed *in vacuo* to yield the **title compound** (47 mg, 72%) as a crystalline solid: ^{31}P NMR (162 MHz, $CDCl_3$) δ 34.01;(ESI^+) *m/z* of $[M]^+$ detected: 850.3744, expected for $C_{45}H_{67}NO_6PPd$: 850.3756.

Synthesis of 14

***N,N*-dimethyl-4-fluorobenzylamine 14**

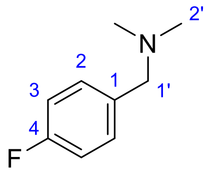

4-Fluorobenzaldehyde (210 µL, 2.00 mmol, 1.0 equiv.), dimethylamine (2.0 M in THF, 1.5 mL, 3.0 mmol, 1.5 equiv.), and sodium triacetoxyborohydride (640 mg, 3.00 mmol, 1.5 equiv.) were combined in CH_2Cl_2 (25 mL). The resulting mixture was stirred at rt for 3 hr, after which the mixture was concentrated *in vacuo*, and aqueous NaOH (1 M, 40 mL) was added. The mixture was separated, and the aqueous components were extracted with Et_2O (3 × 40 mL). The combined organics were dried (Na_2SO_4), concentrated *in vacuo,* and subjected to flash column chromatography (CH_2Cl_2/MeOH/Et_3N 98:1:1) to yield the **title compound** (256 mg, 84%) as a light brown oil: R_f 0.56 (CH_2Cl_2/MeOH/Et_3N 98:1:1); ^{1}H NMR (400 MHz, Chloroform-*d*) δ 7.29 (dd, J_{H-H} = 8.6, J_{H-F} = 5.6 Hz, 2H, H-2), 7.01 (dd, J_{H-H} = 8.6 Hz, J_{H-F} = 8.7 Hz 2H, H-3), 3.44 (s, 2H, H-1'), 2.26 (s, 6H, H-2'); ^{19}F NMR (376 MHz, $CDCl_3$) δ -115.47. The spectral data are in agreement with literature values.[8]

Synthesis of 17

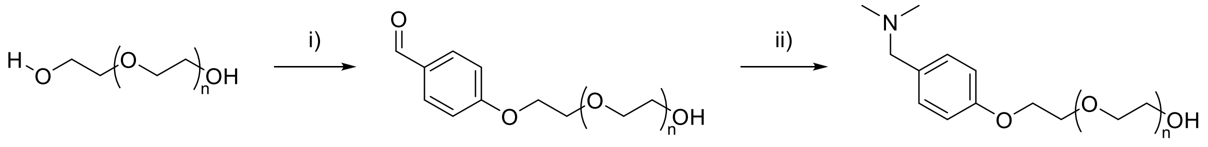

*Scheme 5: Reagents and conditions: i) i) TsCl (0.25 equiv.), DMAP (0.5 mol%), NEt_3 (0.25 equiv.), CH_2Cl_2, 0 °C – rt, 20 h, **then** 4-hydroxybenzaldehyde (1.3 equiv.), K_2CO_3 (2.0 equiv.), DMF, 100 °C, 90 min; ii) $NaBH(OAc)_3$ (1.5 equiv.), $HNMe_2$ (1.5 equiv.), CH_2Cl_2, rt, 72 h.*

4-(PEG-400)benzaldehyde

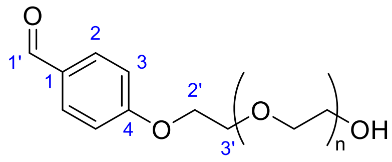

4-Toluenesulfonyl chloride (950 mg, 5.0 mmol, 1.0 equiv.) was added to a solution of PEG-400 (7.3 mL, 21 mmol, 4.1 equiv.), 4-dimethylaminopyridine (12 mg, 0.10 mmol, 0.020 equiv.) and NEt_3 (0.80 mL, 5.5 mmol, 1.1 equiv.) in CH_2Cl_2 (25 mL) that had been cooled to 0 °C. After 20 h, the reaction mixture was diluted with CH_2Cl_2 (40 mL), washed with aqueous HCl (1 M, 40 mL), H_2O (40 mL) and brine (40 mL). The organic component was dried (Na_2SO_4) and concentrated *in vacuo* to yield PEG-400-OTs (2.46 g) as a colourless oil which was used without further purification.

4-Hydroxybenzaldehyde (600 mg, 4.9 mmol, 1.3 equiv.), PEG-400-OTs (2.1 g, 3.8 mmol, 1.0 equiv.) and K_2CO_3 (1.1 g, 7.6 mmol, 2.0 equiv.) were combined in DMF (20 mL) and heated to 100 °C for 90 min. After which, the reaction mixture was concentrated *in vacuo*, dissolved in IPA/CH_2Cl_2 (1:2, 60 mL), the organics washed with HCl (1 M, aq., 60 mL), NaOH (1 M, aq., 60 mL), LiCl (0.5 M, aq., 5 × 60 mL) to yield the **title compound** (1.86 g, 74% over 2 steps) as a colourless oil: ^{1}H NMR (400 MHz, Chloroform-*d*) δ 9.88 (s, 1H, H-1'), 7.82 (d, *J* = 8.7 Hz, 2H, H-2), 7.02 (d, *J* = 8.7 Hz, 2H, H-3), 4.23 – 4.19 (t, *J* = 4.9 Hz 2H, H-2'), 3.90 –

3.87 (t, J = 4.9 Hz, 2H, H-3'), 3.75 – 3.59 (m, 30H, -OCH_2), 2.50 (s, 1H, -OH); ^{13}C NMR (101 MHz, $CDCl_3$) δ 190.94, 163.98, 132.08, 130.18, 115.02, 72.65, 71.05, 70.77, 70.76, 70.71, 70.68, 70.47, 69.61, 67.91, 61.89; (ESI$^+$) *m/z* of $[M+H]^+$ detected: 475.2505, expected for $C_{23}H_{38}O_{10}$ (n = 7): 475.2538; ν̄max (thin film)/cm^{-1} 3485 (O-H, br, w), 2866 (C-H, br, s), 1689 (C=O, s), 1605 (s), 1574 (m), 1518 (w), 1455 (w), 1359 (w), 1300 (w), 1253 (s), 1094 (C-O, br, s)

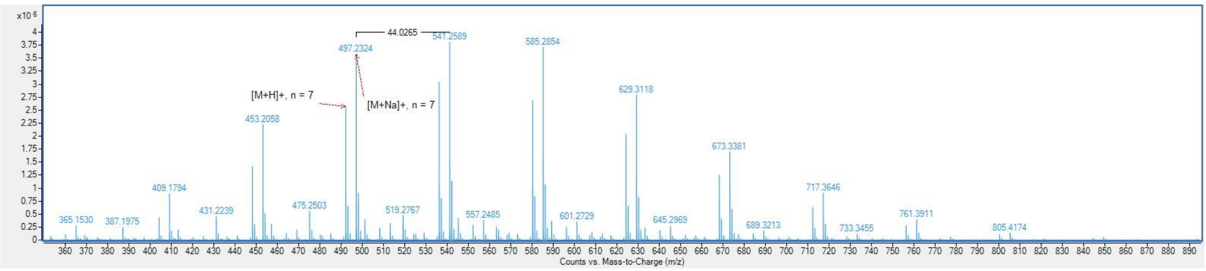

_N,N_-dimethyl(4-(PEG-400))benzylamine 17

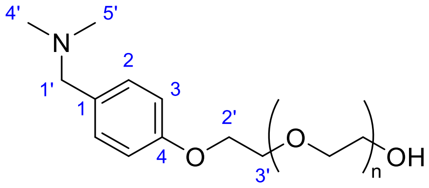

4-(PEG-400)benzaldehyde (1.1 g mg, 2.2 mmol, 1.0 equiv.), dimethylamine (2.0 M in THF, 1.7 mL, 3.3 mmol, 1.5 equiv.) and $NaBH(OAc)_3$ (700 mg, 3.3 mmol, 1.5 equiv.) were combined in DCM (20 mL) and stirred for 72 h at rt. After which, the reaction mixture was diluted with sat. aq. $NaHCO_3$ (60 mL), and the aqueous mixture was extracted with CH_2Cl_2/^{i}PrOH (2:1, 60 mL). The organic component was washed with H_2O (60 mL), brine (60 mL), dried (Na_2SO_4), and concentrated *in vacuo*. The crude material was then purified *via* Strata® SCX (55 µm, 70 Å) 5g cartridge (eluted with CH_2Cl_2/MeOH 90:10 then 2.0 M NH_3 in MeOH) to yield the **title compound** (750 mg, 63%) as a pale yellow oil: ^{1}H NMR (400 MHz, Chloroform-*d*) δ 7.19 (d, J = 8.5 Hz, 2H), 6.86 (d, J = 8.6 Hz, 2H), 4.14 – 4.09 (m, 2H), 3.86

– 3.83 (m, 2H), 3.74 – 3.58 (m, 30H), 3.36 (s, 2H), 2.22 (s, 6H); ^{13}C NMR (101 MHz, $CDCl_3$) δ 134.89, 107.55, 107.23, 91.25, 49.49, 47.72, 47.52, 47.47, 47.24, 46.66, 44.32, 40.48, 38.58, 21.95; (ESI^+) *m/z* of $[M+H]^+$ detected: 504.3166, expected for $C_{25}H_{45}NO_9$ (n = 7): 504.3167; v̄max (thin film)/cm^{-1} 3461 (O-H, br, w), 2866 (C-H, br, s), 2815 (C-H, w), 2775 (C-H, w), 1609 (w), 1506 (m), 1455 (w), 1352 (w), 1300 (w), 1241 (s), 1094 (C-O, br, s), 951 (br, w).

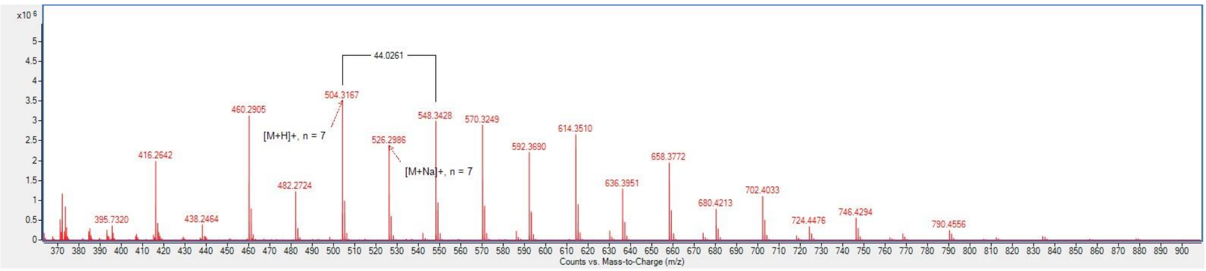

Synthesis of 18

(Dimethylaminomethyl)ferrocene 18

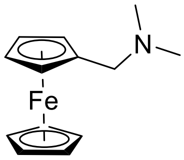

Ferrocene carboxaldehyde (210 mg, 1.0 mmol, 1.0 equiv.), dimethylamine (2.0 M in THF, 0.75 mL, 1.5 mmol, 1.5 equiv.), sodium triacetoxyborohydride (210 mg, 1.0 mmol, 1.0 equiv.) and glacial acetic acid (3 drops) were combined in DCE (10 mL) and stirred at rt. After 2 h, sodium triacetoxyborohydride (110 mg, 0.50 mmol, 0.50 equiv.) was added, and the reaction was stirred for a further 1 h at rt. After which, aqueous sat. $NaHCO_3$ (20 mL) was added, and the aqueous layer was extracted with CH_2Cl_2 (3 × 20 mL). The combined organic portions were washed with brine (30 mL), dried (Na_2SO_4) and concentrated *in vacuo*. The crude residue was subjected to flash column chromatography (gradient from 0:100 to 100:0 EtOAc/Petroleum ether with 1% NEt_3 additive) to yield the **title compound** (212 mg, 84%) as a brown liquid: R_f 0.27 (99:1 EtOAc/NEt_3); ^{1}H NMR (400 MHz, Chloroform-*d*) δ 4.17 (t, *J* = 1.8 Hz, 2H), 4.11 (m, 7H), 3.30 (s, 3H, NCH_2), 2.18 (s, 6H, NCH_3). The spectral data are in agreement with reported literature values.[8]

Synthesis of 19

***N*-Benzyl morpholine 19**

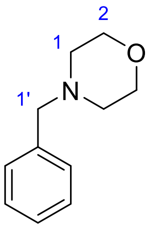

Benzyl bromide (400 µL, 3.3 mmol, 1.1 equiv.) was added dropwise to a suspension of morpholine (260 µL, 3.0 mmol, 1.0 equiv.) and K_2CO_3 (540 mg, 3.9 mmol, 1.3 equiv.) in MeCN (10 mL), and stirred for 16 h. After which, the resulting solids were filtered off, washed with EtOAc (3 × 10 mL) and the combined organics were concentrated *in vacuo*. The crude material subjected to flash column chromatography (6:1 petroleum ether/EtOAc with 1% added NEt_3) to yield the **title compound** (530 mg, >99%) as a colourless liquid: ^{1}H NMR (400 MHz, Chloroform-*d*) δ 7.34 – 7.23 (m, 5H, Ar-H), 3.75 – 3.66 (t, *J* = 4.7 Hz, 2H, H-2), 3.50 (s, 2H, H-1'), 2.44 (t, *J* = 4.7 Hz, 2H, H-1); ^{13}C NMR (101 MHz, $CDCl_3$) δ 137.90, 129.33, 128.38, 127.27, 67.17, 63.61, 53.77. The spectral data are in agreement with reported literature values.[9]

Synthesis of 20 and 21

(*R*)-(+)-*N*,*N*-Dimethyl-1-phenylethylamine 20

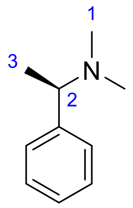

(*R*)-(+)-1-phenylethylamine (0.64 mL, 5.0 mmol, 1.0 equiv.) was added dropwise to formic acid (0.95 mL, 25 mmol, 5.0 equiv.) that had been cooled to 0 °C. After which, formaldehyde (37% in H_2O, 1.23 mL, 3.0 equiv.) was added, and the resulting mixture was heated to 90 °C for 16 h. The mixture was allowed to cool, concentrated *in vacuo*, aq. NaOH (2.0 M, 25 mL) was added, and the aqueous component was extracted with CH_2Cl_2 (3 × 40 mL). The combined organics were dried with Na_2SO_4 and concentrated *in vacuo* to yield the **title compound** (520 mg, 70%) as a pale yellow oil: ^{1}H NMR (400 MHz, Chloroform-*d*) δ 7.34 – 7.21 (m, 5H), 3.24 (q, *J* = 6.7 Hz, 1H, H-2), 2.20 (s, 6H, H-1), 1.37 (d, *J* = 6.7 Hz, 3H, H-3); $[\alpha]_D^{23}$ +40 (*c* 1.0, CH_3OH). The spectral data are in agreement with reported literature values.[10]

(*S*)-(-)-*N*,*N*-Dimethyl-1-phenylethylamine 21

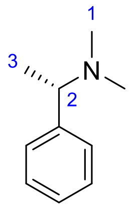

(*S*)-(-)-1-phenylethylamine (0.64 mL, 5.0 mmol, 1.0 equiv.) was added dropwise to formic acid (0.95 mL, 25 mmol, 5.0 equiv.) that had been cooled to 0 °C. After which, formaldehyde (37%

in H_2O, 1.23 mL, 3.0 equiv.) was added, and the resulting mixture was heated to 90 °C for 16 h. The mixture was allowed to cool, concentrated *in vacuo*, aq. NaOH (2.0 M, 25 mL) was added, and the aqueous component was extracted with CH_2Cl_2 (3 × 40 mL). The combined organics were dried with Na_2SO_4 and concentrated *in vacuo* to yield the **title compound** (520 mg, 70%) as a pale-yellow oil: ^{1}H NMR (400 MHz, Chloroform-*d*) δ 7.35 – 7.21 (m, 5H), 3.24 (q, J = 6.7 Hz, 1H, H-2), 2.20 (s, 6H, H-1), 1.37 (d, J = 6.7 Hz, 3H, H-3); $[\alpha]_D^{23}$ -44 (c = 1.0, CH_3OH). The spectral data are in agreement with reported literature values.[11]

Synthesis of 22

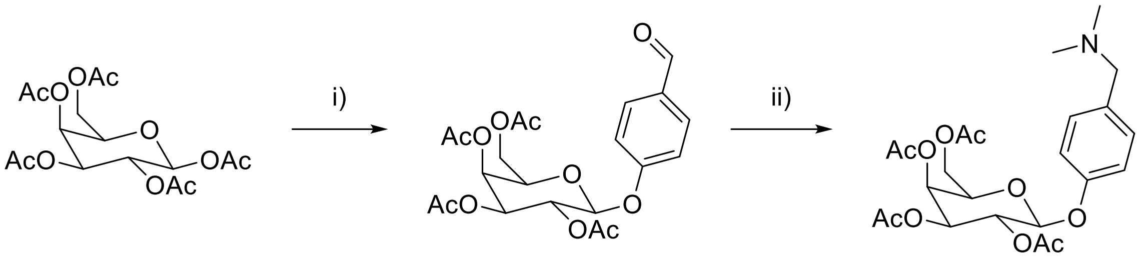

Scheme 6: Reagents and conditions: i) HBr, AcOH, rt, 1 h then 4-hydroxybenzaldehyde (2.0 equiv.), NaOH (aq.), acetone, rt, 18 h. ii) STAB (1.5 equiv.), HNMe$_2$ (1.5 equiv.), CH$_2$Cl$_2$, rt, 16 h

(Per-(*O*)-acetyl-β-D-galactopyranosyl)-4-oxybenzaldehyde

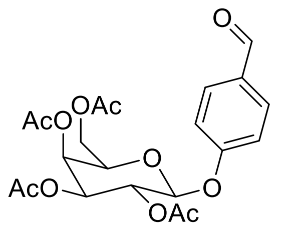

β-D-Galactose pentaacetate (1.95 g, 5.0 mmol, 1.0 equiv.) was suspended in HBr (33 wt% in AcOH, 5 mL) and AcOH (2.5 mL) and stirred for 1 h, after which ice (20 g) was added. The aqueous phase was extracted with CH_2Cl_2 (3 × 40 mL). The combined organics were washed with saturated aqueous $NaHCO_3$ (3 × 40 mL), dried ($MgSO_4$) and concentrated *in vacuo* to yield a colourless oil, which was taken forward without characterisation. The intermediate (1.99 g) in acetone (10 mL) was added dropwise to a solution of 4-hydroxybenzaldehyde (1.22 g, 10 mmol, 2.0 equiv.) in aqueous NaOH (1 M, 10 mL) and the reaction mixture was stirred for 18 h. Aqueous NaOH (50 mL) was added, and the reaction mixture was extracted with CH_2Cl_2 (3 × 50 mL), and the combined organics were washed with aqueous NaOH (1 M, 2 × 50 mL), H_2O (50 mL), dried ($MgSO_4$) and concentrated *in vacuo* to give a pale yellow oil. The crude residue was subjected to flash column chromatography (gradient from 20:80 EtOAc/petroleum ether to 50:50 EtOAc/petroleum ether) to give a clear oil. Trituration with EtOH gave the **title compound** (1.0 g, 44% over two steps) as a colourless solid: ^{1}H NMR

(400 MHz, Chloroform-*d*) δ 9.93 (s, 1H), 7.85 (d, *J* = 8.7 Hz, 2H), 7.11 (d, *J* = 8.7 Hz, 2H), 5.52 (dd, *J* = 10.4, 7.9 Hz, 1H), 5.48 (d, *J* = 3.3 Hz, 1H), 5.17 (d, *J* = 7.9 Hz, 1H), 5.14 (dd, *J* = 10.4, 3.3 Hz, 1H), 4.27 – 4.09 (m, 3H), 2.19 (s, 3H), 2.07 (s, 6H), 2.02 (s, 3H). The spectral data are in agreement with reported literature values.[12]

4-(Per-(*O*)-acetyl-β-D-galactopyranosyl)oxy-*N,N*-dimethylbenzylamine 22

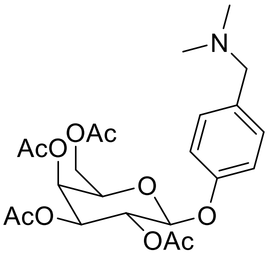

(Per-(O)-acetyl-β-D-galactopyranosyl)-4-oxybenzaldehyde (230 mg, 0.50 mmol, 1.0 equiv.) dimethylamine (2.0 M in THF, 0.38 mL, 0.75 mmol, 1.5 equiv.), and sodium triacetoxyborohydride (320 mg, 0.75 mmol, 1.5 equiv.) were combined in CH_2Cl_2 (20 mL) and stirred for 16 h. Saturated aqueous $NaHCO_3$ (50 mL) was added, the aqueous component was extracted with CH_2Cl_2 (3 × 30 mL), and combined organics were concentrated *in vacuo*. The resulting residue was suspended in Et_2O, filtered, and concentrated *in vacuo* to yield the **title compound** (217 mg, 90%) as a colourless foam: R_f 0.49 (EtOAc/petroleum ether 50:50); m.p. 52 – 53 °C; ^{1}H NMR (400 MHz, Chloroform-*d*) δ 7.22 (app d, *J* = 8.7 Hz, 2H), 6.95 (app d, *J* = 8.7 Hz, 2H), 5.51 – 5.44 (m, 2H), 5.10 (dd, *J* = 10.5, 3.4 Hz, 1H), 5.03 (d, *J* = 7.9 Hz, 1H), 4.26 – 4.13 (m, 2H), 4.07 – 4.03 (m, 1H), 3.37 (s, 2H), 2.22 (s, 6H), 2.18 (s, 3H), 2.06 (s, 3H), 2.06 (s, 3H), 2.01 (s, 3H); ^{13}C NMR (101 MHz, $CDCl_3$) δ 170.5, 170.4, 170.3, 169.5, 156.2, 134.0, 130.4, 116.9, 99.9, 71.2, 71.0, 68.8, 67.0, 63.7, 61.5, 45.4, 20.9, 20.8, 20.7; (ESI$^+$) *m/z* of $[M+H]^+$ detected: 482.2024, expected for $C_{23}H_{31}NO_{10}$: 482.2021; ν̄max (thin film)/cm^{-1} 2948 (w, C-H), 2861 (w, C-H), 2818 (w, C-H), 2775 (w, C-H), 1746 (s, C=O), 1502 (m), 1365 (m), 1208 (s), 1043 (s), 956 (w), 921 (w), 854 (w), 811 (w).

Synthesis of 23

6-(*N*,*N*-Dimethylaminomethyl)coumarin 23

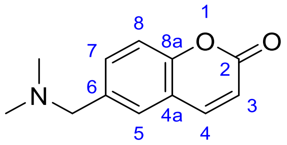

Ferrocene carboxaldehyde (210 mg, 1.0 mmol, 1.0 equiv.), dimethylamine (2.0 M in THF, 0.75 mL, 1.5 mmol, 1.5 equiv.), sodium triacetoxyborohydride (210 mg, 1.0 mmol, 1.0 equiv.) and glacial acetic acid (3 drops) were combined in CH_2Cl_2 (25 mL) and stirred at rt. After which, aqueous sat. $NaHCO_3$ (20 mL) was added, and the aqueous layer was extracted with CH_2Cl_2 (3 × 20 mL). The combined organic portions were dried (Na_2SO_4) and concentrated *in vacuo*. The crude material was subjected to flash column chromatography (gradient from 50:50 EtOAc/petroleum ether to 100% EtOAc with 1% NEt_3) to yield the **title compound** (156 mg, 77%) as a yellow oil; ^{1}H NMR (400 MHz, Chloroform-*d*) δ 7.69 (d, *J* = 9.6 Hz, 1H), 7.51 – 7.42 (m, 2H), 7.29 (d, *J* = 8.4 Hz, 1H), 6.42 (d, *J* = 9.5 Hz, 1H), 3.46 (s, 2H), 2.25 (s, 6H); ^{13}C NMR (101 MHz, $CDCl_3$) δ 161.1, 153.4, 143.6, 135.7, 132.7, 128.0, 118.8, 116.9, 63.6, 45.5; (ESI$^+$) *m/z* of $[M+H]^+$ detected: 204.1021, expected for $C_{12}H_{13}NO_2$: 204.1019; v̄max (thin film)/cm^{-1} 2921 (C-H, m), 2868 (C-H, m), 2827 (C-H, m), 2762 (C-H, m), 1723 (C=O, s), 1627 (m), 1572 (s), 1442 (s), 1385 (m), 1263 (m), 1169 (s), 1100 (s), 982 (m), 904 (m), 851 (m), 827 (s), 794 (s), 758 (m), 619 (m).

Synthesis of 24

4-Fluoroacetanilide 24

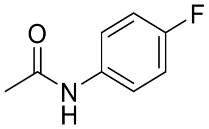

Acetyl chloride (160 µL, 2.2 mmol, 1.1 equiv.) was added dropwise to a solution of 4-fluoroaniline (190 µL, 2.0 mmol, 1.0 equiv.) and NEt_3 (560 µL, 4.0 mmol, 2.0 equiv.) in CH_2Cl_2 at 0 °C. Following complete addition, the solution was allowed to warm to rt, and stirred for 90 min. The reaction mixture was washed with saturated aqueous NH_4Cl (30 mL) and saturated aqueous $NaHCO_3$ (30 mL), dried (Na_2SO_4) and concentrated *in vacuo* to give the **title compound** (305 mg, >99%) as an off-white solid: ^{1}H NMR (400 MHz, Chloroform-*d*) δ 7.48 – 7.42 (m, 2H, Ar-H), 7.15 (s, 1H, N-H), 7.01 (t, *J* = 8.5 Hz, 2H, Ar-H), 2.17 (s, 3H, CH_3); ^{19}F NMR (376 MHz, $CDCl_3$) δ -118.01. The spectral data are in agreement with reported literature values.[13]

Synthesis of 27

Biotin-*N*-phenyl amide 27

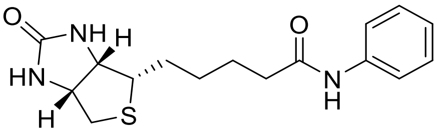

Biotin (122 mg, 0.50 mmol, 1.0 equiv.), EDC hydrochloride (115 mg, 0.60 mmol, 1.2 equiv.), HOBt hydrate (81 mg, 0.60 mmol, 1.2 equiv.) and NEt_3 (70 µL, 0.50 mmol, 1.0 equiv.) were combined in anhydrous DMF (3 mL) and stirred for 15 min. After which, aniline (46 µL, 0.50 mmol, 1.0 equiv.) was added, the mixture was heated to 40 °C and stirred for 3 h before being concentrated *in vacuo*. The residue was suspended in CH_2Cl_2 (10 mL) and filtered. The collected solid was further washed with CH_2Cl_2 (2 × 5 mL) to yield the **title compound** (117 mg, 73 %) as a colourless solid: m.p. decomposes at 150 °C; ^{1}H NMR (400 MHz, Methanol-d_4) δ 7.61 – 7.52 (m, 2H), 7.34 – 7.29 (m, 2H), 7.14 – 7.06 (m, 1H), 4.51 (ddd, *J* = 7.9, 5.0, 1.0 Hz, 1H), 4.33 (dd, *J* = 7.9, 4.5 Hz, 1H), 3.28 – 3.22 (m, 1H), 2.95 (dd, *J* = 12.8, 5.0 Hz, 1H), 2.72 (d, *J* = 12.7 Hz, 1H), 2.42 (t, *J* = 7.3 Hz, 2H), 1.81 – 1.49 (m, 6H); ^{13}C NMR (101 MHz, MeOD) δ 174.46, 166.12, 139.86, 129.75, 125.13, 121.28, 63.36, 61.64, 56.96, 41.04, 37.65, 29.81, 29.52, 26.76; ν̄max (thin film)/cm^{-1} 3293 (br, C-H), 2936 (w, C-H), 2861 (w, C-H), 1695 (s, C=O), 1659 (m), 1600 (m), 1530 (m), 1432 (m), 1310 (w), 1266 (w), 1078 (w), 878 (w), 756 (m), 689 (s).

LC-MS Analysis

Peptide conjugation LC-MS analyses were performed using an Agilent QTOF 6545 with Jetstream ESI spray source coupled to an Agilent 1260 Infinity II Quat pump HPLC with 1260 autosampler, column oven compartment and variable wavelength detector (VWD). The MS was operated in positive ionization mode with the gas temperature at 250°C, the drying gas at 12 L/min and the nebulizer gas at 45 psi (3.10 bar). The sheath gas temperature and flow were set to 350°C and 12 L/min, respectively. The MS was calibrated using reference calibrant introduced from the independent ESI reference sprayer. The VCap, Fragmentor and Skimmer was set to 3500, 100 and 45 respectively. Chromatographic separation of a 5 µL sample injection was performed on an InfinityLab Poroshell 120 EC-C18 (3.0 x 50 mm, 2.7 µm) column using water (H_2O, Merck, LC-MS grade) with 0.1 % formic acid (FA, Fluka) *v/v,* and methanol (MeOH, VWR, HiPerSolv) with 0.1 % FA *v/v,* as mobile phases A and B, respectively. The column was operated at flow rate of 0.3 mL/min at 40°C starting with 1 % mobile phase B for 3 min, thereafter the gradient was initiated and ran for 2 min to a final 100% B, held at 100% B for 3 min then returned to 1% B, and held for re-equilibration for 3.9 min, with a total 12 min run time. The VWD was set to collect data at 254 and 320 nm wavelengths at 2.5 Hz. Data processing was in Agilent Qual B 07.00 with a 'Find by formula' workflow, and matching tolerance of 5 ppm.

Reported conversions were determined using integrated peak areas of the Extracted Ion Chromatograms (EICs). Due to the different ionisation characteristics of labelled glutathione species, conversions were determined by calculating the remaining concentration of glutathione. Chromatographic peak areas were normalised to reflect the final dilution factor of the reaction mixture prior to LC-MS analysis. In all cases, only singly arylated glutathione (*m/z* specified under EICs) was detected, with no other peptide-based side products present in the mass spectra.

Peptide MS/MS analyses were performed using an Agilent QTOF 6545 with Jetstream ESI spray source coupled to an Agilent 1260 Infinity II Quat pump HPLC with 1260 autosampler, column oven compartment and variable wavelength detector (VWD). The MS was operated in positive ionization mode with the gas temperature at 325°C, the drying gas at 13 L/min and the nebulizer gas at 35 psi (2.41 bar) in the 100 – 2000 *m/z* ranges collecting 5 spectra/sec. The sheath gas temperature and flow were set to 300°C and 12 L/min, respectively. For MS/MS the mass ranges were 50 – 2000 *m/z* collecting 3 spectra/sec with an

isolation width set to medium (4 amu). Ions with charge states of 2, 3 or more were selected for fragmentation. For 2+ the slope was 3.1 with an offset of 1, 3+ the slope was 3.6 with an offset of -4.8, and more than 3 the slope was 3.6 with an offset of -4.8. Ten precursors were selected per cycle, actively excluded after 3 spectra for 0.2 min. The MS was calibrated using reference calibrant introduced from the independent ESI reference sprayer. Chromatographic separation was performed on a Water Acquity BEH C18 2.1 x 50 mm, 1.7 µm using H_20 (Merck, LC-MS grade) with 0.1% formic acid (FA, Fluka) *v/v* and acetonitrile (ACN, VWR, HiPerSolv) with 0.1% FA *v/v* as mobile phase A and B, respectively. The column was operated at flow rate of 0.3 mL/min at 50°C starting with 1 % mobile phase B for 0.5 min, thereafter the was gradient set to 5 min at 40% B, then 100% B at 7 min, held at 100% B for 2 min then returned to 1% B at 9.1 min in a total 12 min run time. The VWD was set to collect 280 and 320 nm wavelengths at 2.5 Hz (unless specified differently by user). Ten microliter injections of the samples were made. Data processing was automated in BioConfirm v 10 (Build 10.01.10136) or Qual B 07.00.

General procedure for glutathione labelling was adapted from the work of Buchwald and Pentelute.[14] Glutathione (4 µL, 150 µM in H_2O), H_2O (47 µL), organic solvent (DMF or MeCN, 2 µL), and phosphate buffer (6 µL, 100 mM, pH 7.5) were combined in a 1.0 mL plastic Eppendorf tube and the resulting solution was mixed by vortexing for 5 s. A stock solution of the palladium complex (1 µL, 1.2 mM in organic solvent) as added, the Eppendorf was vortexed for 5 s and left at room temperature for 5 min. The reaction was quenched by the addition of 3-mercaptopropionic acid (0.05 µL/mL solution in water, 3 equiv. to the palladium complex), H_2O (1000 µL) was added to the Eppendorf and the reaction mixture was analysed by LC-MS. Final concentrations of the reaction before quenching: peptide – 10 µM, Pd(II) complex – 20 µM, phosphate buffer – 10 mM; CH_3CN : H_2O = 5 : 95. Expected and observed *m/z*, EIC peak area, and retention time (rt) of both free and arylated GSH are reported underneath the EICs.

Glutathione calibration curve

H_2O (47 µL), CH_3CN (1 µL), a solution of *in situ* generated (RuPhos)Pd(II)(dmba)Cl (1 µL, 1.2 mM in CH_3CN), phosphate buffer (6 µL, 100 mM, pH 7.5) and 3-MPA (6.3 µL, 0.5 µL/mL in H_2O) were combined in a 1.5 mL plastic Eppendorf tube and the resulting solution was mixed by vortexing for 5 s. After 5 mins, glutathione (4 µL, varying concentrations) was added, H_2O (1000 µL) was added to the Eppendorf and the reaction mixture was analysed by LC-MS.

Concentration of glutathione added/µM	Reaction Concentration/µM	Concentration after dilution/nM	Counts
**188**	12.5	776	15,473,411
**150**	10.0	620	11,805,745
**93.8**	6.25	388	7,467,338
**46.9**	3.13	194	3,532,585
**23.4**	1.56	97.0	1,807,997
**11.7**	0.781	48.5	1,066,961
**7.50**	0.500	31.0	443,673
**3.75**	0.250	15.5	130,441
**1.88**	0.125	7.76	62,833

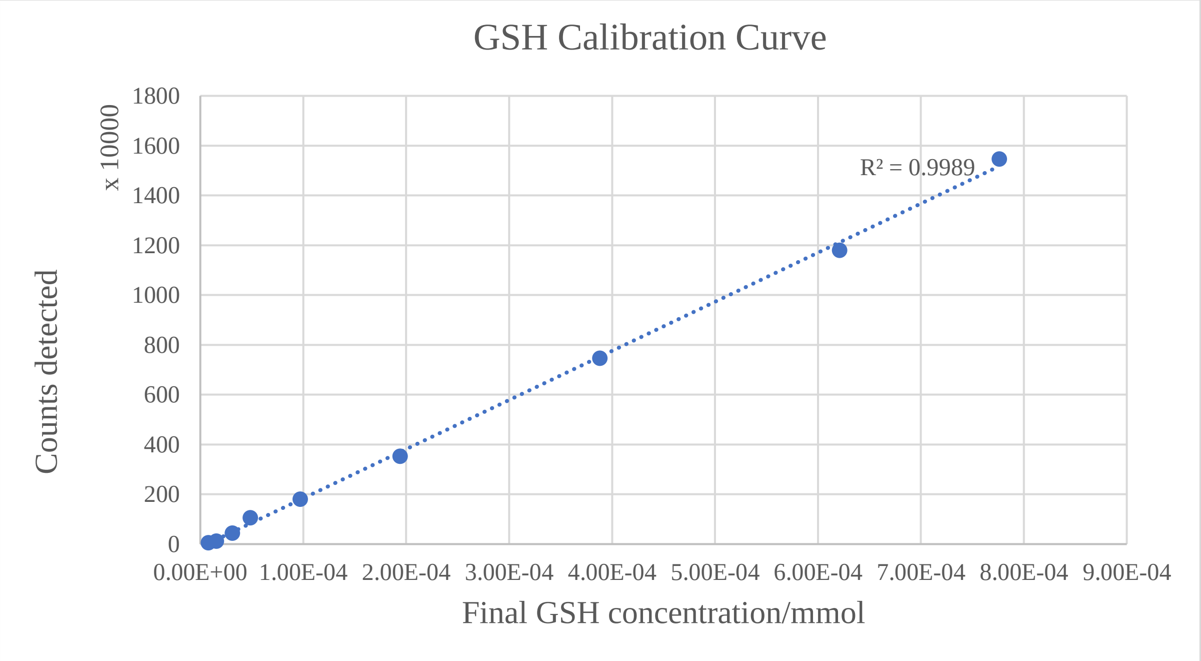

Graph 1: GSH calibration curve. Equation: $y = (1.98 \times 10^{10})x - 1.70 \times 10^5$. $R^2 = 0.999$

(dmba)Pd(II)Cl(Xantphos) Residue Selectivity

(dmba)Pd(II)Cl(Xantphos) was used in **general procedure** for arylation, *N*-acetyl cysteine, and H-Lys-OMe to assess selectivity.

	Unlabelled			Labelled		
**Amino acid**	LC Rt (min)	m/z detected	m/z expected	LC Rt (min)	m/z detected	m/z expected
***N*-Acetyl cysteine**	2.63	164.0377	164.0376 $(M+H)^+$	7.69	297.1272	297.1267 $(M+H)^+$
**H-Lys-OMe**	0.88	161.1287	161.1285 $(M+H)^+$	N/A	N/A	N/A

Table 1: Detected species in reaction of (dmba)Pd(II)Cl(Xantphos) and N-acetyl cysteine, and H-Lys-OMe.

(dmba)Pd(II)Cl(Xantphos) was used in **general procedure** for arylation and subjected to LC-MS/MS analysis. Targeted MS/MS was scheduled for the 308.09 *m/z* (GSH) and 441.17 *m/z* (arylated GSH) precursors using collision energies of 10, 20 and 40 eV at their respective elution times.

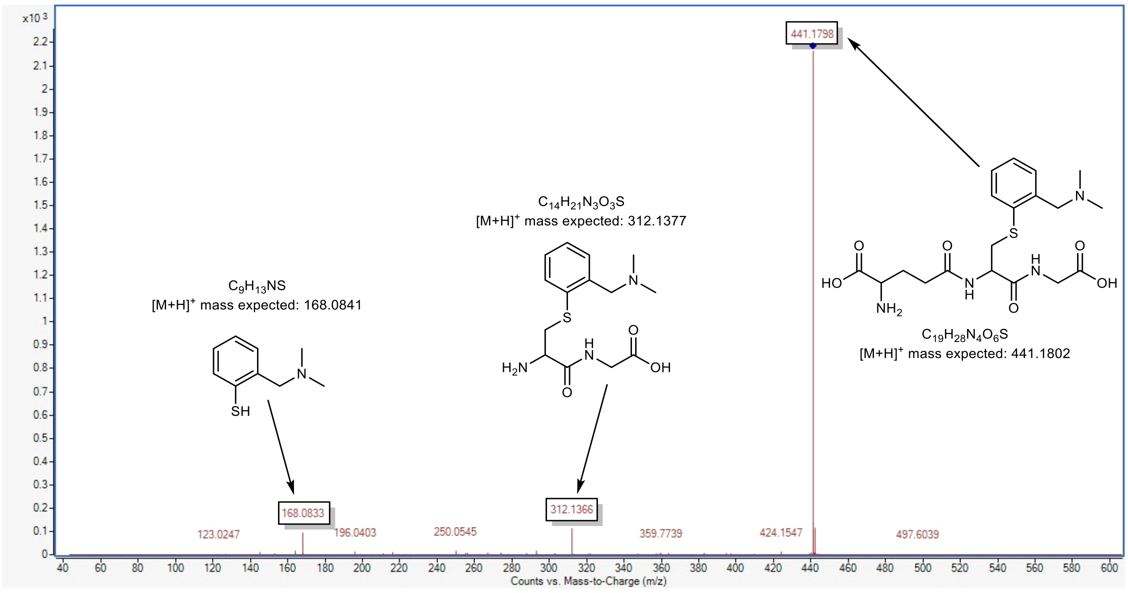

*Figure 1: MS/MS spectrum of arylated glutathione after using **1a** in **general procedure** for glutathione labelling.*

Glutathione arylation using isolated Pd(II) complexes

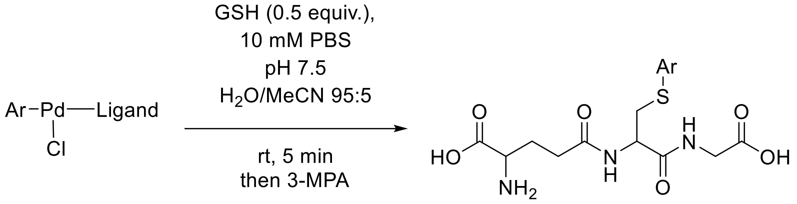

Ar =	X =	Ligand	Organic solvent	Dilution corrected GSH counts	GSH Conversion	LC-MS EIC trace
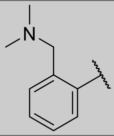	Cl	RuPhos	MeCN	540,454	94%	EIC 1
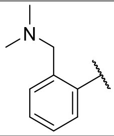	Cl	Xantphos	MeCN	396,801	95%	EIC 2
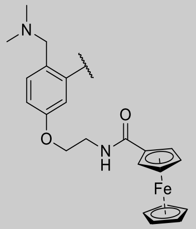	Cl	RuPhos	MeCN	1,033,322	90%	EIC 3
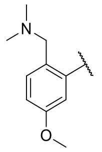	Cl	RuPhos	MeCN	700,161	93%	EIC 4
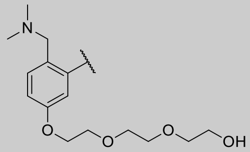	Cl	RuPhos	MeCN	3,887,346	67%	EIC 5
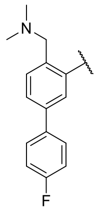	Cl	RuPhos	MeCN	2,064,537	82%	EIC 6
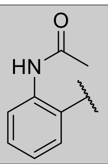	OTs	RuPhos	DMF	66,539	98%	EIC 7
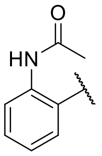	OTs	Xantphos	DMF	170,837	97%	EIC 8

Table 2: Labelling efficiency of cyclopalladated Pd(II) complexes.

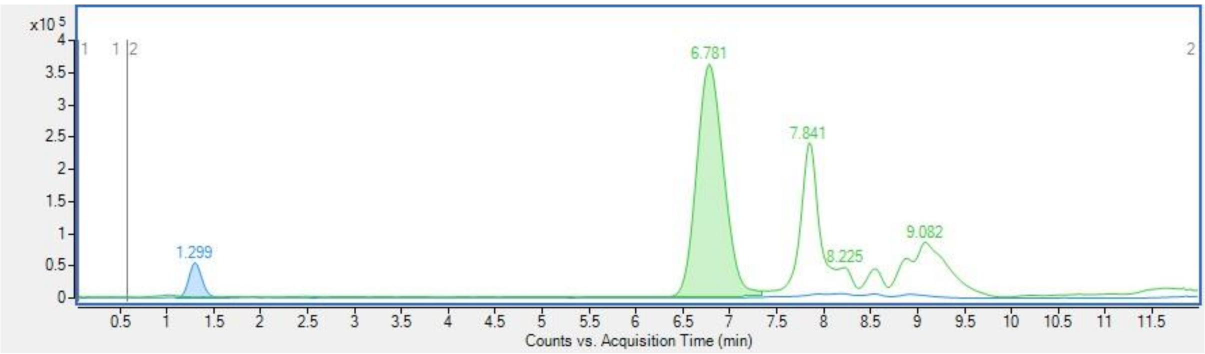

EIC 1: GSH (ESI$^+$) m/z of $[M+H]^+$ detected: 308.0910, expected for $C_{10}H_{17}N_3O_6S$: 308.0911. GSH EIC peak area: 540454 (Rt = 1.30 min). Arylated GSH (ESI$^+$) m/z of $[M+H]^+$ detected: 441.1800, expected for $C_{19}H_{28}N_4O_6S$: 441.1802. Arylated GSH EIC peak area: 7223664 (Rt = 6.78 min).

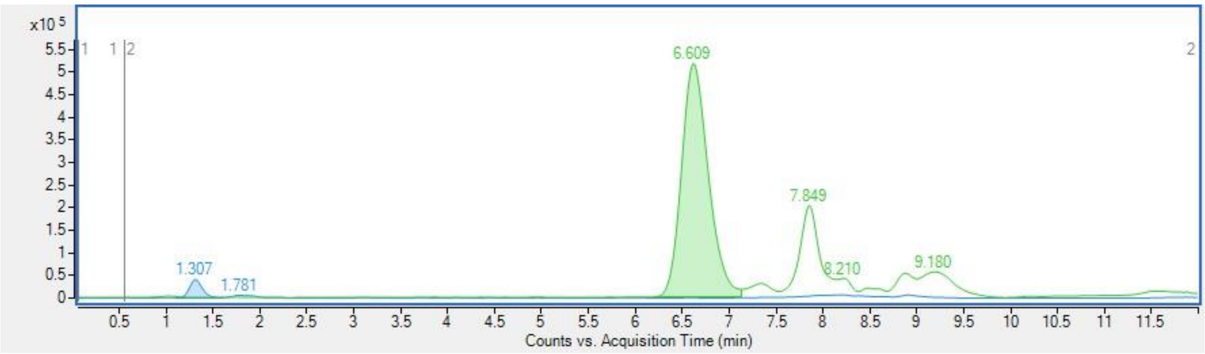

EIC 2: GSH (ESI$^+$) m/z of $[M+H]^+$ detected: 308.0910, expected for $C_{10}H_{17}N_3O_6S$: 308.0911. GSH EIC peak area: 396801 (Rt = 1.31 min). Arylated GSH (ESI$^+$) m/z of $[M+H]^+$ detected: 441.1800, expected for $C_{19}H_{28}N_4O_6S$: 441.1802. Arylated GSH EIC peak area: 10289154 (Rt = 6.61 min).

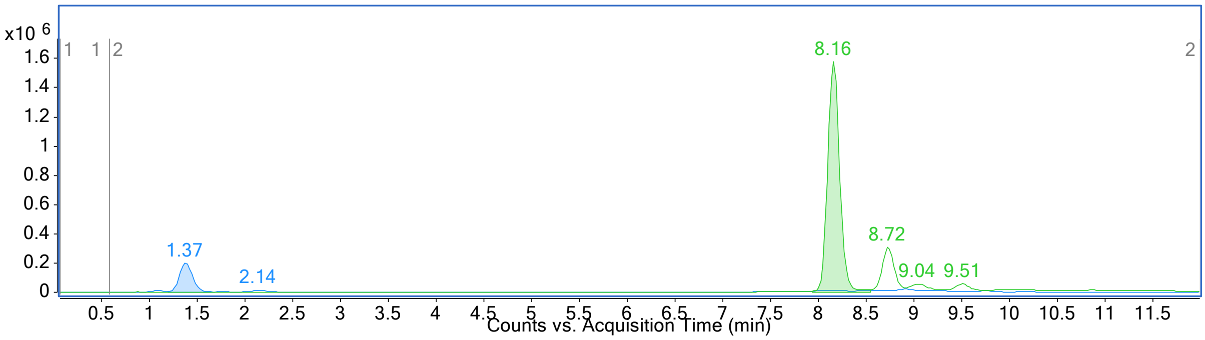

EIC 3: GSH (ESI$^+$) m/z of $[M+H]^+$ detected: 308.0911, expected for $C_{10}H_{17}N_3O_6S$: 308.0911. GSH EIC peak area: 1909012 (Rt = 1.37 min). Arylated GSH (ESI$^+$) m/z of $[M]^+$ detected:710.2131 expected for $C_{32}H_{41}FeN_5O_8S$: 710.2145. Arylated GSH EIC peak area: 13869245 (Rt = 8.16 min).

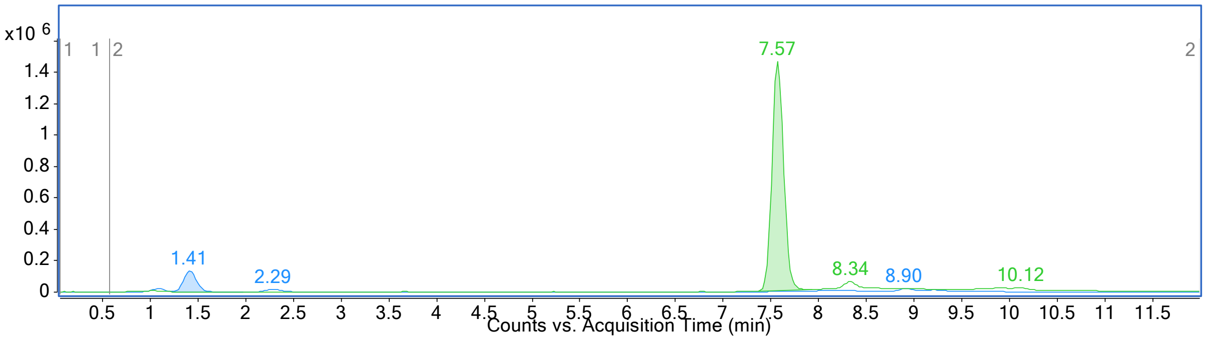

EIC 4: GSH (ESI+) m/z of [M+H]+ detected: 308.0910, expected for $C_{10}H_{17}N_3O_6S$: 308.0911. GSH EIC peak area: 1311860 (Rt = 1.41 min). Arylated GSH (ESI+) m/z of [M+H]+ detected: 471.1886, expected for $C_{20}H_{30}N_4O_7S$: 471.1908. Arylated GSH EIC peak area: 12487789 (Rt = 7.57 min).

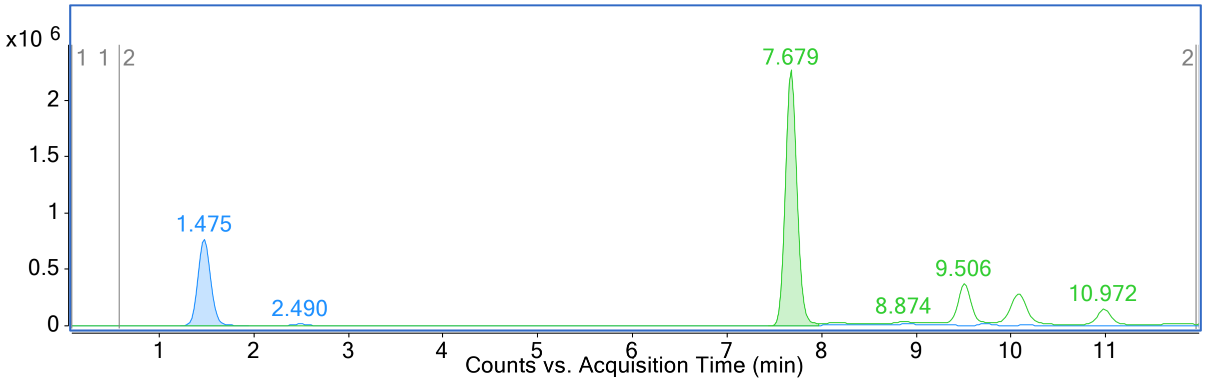

EIC 5: GSH (ESI+) m/z of [M+H]+ detected: 308.0912, expected for $C_{10}H_{17}N_3O_6S$: 308.0911. GSH EIC peak area: 19675679 (Rt = 1.48 min). Arylated GSH (ESI+) m/z of [M+H]+ detected: 589.2534, expected for $C_{25}H_{40}N_4O_{10}S$: 589.2538. Arylated GSH EIC peak area: 26995863 (Rt = 7.68 min)

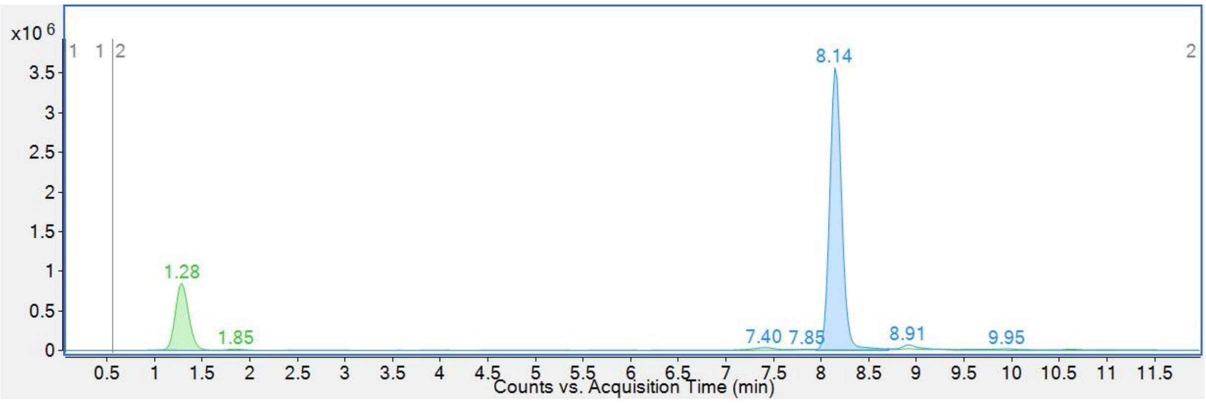

EIC 6: GSH (ESI+) m/z of [M+H]+ detected: 308.0909, expected for $C_{10}H_{17}N_3O_6S$: 308.0911. GSH EIC peak area: 8266674 (Rt = 1.28 min). Arylated GSH (ESI+) m/z of [M+H]+ detected: 535.2012, expected for $C_{25}H_{31}FN_4O_6S$: 535.2021. Arylated GSH EIC peak area: 31245140 (Rt = 8.14 min).

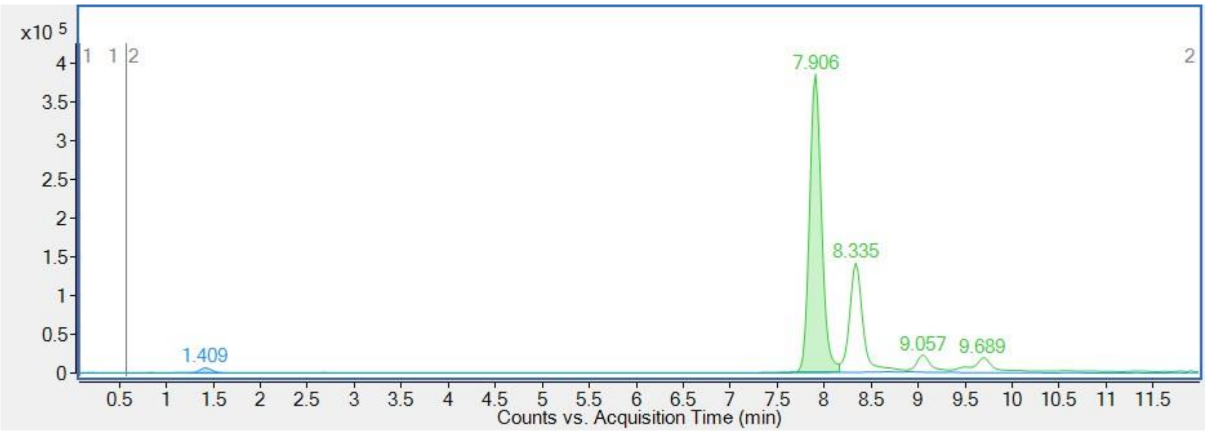

EIC 7: GSH (ESI⁺) m/z of [M+H]⁺ detected: 308.0916, expected for $C_{10}H_{17}N_3O_6S$: 308.0911. GSH counts: 66,539 (rt = 1.41 min). Arylated GSH (ESI⁺) m/z of [M+H]⁺ detected: 441.1447, expected for $C_{18}H_{24}N_4O_7S$: 441.1438. Arylated GSH counts: 3,414,065 (rt = 7.91 min).

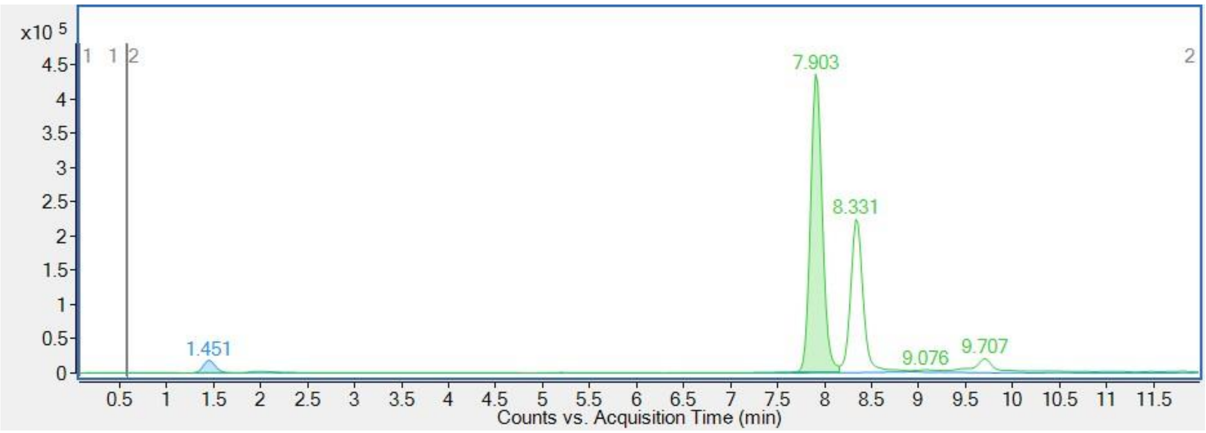

EIC 8: GSH (ESI⁺) m/z of [M+H]⁺ detected: 308.0916, expected for $C_{10}H_{17}N_3O_6S$: 308.0911. GSH counts: 170,837 (rt = 1.45 min). Arylated GSH (ESI⁺) m/z of [M+H]⁺ detected: 441.1448, expected for $C_{18}H_{24}N_4O_7S$: 441.1438. Arylated GSH counts: 3,814,698 (rt = 7.90 min).

Results of Pd(II)-Ar complex forming conditions A and B

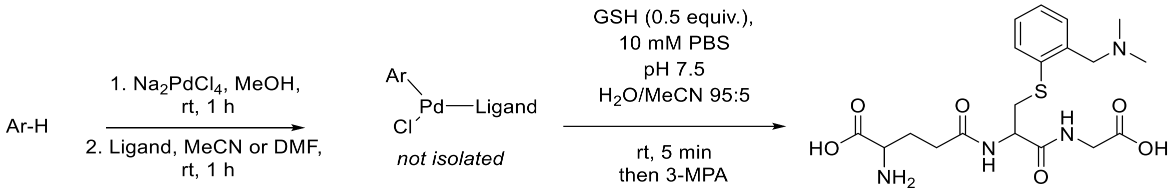

A: Ar-H (15 µL, 80 mM solution in MeOH, 2.0 equiv.) and Na_2PdCl_4 (15 µL, 40 mM solution in MeOH, 1.0 equiv.) and MeOH (20 µL) were combined and stirred for 1 h at rt. After which, RuPhos (30 µL, 20 mM in MeCN, 1.0 equiv.) was added, the reaction mixture was diluted with MeCN (420 µL) and stirred for another 1 h at rt, resulting in a stock solution of Pd(II) complex (500 µL, 1.2 mM). This stock solution was then subjected to the conditions of **general procedure** for glutathione labelling.

B: Ar-H (15 µL, 80 mM solution in MeOH, 2.0 equiv.) and Na_2PdCl_4 (15 µL, 40 mM solution in MeOH, 1.0 equiv.) and MeOH (20 µL) were combined and stirred for 1 h at rt. After which, Xantphos (30 µL, 20 mM in MeCN, 1.0 equiv.) was added, the reaction mixture was diluted with DMF (420 µL) and stirred for another 1 h at rt, resulting in a stock solution of Pd(II) complex (500 µL, 1.2 mM). This stock solution was then subjected to the conditions of **general procedure** for glutathione labelling.

Ar =	Ligand/organic solvent	Dilution corrected GSH counts	GSH Conversion	LC-MS EIC trace
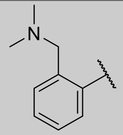	RuPhos/MeCN	2,596,898	78%	EIC 9
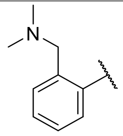	Xantphos/DMF	2,252,129	80%	EIC 10
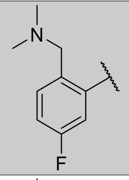	RuPhos/MeCN	5,891,705	51%	EIC 11
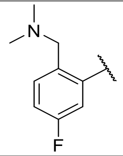	Xantphos/DMF	9,554,814	21%	EIC 12

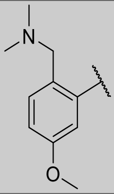	RuPhos/MeCN	4,508,158	62%	EIC 13
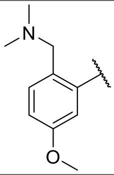	Xantphos/DMF	3,011,416	74%	EIC 14
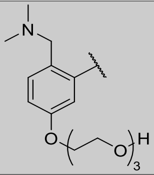	RuPhos/MeCN	2,019,699	82%[a]	EIC 15
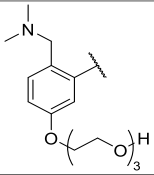	Xantphos/DMF	533,460	94%[a]	EIC 16
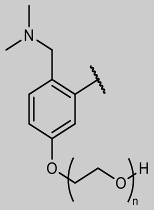	RuPhos/MeCN	1,494,721	87%[a]	EIC 17
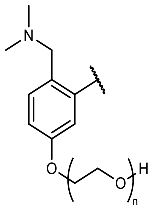	Xantphos/DMF	2,410,473	79%[a]	EIC 18
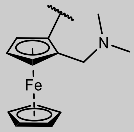	RuPhos/MeCN	9,511,125	22%	EIC 19
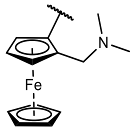	Xantphos/DMF	8,569,304	29%	EIC 20
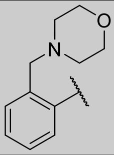	RuPhos/MeCN	3,262,707	72%	EIC 21
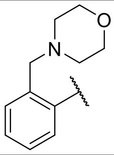	Xantphos/DMF	3,992,355	66%	*EIC 22*
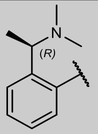	RuPhos/MeCN	3,273,043	72%	*EIC 23*

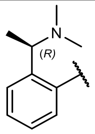	Xantphos/DMF	4,402,912	63%	*EIC 24*
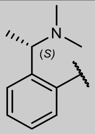	RuPhos/MeCN	5,504,666	54%	*EIC 25*
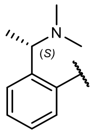	Xantphos/DMF	2,450,568	79%	*EIC 26*
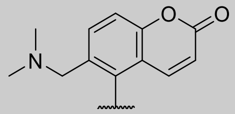	RuPhos/MeCN	3,049,893	74%	*EIC 27*
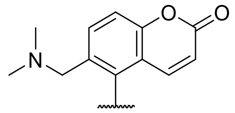	Xantphos/DMF	6,650,626	45%	*EIC 28*

*Table 3: Labelling efficiency of cyclopalladated Pd(II) complexes using Pd(II)-Ar complex forming conditions **A** and **B**.[a] Initial cyclopalladation was conducted for 18 h, instead of 1 h.*

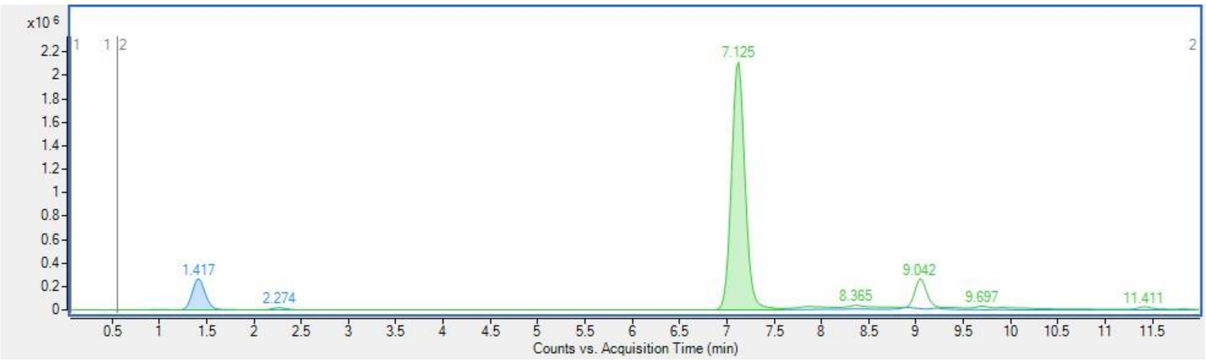

EIC 9: GSH (ESI$^+$) m/z of $[M+H]^+$ detected: 308.0912, expected for $C_{10}H_{17}N_3O_6S$: 308.0911. GSH counts: 2,596,898 (rt = 1.42 min). Arylated GSH (ESI$^+$) m/z of $[M+H]^+$ detected: 441.1803, expected for $C_{19}H_{28}N_4O_6S$: 441.1802. Arylated GSH counts: 21,083,331 (rt = 7.13 min).

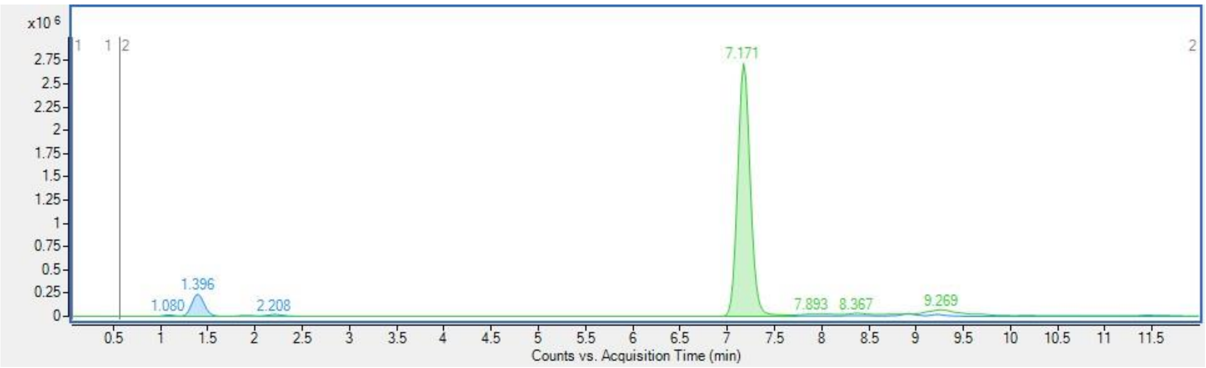

EIC 10: GSH (ESI$^+$) m/z of $[M+H]^+$ detected: 308.0911, expected for $C_{10}H_{17}N_3O_6S$: 308.0911. GSH counts: 2,596,898 (rt = 1.42 min). Arylated GSH (ESI$^+$) m/z of $[M+H]^+$ detected: 441.1804, expected for $C_{19}H_{28}N_4O_6S$: 441.1802. Arylated GSH counts: 21,083,331 (rt = 7.17 min).

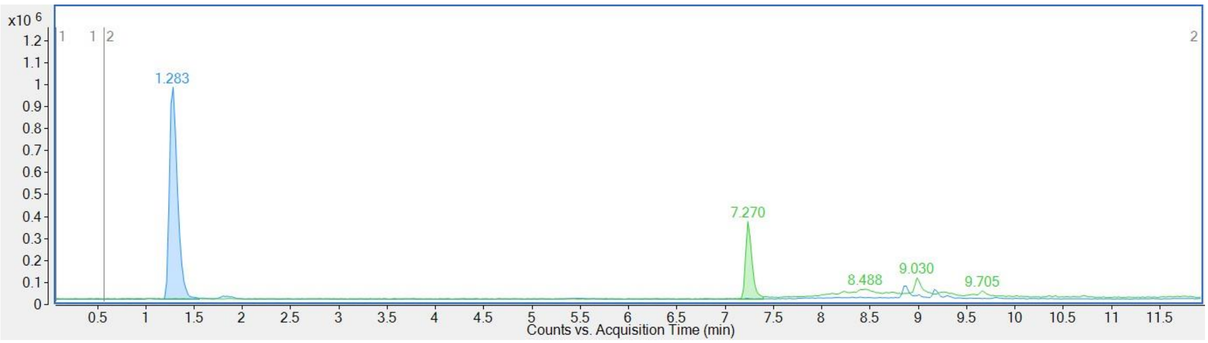

EIC 11: GSH (ESI$^+$) m/z of $[M+H]^+$ detected: 308.0913, expected for $C_{10}H_{17}N_3O_6S$: 308.0911. GSH counts: 5,891,705 (rt = 1.28 min). Arylated GSH (ESI$^+$) m/z of $[M+H]^+$ detected: 459.1706, expected for $C_{19}H_{27}FN_4O_6S$: 459.1708. Arylated GSH counts: 1,743,145 (rt = 7.27 min).

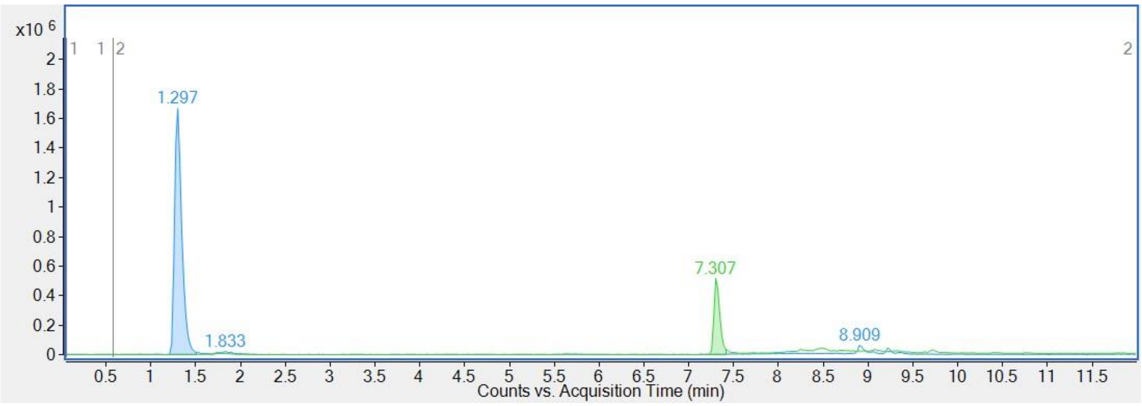

EIC 12: (ESI$^+$) m/z of $[M+H]^+$ detected: 308.0913, expected for $C_{10}H_{17}N_3O_6S$: 308.0911. GSH counts: 9,554,814 (rt = 1.30 min). Arylated GSH (ESI$^+$) m/z of $[M+H]^+$ detected: 459.1705, expected for $C_{19}H_{27}FN_4O_6S$: 459.1708. Arylated GSH counts: 2,499,100 (rt = 7.31 min).

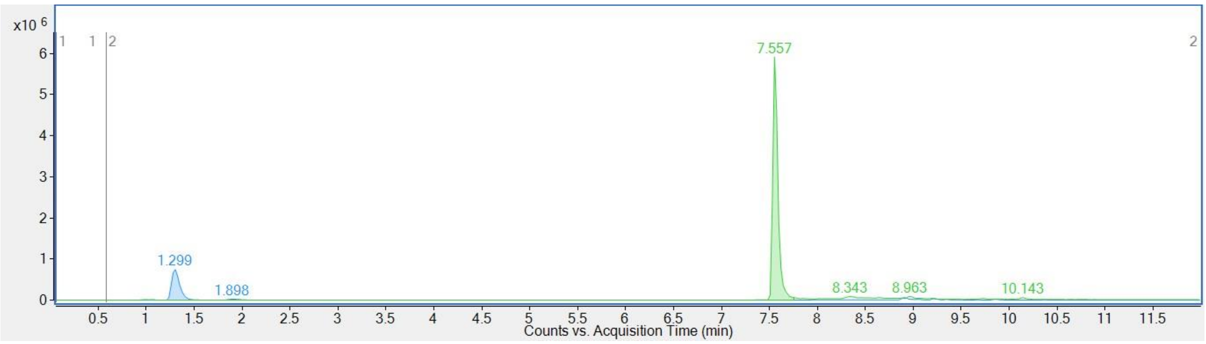

EIC 13: (ESI$^+$) m/z of $[M+H]^+$ detected: 308.0913, expected for $C_{10}H_{17}N_3O_6S$: 308.0911. GSH counts: 4,508,158 (rt = 1.30 min). Arylated GSH (ESI$^+$) m/z of $[M+H]^+$ detected: 471.1906, expected for $C_{20}H_{30}N_4O_7S$: 471.1908. Arylated GSH counts: 22,898,497 (rt = 7.56 min).

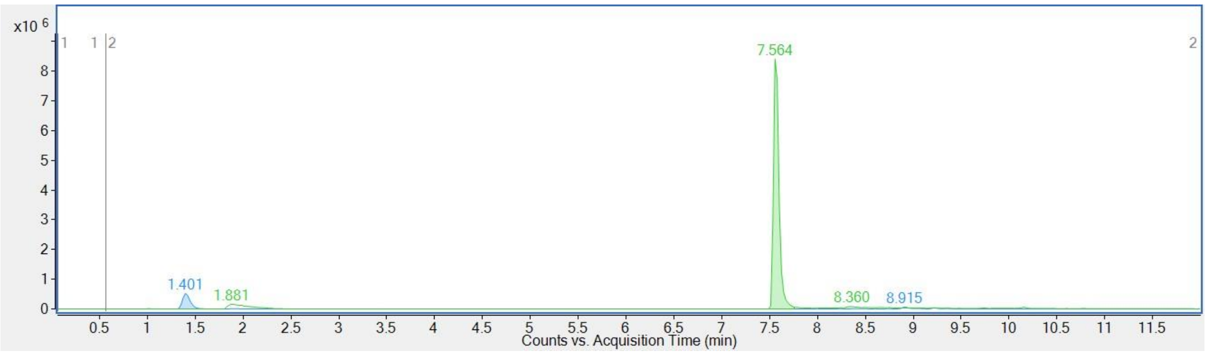

EIC 14: (ESI⁺) m/z of [M+H]⁺ detected: 308.0911, expected for $C_{10}H_{17}N_3O_6S$: 308.0911. GSH counts: 4,508,158 (rt = 1.30 min). Arylated GSH (ESI⁺) m/z of [M+H]⁺ detected: 471.1908, expected for $C_{20}H_{30}N_4O_7S$: 471.1908. Arylated GSH counts: 34,639,951 (rt = 7.56 min).

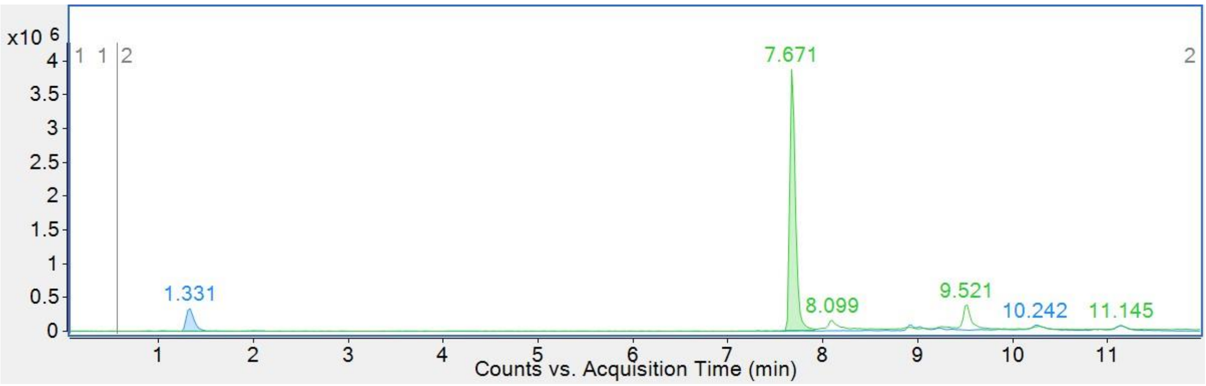

EIC 15: (ESI⁺) m/z of [M+H]⁺ detected: 308.0912, expected for $C_{10}H_{17}N_3O_6S$: 308.0911. GSH counts: 2,019,699 (rt = 1.33 min). Arylated GSH (ESI⁺) m/z of [M+H]⁺ detected: 589.2532, expected for $C_{25}H_{40}N_4O_{10}S$: 589.2538. Arylated GSH counts: 16,095,002 (rt = 7.67 min).

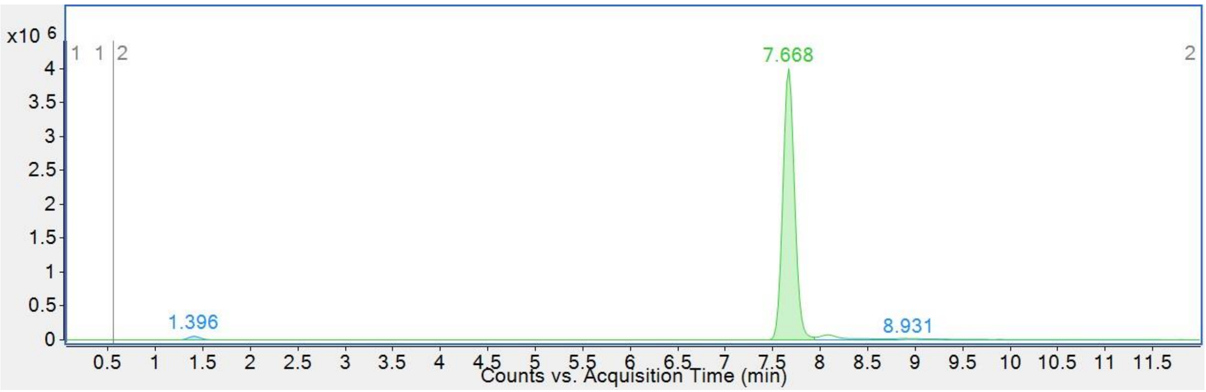

EIC 16: (ESI⁺) m/z of [M+H]⁺ detected: 308.0910, expected for $C_{10}H_{17}N_3O_6S$: 308.0911. GSH counts: 2,019,699 (rt = 1.40 min). Arylated GSH (ESI⁺) m/z of [M+H]⁺ detected: 589.2533, expected for $C_{25}H_{40}N_4O_{10}S$: 589.2538. Arylated GSH counts: 16,095,002 (rt = 7.67 min).

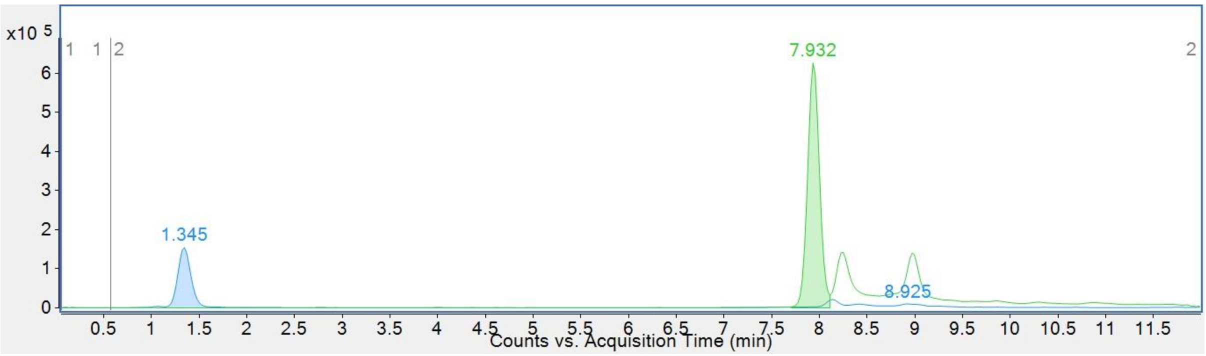

EIC 17: (ESI^+) m/z of $[M+H]^+$ detected: 308.0911, expected for $C_{10}H_{17}N_3O_6S$: 308.0911. GSH counts: 1,494,721 (rt = 1.35 min). Arylated GSH (ESI^+) m/z of $[M+Na]^+$ detected: 831.3653, expected for $C_{35}H_{60}N_4O_{16}S$ (n = 8): 831.3668 (rt = 7.93 min). Note: n = 8 chosen as representative arylation product, other lengths of polymer chain also detected.

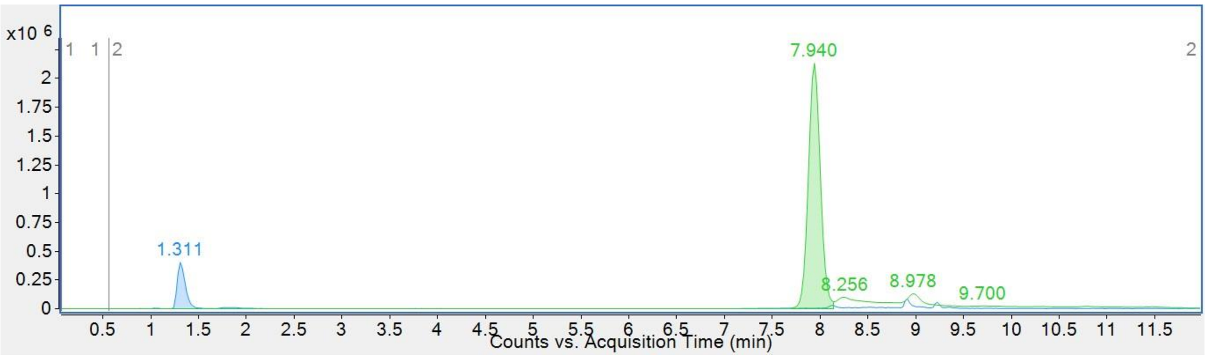

EIC 18: (ESI^+) m/z of $[M+H]^+$ detected: 308.0911, expected for $C_{10}H_{17}N_3O_6S$: 308.0911. GSH counts: 1,494,721 (rt = 1.31 min). Arylated GSH (ESI^+) m/z of $[M+2H]^{2+}$ detected: 405.1964, expected for $C_{35}H_{60}N_4O_{16}S$ (n = 8): 405.1961 (rt = 7.94 min). Note: n = 8 chosen as representative arylation product, other lengths of polymer chain also detected.

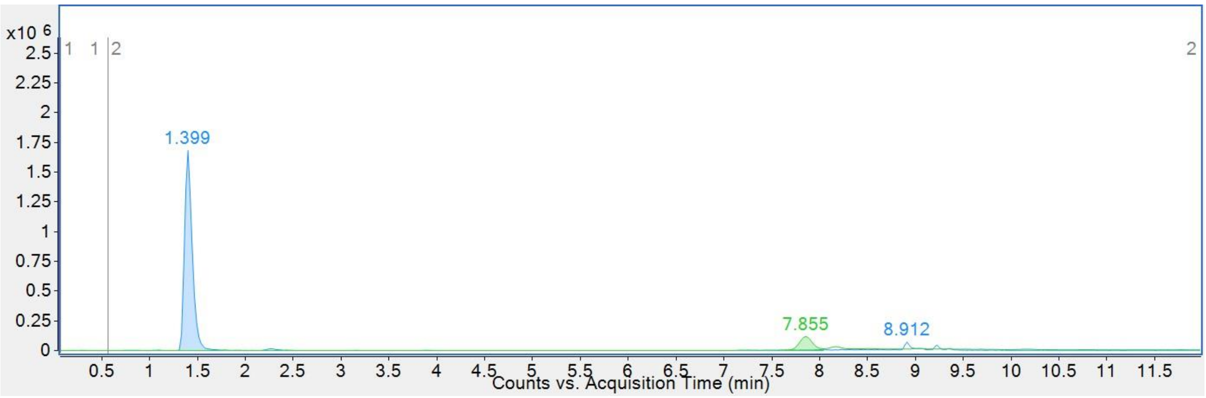

EIC 19: GSH (ESI^+) m/z of $[M+H]^+$ detected: 308.0913, expected for $C_{10}H_{17}N_3O_6S$: 308.0911. GSH counts: 9,511,125 (rt = 1.40 min). Arylated GSH (ESI^+) m/z of $[M+H]^+$ detected: 549.1459, expected for $C_{23}H_{32}FeN_4O_6S$: 549.1465. Arylated GSH counts: 117,128 (rt = 7.86 min).

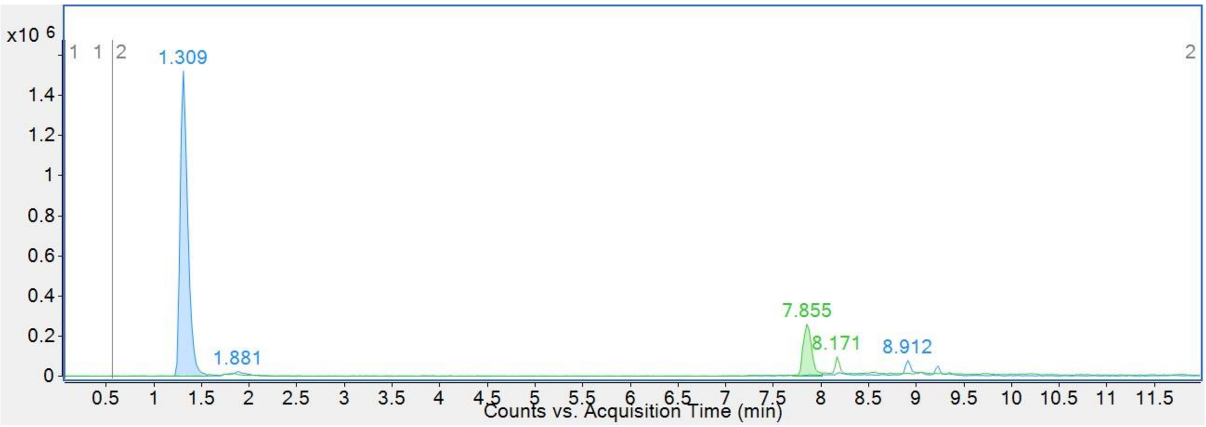

EIC 20: GSH (ESI$^+$) m/z of $[M+H]^+$ detected: 308.0913, expected for $C_{10}H_{17}N_3O_6S$: 308.0911. GSH counts: 8,569,304 (rt = 1.31 min). Arylated GSH (ESI$^+$) m/z of $[M+H]^+$ detected: 549.1456, expected for $C_{23}H_{32}FeN_4O_6S$: 549.1465. Arylated GSH counts: 1,691,948 (rt = 7.86 min).

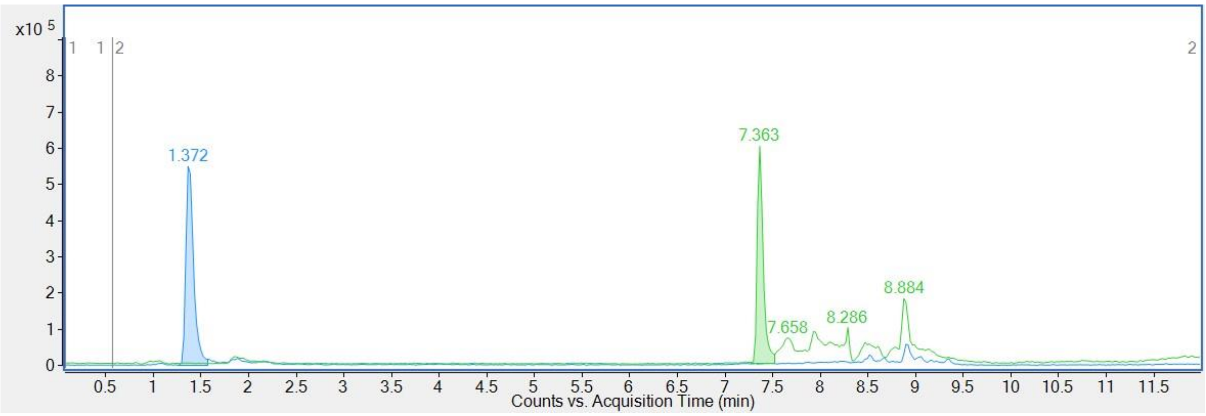

EIC 21: GSH (ESI$^+$) m/z of $[M+H]^+$ detected: 308.0909, expected for $C_{10}H_{17}N_3O_6S$: 308.0911. GSH counts: 3,262,707 (rt = 1.37 min). Arylated GSH (ESI$^+$) m/z of $[M+H]^+$ detected: 483.1901, expected for $C_{21}H_{30}N_4O_7S$: 483.1908. Arylated GSH counts: 2,746,639 (rt = 7.37 min).

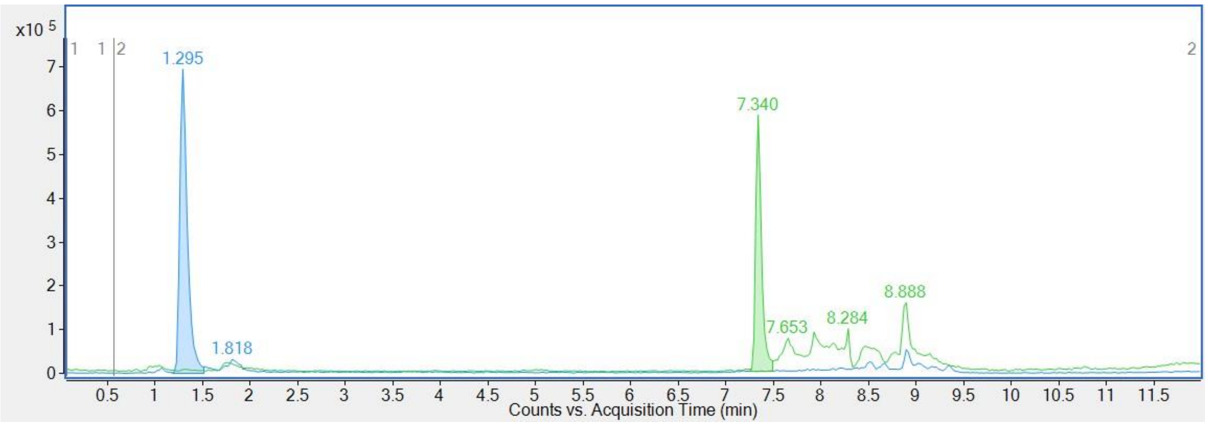

EIC 22: GSH (ESI$^+$) m/z of $[M+H]^+$ detected: 308.0909, expected for $C_{10}H_{17}N_3O_6S$: 308.0911. GSH counts: 3,992,355 (rt = 1.30 min). Arylated GSH (ESI$^+$) m/z of $[M+H]^+$ detected: 483.1900, expected for $C_{21}H_{30}N_4O_7S$: 483.1908. Arylated GSH counts: 2,626,363 (rt = 7.34 min).

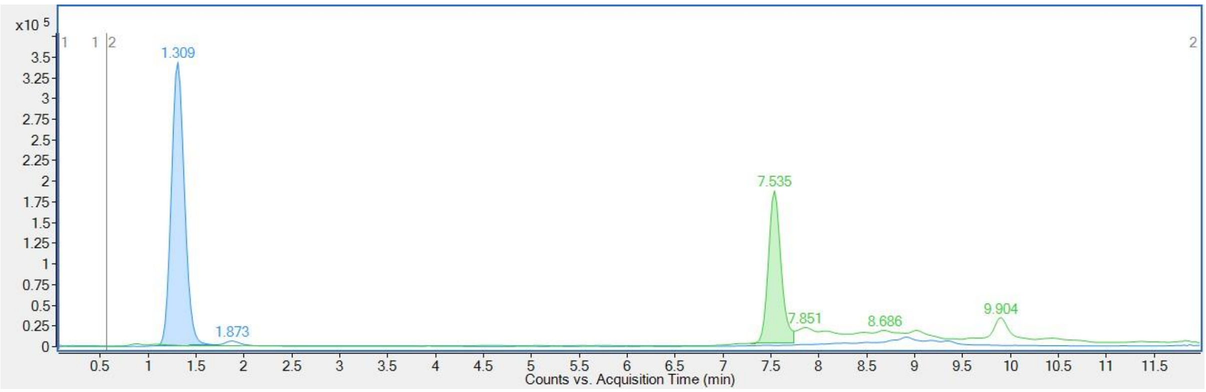

EIC 23: GSH (ESI$^+$) m/z of [M+H]$^+$ detected: 308.0910, expected for $C_{10}H_{17}N_3O_6S$: 308.0911. GSH counts: 3,273,043 (rt = 1.31 min). Arylated GSH (ESI$^+$) m/z of [M+H]$^+$ detected: 455.1955, expected for $C_{20}H_{30}N_4O_6S$: 455.1959. Arylated GSH counts: 1,697,237 (rt = 7.54 min).

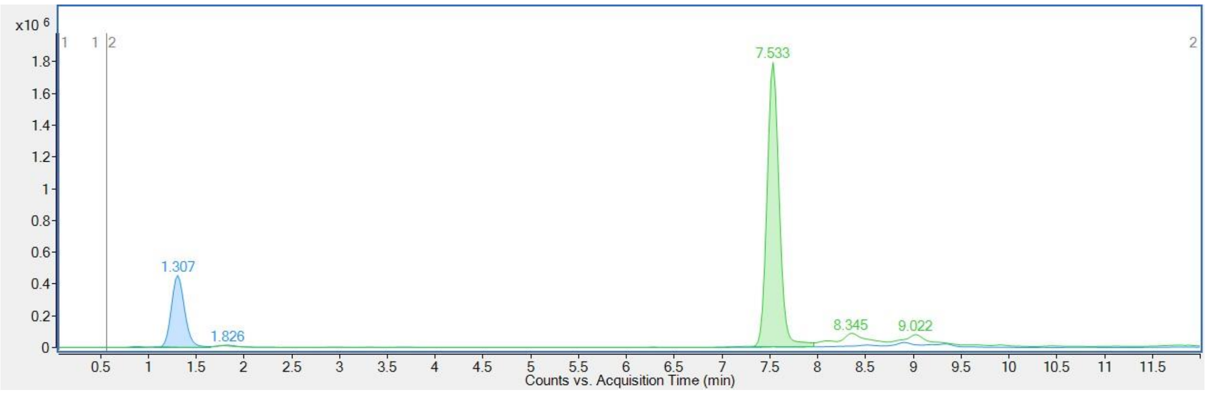

EIC 24: GSH (ESI$^+$) m/z of [M+H]$^+$ detected: 308.0909, expected for $C_{10}H_{17}N_3O_6S$: 308.0911. GSH counts: 4,402,912 (rt = 1.31 min). Arylated GSH (ESI$^+$) m/z of [M+H]$^+$ detected: 455.1956, expected for $C_{20}H_{30}N_4O_6S$: 455.1959. Arylated GSH counts: 15,980,466 (rt = 7.53 min).

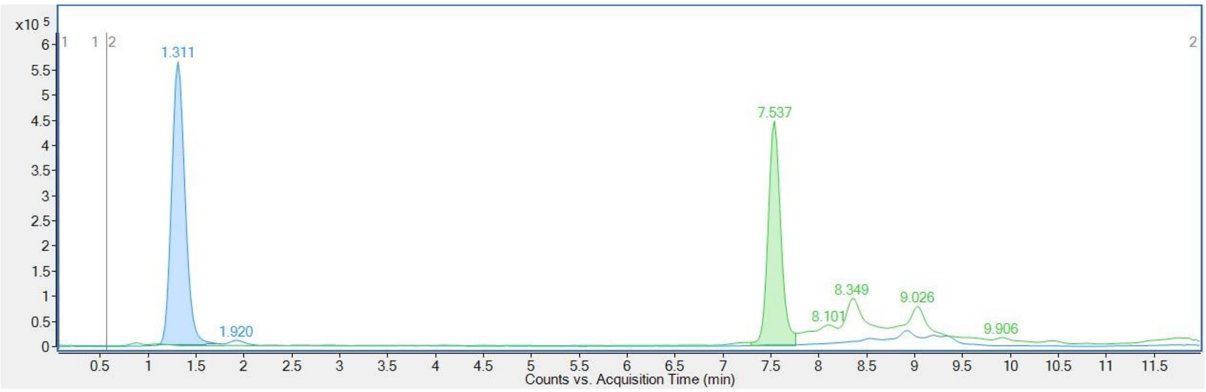

EIC 25: GSH (ESI$^+$) m/z of [M+H]$^+$ detected: 308.0909, expected for $C_{10}H_{17}N_3O_6S$: 308.0911. GSH counts: 5,504,666 (rt = 1.31 min). Arylated GSH (ESI$^+$) m/z of [M+H]$^+$ detected: 455.1952, expected for $C_{20}H_{30}N_4O_6S$: 455.1959. Arylated GSH counts: 4,127,423 (rt = 7.54 min).

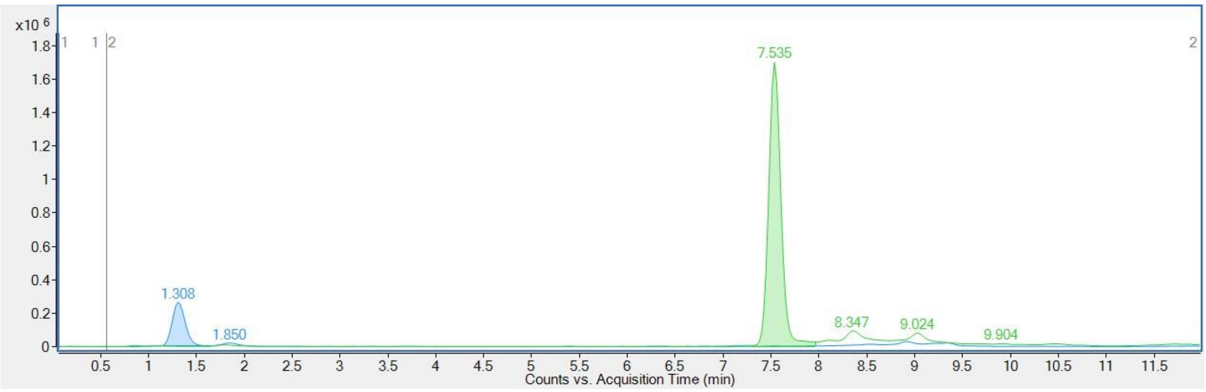

EIC 26: GSH (ESI$^+$) m/z of $[M+H]^+$ detected: 308.0908, expected for $C_{10}H_{17}N_3O_6S$: 308.0911. GSH counts: 2,450,568 (rt = 1.31 min). Arylated GSH (ESI$^+$) m/z of $[M+H]^+$ detected: 455.1954, expected for $C_{20}H_{30}N_4O_6S$: 455.1959. Arylated GSH counts: 15,208,163 (rt = 7.54 min).

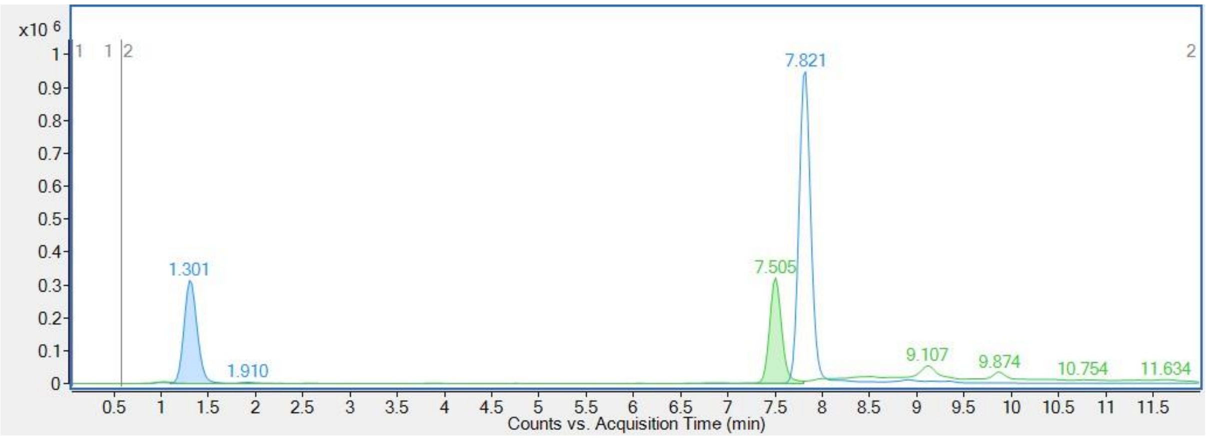

EIC 27: GSH (ESI$^+$) m/z of $[M+H]^+$ detected: 308.0910, expected for $C_{10}H_{17}N_3O_6S$: 308.0911. GSH counts: 3,049,893 (rt = 1.30 min). Arylated GSH (ESI$^+$) m/z of $[M+H]^+$ detected: 501.1695, expected for $C_{22}H_{28}N_4O_8S$: 501.1701. Arylated GSH counts: 2,889,766 (rt = 7.51 min). Note: GSH peak at 7.82 min likely due to degradation of conjugate.

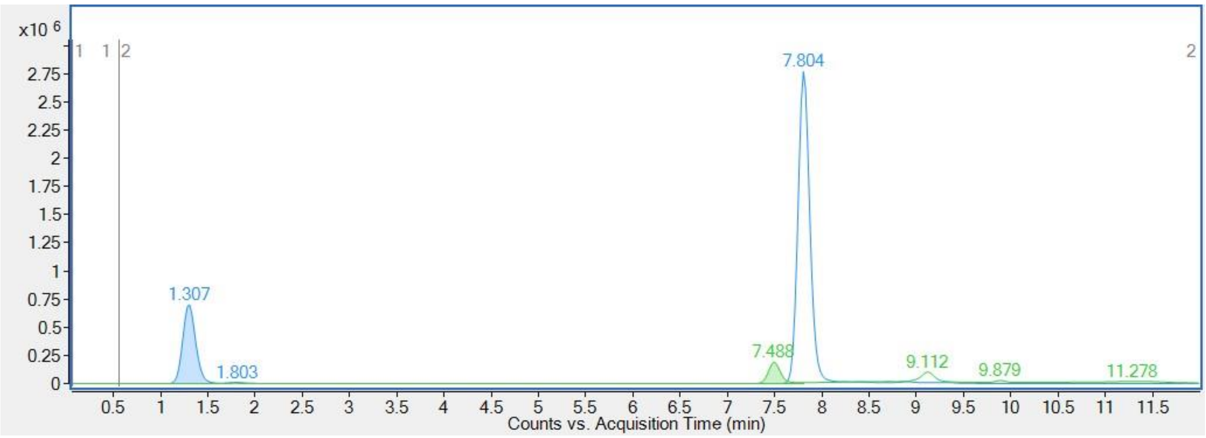

EIC 28: GSH (ESI$^+$) m/z of $[M+H]^+$ detected: 308.0910, expected for $C_{10}H_{17}N_3O_6S$: 308.0911. GSH counts: 6,650,626 (rt = 1.31 min). Arylated GSH (ESI$^+$) m/z of $[M+H]^+$ detected: 501.1693, expected for $C_{22}H_{28}N_4O_8S$: 501.1701. Arylated GSH counts: 1,764,547 (rt = 7.51 min). Note: GSH peak at 7.82 min likely due to degradation of conjugate.

Results of Pd(II)-Ar complex forming conditions C and D

C: Ar-H (15 µL, 40 mM solution in MeOH, 1.0 equiv.), Na_2PdCl_4 (15 µL, 40 mM solution in MeOH, 1.0 equiv.), NaOAc (15 µL, 40 mM solution in MeOH,1.0 equiv.) and MeOH (5 µL) were combined and stirred for 1 h at rt. After which, RuPhos (30 µL, 20 mM MeCN, 1.0 equiv.) was added, the reaction mixture was diluted with MeCN (420 µL) and stirred for another 1 h at rt, resulting in a stock solution of Pd(II) complex (500 µL, 1.2 mM). This stock solution was then subjected to the conditions of **general procedure** for glutathione labelling.

D: Ar-H (15 µL, 40 mM solution in MeOH, 1.0 equiv.), Na_2PdCl_4 (15 µL, 40 mM solution in MeOH, 1.0 equiv.), NaOAc (15 µL, 40 mM solution in MeOH,1.0 equiv.) and MeOH (5 µL) were combined and stirred for 1 h at rt. After which, Xantphos (30 µL, 20 mM DMF, 1.0 equiv.) was added, the reaction mixture was diluted with DMF (420 µL) and stirred for another 1 h at rt, resulting in a stock solution of Pd(II) complex (500 µL, 1.2 mM). This stock solution was then subjected to the conditions of **general procedure** for glutathione labelling.

Ar =	Ligand/organic solvent	Dilution corrected GSH counts	GSH Conversion
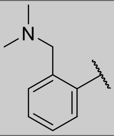	RuPhos/MeCN	666,901	93%
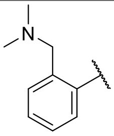	Xantphos/DMF	60,647	98%
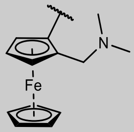	RuPhos/MeCN	3,988,540	66%
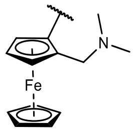	Xantphos/DMF	2,709,453	77%
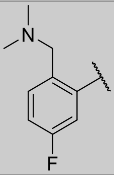	RuPhos/MeCN	1,404,054	87%
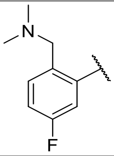	Xantphos/DMF	3,464,725	71%

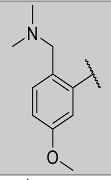	RuPhos/MeCN	170,258	97%
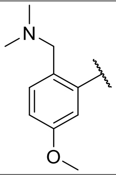	Xantphos/DMF	1,201,472	89%
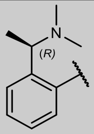	RuPhos/MeCN	6,345,336	47%
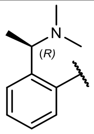	Xantphos/DMF	4,677,660	61%
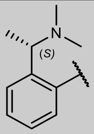	RuPhos/MeCN	5,000,751	58%
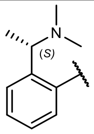	Xantphos/DMF	5,218,068	56%
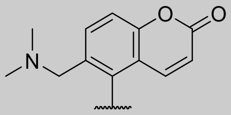	RuPhos/MeCN	3,382,310	71%
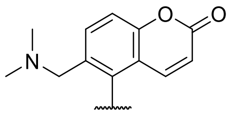	Xantphos/DMF	3,886,774	67%
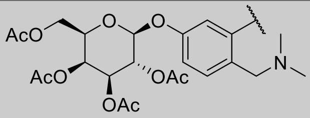	RuPhos/MeCN	1,781,986 (EIC 29)	84%
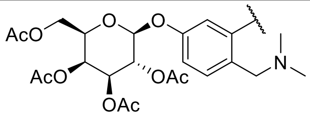	Xantphos/DMF	761,146 (EIC 30)	93%

*Table 4: Labelling efficiency of cyclopalladated Pd(II) complexes using Pd(II)-Ar complex forming conditions **C** and **D**.*

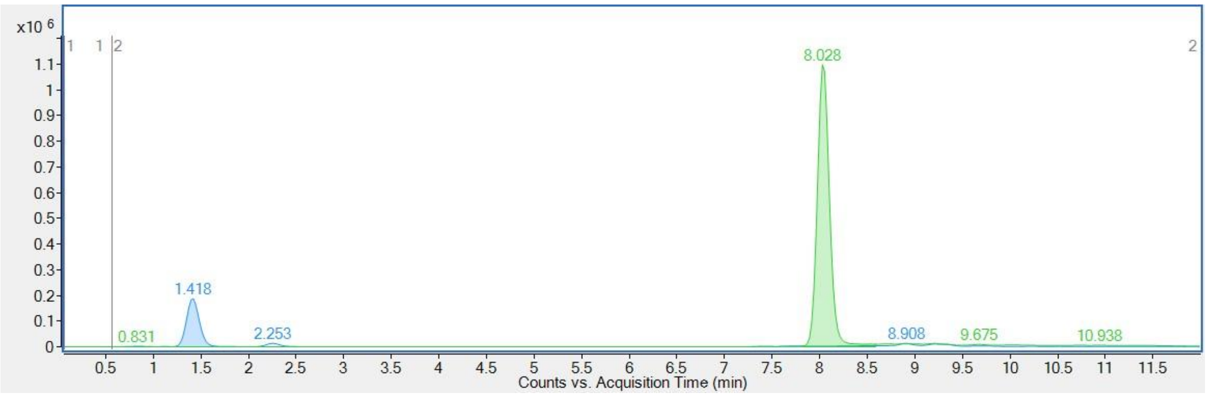

EIC 29: GSH (ESI⁺) m/z of [M+H]⁺ detected: 308.0907, expected for $C_{10}H_{17}N_3O_6S$: 308.0911. GSH counts: 1,781,986 (rt = 1.42 min). Arylated GSH (ESI⁺) m/z of [M+H]⁺ detected: 787.2692, expected for $C_{33}H_{46}N_4O_{16}S$: 787.2702. Arylated GSH counts: 9,841,686 (rt = 8.03 min).

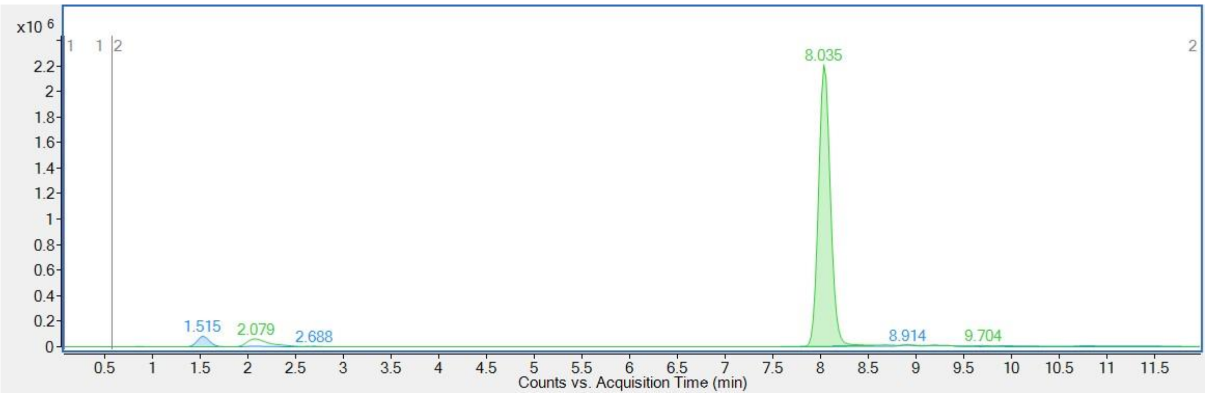

EIC 30: GSH (ESI⁺) m/z of [M+H]⁺ detected: 308.0908, expected for $C_{10}H_{17}N_3O_6S$: 308.0911. GSH counts: 761,146 (rt = 1.52 min). Arylated GSH (ESI⁺) m/z of [M+H]⁺ detected: 787.2695, expected for $C_{33}H_{46}N_4O_{16}S$: 787.2702. Arylated GSH counts: 19,177,474 (rt = 8.04 min).

Results of Pd(II)-Ar complex forming conditions E and F

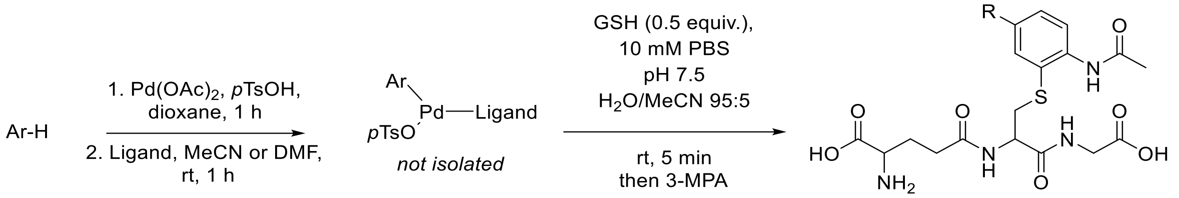

E: Ar-H (15 µL, 80 mM solution in dioxane, 2.0 equiv.), $Pd(OAc)_2$ (15 µL, 40 mM solution in dioxane, 1.0 equiv.), *p*TsOH (15 µL, 40 mM solution in dioxane) and dioxane (5 µL) were combined and stirred for 1 h at rt. After which, RuPhos (30 µL, 20 mM MeCN, 1.0 equiv.) was added, the reaction mixture was diluted with MeCN (420 µL) and stirred for another 1 h at rt, resulting in a stock solution of Pd(II) complex (500 µL, 1.2 mM). This stock solution was then subjected to the conditions of **general procedure** for glutathione labelling.

F: Ar-H (15 µL, 80 mM solution in dioxane, 2.0 equiv.), Pd(OAc)2 (15 µL, 40 mM solution in dioxane, 1.0 equiv.), pTsOH (15 µL, 40 mM solution in dioxane) and dioxane (5 µL) were combined and stirred for 1 h at rt. After which, Xantphos (30 µL, 20 mM DMF, 1.0 equiv.) was added, the reaction mixture was diluted with DMF (420 µL) and stirred for another 1 h at rt, resulting in a stock solution of Pd(II) complex (500 µL, 1.2 mM). This stock solution was then subjected to the conditions of general procedure for glutathione labelling.

Ar =	Ligand/organic solvent	Dilution corrected GSH counts	GSH Conversion	LC-MS EIC trace
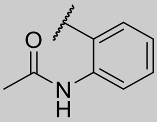	RuPhos/MeCN	1,459,491	87%	EIC 31
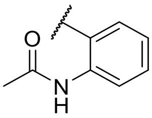	Xantphos/DMF	840,635	92%	EIC 32
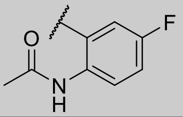	RuPhos/MeCN	1,036,095	90%	EIC 33
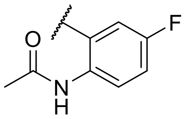	Xantphos/DMF	2,561,539	78%	EIC 34
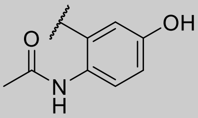	RuPhos/MeCN	288,511	96%	EIC 35
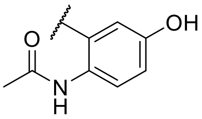	Xantphos/DMF	1,911,222	83%	EIC 36

Table 5: Labelling efficiency of cyclopalladated Pd(II) complexes using Pd(II)-Ar complex forming conditions ***E*** *and* ***F****.*

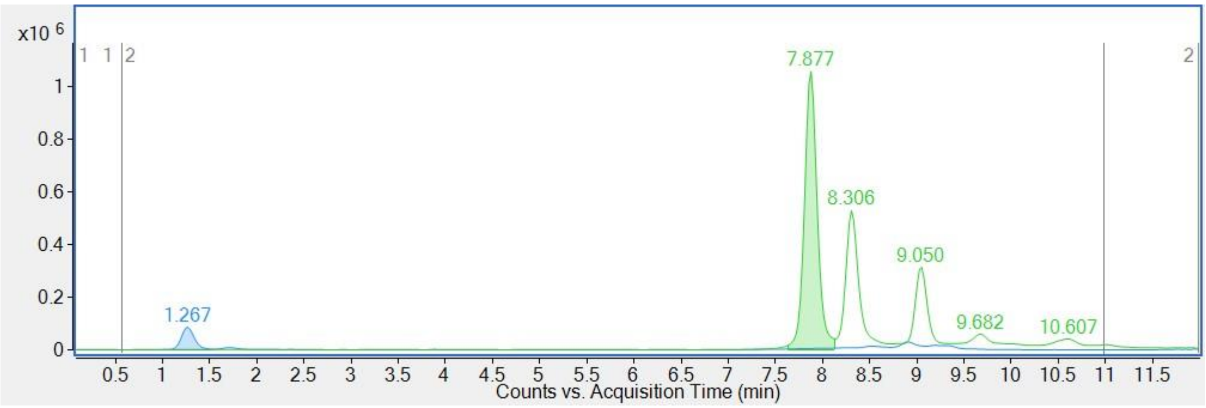

EIC 31: GSH (ESI+) m/z of [M+H]+ detected: 308.0907, expected for $C_{10}H_{17}N_3O_6S$: 308.0911. GSH counts: 1,459,491 (rt = 1.27 min). Arylated GSH (ESI+) m/z of [M+H]+ detected:441.1438, expected for $C_{18}H_{24}N_4O_7S$: 441.1438. Arylated GSH counts: 10,040,154 (rt = 7.88 min).

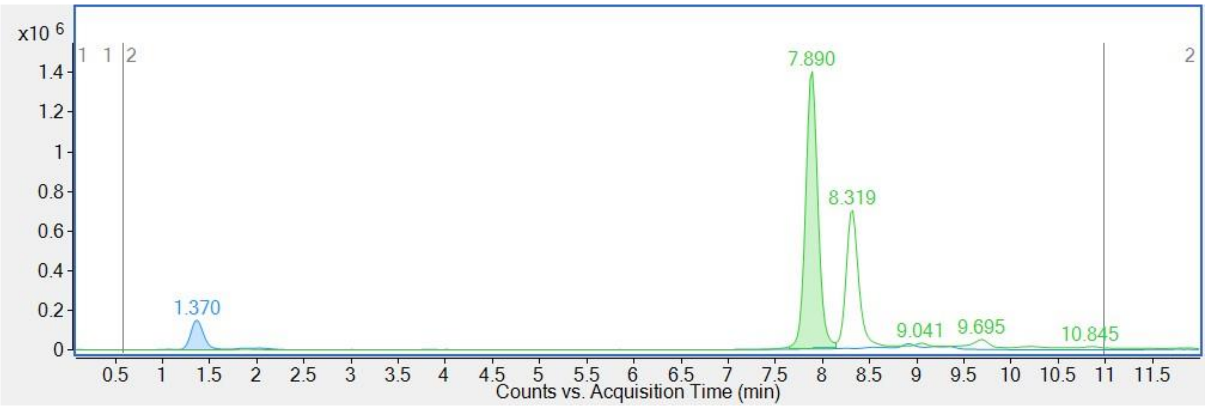

EIC 32: GSH (ESI+) m/z of [M+H]+ detected: 308.0908, expected for $C_{10}H_{17}N_3O_6S$: 308.0911. GSH counts: 1,459,491 (rt = 1.27 min). Arylated GSH (ESI+) m/z of [M+H]+ detected:441.1437, expected for $C_{18}H_{24}N_4O_7S$: 441.1438. Arylated GSH counts: 10,040,154 (rt = 7.88 min).

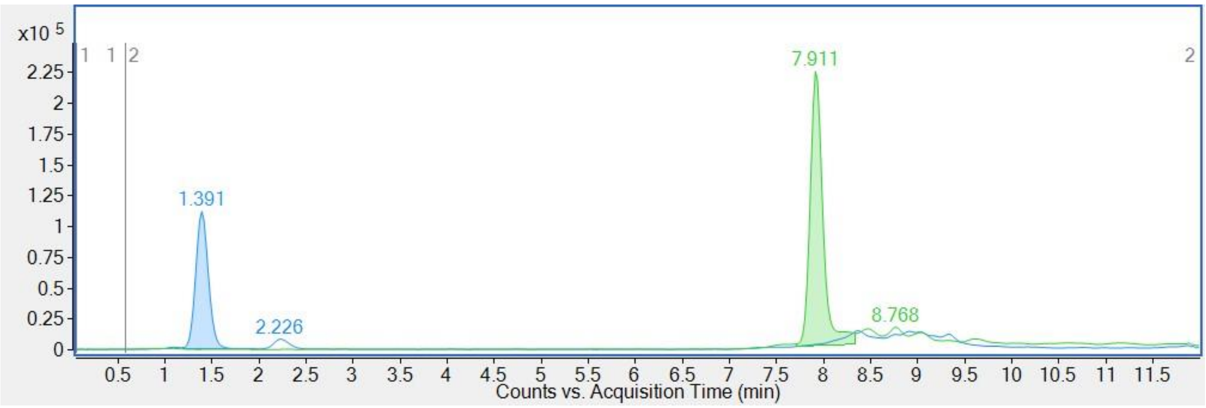

EIC 33: GSH (ESI+) m/z of [M+H]+ detected: 308.0904, expected for $C_{10}H_{17}N_3O_6S$: 308.0911. GSH counts: 1,036,095 (rt = 1.39 min). Arylated GSH (ESI+) m/z of [M+H]+ detected:459.1339, expected for $C_{18}H_{23}FN_4O_7S$: 459.1344. Arylated GSH counts: 2,165,093 (rt = 7.91 min).

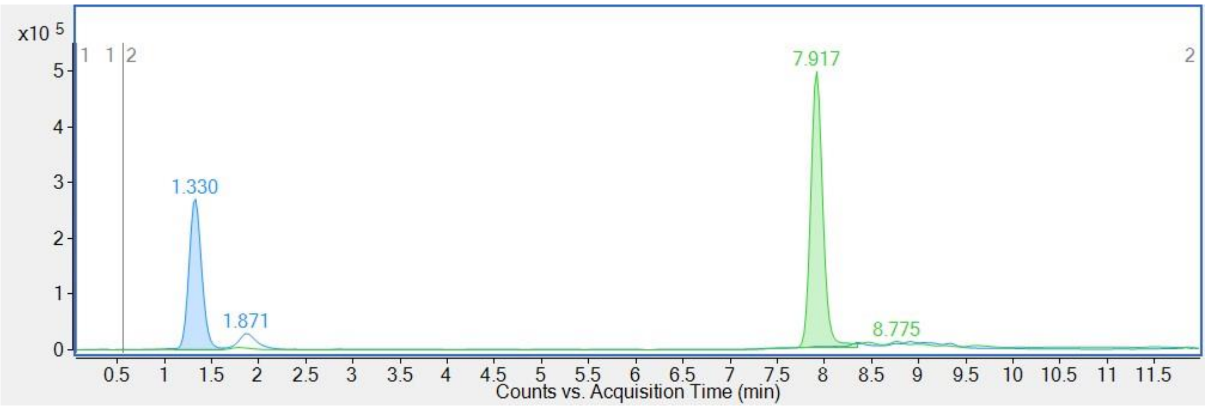

EIC 34: GSH (ESI^+) m/z of $[M+H]^+$ detected: 308.0904, expected for $C_{10}H_{17}N_3O_6S$: 308.0911. GSH counts: 2,561,539 (rt = 1.33 min). Arylated GSH (ESI^+) m/z of $[M+H]^+$ detected:459.1341, expected for $C_{18}H_{23}FN_4O_7S$: 459.1344. Arylated GSH counts: 2,561,539 (rt = 7.92 min).

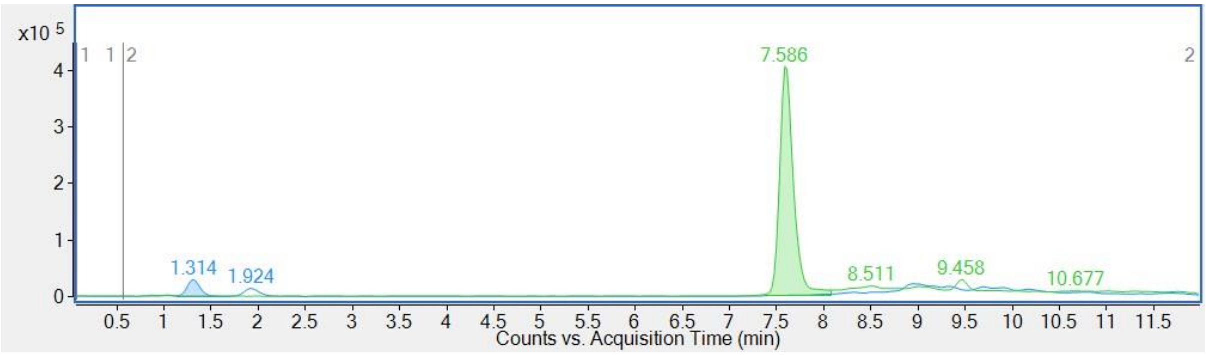

EIC 35: GSH (ESI^+) m/z of $[M+H]^+$ detected: 308.0904, expected for $C_{10}H_{17}N_3O_6S$: 308.0911. GSH counts: 288,511 (rt = 1.31 min). Arylated GSH (ESI^+) m/z of $[M+H]^+$ detected:457.1383, expected for $C_{18}H_{24}N_4O_8S$: 457.1388. Arylated GSH counts: 4,228,258 (rt = 7.59 min).

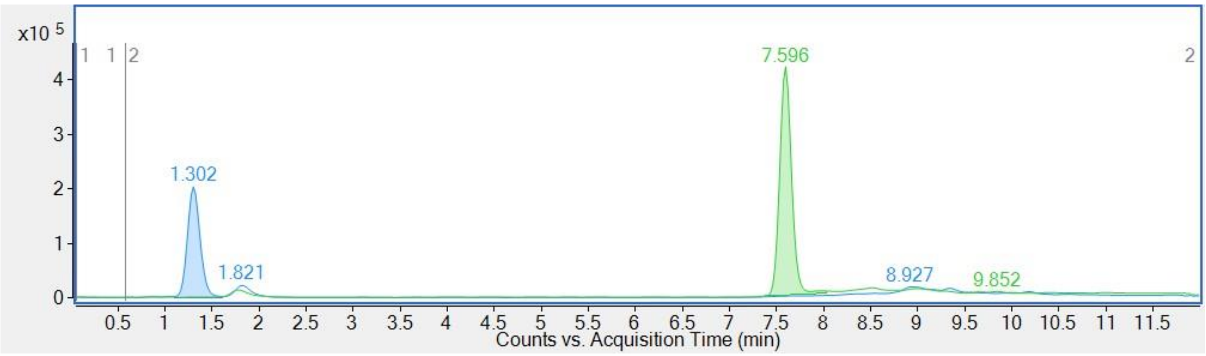

EIC 36: GSH (ESI^+) m/z of $[M+H]^+$ detected: 308.0904, expected for $C_{10}H_{17}N_3O_6S$: 308.0911. GSH counts: 1,911,222 (rt = 1.30 min). Arylated GSH (ESI^+) m/z of $[M+H]^+$ detected:457.1383, expected for $C_{18}H_{24}N_4O_8S$: 457.1388. Arylated GSH counts: 3,690,628 (rt = 7.60 min).

Labelling of BSA using *in situ* generated Pd(II) complexes

N,*N*-dimethylbenzylamine analogue (**16** or **22**) (25 µL, 80 mM in MeOH), NaOAc (25 µL, 80 mM in MeOH) and Na_2PdCl_4 (50 µL, 40 mM in MeOH) were combined in a 1.5 mL Eppendorf tube and vortexed. After 1 h, phosphine ligand (100 µL, 20 mM in MeCN or DMF) was added, the reaction was vortexed, and left to stand for 1 h to form a 10 mM stock solution of Pd(II) complex.

Pd(II) stock solution (10 µL, 10 mM in MeOH/MeCN or DMF 1:1, 10 equiv.) was added to a mixture of BSA (10 µL, 1 mM in H_2O, 1 equiv.) and PBS (80 µL, Dulbecco A) in a 1.5 mL Eppendorf tube. After vortexing, the reaction mixture was held at 37 °C for 1 h. An aliquot (15 µL) was taken and combined with trypsin (*50:1 protein/trypsin)* in NH_4HCO_3 (85 µL, 50 mM). After vortexing, the reaction mixture was held at 37 °C for 20 h, and then analysed by **peptide MS/MS**.

n.b. if RuPhos was used, co-solvent was MeCN. If Xantphos was used, co-solvent was DMF.

Estimation of conversions:

Since only arylated Cys-34 (confirmed by MS/MS) was detected, conversions to arylated BSA are estimated by examining the EIC counts of the modified and unmodified Cys-34 containing peptide (H-GLVLIAFSQYLQQCPFDEHVK-OH) following trypsin digest. Conversions are estimated as follows: $\frac{\text{Counts(modified)}}{\text{Counts(total)}} \times 100$. Counts determined from EICs. Data was processed in Agilent Masshunter Qualitative Analysis B.07.00.

Compound	Ligand	Unmodified peptide EIC counts	Modified peptide EIC counts	Estimated conversion
16	RuPhos	980,095	43,031,491	98%
16	Xantphos	73,763	11,164,356	99%
22	RuPhos	1,426,750	18,339,359	93%
22	Xantphos	1,341,270	13,691,649	91%

Table 6: Estimation of conversion of BSA to arylated BSA at Cys-34.

Compound 16: Ligand = RuPhos

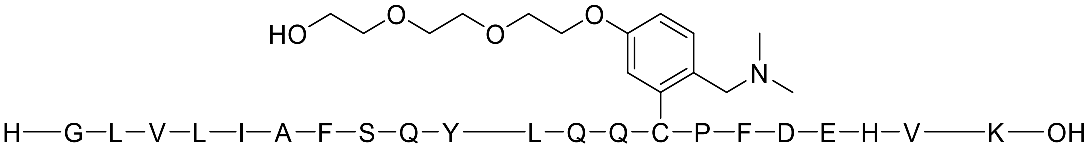

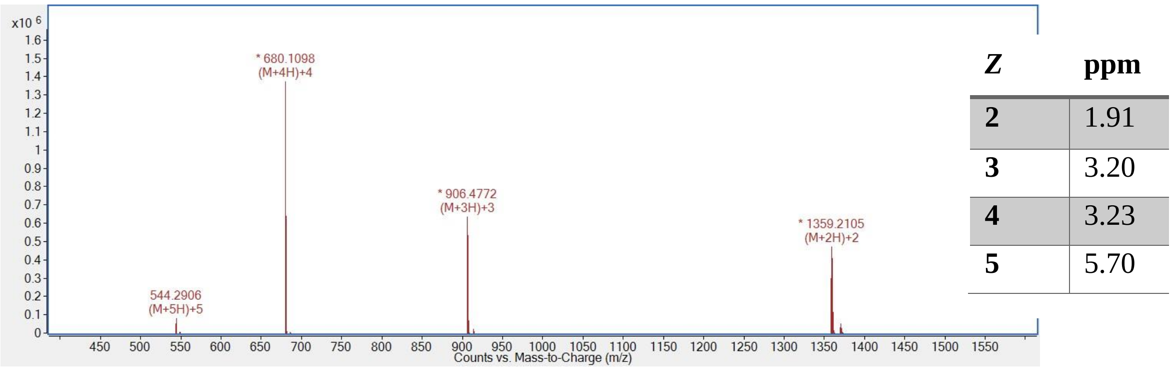

Z	ppm
2	1.91
3	3.20
4	3.23
5	5.70

Figure 2: MS spectrum of labelled GLVLIAFSQYLQQCPFDEHVK with ppm errors for parent ions.

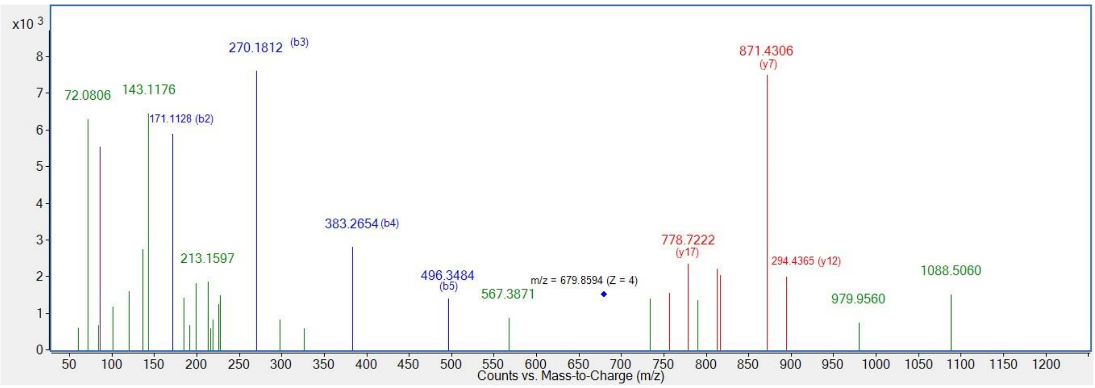

Figure 3: MS/MS spectrum from (Z = 4) parent ion.

Sequence	m/z	m/z (predicted)	Diff (ppm)	Z (prod.)	Ion
V	**72.0806**	**72.0808**	**-2.7**	**1**	**V**
L	**86.0963**	**86.0964**	**-1.4**	**1**	**L**
I	**86.0963**	**86.0964**	**-1.4**	**1**	**I**
Q	**101.0703**	**101.0709**	**-5.9**	**1**	**Q**
Y	**136.0752**	**136.0757**	**-3.4**	**1**	**Y**
GL	**171.1128**	**171.1128**	**-0.1**	**1**	**b2**
GLV	**270.1812**	**270.1812**	**-0.2**	**1**	**b3**
GLVL	**383.2654**	**383.2653**	**0.4**	**1**	**b4**
GLVLI	**496.3484**	**496.3493**	**-2**	**1**	**b5**
QQCPFDEHVK	**756.3652**	**756.3636**	**2.2**	**2**	**y10**
IAFSQYLQQCPFDEHVK	**778.7222**	**778.7207**	**2**	**3**	**y17**
LQQCPFDEHVK	**812.9065**	**812.9056**	**1.1**	**2**	**y11**
LIAFSQYLQQCPFDEHVK	**816.4168**	**816.4154**	**1.7**	**3**	**y18**
PFDEHVK	**871.4306**	**871.4308**	**-0.2**	**1**	**y7**
YLQQCPFDEHVK	**894.4365**	**894.4373**	**-0.8**	**2**	**y12**

Table 7: Confirmation of b and y ions.

Compound 16: Ligand = Xantphos

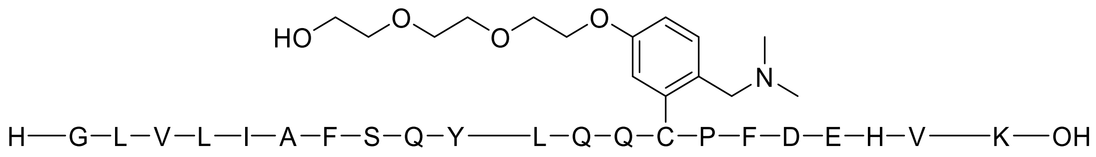

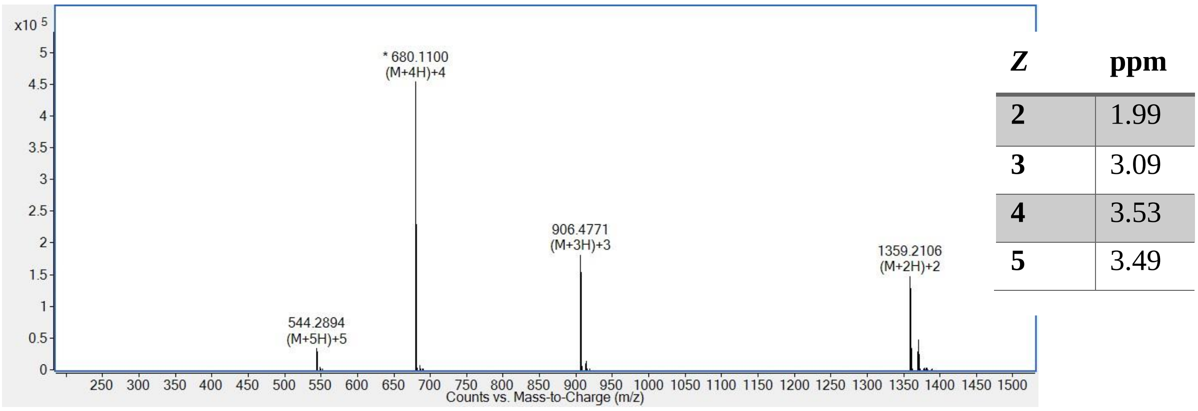

Z	ppm
2	1.99
3	3.09
4	3.53
5	3.49

Figure 4: MS spectrum of labelled GLVLIAFSQYLQQCPFDEHVK with ppm errors for parent ions.

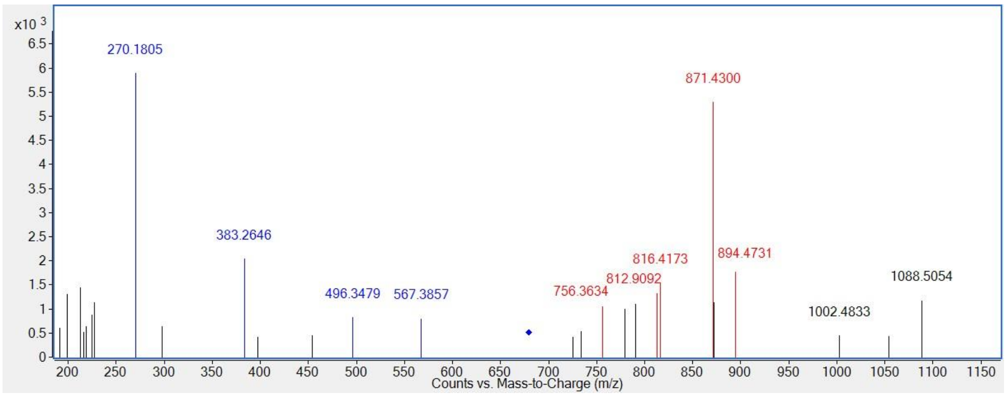

Figure 5: MS/MS spectrum from (Z = 4) parent ion.

Sequence	m/z	m/z (predicted)	Diff (ppm)	Z (prod.)	Ion
V	**72.0803**	**72.0808**	**-5.9**	**1**	**V**
L	**86.0962**	**86.0964**	**-2.1**	**1**	**L**
I	**86.0962**	**86.0964**	**-2.1**	**1**	**I**
Q	**101.0704**	**101.0709**	**-5.1**	**1**	**Q**
Y	**136.0752**	**136.0757**	**-3.6**	**1**	**Y**
GL	**171.1126**	**171.1128**	**-1.4**	**1**	**b2**
GLV	**270.1805**	**270.1812**	**-2.5**	**1**	**b3**
GLVL	**383.2646**	**383.2653**	**-1.8**	**1**	**b4**
GLVLI	**496.3479**	**496.3493**	**-2.9**	**1**	**b5**
GLVLIA	**567.3857**	**567.3865**	**-1.3**	**1**	**b6**
QQCPFDEHVK	**756.3634**	**756.3636**	**-0.2**	**2**	**y10**
LQQCPFDEHVK	**812.9092**	**812.9056**	**4.5**	**2**	**y11**
LIAFSQYLQQCPFDEHVK	**816.4173**	**816.4154**	**2.4**	**3**	**y18**
PFDEHVK	**871.43**	**871.4308**	**-0.9**	**1**	**y7**
YLQQCPFDEHVK	**894.4371**	**894.4373**	**-0.2**	**2**	**y12**

Table 8: Confirmation of b and y ions.

Compound 22: Ligand = RuPhos

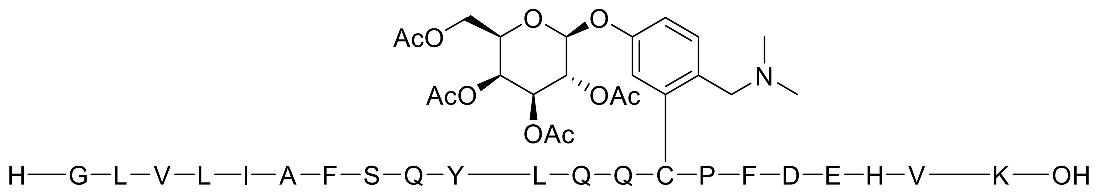

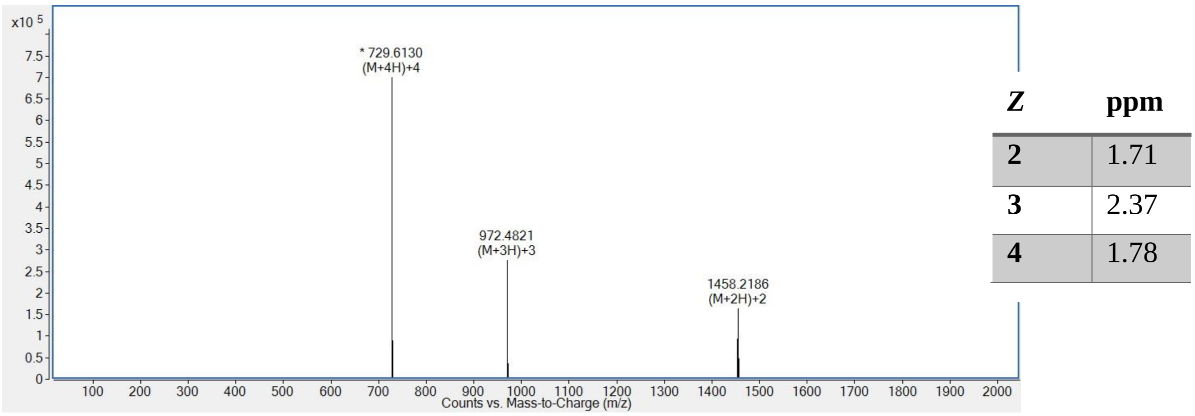

Z	ppm
2	1.71
3	2.37
4	1.78

Figure 6: MS spectrum of labelled GLVLIAFSQYLQQCPFDEHVK with ppm errors for parent ions.

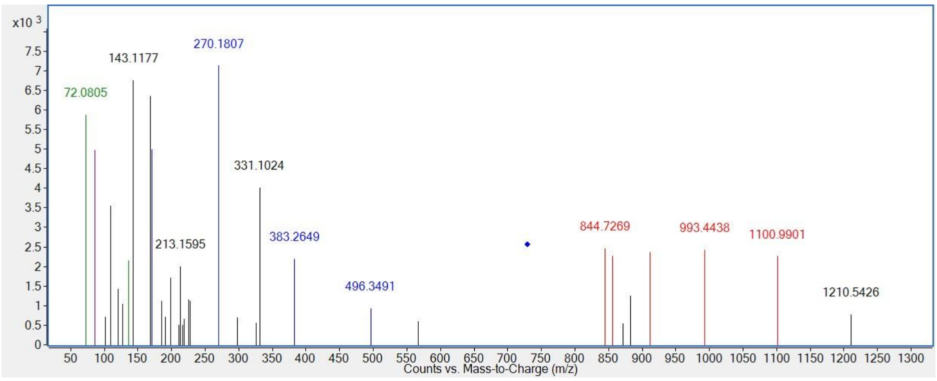

Figure 7: MS/MS spectrum from (Z = 4) parent ion.

Sequence	m/z	m/z (predicted)	Diff ppm)	Z (prod.)	Ion
V	**72.0805**	**72.0808**	**-3.3**	**1**	**V**
L	**86.0963**	**86.0964**	**-1.6**	**1**	**L**
I	**86.0963**	**86.0964**	**-1.6**	**1**	**I**
Y	**136.0754**	**136.0757**	**-1.8**	**1**	**Y**
GL	**171.1124**	**171.1128**	**-2.6**	**1**	**b2**
GLV	**270.1807**	**270.1812**	**-2**	**1**	**b3**
GLVL	**383.2649**	**383.2653**	**-0.9**	**1**	**b4**
GLVLI	**496.3491**	**496.3493**	**-0.6**	**1**	**b5**
IAFSQYLQQCPFDEHVK	**844.7269**	**844.7262**	**0.9**	**3**	**y17**
QQCPFDEHVK	**855.3713**	**855.3718**	**-0.6**	**2**	**y10**
LQQCPFDEHVK	**911.9146**	**911.9138**	**0.9**	**2**	**y11**
YLQQCPFDEHVK	**993.4438**	**993.4455**	**-1.7**	**2**	**y12**
SQYLQQCPFDEHVK	**1100.9901**	**1100.9908**	**-0.6**	**2**	**y14**

Table 9: Confirmation of b and y ions.

Compound 22: Ligand = Xantphos

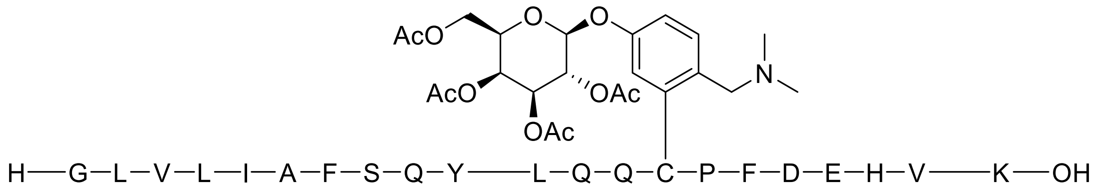

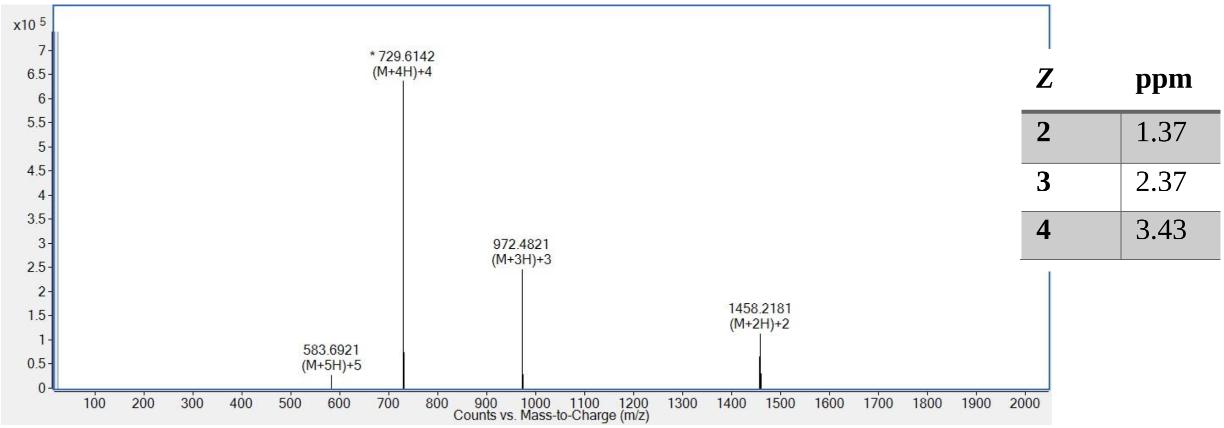

Z	ppm
2	1.37
3	2.37
4	3.43

Figure 8: MS spectrum of labelled GLVLIAFSQYLQQCPFDEHVK with ppm errors for parent ions.

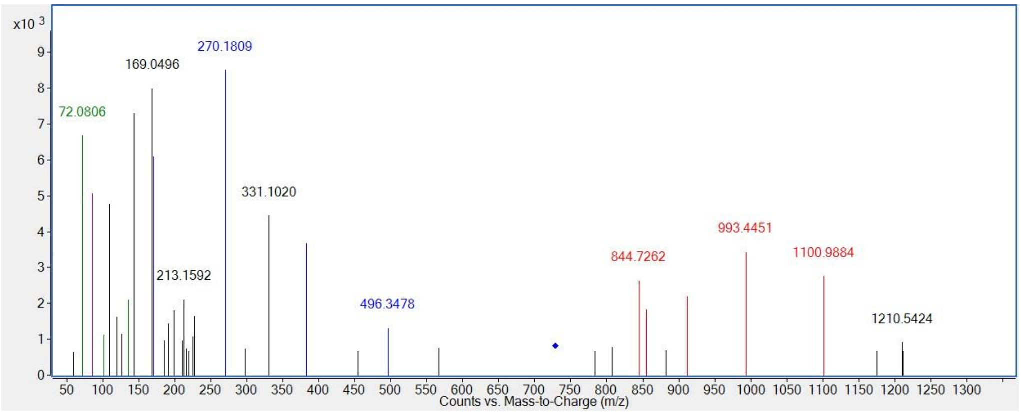

Figure 9: MS/MS spectrum from (Z = 4) parent ion.

Sequence	m/z	m/z (predicted)	Diff (ppm)	Z (prod.)	Ion
	**72.0806**	**72.0808**	**-2.6**	**1**	**V**
	**86.0964**	**86.0964**	**-0.5**	**1**	**L**
	**86.0964**	**86.0964**	**-0.5**	**1**	**I**
	**101.0709**	**101.0709**	**-0.8**	**1**	**Q**
	**136.0757**	**136.0757**	**0.3**	**1**	**Y**
GL	**171.1122**	**171.1128**	**-3.7**	**1**	**b2**
GLV	**270.1809**	**270.1812**	**-1.3**	**1**	**b3**
GLVL	**383.2647**	**383.2653**	**-1.4**	**1**	**b4**
GLVLI	**496.3478**	**496.3493**	**-3.1**	**1**	**b5**
IAFSQYLQQCPFDEHVK	**844.7262**	**844.7262**	**0.1**	**3**	**y17**
QQCPFDEHVK	**855.3708**	**855.3718**	**-1.2**	**2**	**y10**
LQQCPFDEHVK	**911.9118**	**911.9138**	**-2.2**	**2**	**y11**
YLQQCPFDEHVK	**993.4451**	**993.4455**	**-0.4**	**2**	**y12**
SQYLQQCPFDEHVK	**1100.9884**	**1100.9908**	**-2.2**	**2**	**y14**

Table 10: Confirmation of b and y ions.

Stability of cyclopalladated complexes

All Pd(II) complexes were stored in closed glass vials at ambient temperature.

(dmba)Pd(II)Cl(RuPhos)

The stability of (dmba)Pd(II)(RuPhos) was assessed at 8 months by ^{1}H and ^{31}P NMR in $CDCl_3$. **General procedure** for glutathione labelling was used to assess labelling efficiency after 8 months. Dilution corrected GSH EIC peak area: 441,249: 95% conversion of GSH.

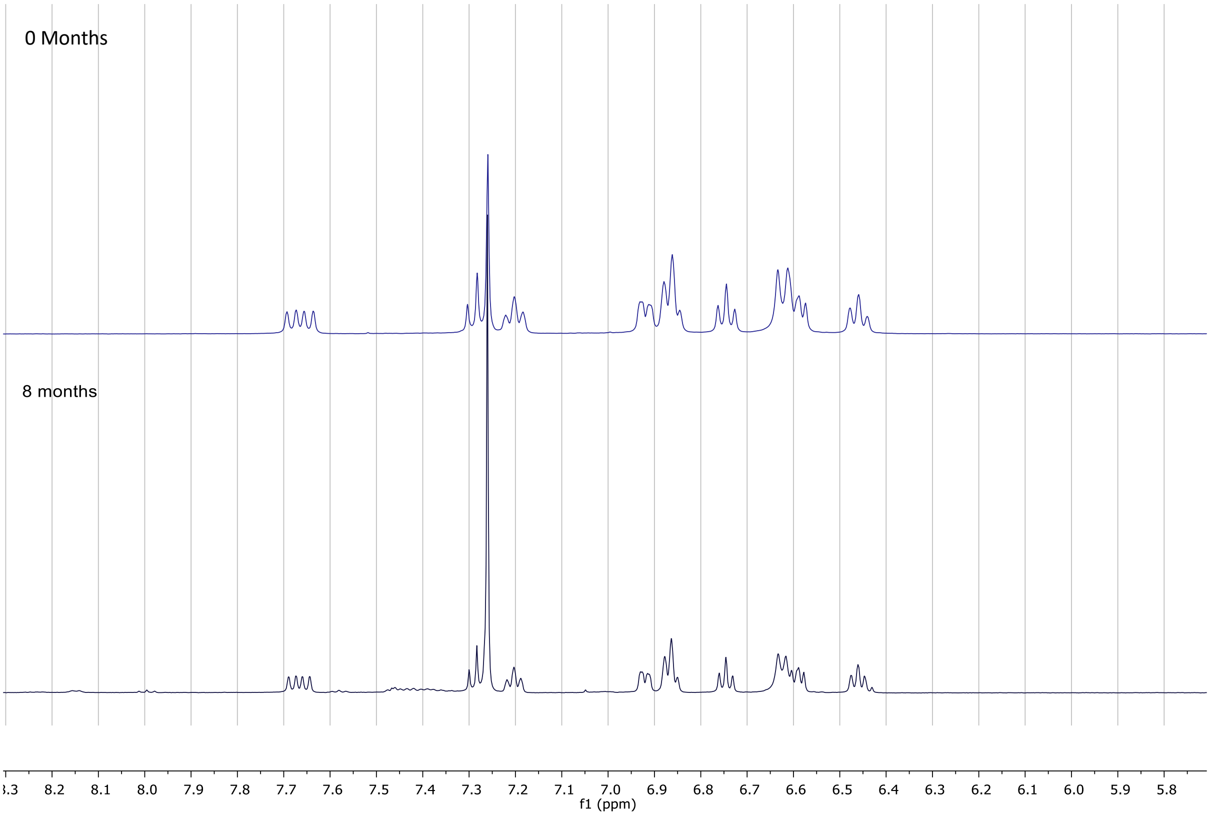

Figure 10: ^{1}H NMR spectra in $CDCl_3$ of newly synthesised (dmba)Pd(II)Cl(RuPhos) vs. 8 month 'aged' complex. Only the aromatic region is shown for clarity. Spectra overlayed using MestReNova v11.0.2-18153.

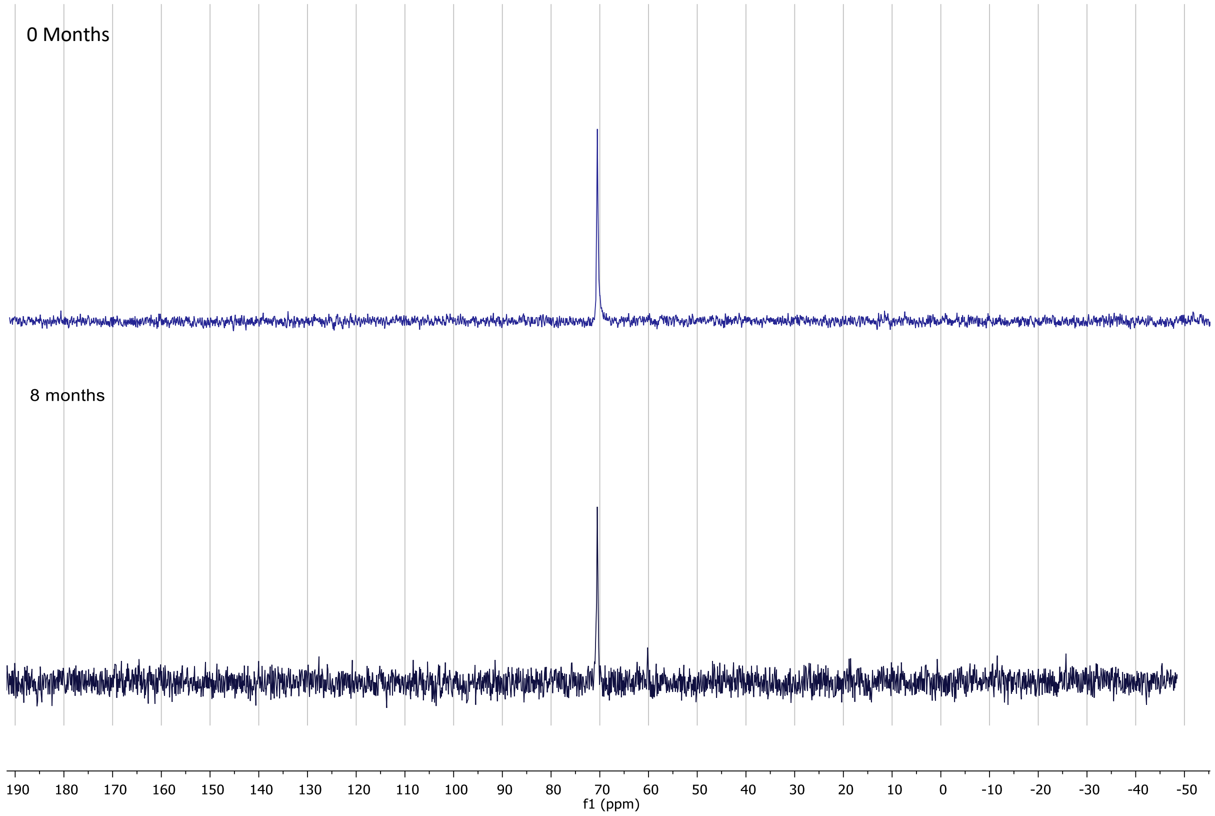

Figure 11: ^{31}P NMR spectra in $CDCl_3$ of newly synthesised (dmba)Pd(II)Cl(RuPhos) vs. 8 month 'aged' complex. Spectra overlayed using MestReNova v11.0.2-18153.

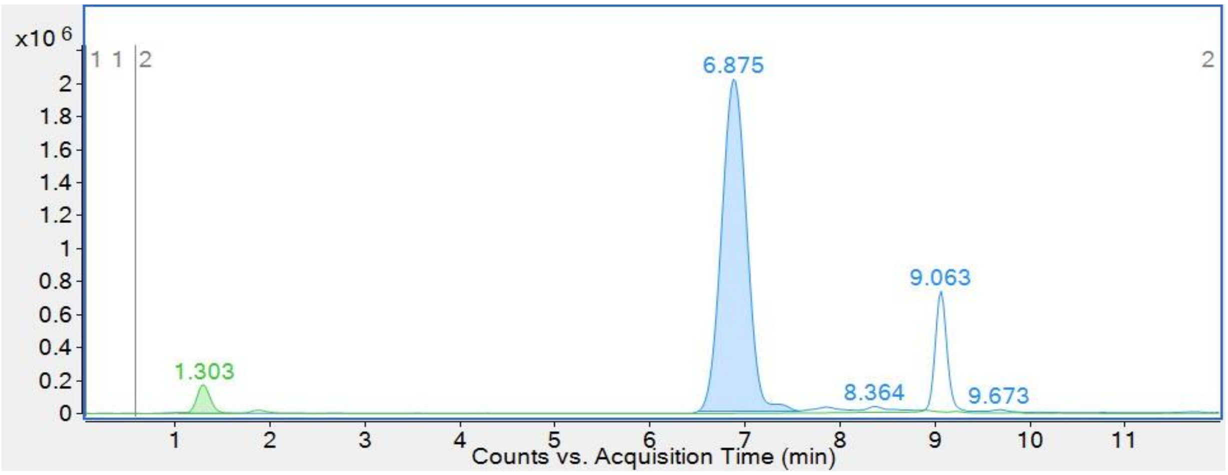

EIC 37: GSH EIC peak area: 1,766,742 (Rt = 1.30 min), arylated GSH EIC peak area: 37,181,391 (Rt = 6.88 min).

(dmba)Pd(II)(Xantphos)

The stability of (dmba)Pd(II)(Xantphos) was assessed at 8 months by ^{1}H and ^{31}P NMR in $CDCl_3$ at 323 K. **General procedure** for glutathione labelling was used to assess labelling efficiency after 8 months. Dilution corrected GSH EIC peak area: 834,735: 92% conversion of GSH.

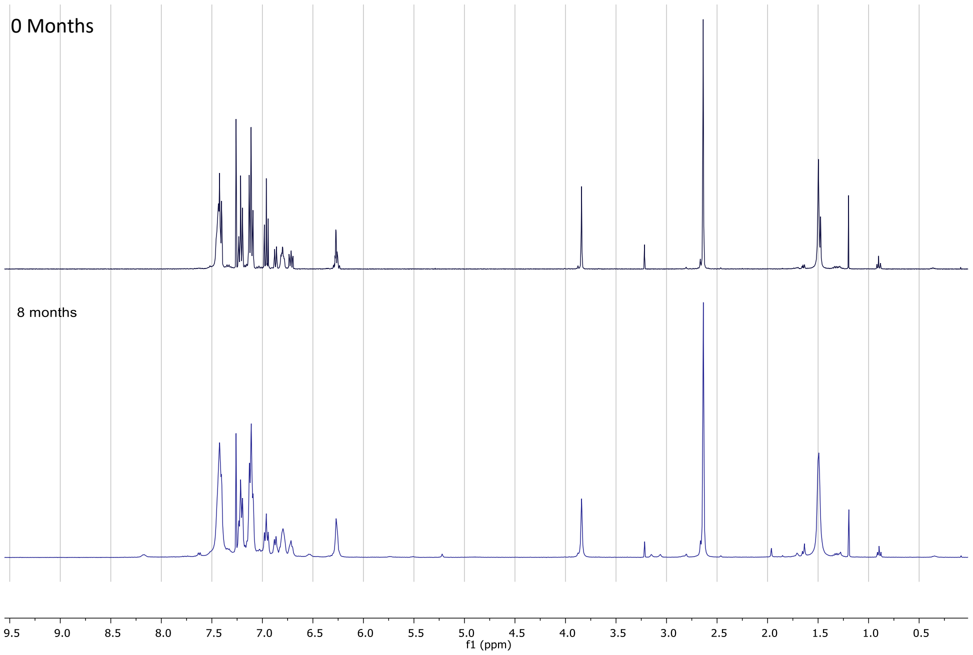

Figure 12: ^{1}H NMR spectra in $CDCl_3$ at 323 K of newly synthesised (dmba)Pd(II)Cl(Xantphos) vs. 8 month 'aged' complex. Spectra overlayed using MestReNova v11.0.2-18153.

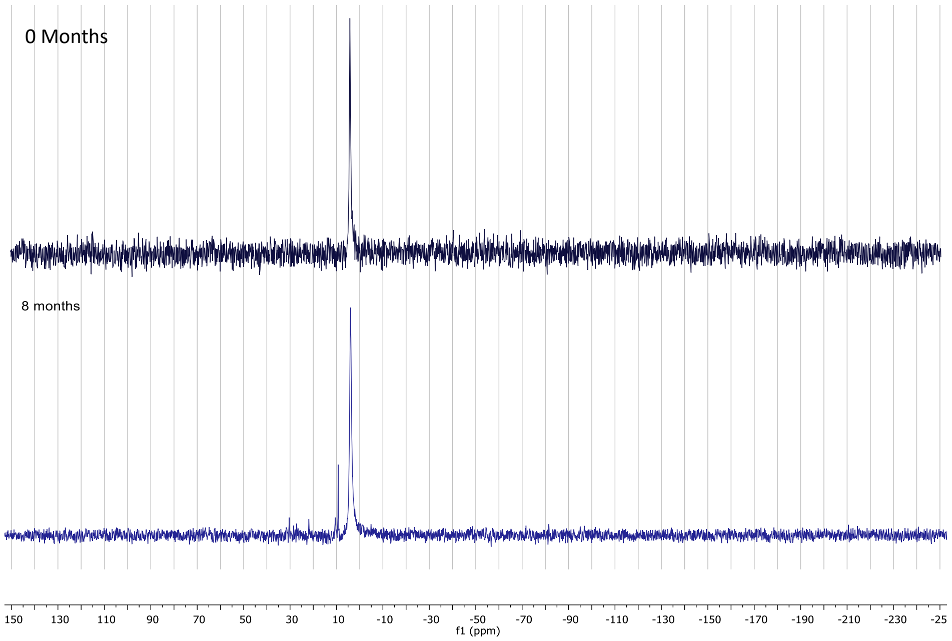

Figure 13: ^{31}P NMR spectra in $CDCl_3$ at 323 K of newly synthesised (dmba)Pd(II)Cl(Xantphos) vs. 8 month 'aged' complex. Spectra overlayed using MestReNova v11.0.2-18153.

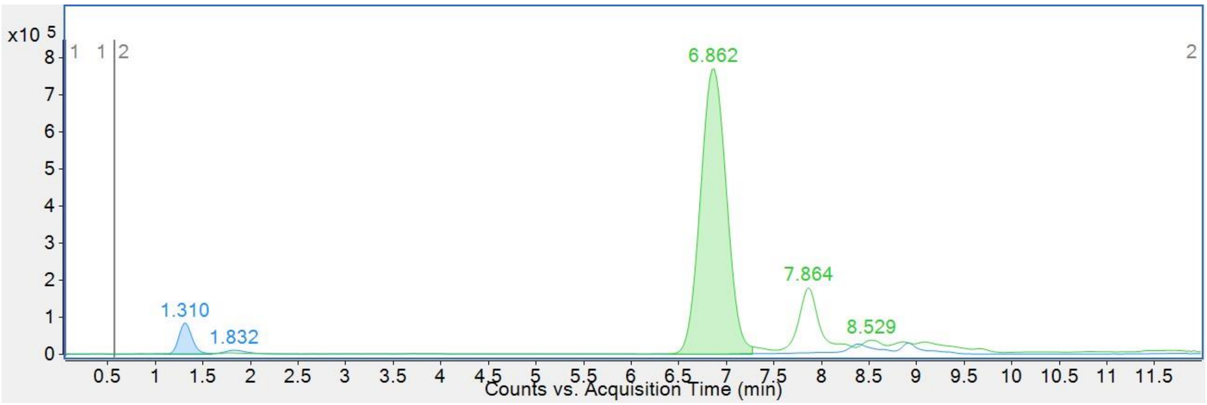

EIC 38: 8 months 'aged' (dmba)Pd(II)Cl(Xantphos) GSH EIC peak area: 834,735 (Rt = 1.31 min), arylated GSH EIC peak area: 14,680,068 (Rt = 6.88 min).

NMR spectra

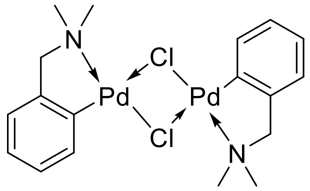

^{1}H (400 MHz, $CDCl_3$)

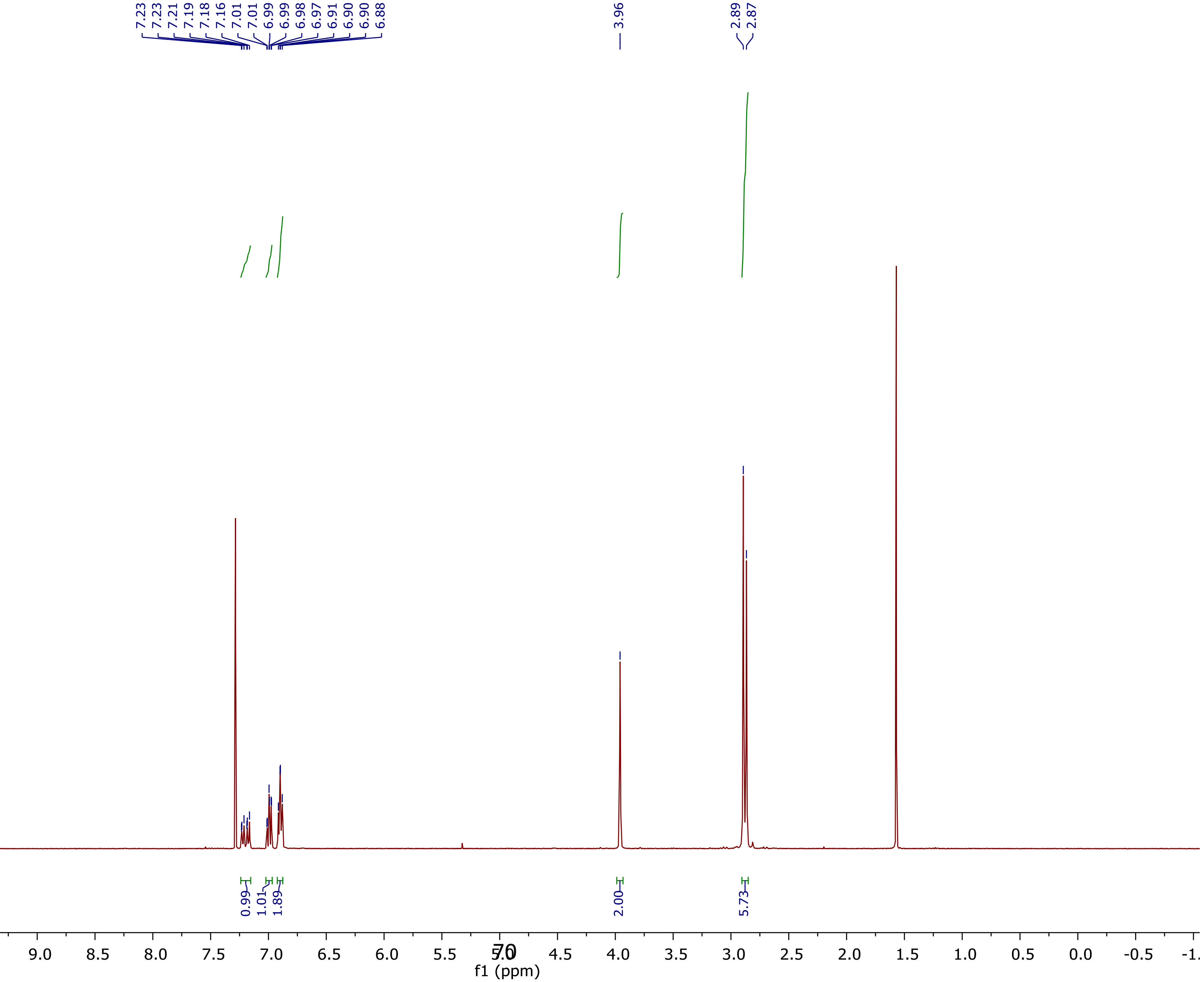

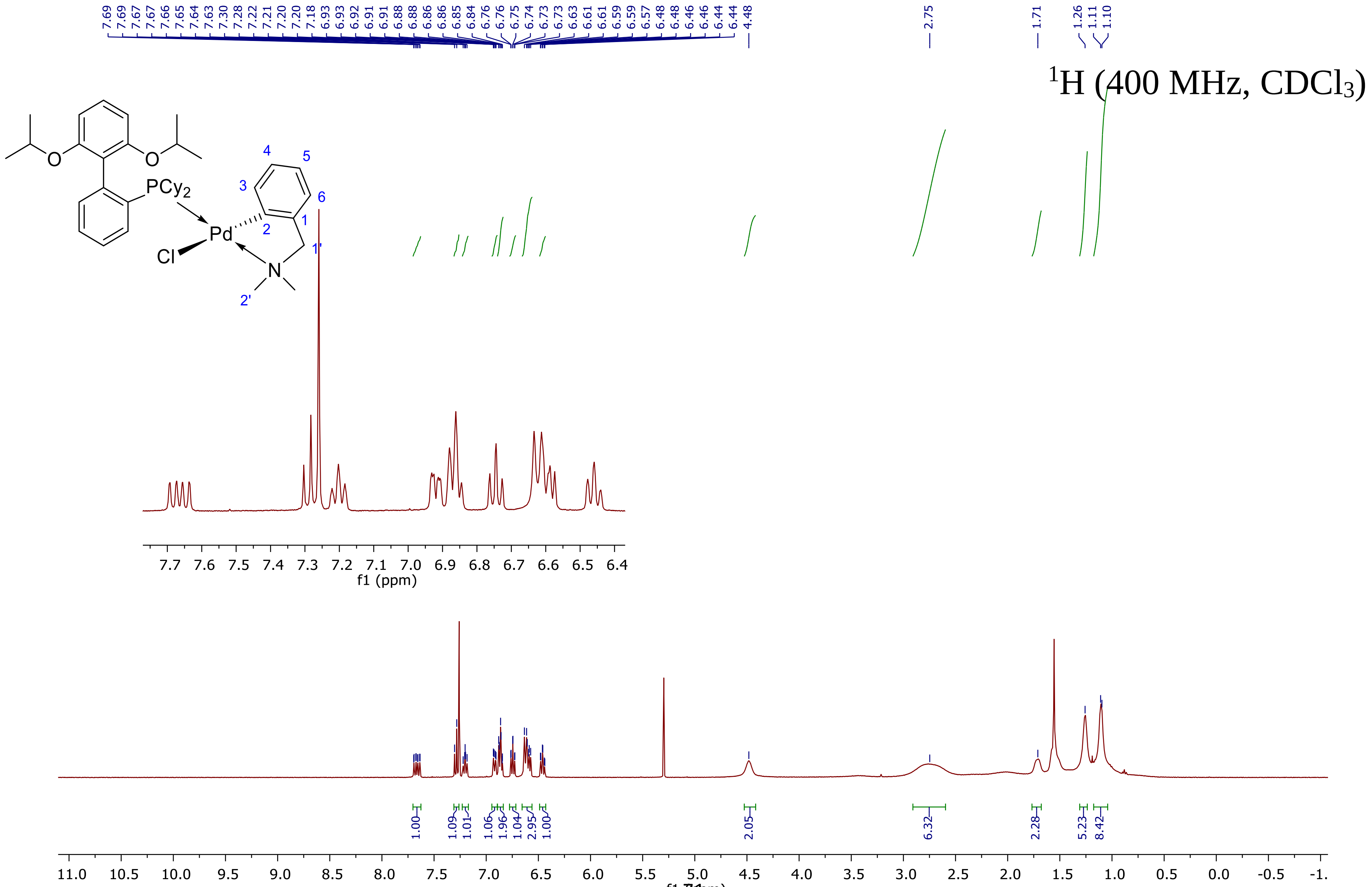

^{1}H (400 MHz, CDCl$_3$)
7.69 7.69 7.67 7.67 7.66 7.65 7.64 7.63 7.30 7.28 7.22 7.21 7.20 7.20 7.18 6.93 6.93 6.92 6.91 6.91 6.88 6.88 6.86 6.86 6.85 6.84 6.76 6.76 6.75 6.74 6.73 6.73 6.63 6.61 6.61 6.59 6.59 6.57 6.48 6.48 6.46 6.46 6.44 6.44
4.48
2.75
1.71
1.26 1.11 1.10
PCy2
Pd
Cl
N
O
O
1 2 3 4 5 6 1' 2'
1.00 1.09 1.01 1.06 1.96 1.04 2.95 1.00 2.05 6.32 2.28 5.23 8.42
7.7 7.6 7.5 7.4 7.3 7.2 7.1 7.0 6.9 6.8 6.7 6.6 6.5 6.4
f1 (ppm)
11.0 10.5 10.0 9.5 9.0 8.5 8.0 7.5 7.0 6.5 6.0 5.5 5.0 4.5 4.0 3.5 3.0 2.5 2.0 1.5 1.0 0.5 0.0 -0.5 -1.
f1 (ppm)

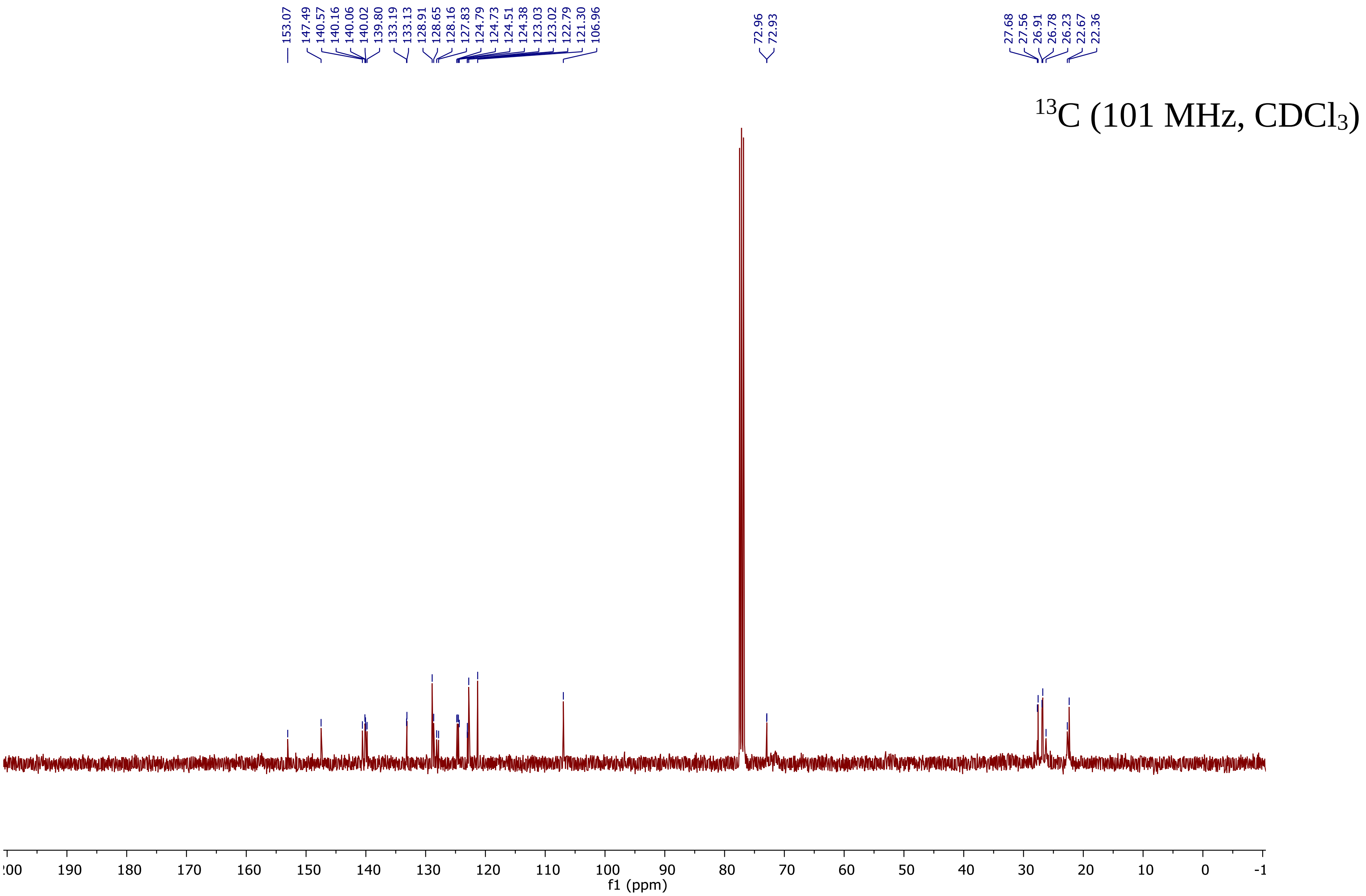
153.07
147.49
140.57
140.16
140.06
140.02
139.80
133.19
133.13
128.91
128.65
128.16
127.83
124.79
124.73
124.51
124.38
123.03
123.02
122.79
121.30
106.96
72.96
72.93
27.68
27.56
26.91
26.78
26.23
22.67
22.36
^{13}C (101 MHz, $CDCl_3$)
f1 (ppm)

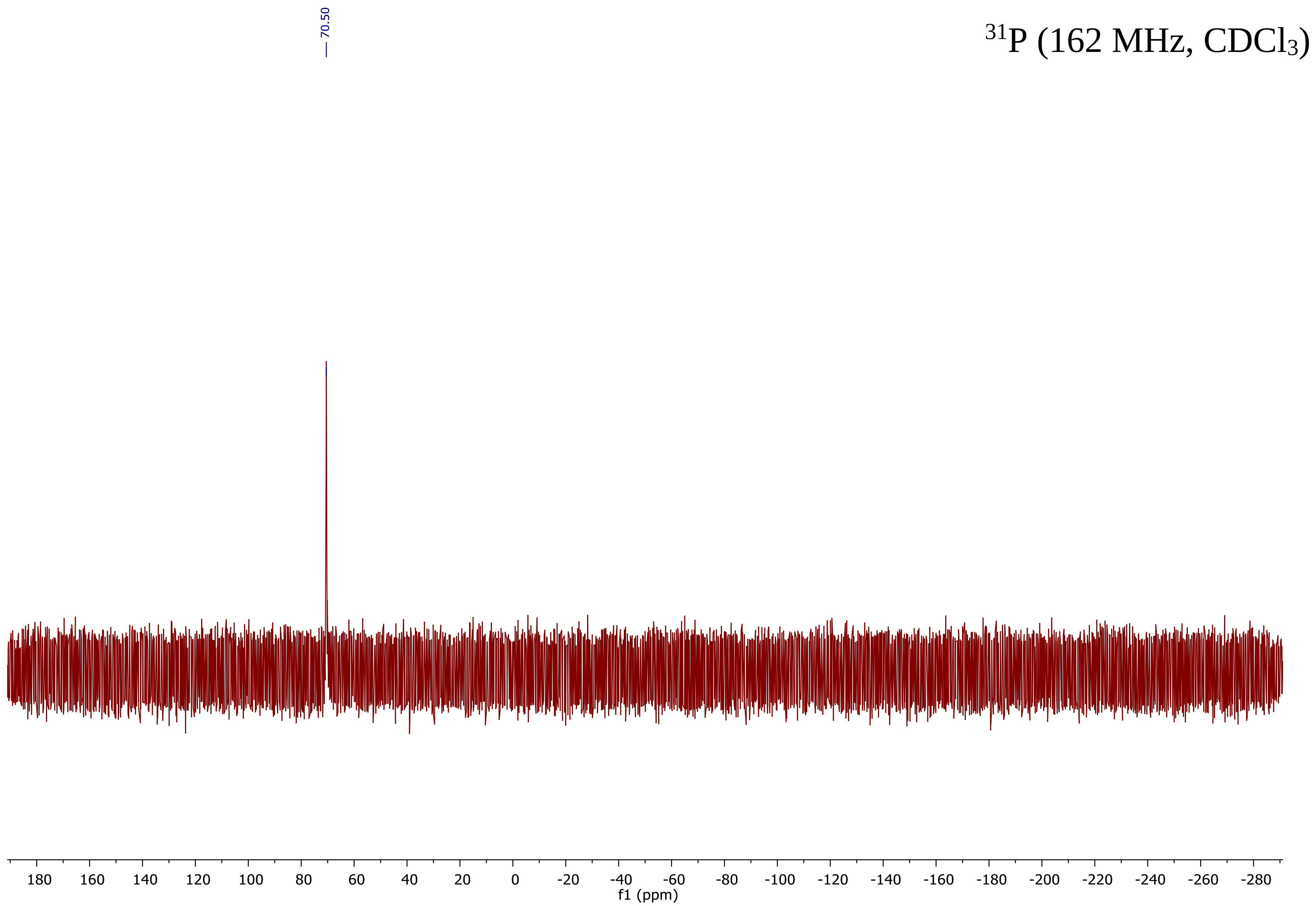

70.50
^{31}P (162 MHz, $CDCl_3$)
180 160 140 120 100 80 60 40 20 0 -20 -40 -60 -80 -100 -120 -140 -160 -180 -200 -220 -240 -260 -280
f1 (ppm)

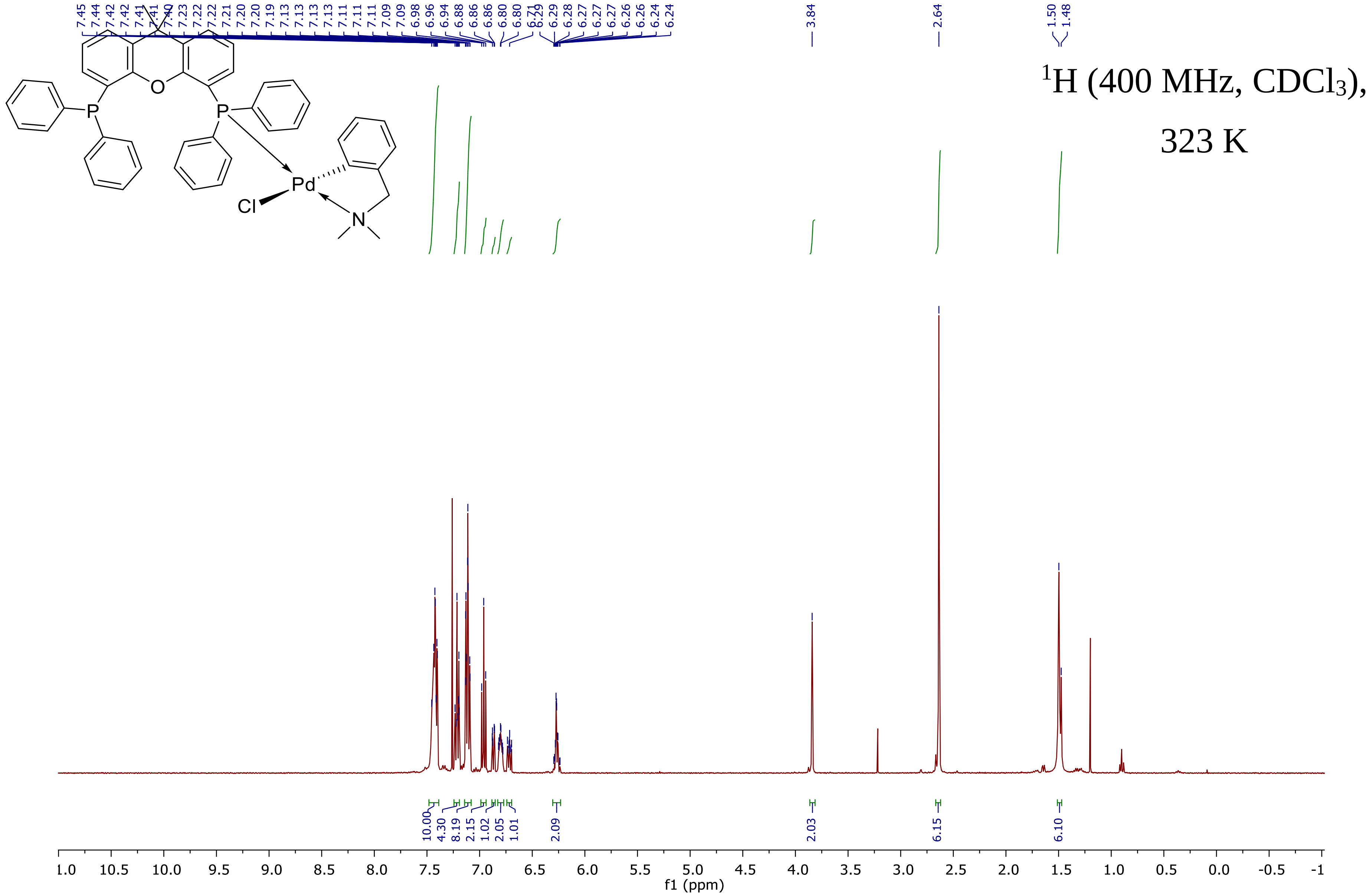

¹H (400 MHz, CDCl3),
323 K
f1 (ppm)

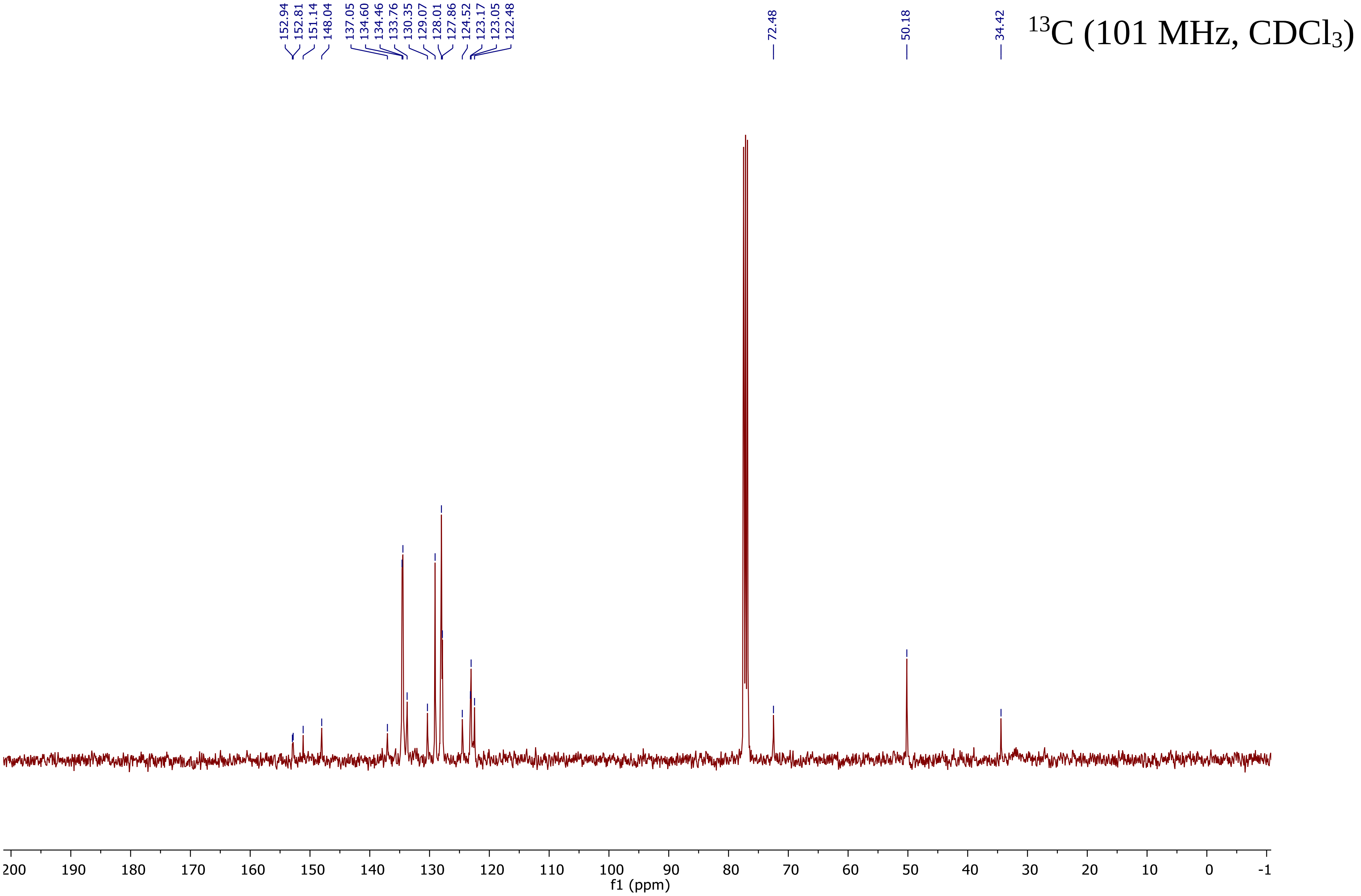

152.94
152.81
151.14
148.04
137.05
134.60
134.46
133.76
130.35
129.07
128.01
127.86
124.52
123.17
123.05
122.48
72.48
50.18
34.42
^{13}C (101 MHz, $CDCl_3$)
200 190 180 170 160 150 140 130 120 110 100 90 80 70 60 50 40 30 20 10 0 -1
f1 (ppm)

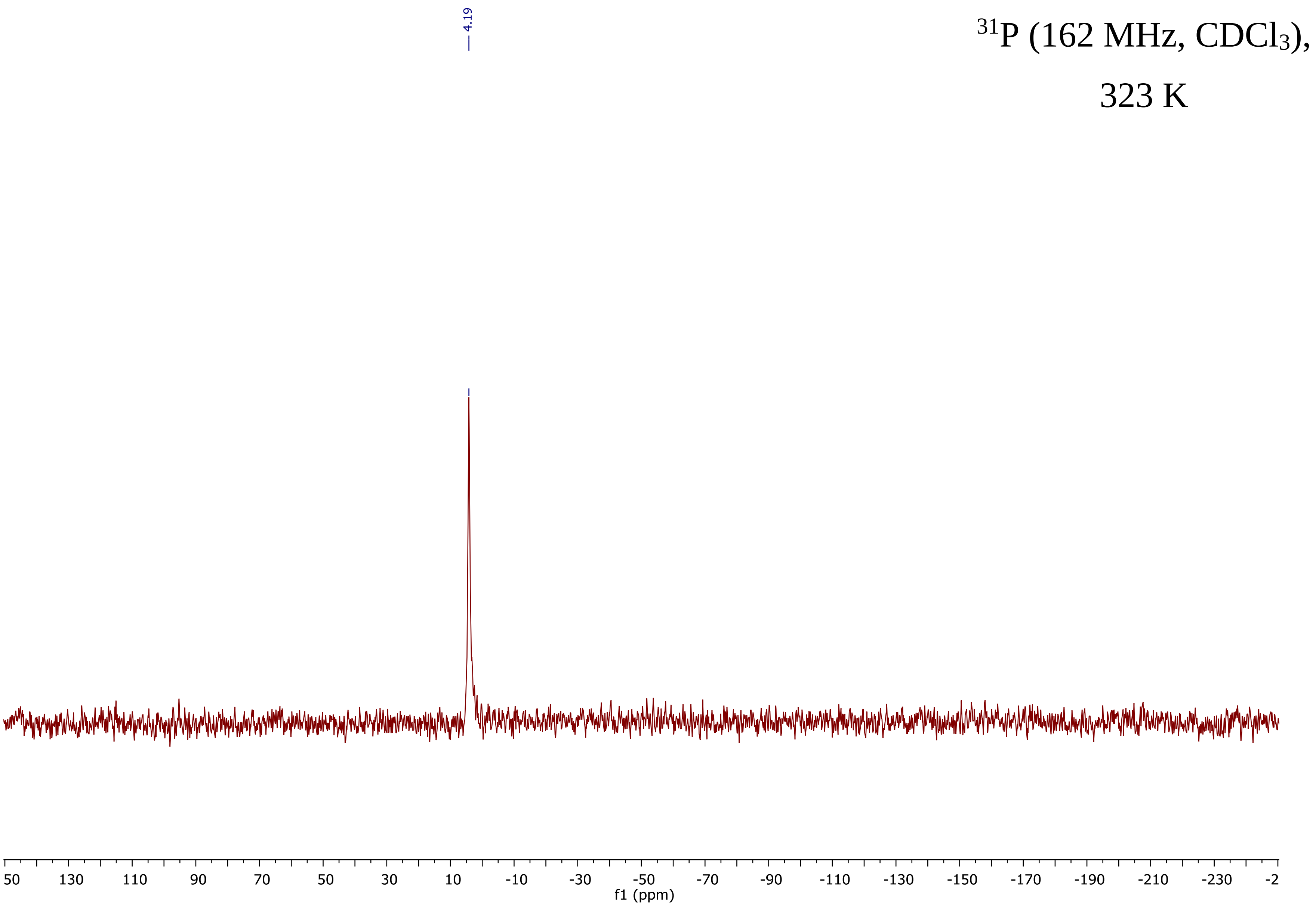

4.19
^{31}P (162 MHz, $CDCl_3$),
323 K
50
130
110
90
70
50
30
10
-10
-30
-50
-70
-90
-110
-130
-150
-170
-190
-210
-230
-2
f1 (ppm)

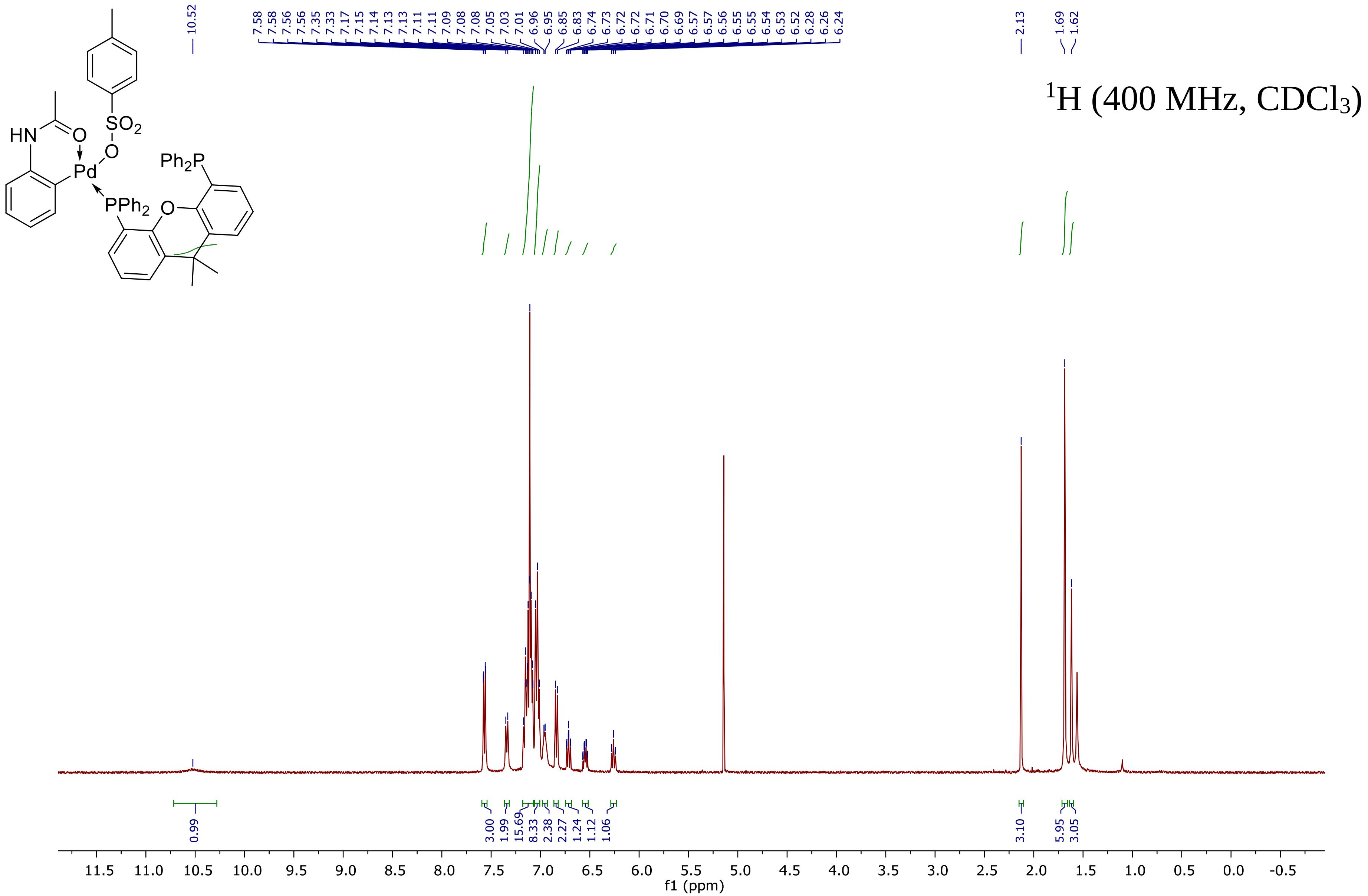

HN
O
SO2
O
Pd
Ph2P
PPh2
O
10.52
7.58
7.58
7.56
7.35
7.33
7.17
7.15
7.14
7.13
7.13
7.11
7.11
7.09
7.08
7.08
7.05
7.03
7.01
6.96
6.95
6.85
6.83
6.74
6.73
6.72
6.72
6.71
6.70
6.69
6.57
6.57
6.56
6.55
6.55
6.54
6.53
6.52
6.28
6.26
6.24
2.13
1.69
1.62
1H (400 MHz, CDCl3)
0.99
3.00
1.99
15.69
8.33
2.38
2.27
1.24
1.12
1.06
3.10
5.95
3.05
11.5 11.0 10.5 10.0 9.5 9.0 8.5 8.0 7.5 7.0 6.5 6.0 5.5 5.0 4.5 4.0 3.5 3.0 2.5 2.0 1.5 1.0 0.5 0.0 -0.5
f1 (ppm)

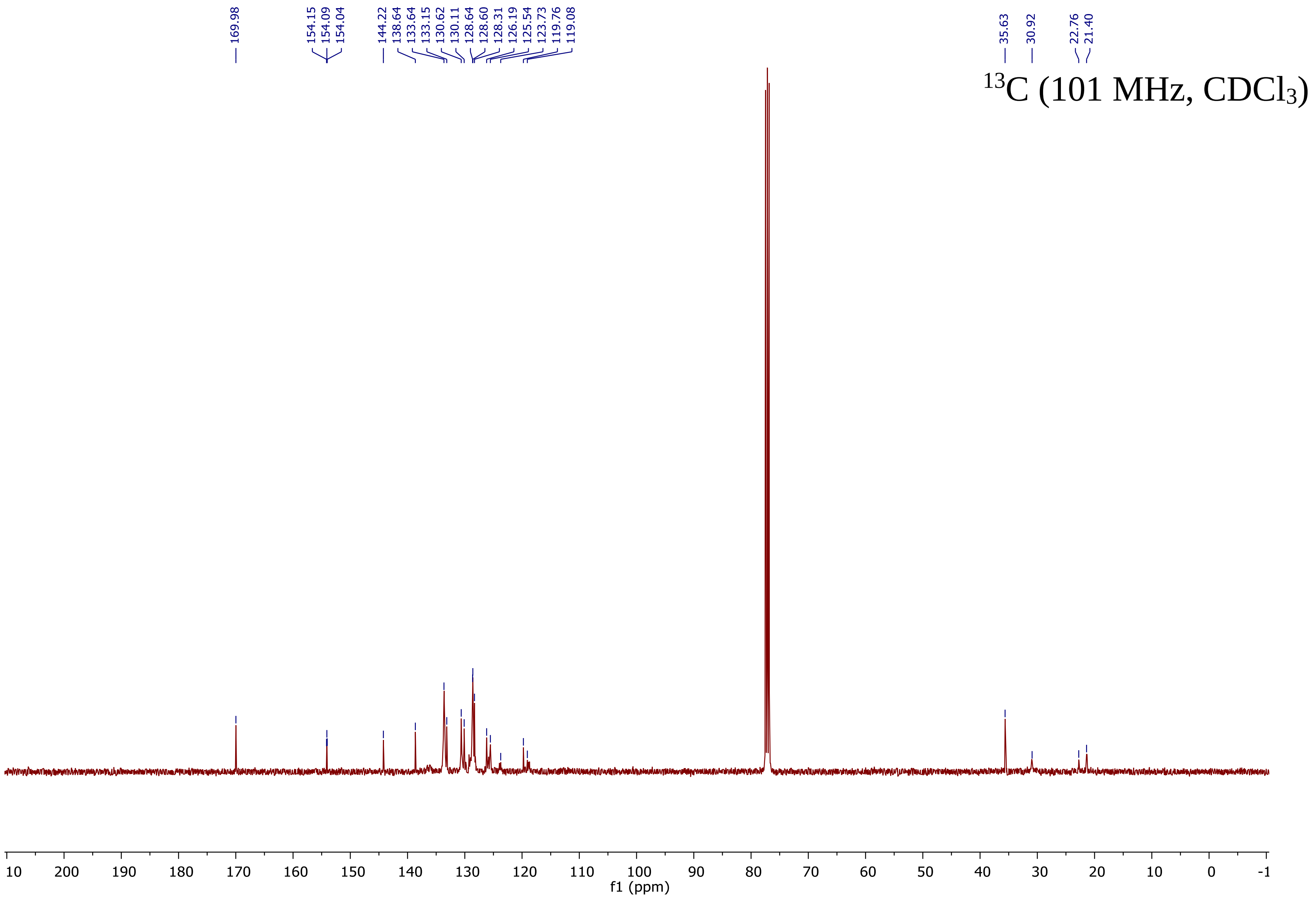
169.98
154.15
154.09
154.04
144.22
138.64
133.64
133.15
130.62
130.11
128.64
128.60
128.31
126.19
125.54
123.73
119.76
119.08
35.63
30.92
22.76
21.40
13C (101 MHz, CDCl3)
10 200 190 180 170 160 150 140 130 120 110 100 90 80 70 60 50 40 30 20 10 0 -1
f1 (ppm)

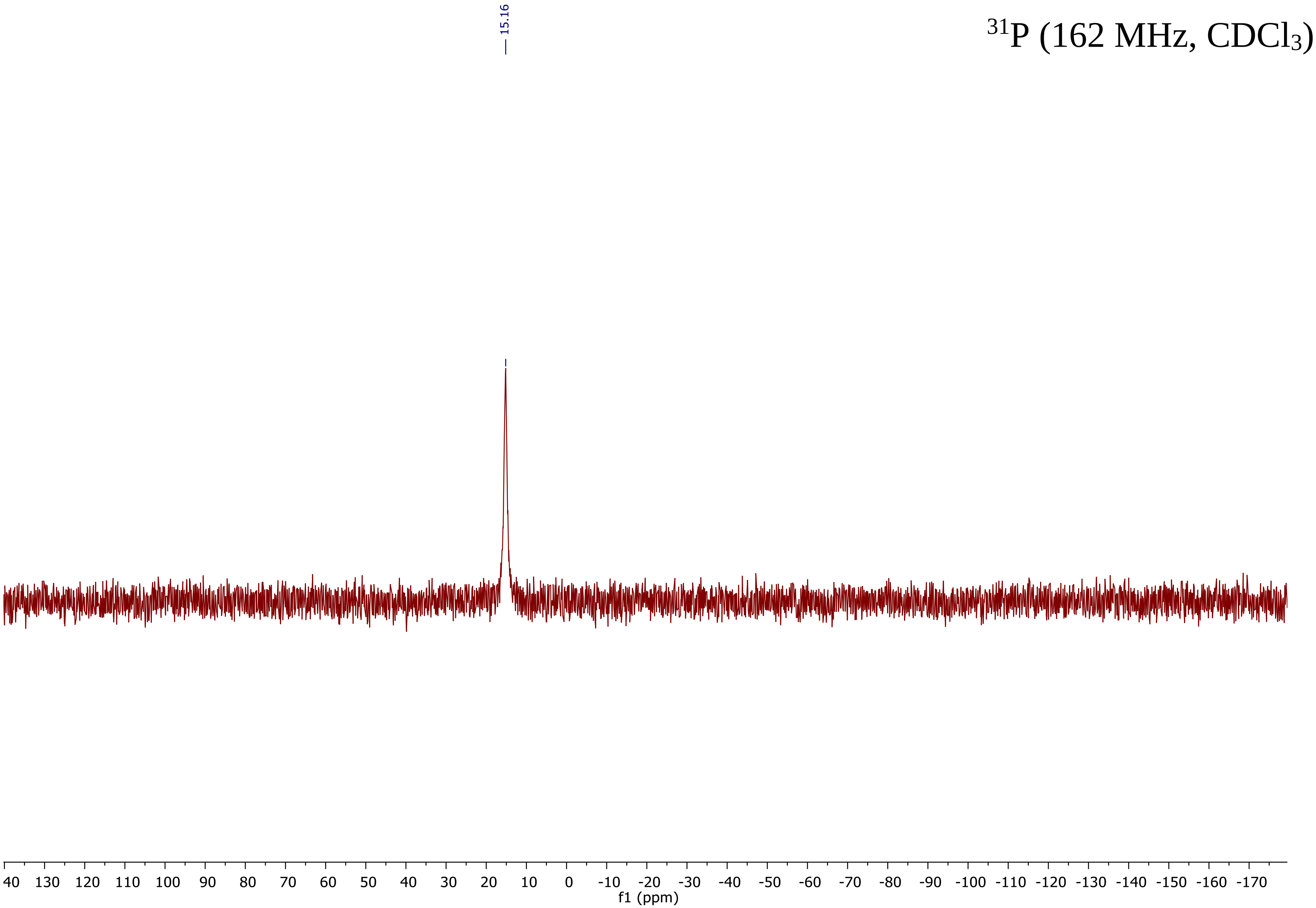

^{31}P (162 MHz, $CDCl_3$)
15.16
40 130 120 110 100 90 80 70 60 50 40 30 20 10 0 -10 -20 -30 -40 -50 -60 -70 -80 -90 -100 -110 -120 -130 -140 -150 -160 -170
f1 (ppm)

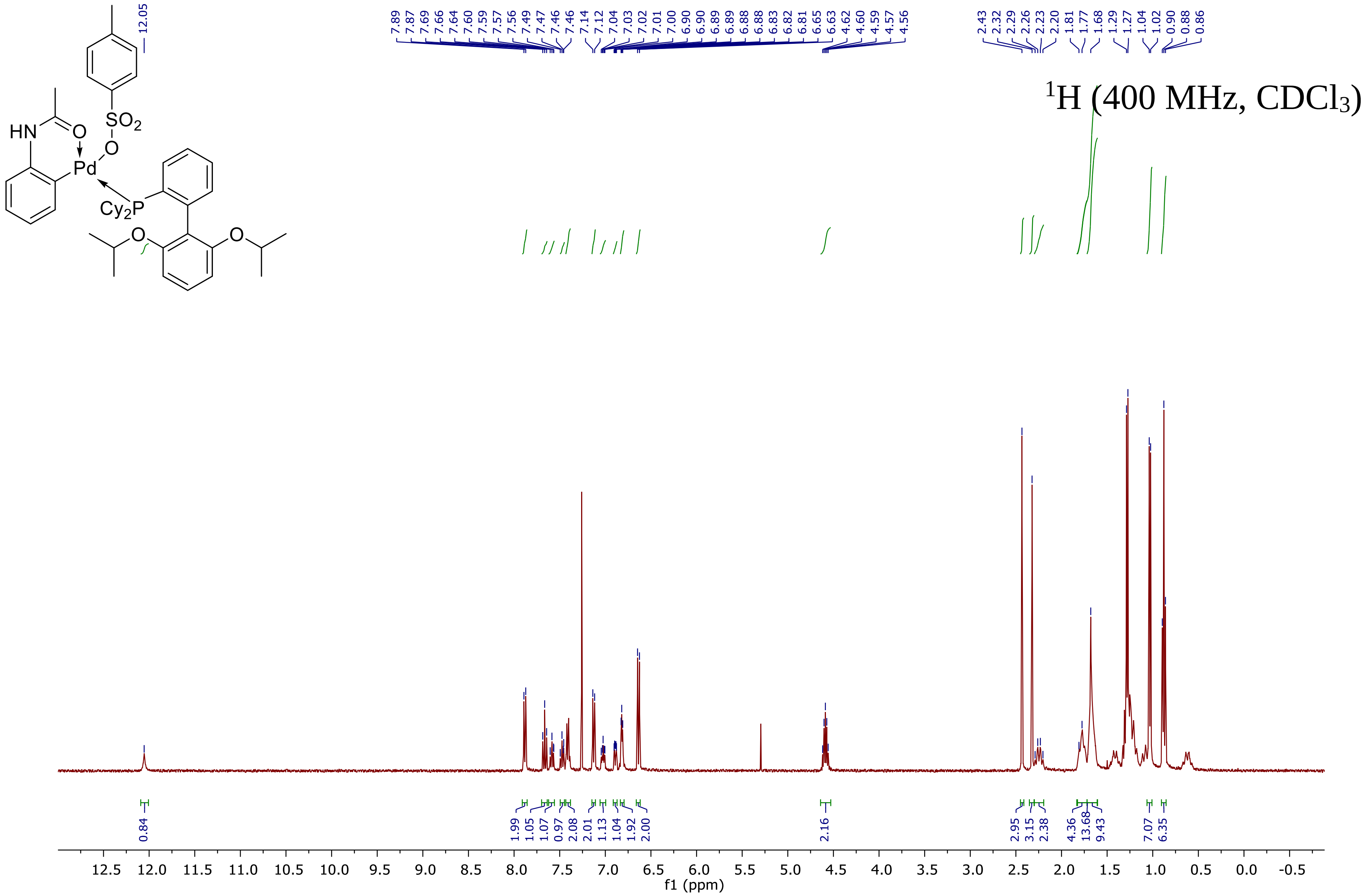
HN
O
Pd
SO2
O
Cy2P
O
O
12.05
7.89 7.87 7.69 7.66 7.64 7.60 7.59 7.57 7.56 7.49 7.47 7.46 7.46 7.14 7.12 7.04 7.03 7.02 7.01 7.00 6.90 6.90 6.89 6.88 6.88 6.83 6.82 6.81 6.65 6.63 4.62 4.60 4.59 4.57 4.56
2.43 2.32 2.29 2.26 2.23 2.20 1.81 1.77 1.68 1.29 1.27 1.04 1.02 0.90 0.88 0.86
1H (400 MHz, CDCl3)
0.84
1.99 1.05 1.07 0.97 2.08 2.01 1.13 1.04 1.92 2.00
2.16
2.95 3.15 2.38 4.36 13.68 9.43 7.07 6.35
12.5 12.0 11.5 11.0 10.5 10.0 9.5 9.0 8.5 8.0 7.5 7.0 6.5 6.0 5.5 5.0 4.5 4.0 3.5 3.0 2.5 2.0 1.5 1.0 0.5 0.0 -0.5
f1 (ppm)

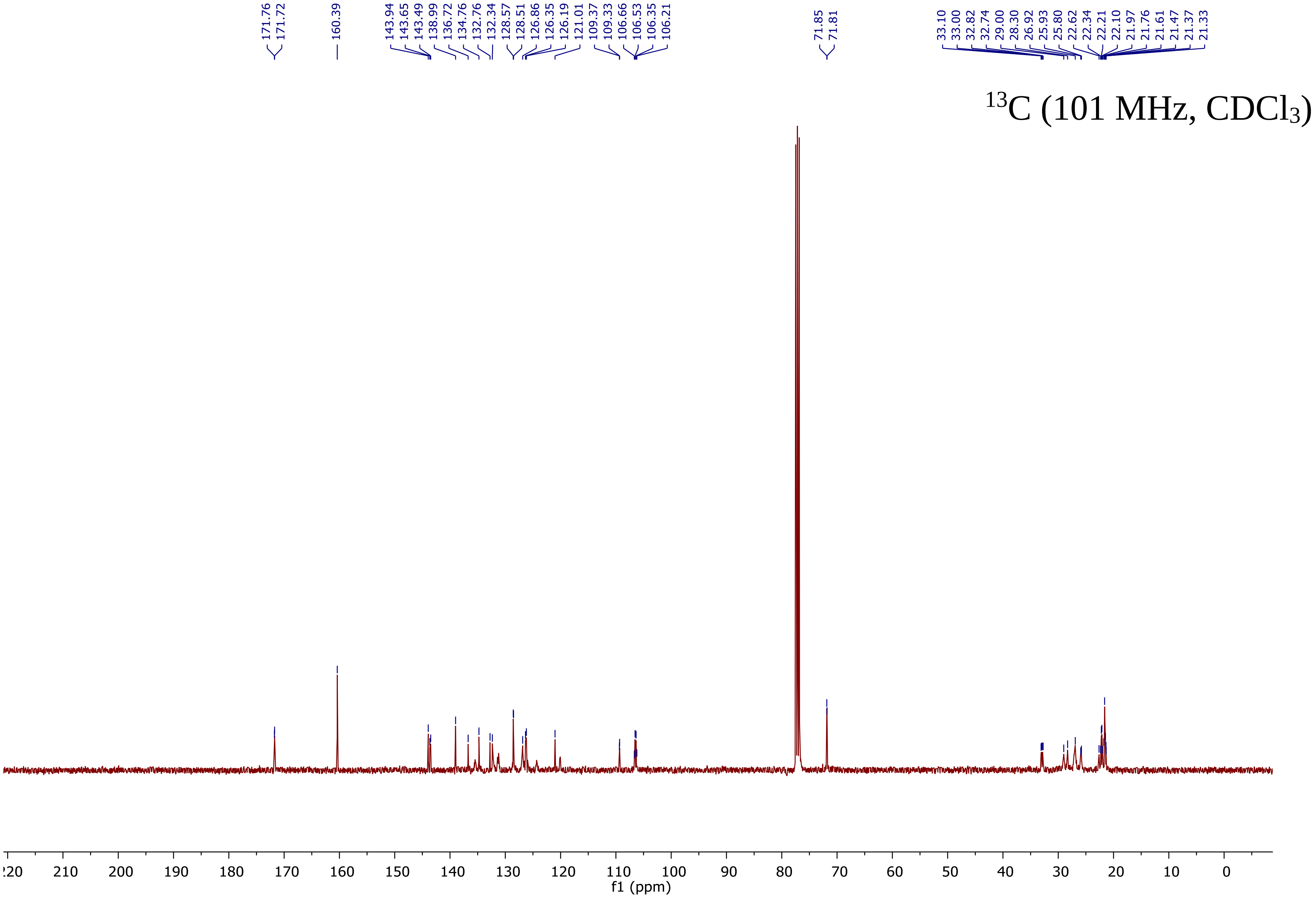
171.76
171.72
160.39
143.94
143.65
143.49
138.99
136.72
134.76
132.76
132.34
128.57
128.51
126.86
126.35
126.19
121.01
109.37
109.33
106.66
106.53
106.35
106.21
71.85
71.81
33.10
33.00
32.82
32.74
29.00
28.30
26.92
25.93
25.80
22.62
22.34
22.21
22.10
21.97
21.76
21.61
21.47
21.37
21.33
^{13}C (101 MHz, $CDCl_3$)
f1 (ppm)

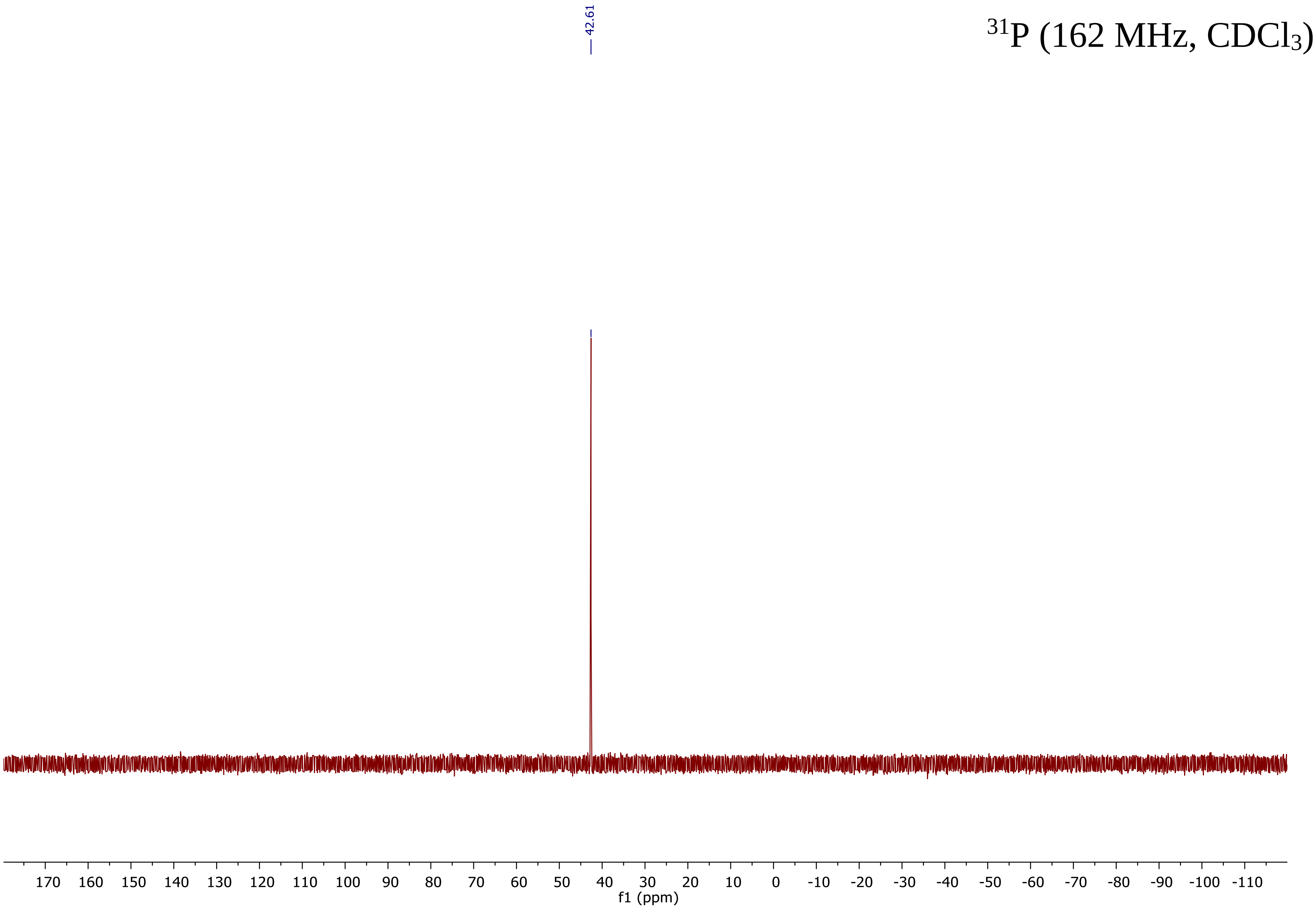

42.61
^{31}P (162 MHz, $CDCl_3$)
170 160 150 140 130 120 110 100 90 80 70 60 50 40 30 20 10 0 -10 -20 -30 -40 -50 -60 -70 -80 -90 -100 -110
f1 (ppm)

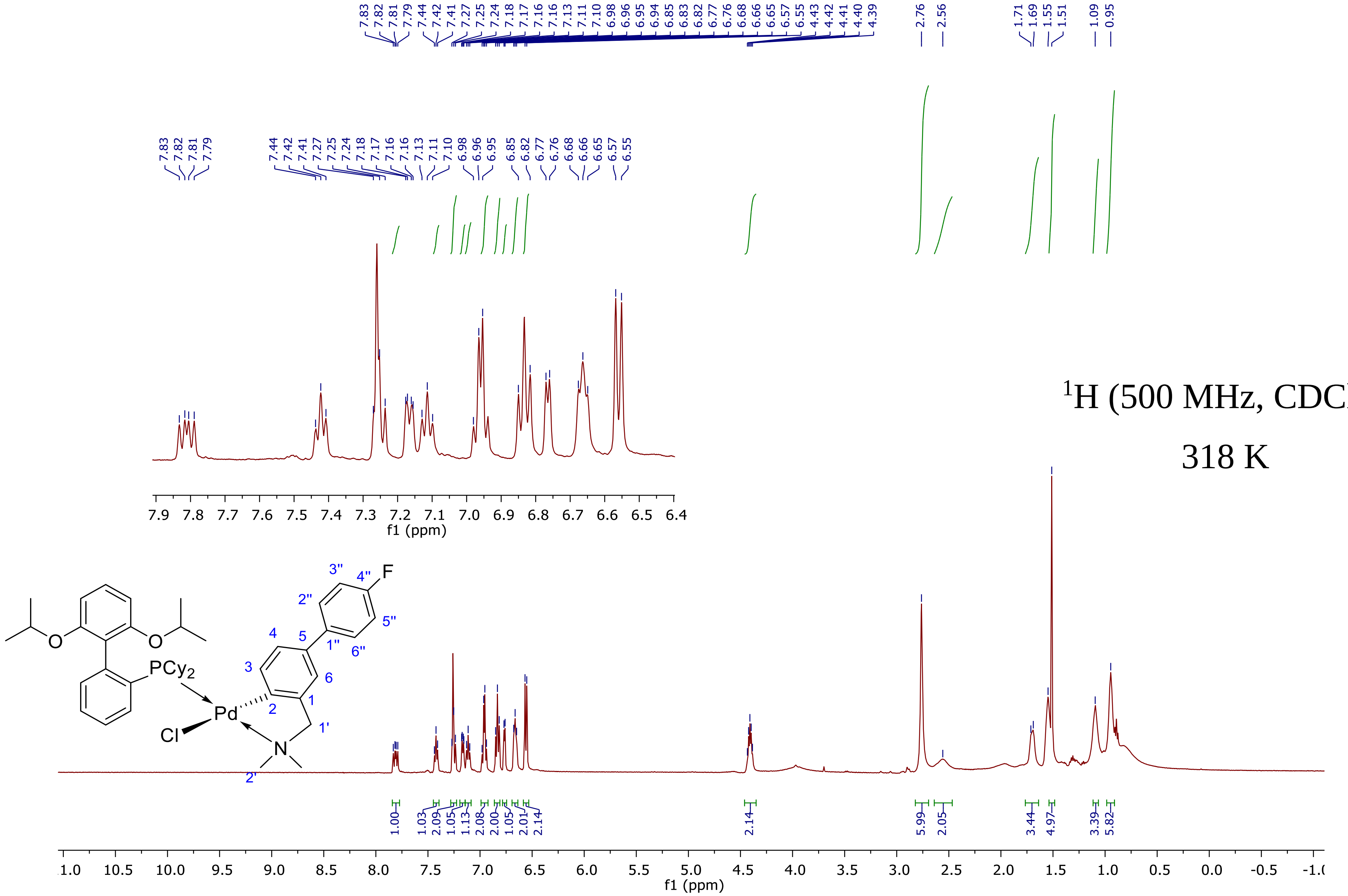

^{1}H (500 MHz, $CDCl_3$), 318 K

^{19}F (376 MHz, $CDCl_3$)

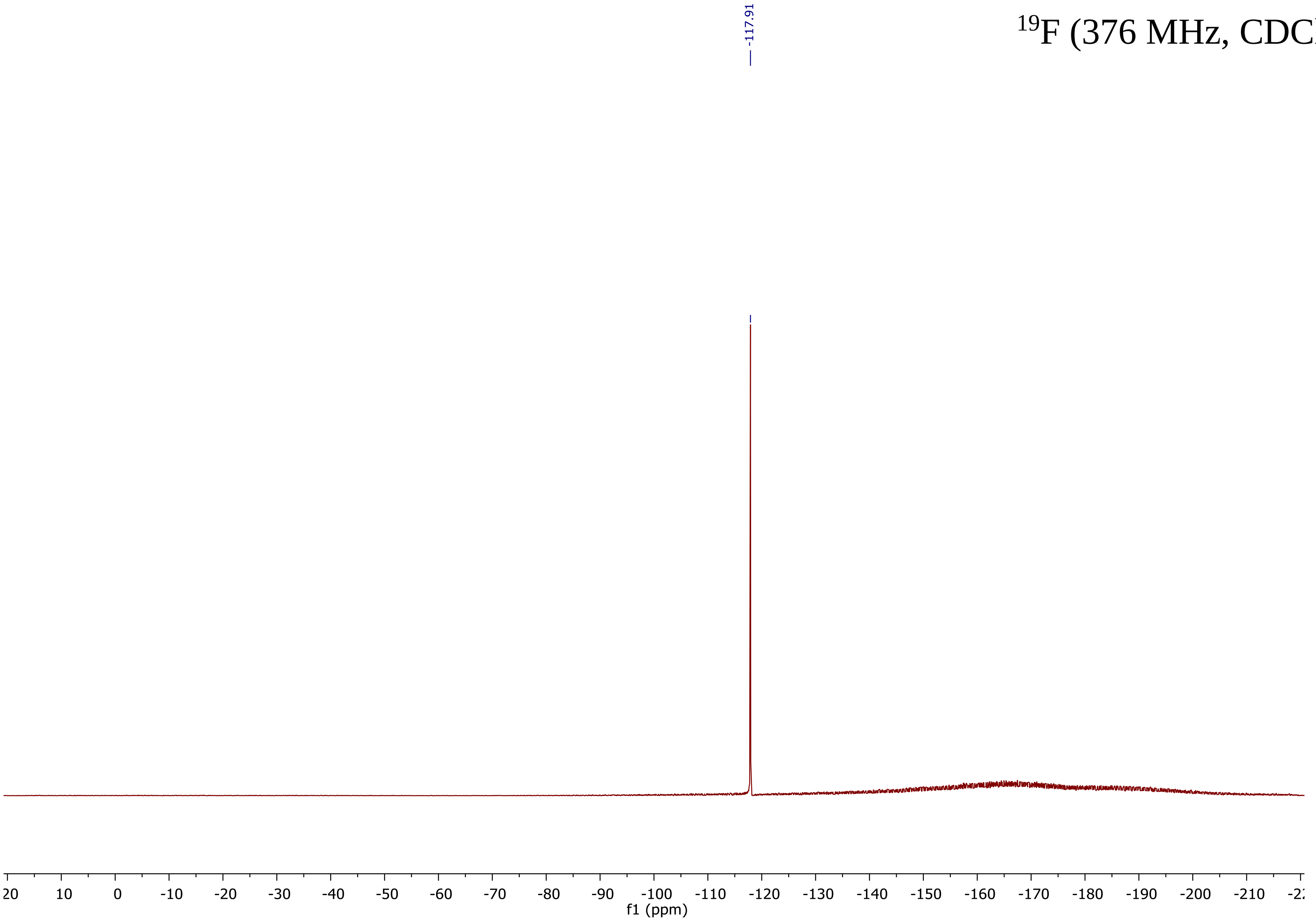

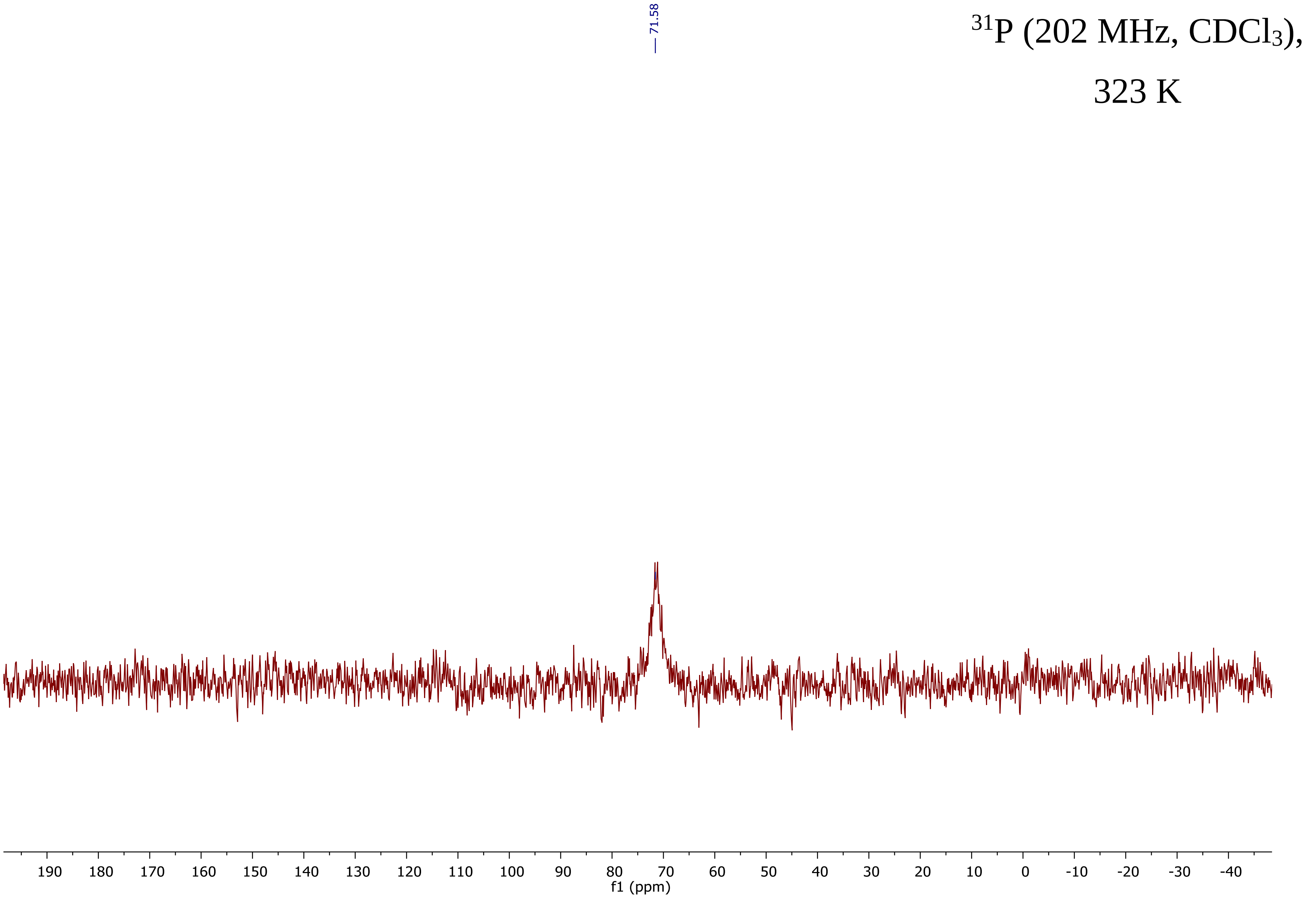

^{31}P (202 MHz, $CDCl_3$),
323 K
71.58
190 180 170 160 150 140 130 120 110 100 90 80 70 60 50 40 30 20 10 0 -10 -20 -30 -40
f1 (ppm)

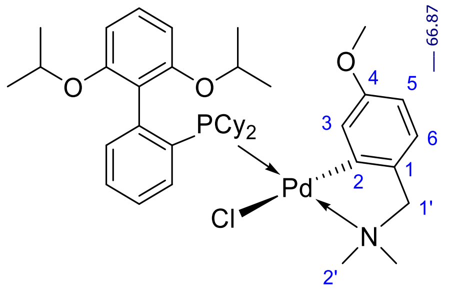

^{31}P (162 MHz, $CDCl_3$),

328 K

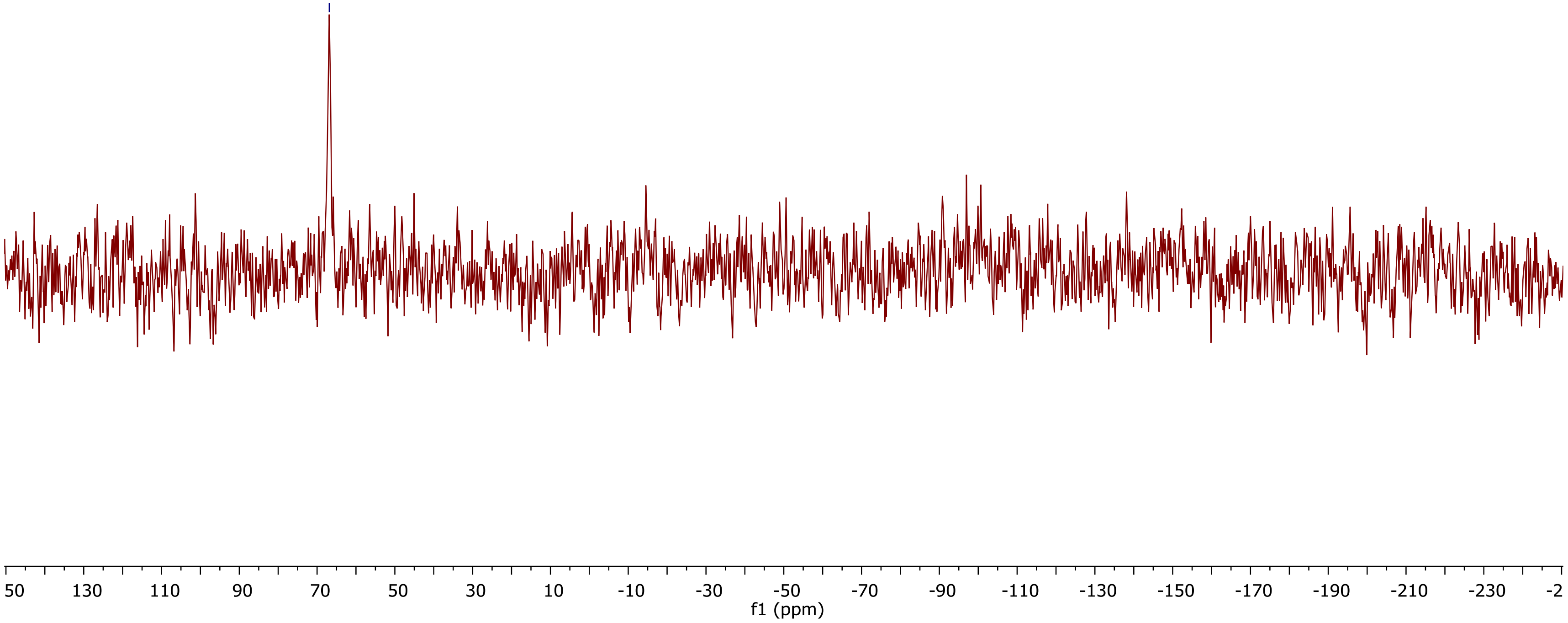

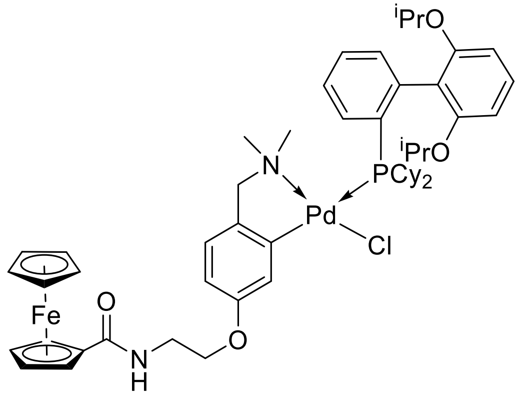

^{31}P (162 MHz, $CDCl_3$)

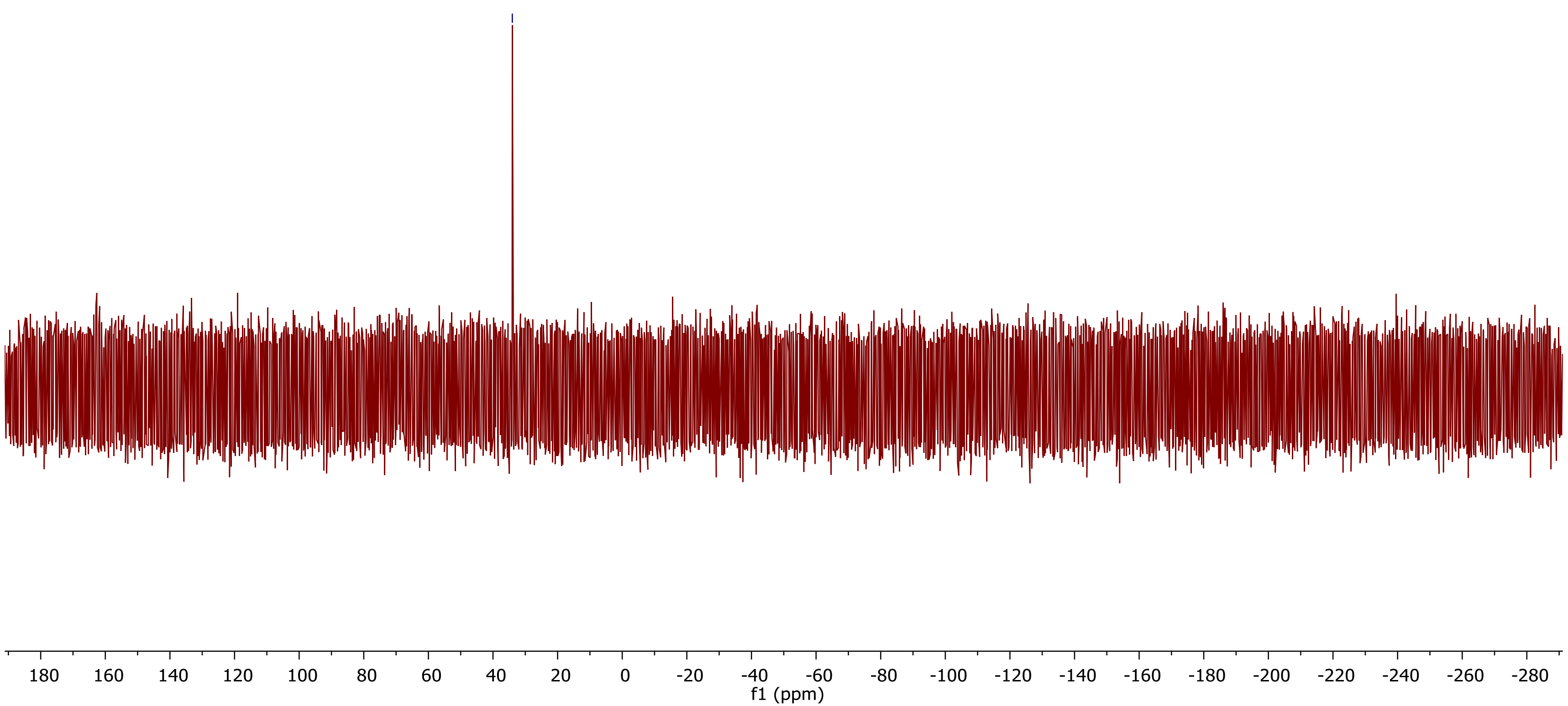

^{31}P (162 MHz, $CDCl_3$)

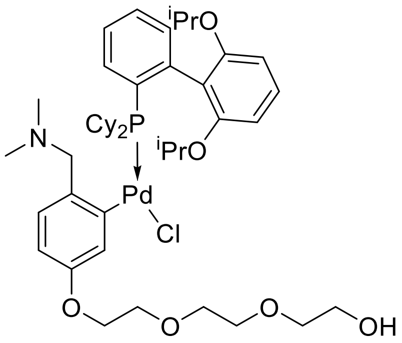

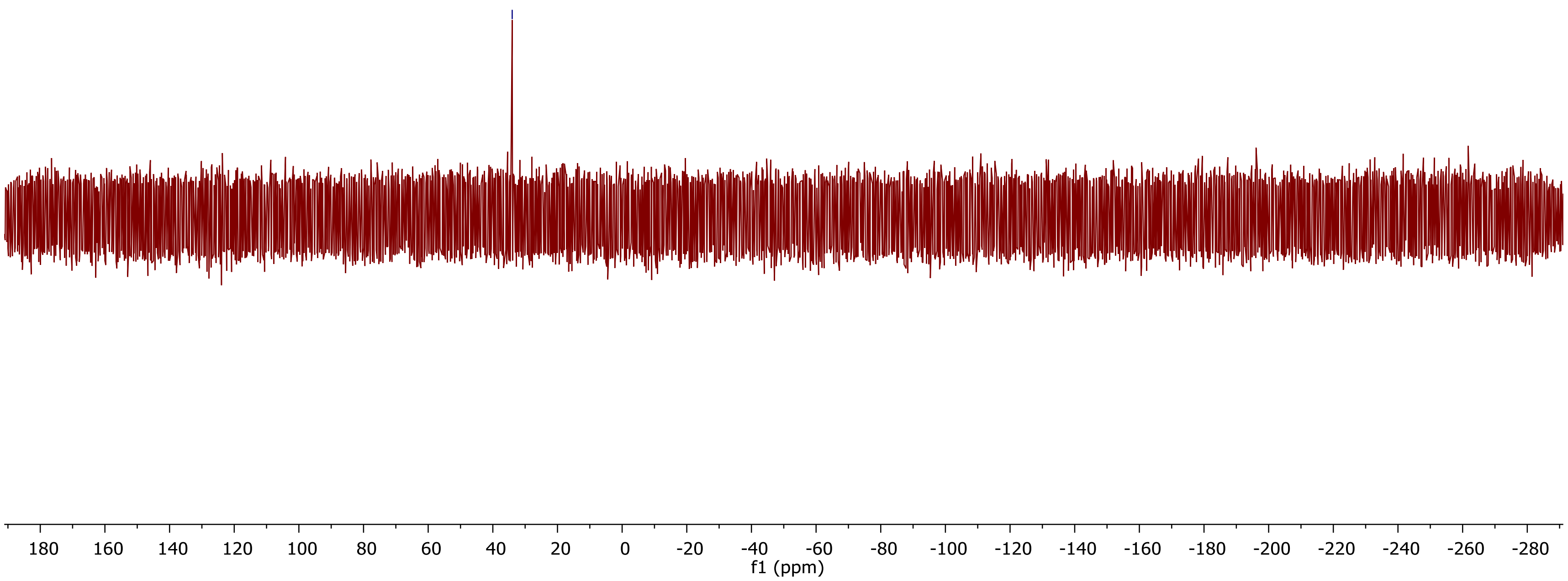

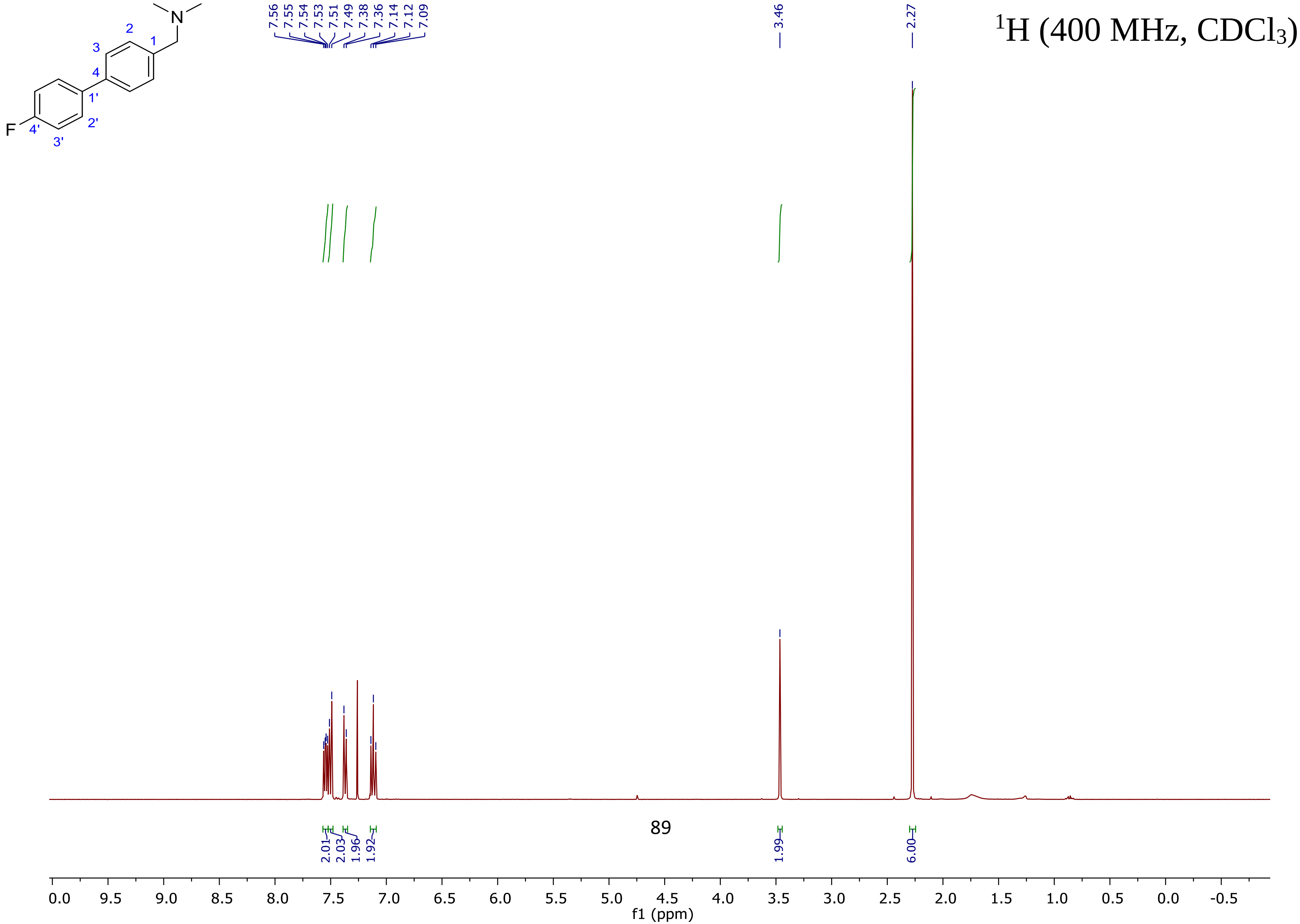
^{1}H (400 MHz, CDCl$_3$)
N
F
1
2
3
4
1'
2'
3'
4'
7.56
7.55
7.54
7.53
7.51
7.49
7.38
7.36
7.14
7.12
7.09
3.46
2.27
2.01
2.03
1.96
1.92
1.99
6.00
0.0 9.5 9.0 8.5 8.0 7.5 7.0 6.5 6.0 5.5 5.0 4.5 4.0 3.5 3.0 2.5 2.0 1.5 1.0 0.5 0.0 -0.5
f1 (ppm)

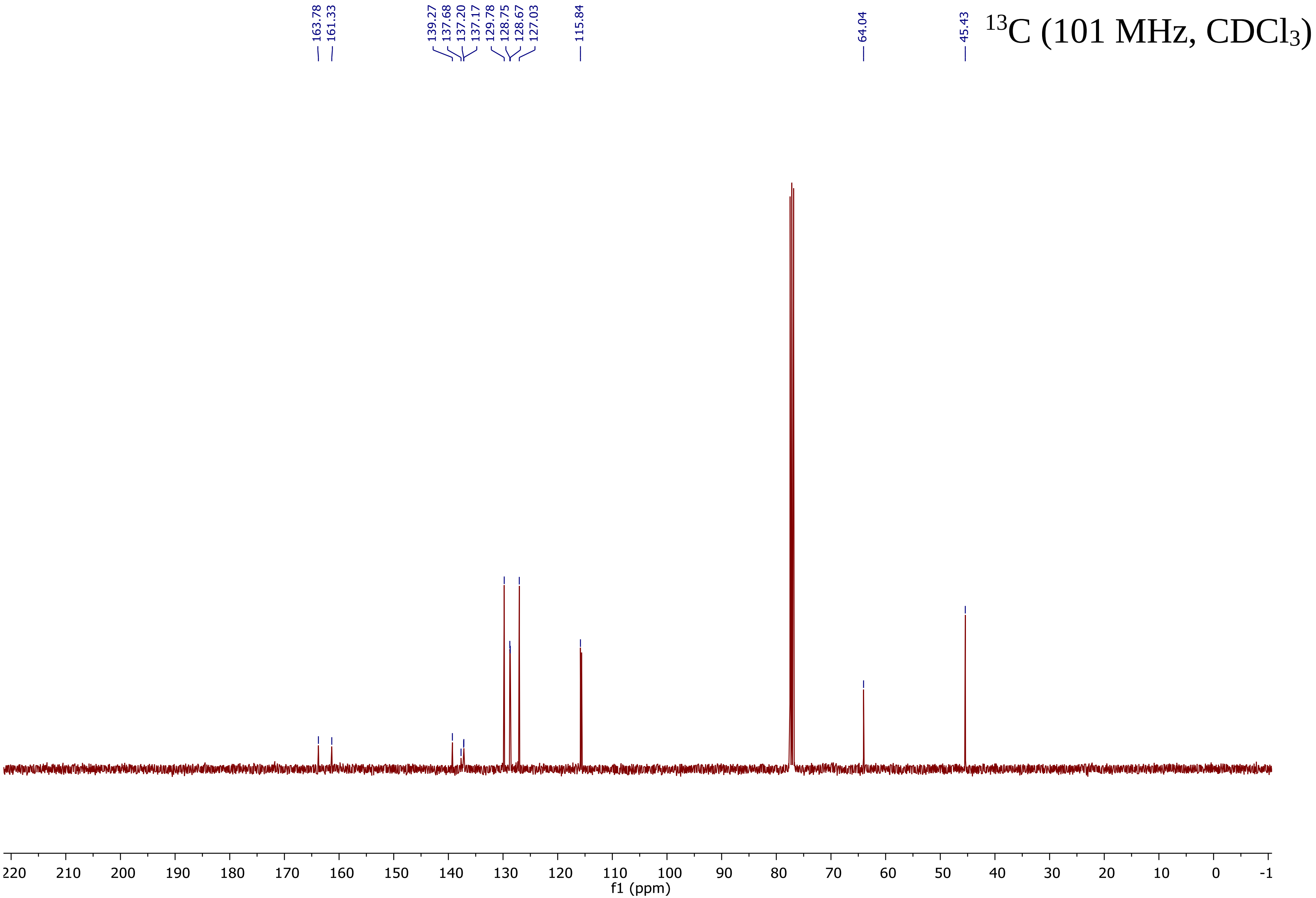
163.78
161.33
139.27
137.68
137.20
137.17
129.78
128.75
128.67
127.03
115.84
64.04
45.43
^{13}C (101 MHz, $CDCl_3$)
220
210
200
190
180
170
160
150
140
130
120
110
100
90
80
70
60
50
40
30
20
10
0
-1
f1 (ppm)

^{19}F (376 MHz, $CDCl_3$)

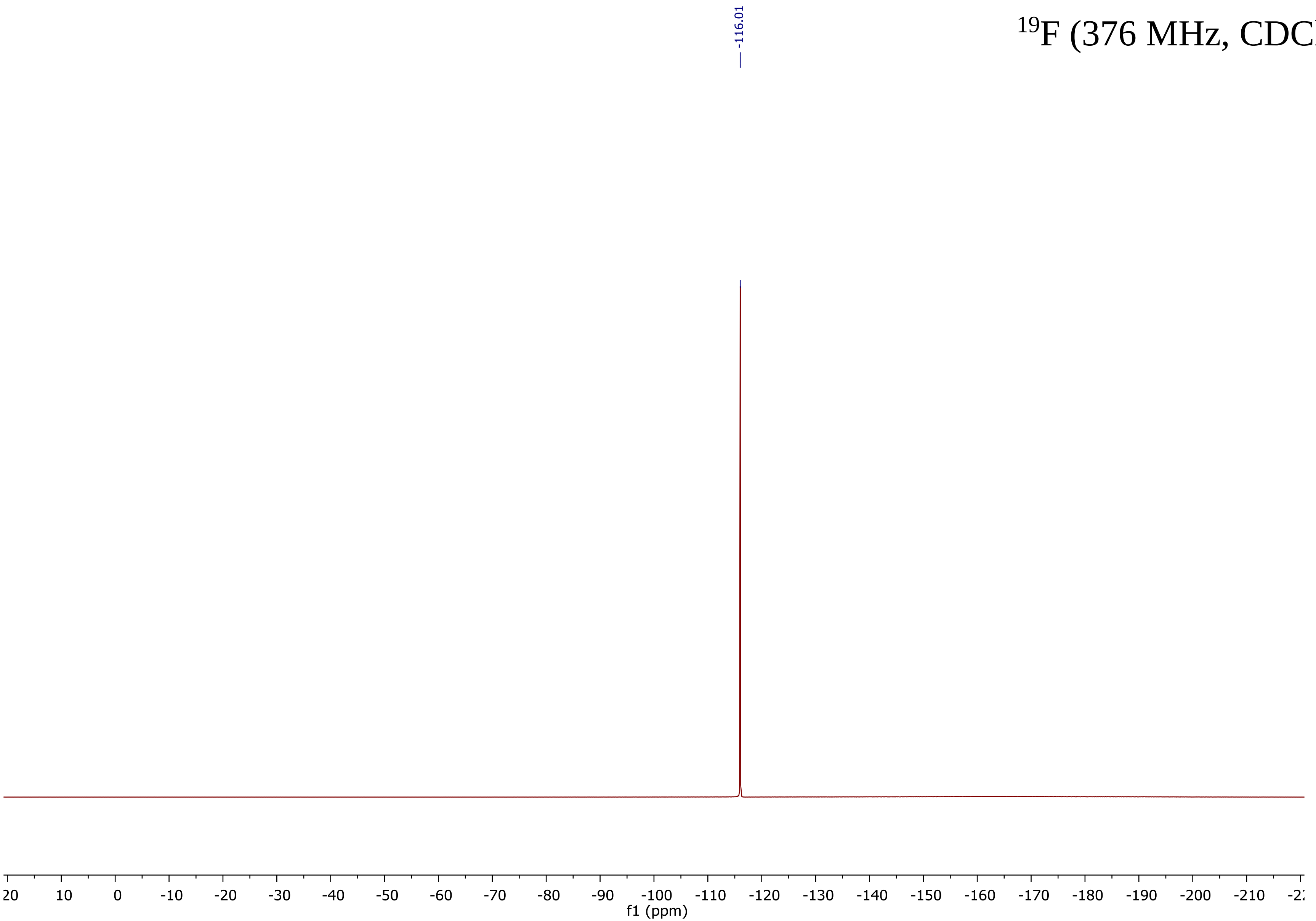

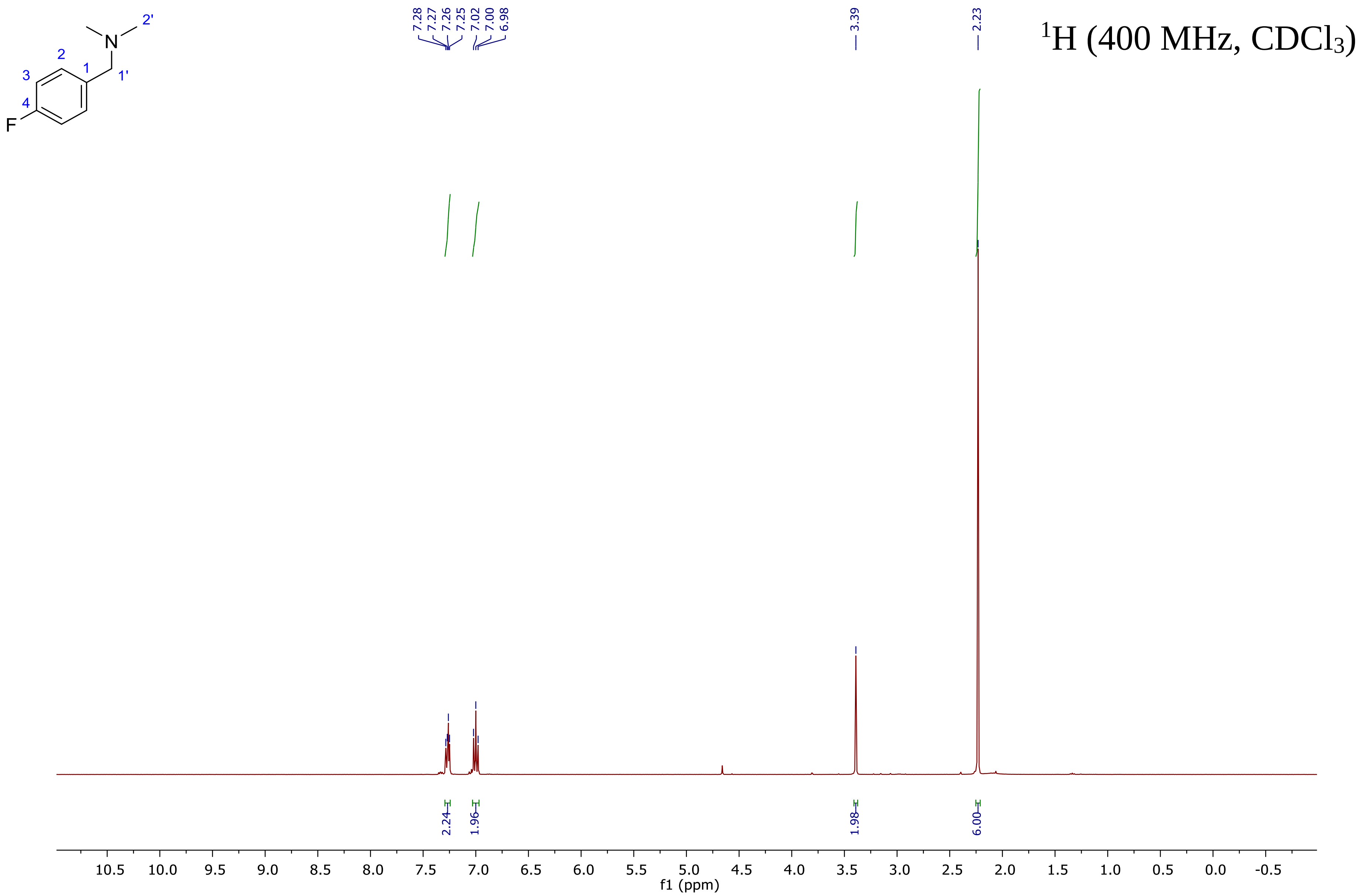

1H (400 MHz, CDCl3)
F
N
1
2
3
4
1'
2'
7.28
7.27
7.26
7.25
7.02
7.00
6.98
3.39
2.23
2.24
1.96
1.98
6.00
10.5 10.0 9.5 9.0 8.5 8.0 7.5 7.0 6.5 6.0 5.5 5.0 4.5 4.0 3.5 3.0 2.5 2.0 1.5 1.0 0.5 0.0 -0.5
f1 (ppm)

^{19}F (376 MHz, $CDCl_3$)

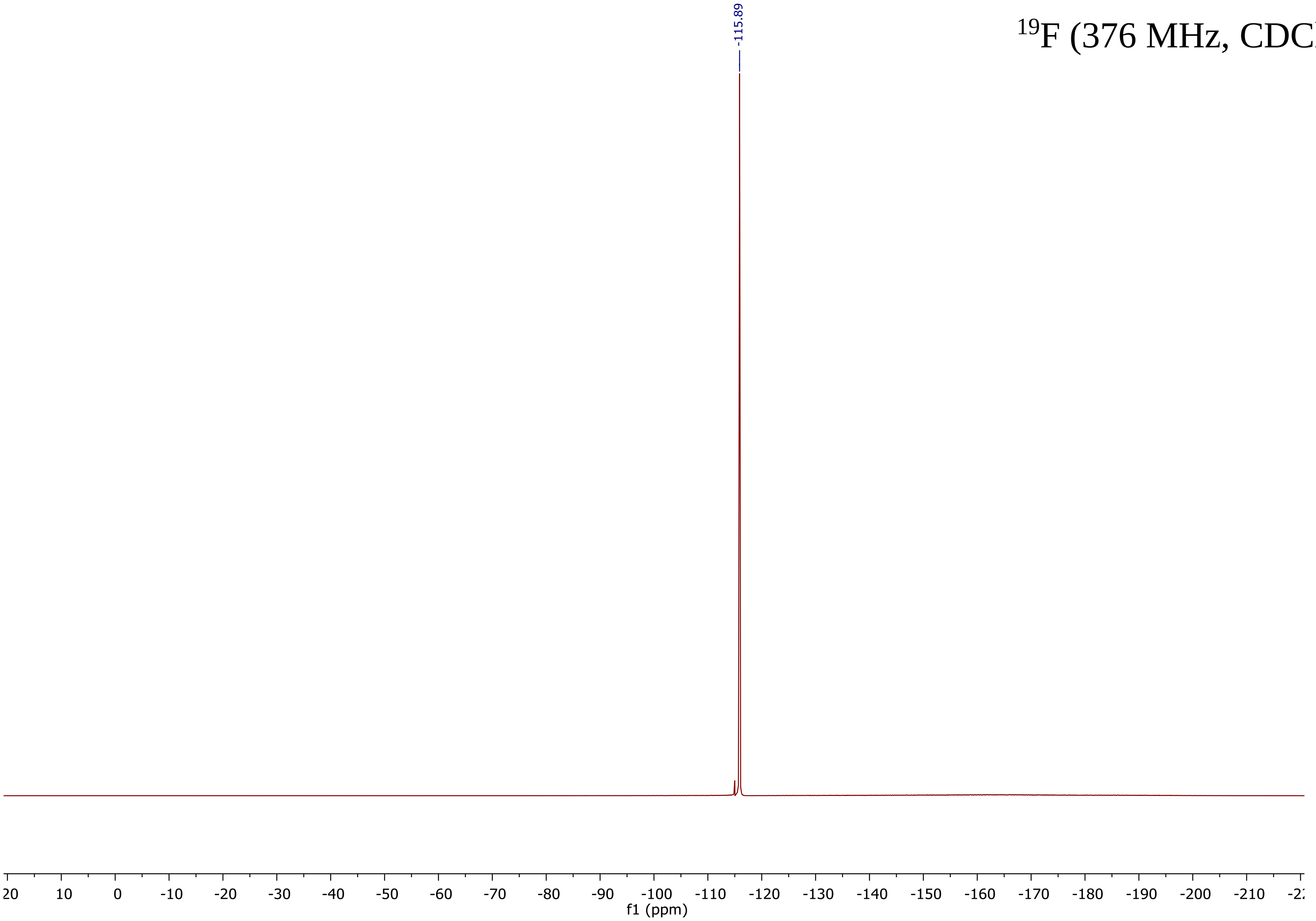

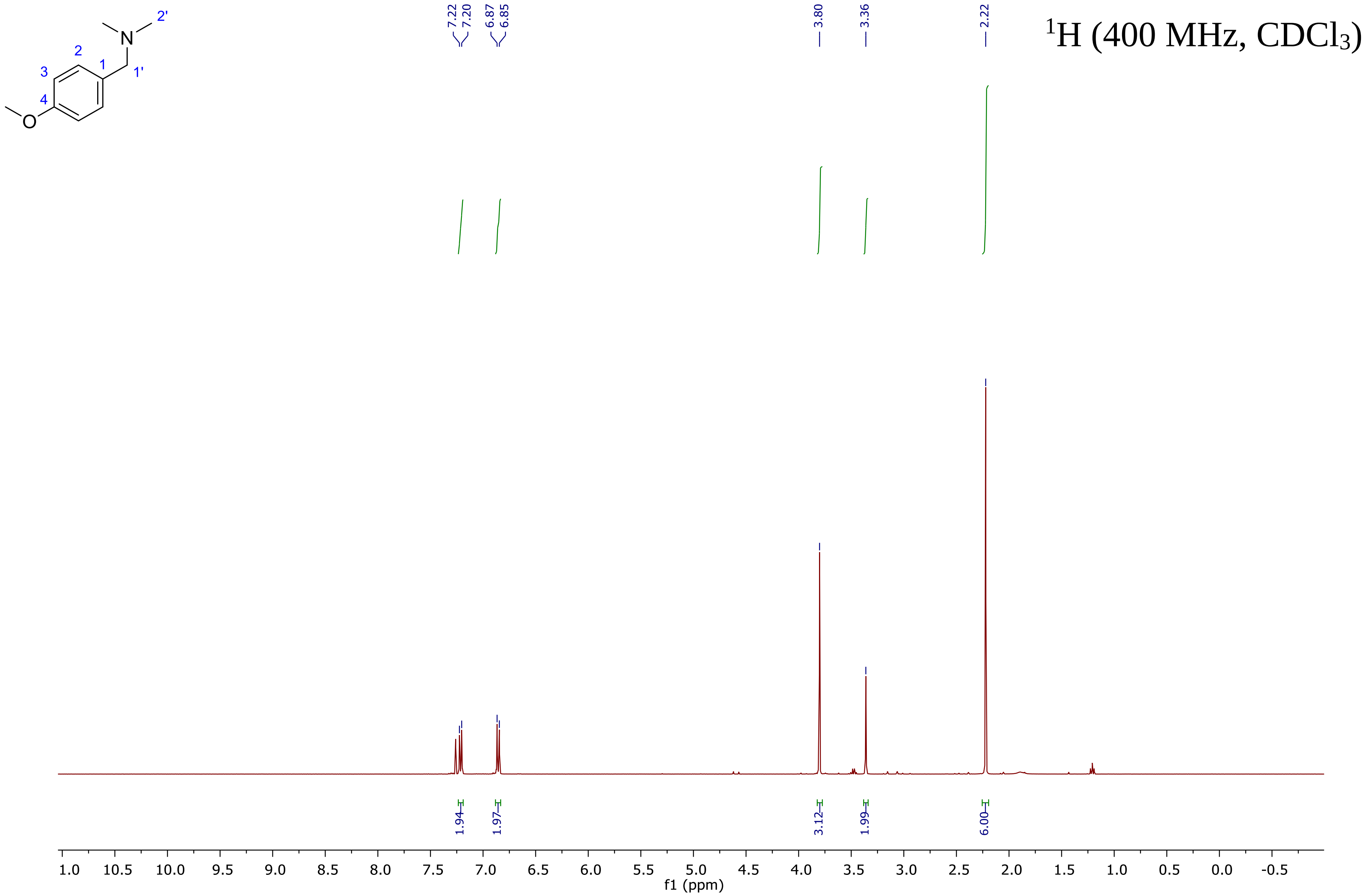

2'
2
1
3
1'
4
N
O
7.22
7.20
6.87
6.85
3.80
3.36
2.22
¹H (400 MHz, CDCl₃)
1.94
1.97
3.12
1.99
6.00
f1 (ppm)

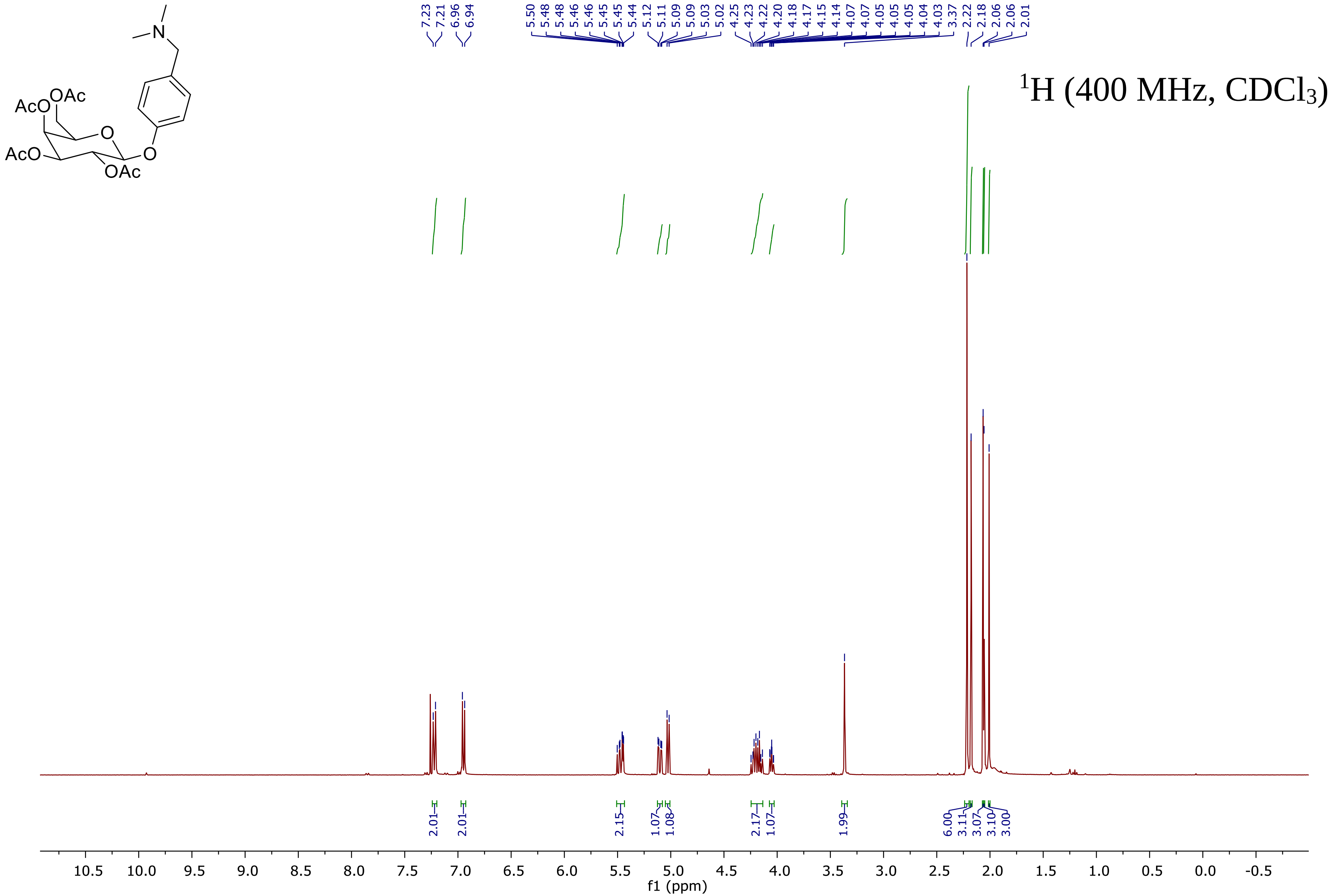
N
AcO
OAc
O
AcO
OAc
O
¹H (400 MHz, CDCl3)
7.23 7.21 6.96 6.94 5.50 5.48 5.48 5.46 5.46 5.45 5.45 5.44 5.12 5.11 5.09 5.09 5.03 5.02 4.25 4.23 4.22 4.20 4.18 4.17 4.15 4.14 4.07 4.07 4.05 4.05 4.04 4.03 3.37 2.22 2.18 2.06 2.06 2.01
2.01 2.01 2.15 1.07 1.08 2.17 1.07 1.99 6.00 3.11 3.07 3.10 3.00
10.5 10.0 9.5 9.0 8.5 8.0 7.5 7.0 6.5 6.0 5.5 5.0 4.5 4.0 3.5 3.0 2.5 2.0 1.5 1.0 0.5 0.0 -0.5
f1 (ppm)

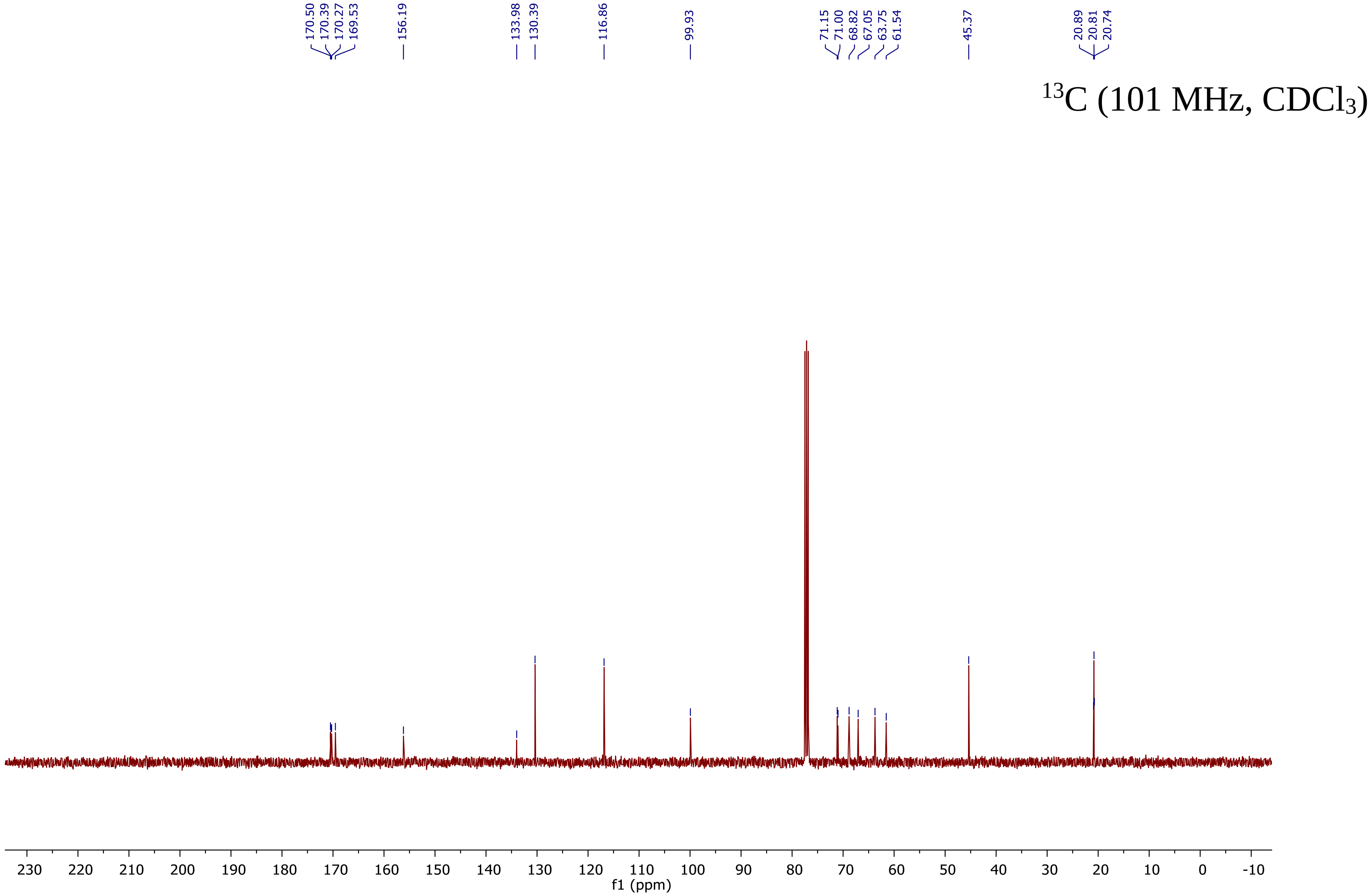
170.50
170.39
170.27
169.53
156.19
133.98
130.39
116.86
99.93
71.15
71.00
68.82
67.05
63.75
61.54
45.37
20.89
20.81
20.74
^{13}C (101 MHz, $CDCl_3$)
230 220 210 200 190 180 170 160 150 140 130 120 110 100 90 80 70 60 50 40 30 20 10 0 -10
f1 (ppm)

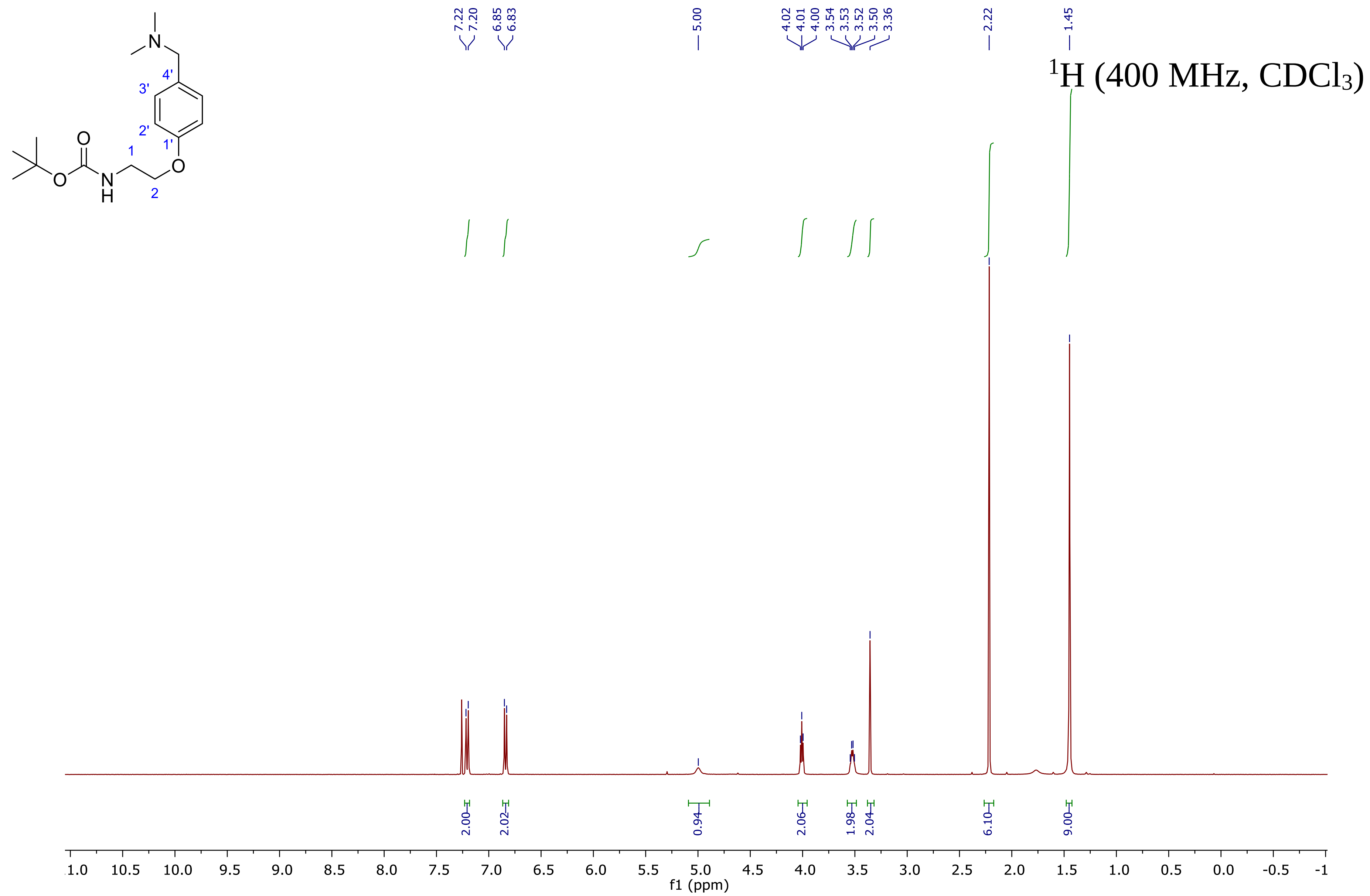

1H (400 MHz, CDCl3)
7.22
7.20
6.85
6.83
5.00
4.02
4.01
4.00
3.54
3.53
3.52
3.50
3.36
2.22
1.45
2.00
2.02
0.94
2.06
1.98
2.04
6.10
9.00
f1 (ppm)
O
N
H
1
2
1'
2'
3'
4'

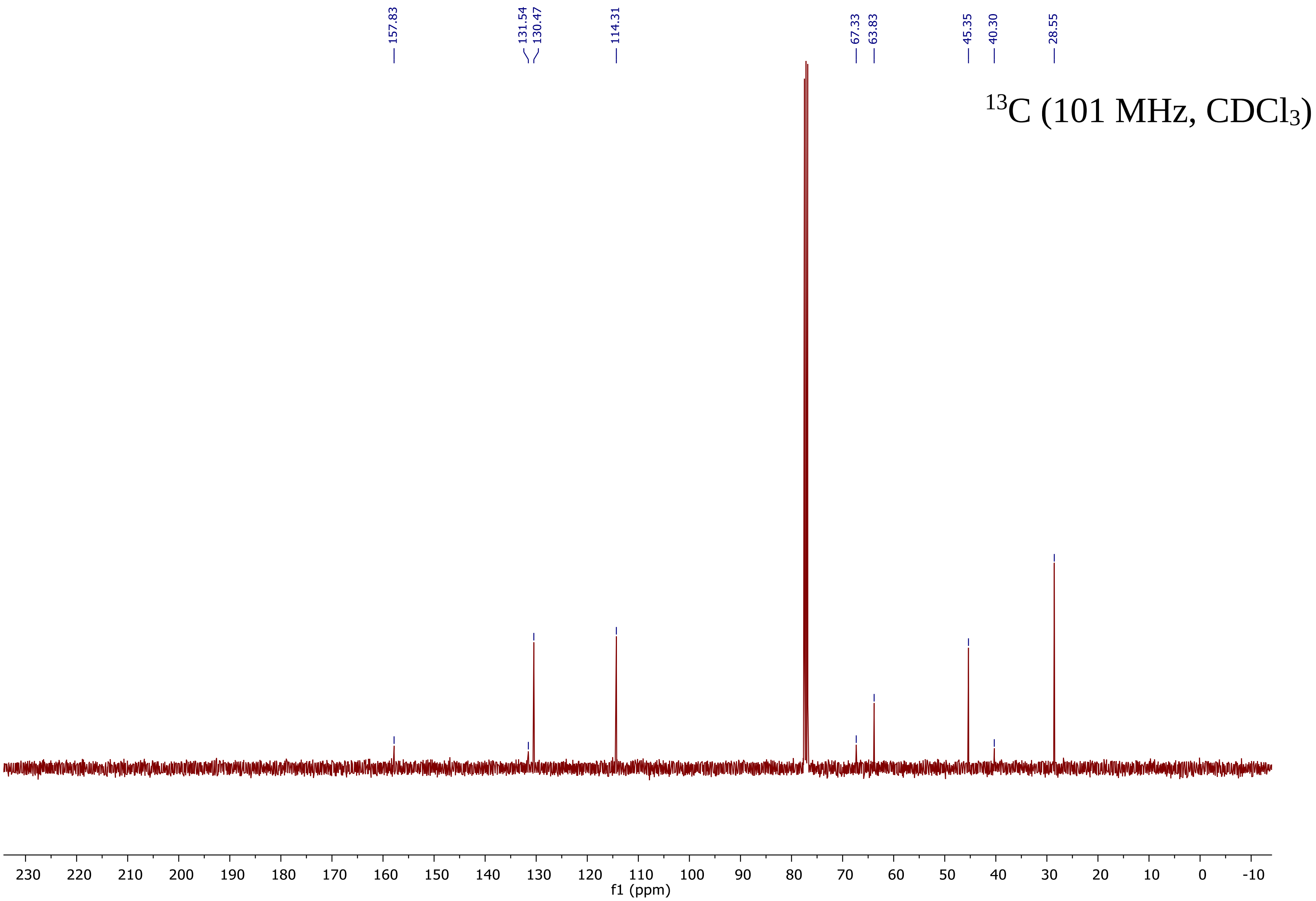
157.83
131.54
130.47
114.31
67.33
63.83
45.35
40.30
28.55
^{13}C (101 MHz, $CDCl_3$)
230
220
210
200
190
180
170
160
150
140
130
120
110
100
90
80
70
60
50
40
30
20
10
0
-10
f1 (ppm)

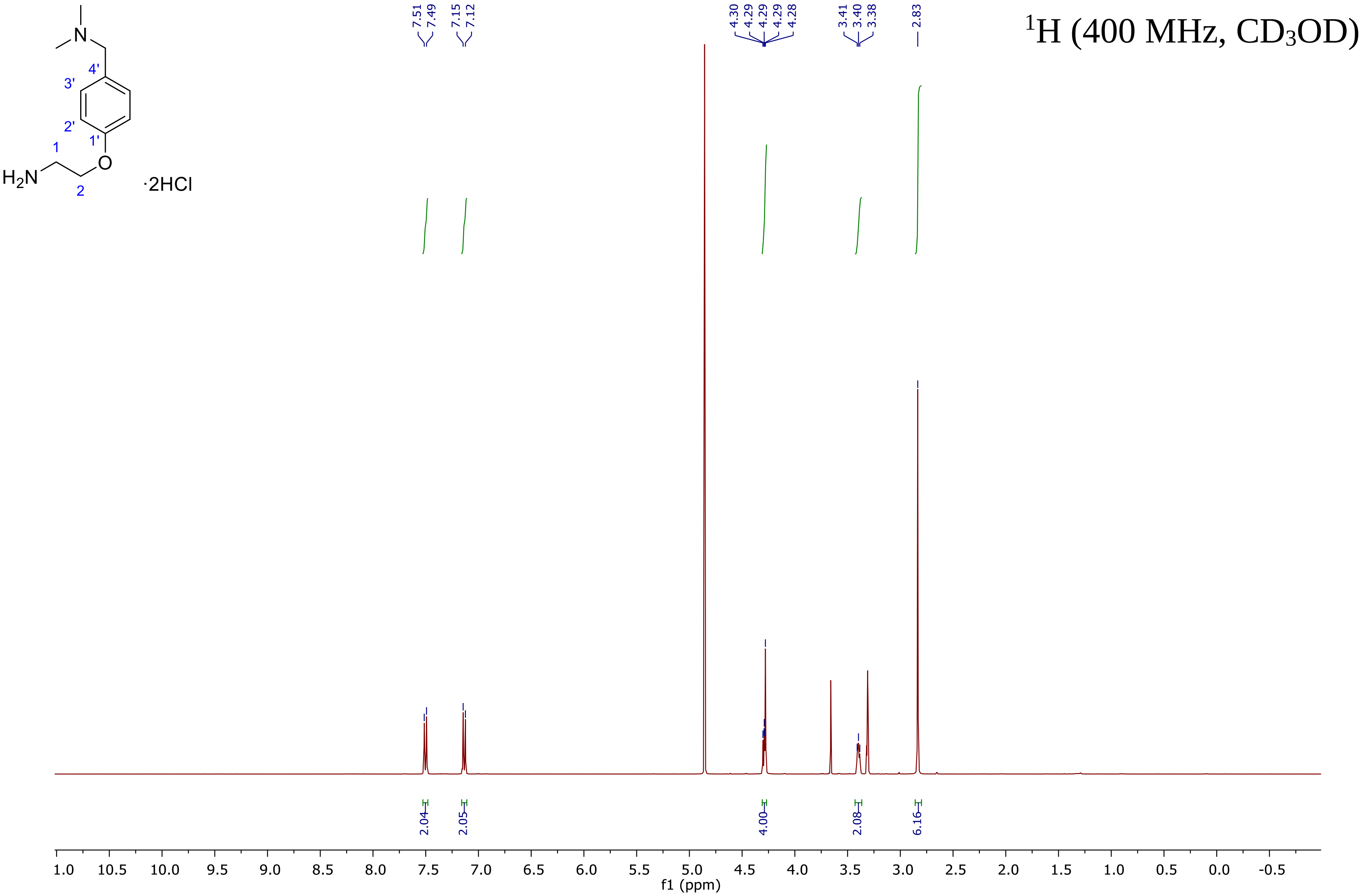
H2N
·2HCl
1
2
1'
2'
3'
4'
7.51
7.49
7.15
7.12
4.30
4.29
4.29
4.29
4.28
3.41
3.40
3.38
2.83
1H (400 MHz, CD3OD)
2.04
2.05
4.00
2.08
6.16
f1 (ppm)

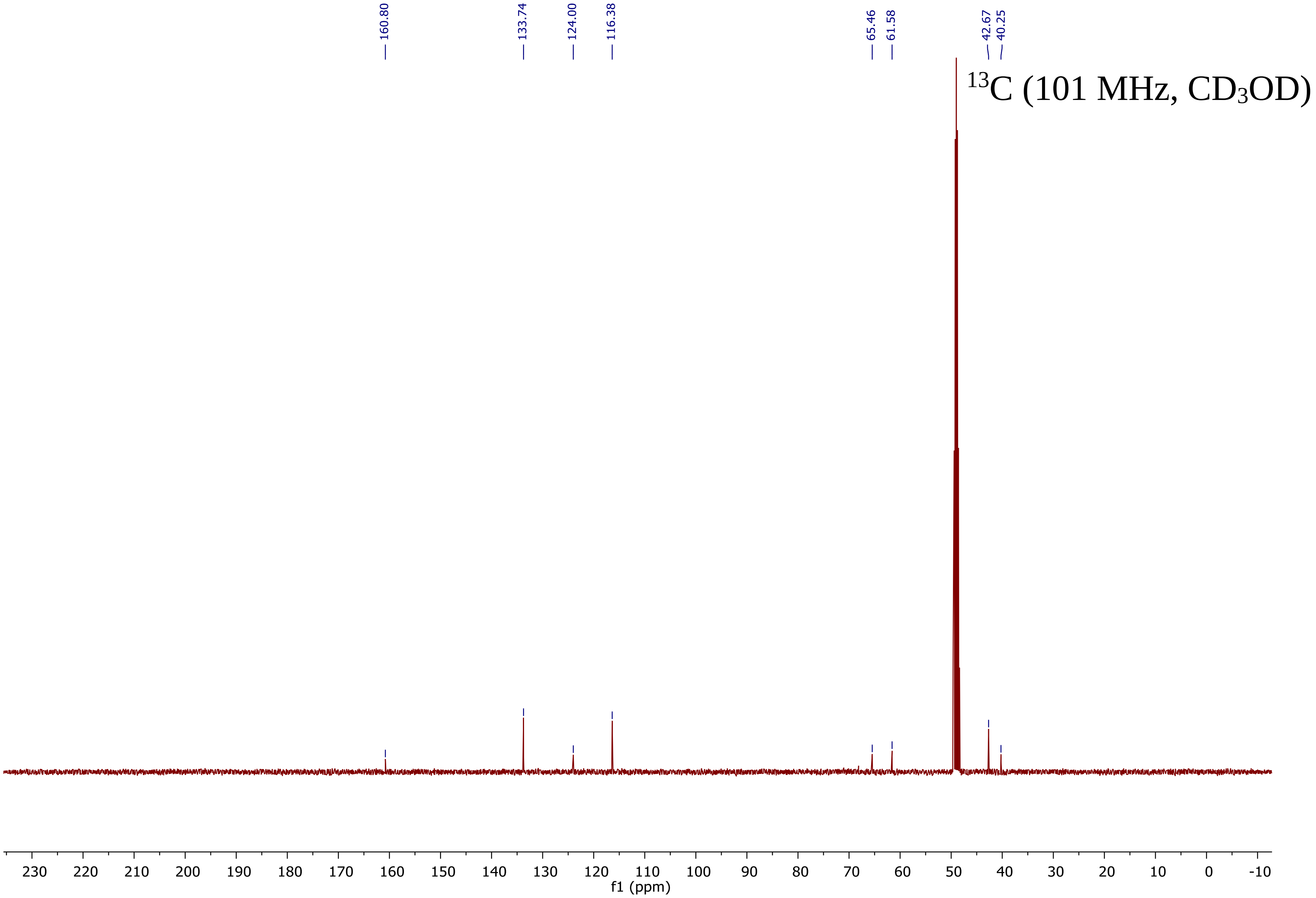
160.80
133.74
124.00
116.38
65.46
61.58
42.67
40.25
13C (101 MHz, CD3OD)
230 220 210 200 190 180 170 160 150 140 130 120 110 100 90 80 70 60 50 40 30 20 10 0 -10
f1 (ppm)

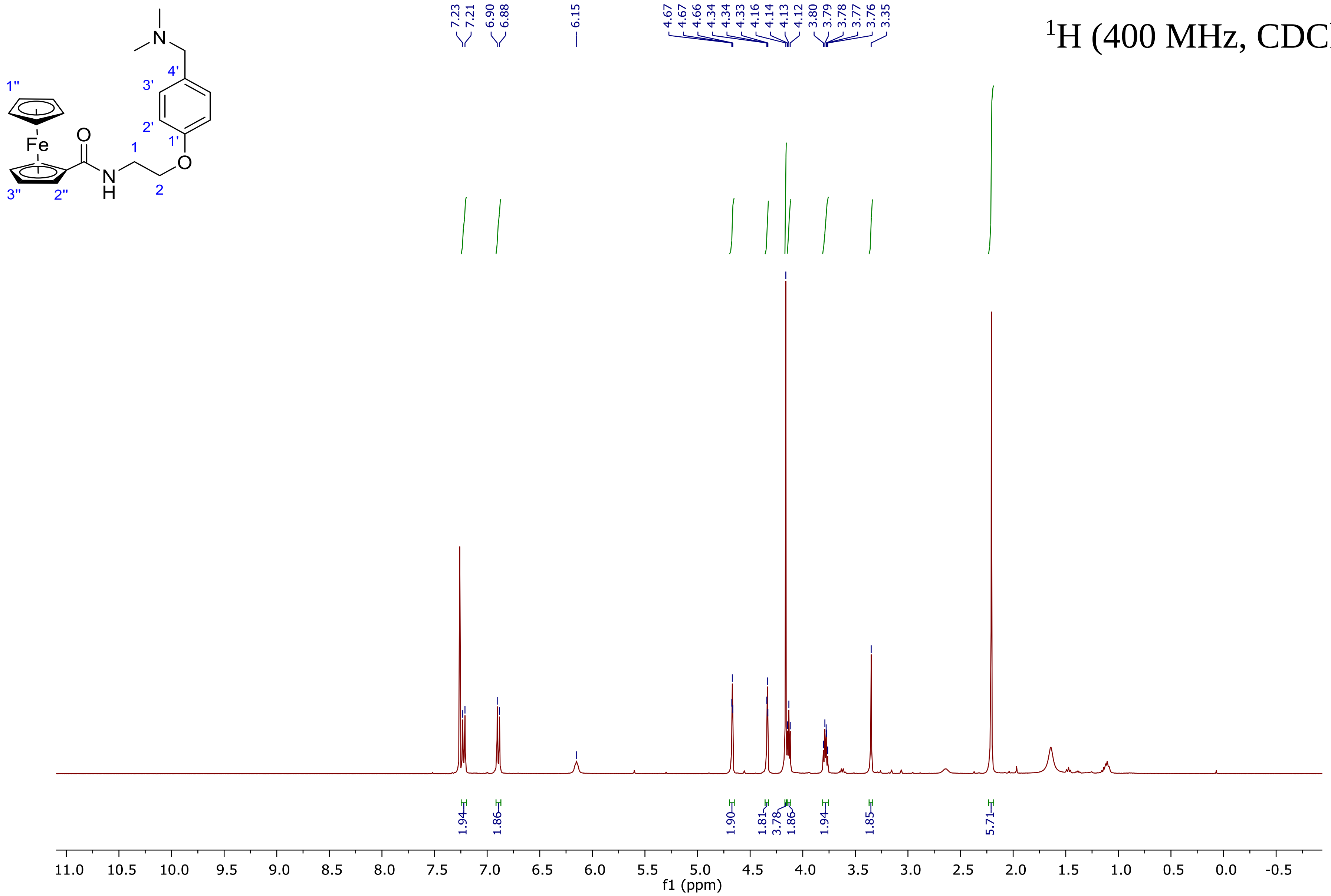

^{1}H (400 MHz, CDCl$_3$)

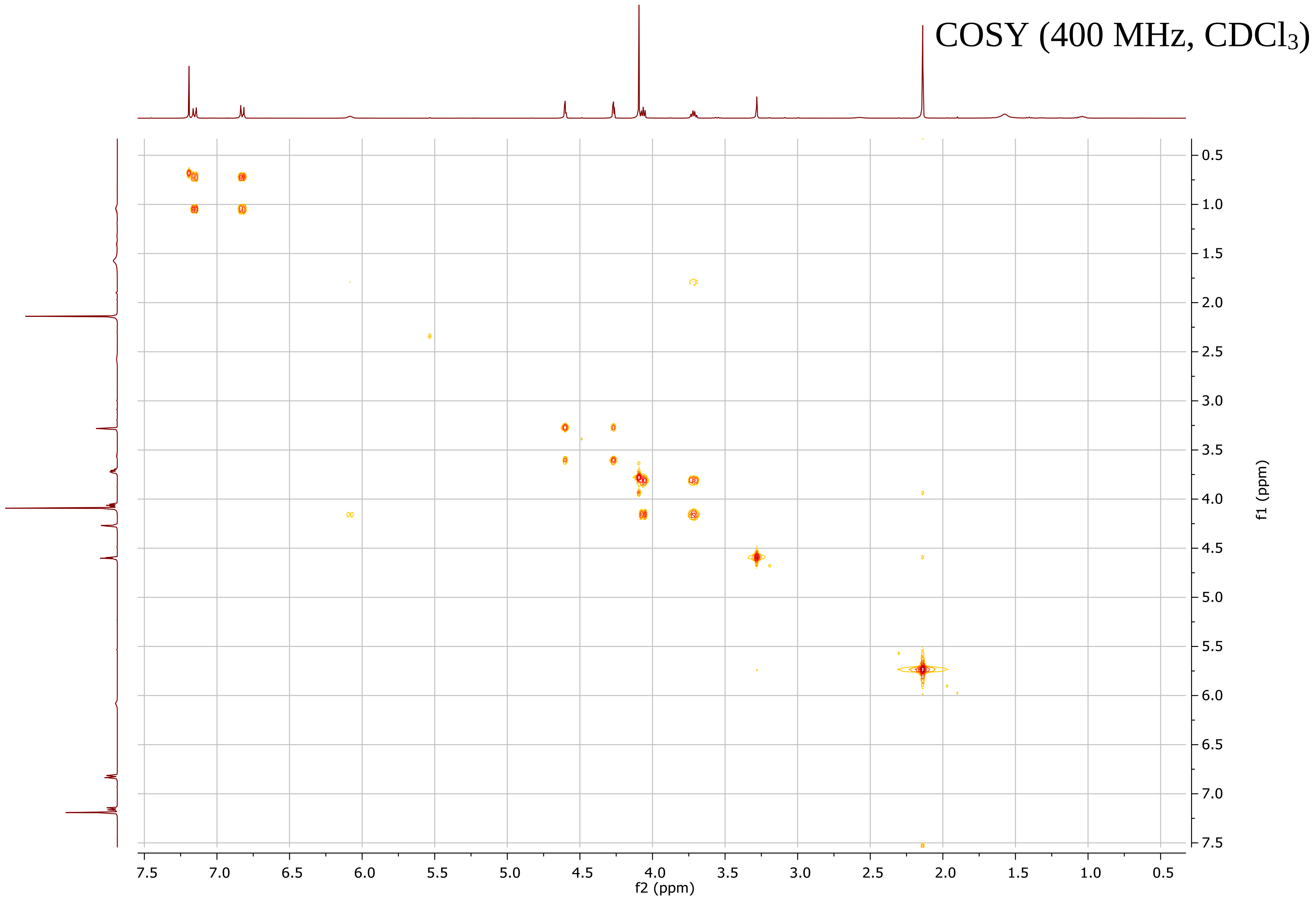

COSY (400 MHz, CDCl3)
7.5
7.0
6.5
6.0
5.5
5.0
4.5
4.0
3.5
3.0
2.5
2.0
1.5
1.0
0.5
f2 (ppm)
f1 (ppm)

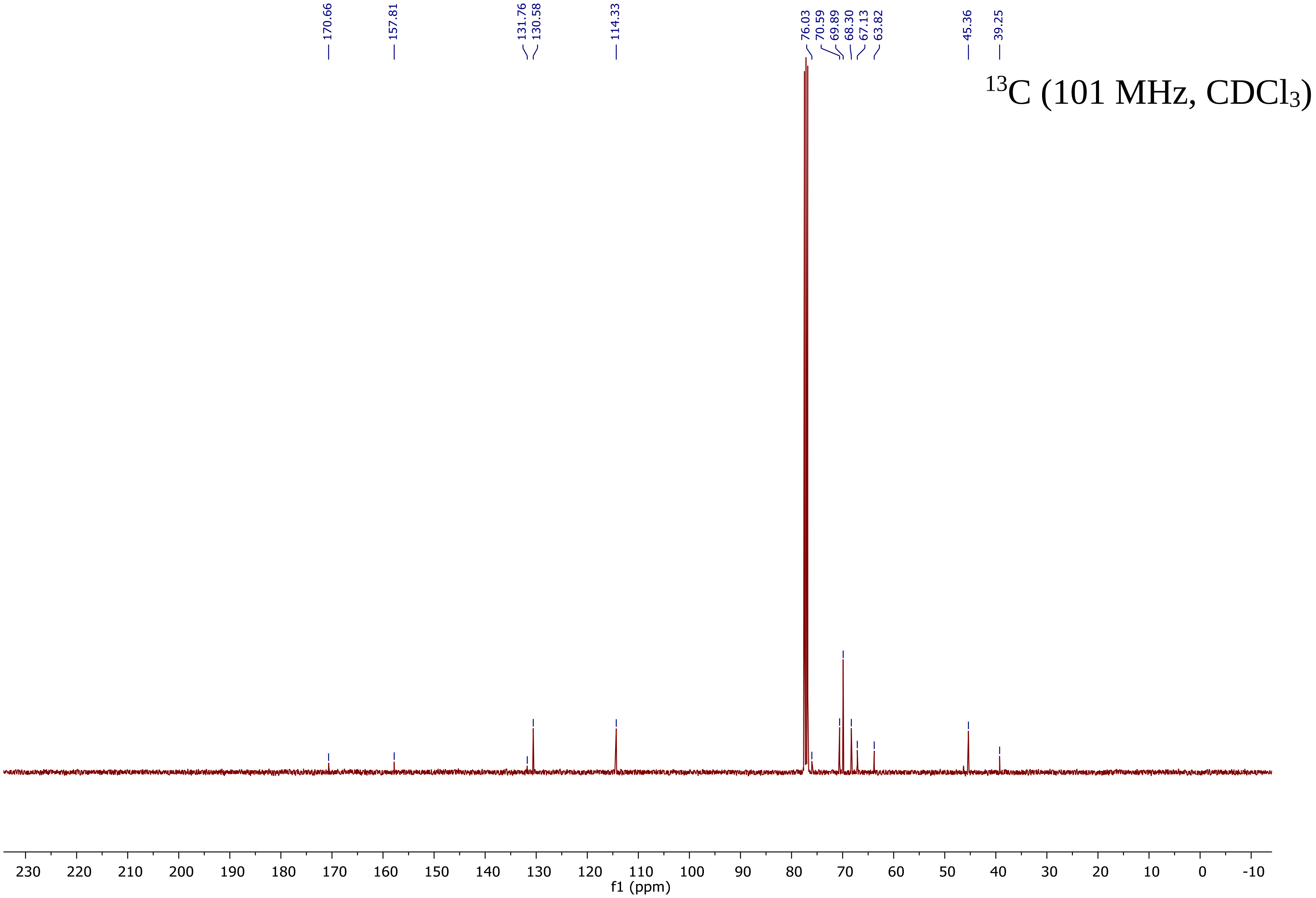
170.66
157.81
131.76
130.58
114.33
76.03
70.59
69.89
68.30
67.13
63.82
45.36
39.25
13C (101 MHz, CDCl3)
230 220 210 200 190 180 170 160 150 140 130 120 110 100 90 80 70 60 50 40 30 20 10 0 -10
f1 (ppm)

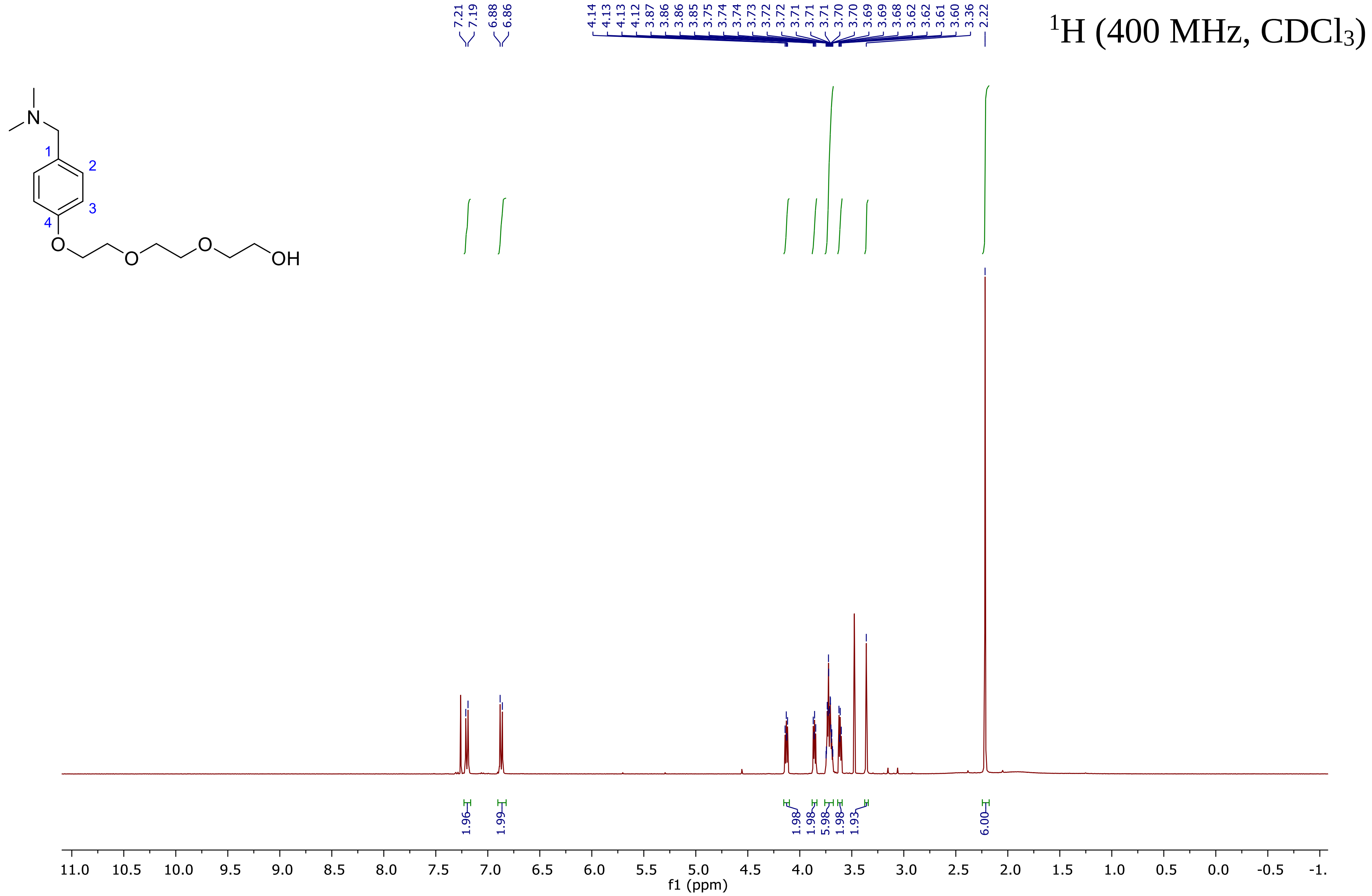

^1H (400 MHz, $CDCl_3$)
7.21
7.19
6.88
6.86
4.14
4.13
4.13
4.12
3.87
3.86
3.86
3.85
3.75
3.74
3.74
3.73
3.72
3.72
3.71
3.71
3.71
3.70
3.70
3.69
3.69
3.68
3.62
3.62
3.61
3.60
3.36
2.22
N
1
2
3
4
O
O
O
OH
1.96
1.99
1.98
1.98
5.98
1.98
1.93
6.00
11.0 10.5 10.0 9.5 9.0 8.5 8.0 7.5 7.0 6.5 6.0 5.5 5.0 4.5 4.0 3.5 3.0 2.5 2.0 1.5 1.0 0.5 0.0 -0.5 -1.
f1 (ppm)

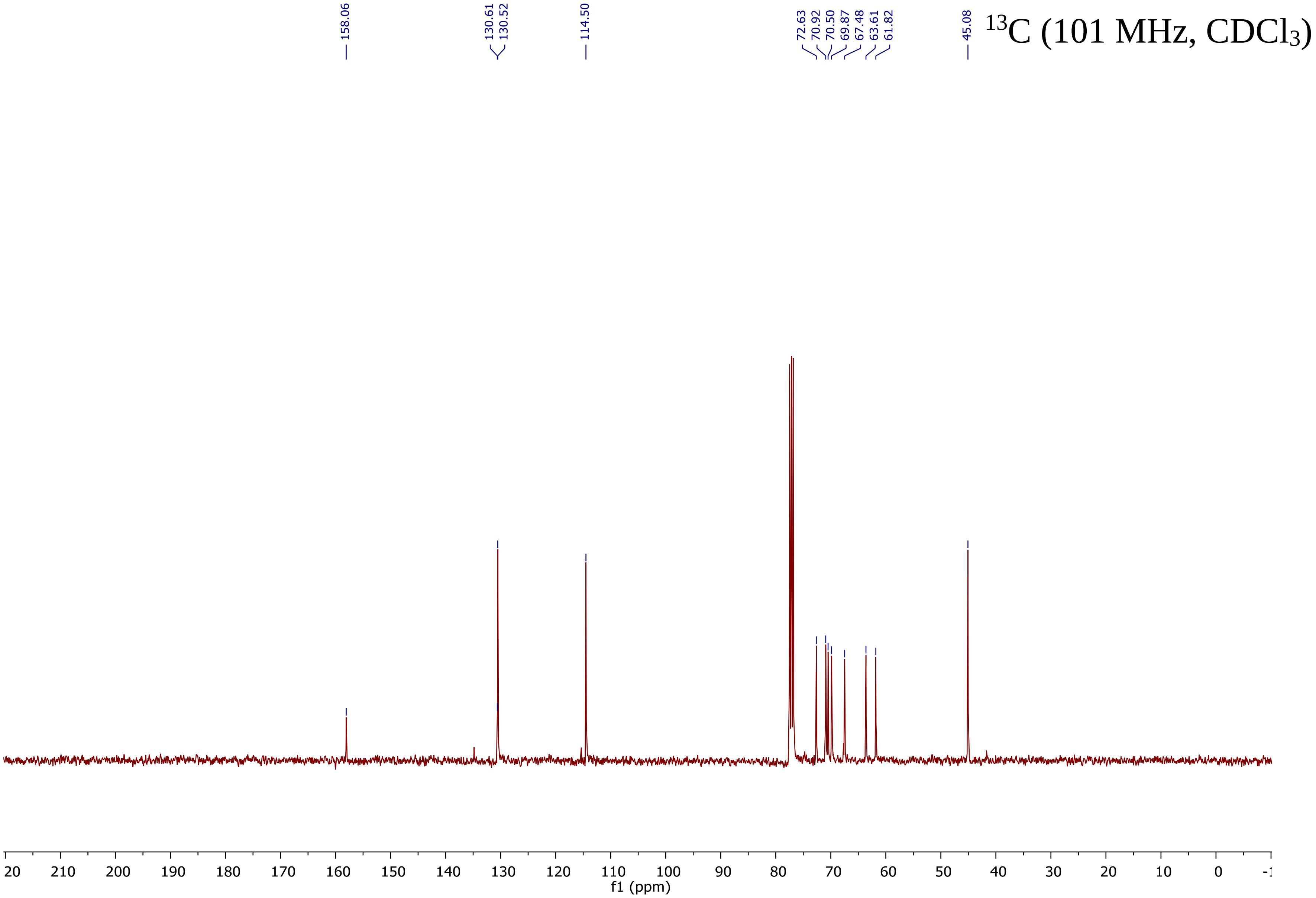
158.06
130.61
130.52
114.50
72.63
70.92
70.50
69.87
67.48
63.61
61.82
45.08
^{13}C (101 MHz, $CDCl_3$)
f1 (ppm)

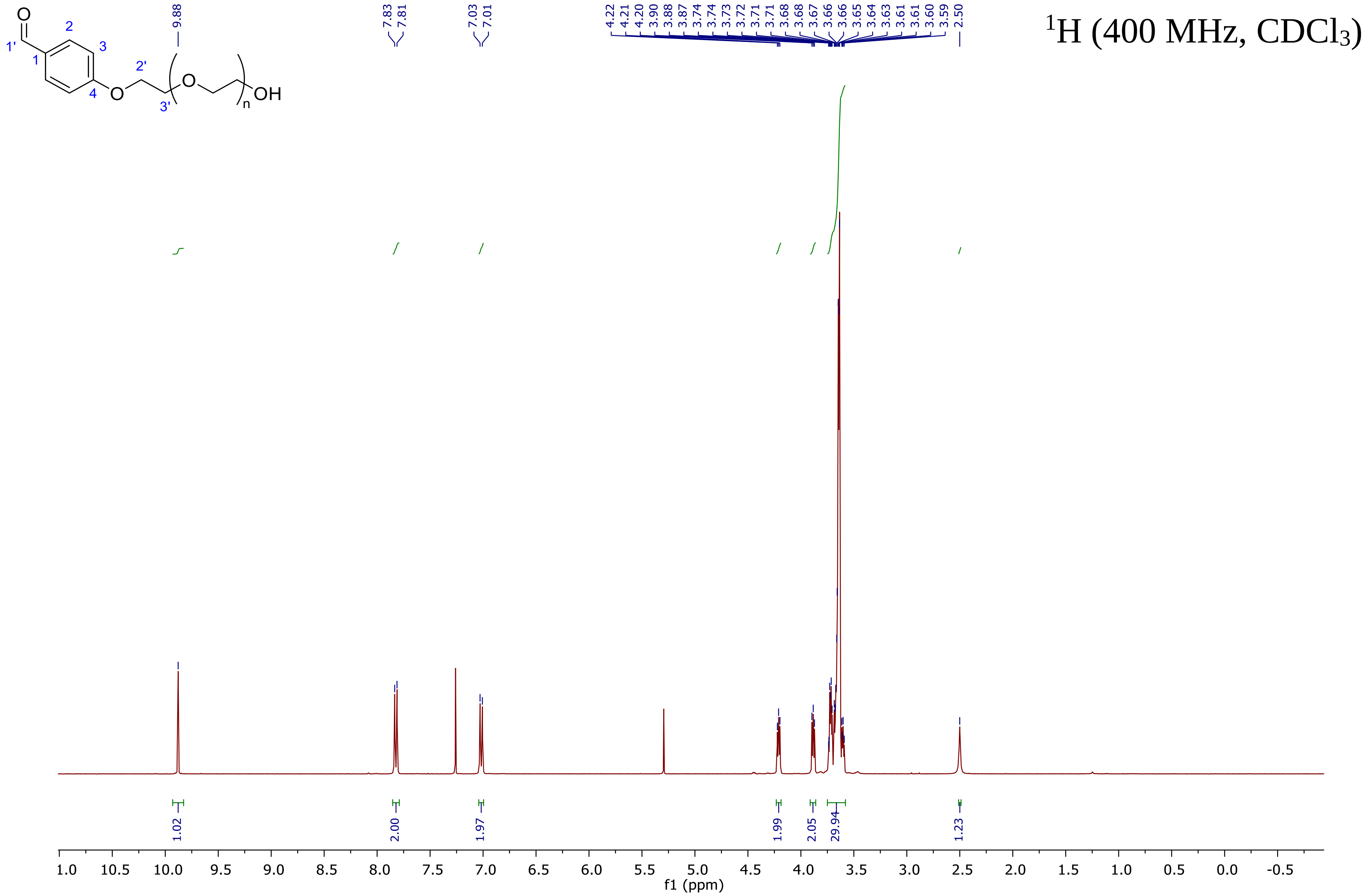

1H (400 MHz, CDCl3)
9.88
7.83
7.81
7.03
7.01
4.22 4.21 4.20 3.90 3.88 3.87 3.74 3.74 3.73 3.72 3.71 3.71 3.68 3.68 3.67 3.66 3.66 3.65 3.64 3.63 3.61 3.61 3.60 3.59 2.50
1.02
2.00
1.97
1.99
2.05
29.94
1.23
f1 (ppm)

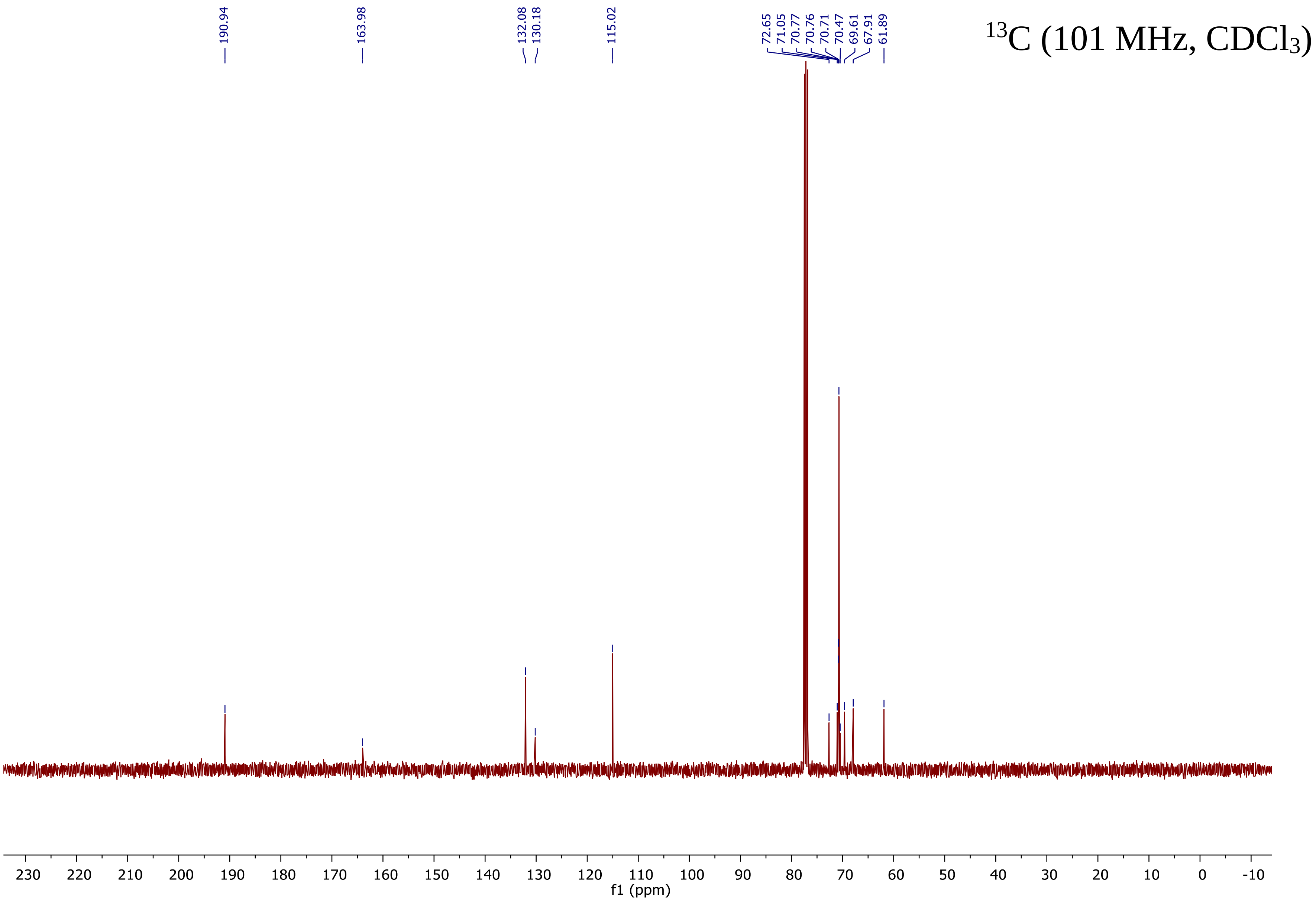

190.94
163.98
132.08
130.18
115.02
72.65
71.05
70.77
70.76
70.71
70.47
69.61
67.91
61.89
^{13}C (101 MHz, $CDCl_3$)
230 220 210 200 190 180 170 160 150 140 130 120 110 100 90 80 70 60 50 40 30 20 10 0 -10
f1 (ppm)

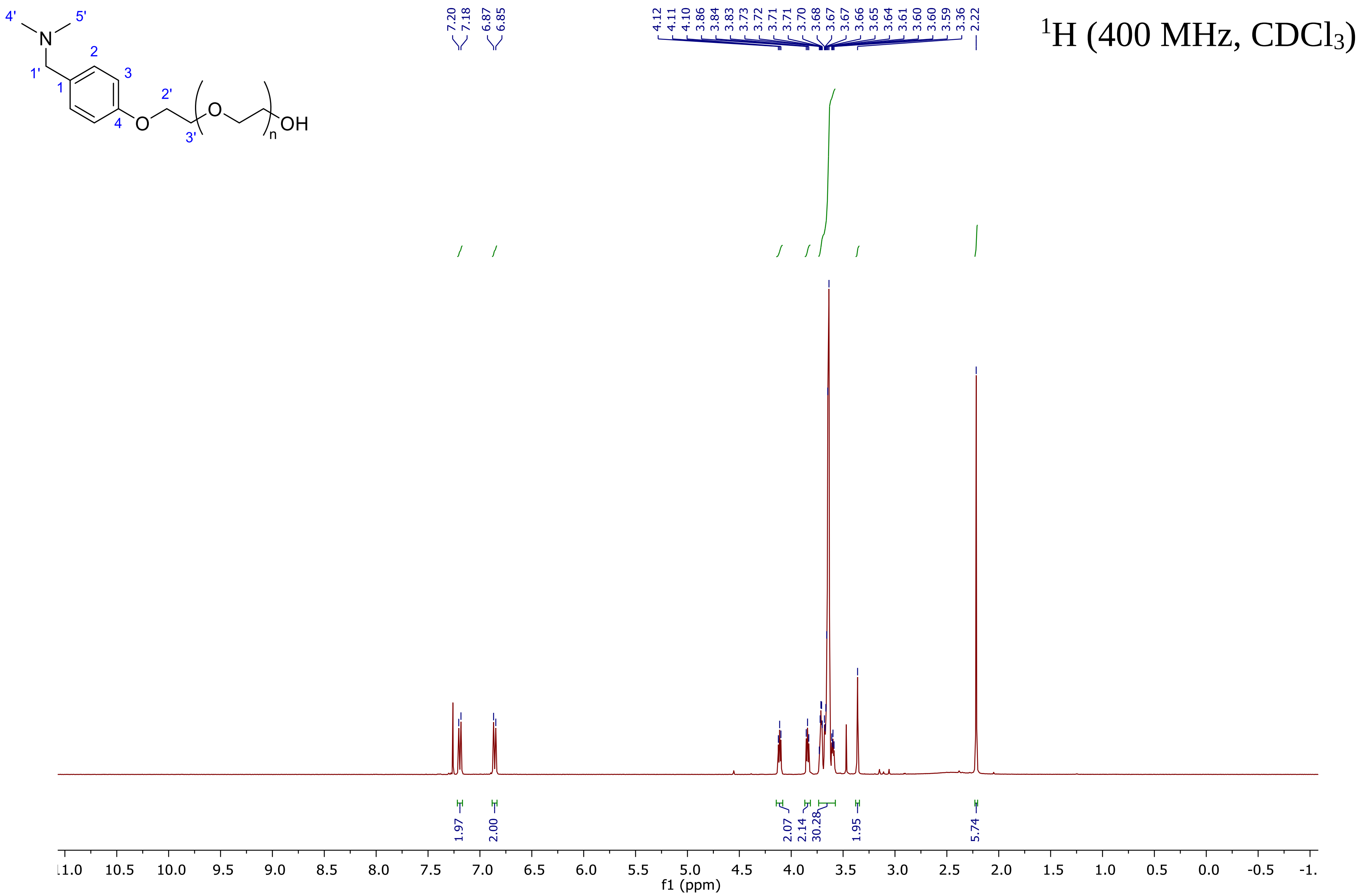

¹H (400 MHz, CDCl₃)
4'
5'
N
1'
2
3
1
4
O
2'
3'
O
n
OH
7.20
7.18
6.87
6.85
4.12
4.11
4.10
3.86
3.84
3.83
3.73
3.72
3.71
3.71
3.70
3.68
3.67
3.67
3.66
3.65
3.64
3.61
3.60
3.60
3.59
3.36
2.22
1.97
2.00
2.07
2.14
30.28
1.95
5.74
11.0 10.5 10.0 9.5 9.0 8.5 8.0 7.5 7.0 6.5 6.0 5.5 5.0 4.5 4.0 3.5 3.0 2.5 2.0 1.5 1.0 0.5 0.0 -0.5 -1.
f1 (ppm)

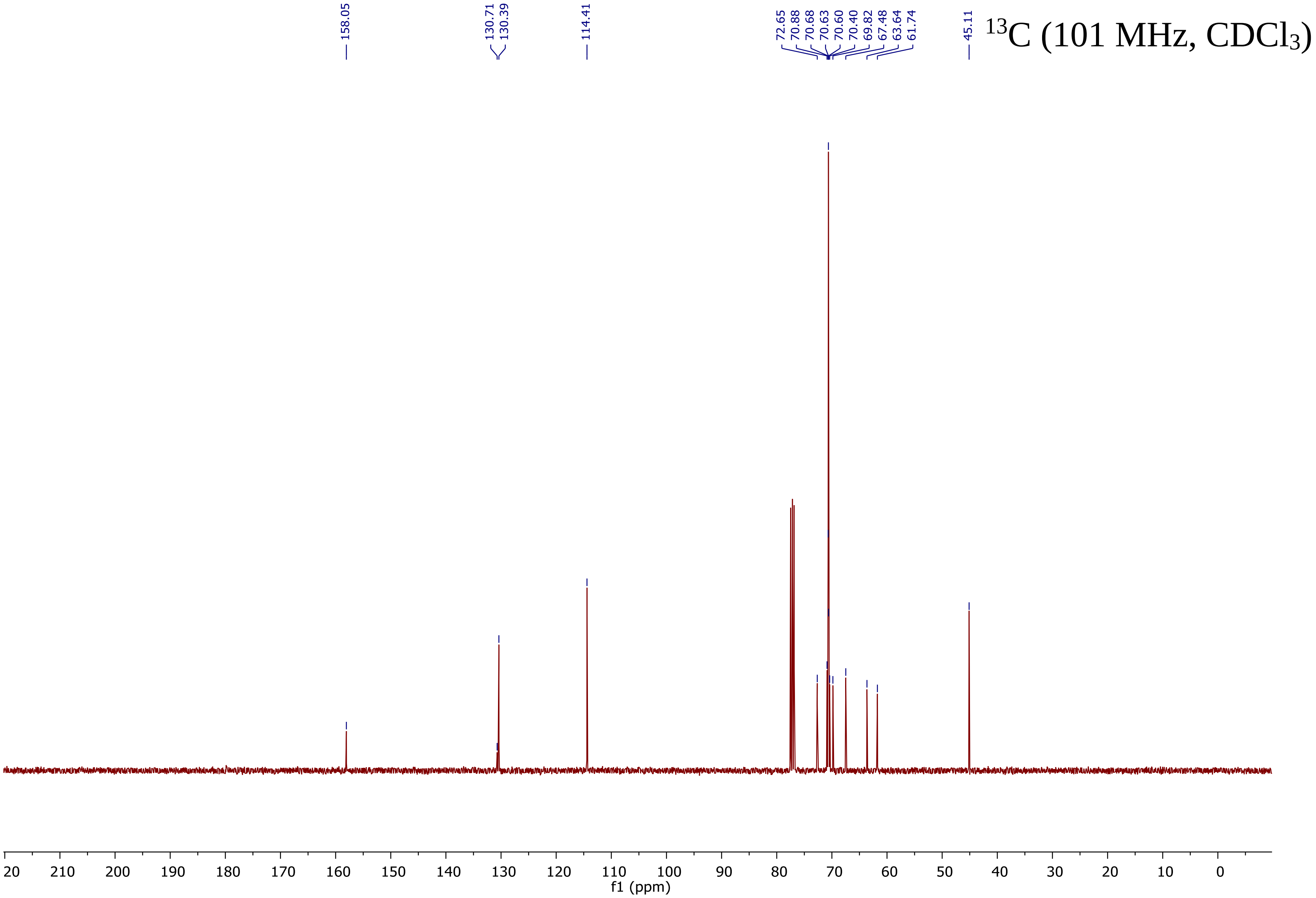

158.05
130.71
130.39
114.41
72.65
70.88
70.68
70.63
70.60
70.40
69.82
67.48
63.64
61.74
45.11
^{13}C (101 MHz, $CDCl_3$)
20
210
200
190
180
170
160
150
140
130
120
110
100
90
80
70
60
50
40
30
20
10
0
f1 (ppm)

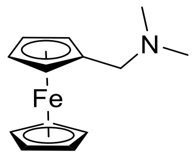

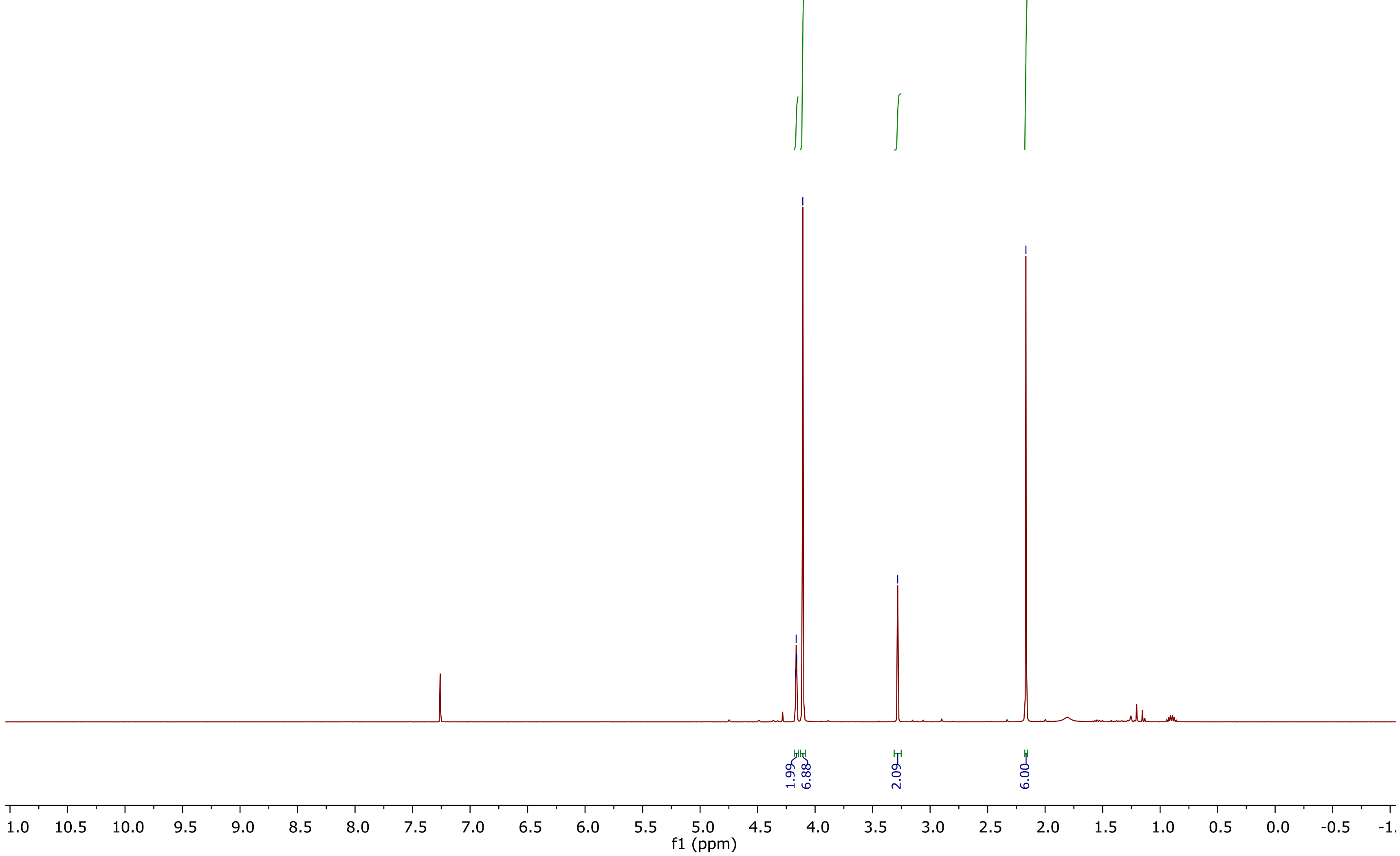

^{1}H (400 MHz, $CDCl_3$)

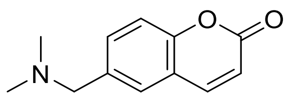

^1H (400 MHz, $CDCl_3$)

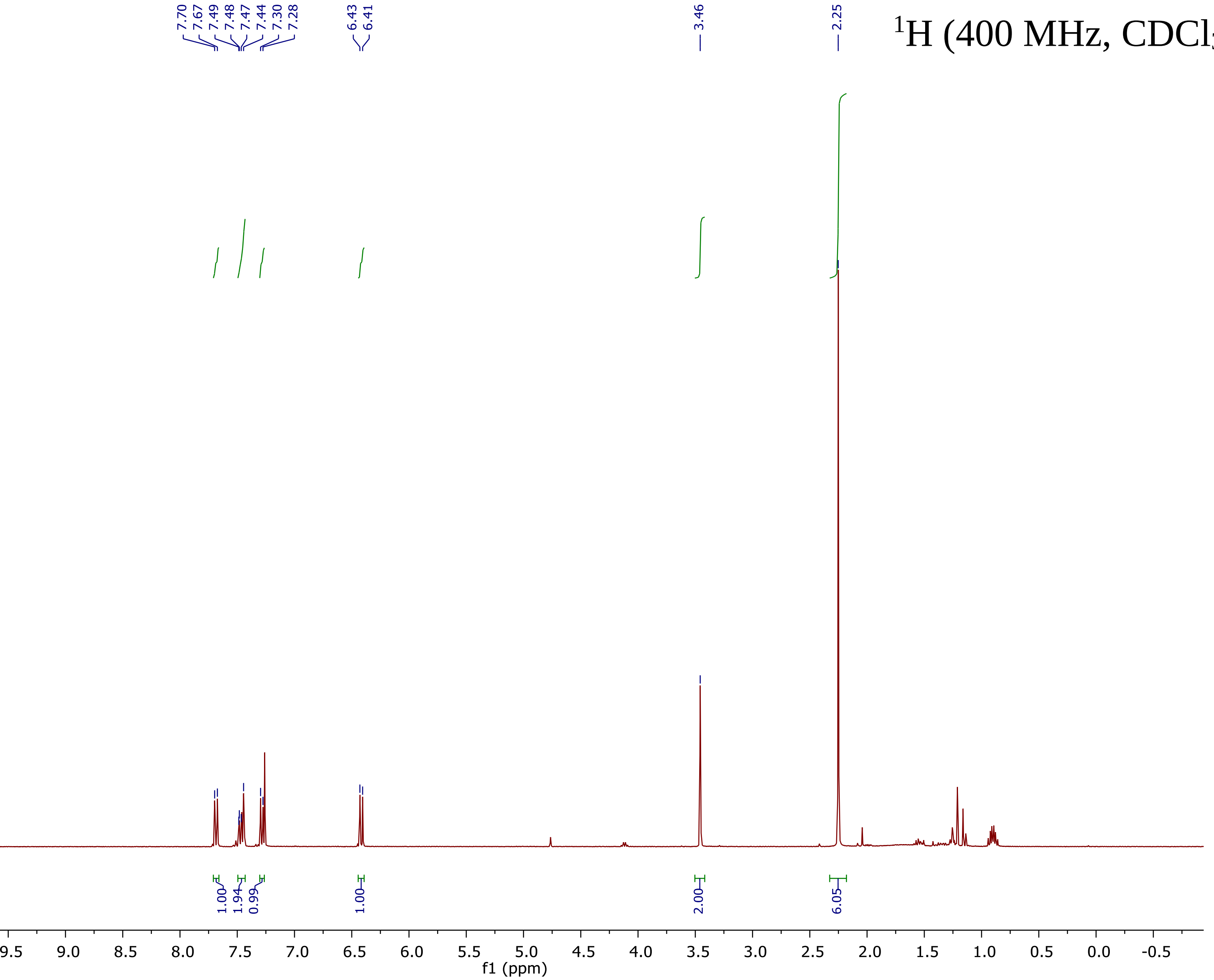

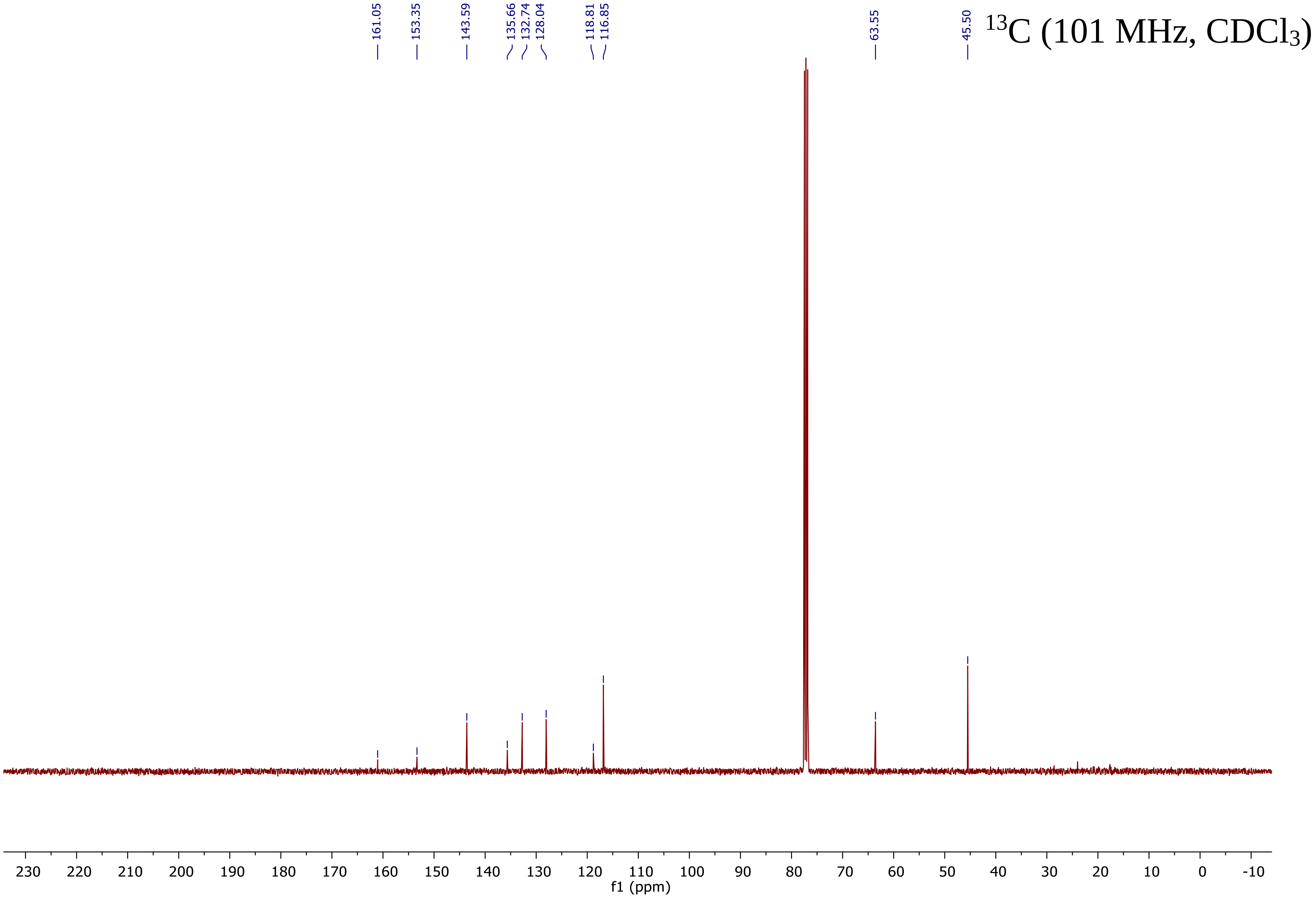
161.05
153.35
143.59
135.66
132.74
128.04
118.81
116.85
63.55
45.50
^{13}C (101 MHz, $CDCl_3$)
230 220 210 200 190 180 170 160 150 140 130 120 110 100 90 80 70 60 50 40 30 20 10 0 -10
f1 (ppm)

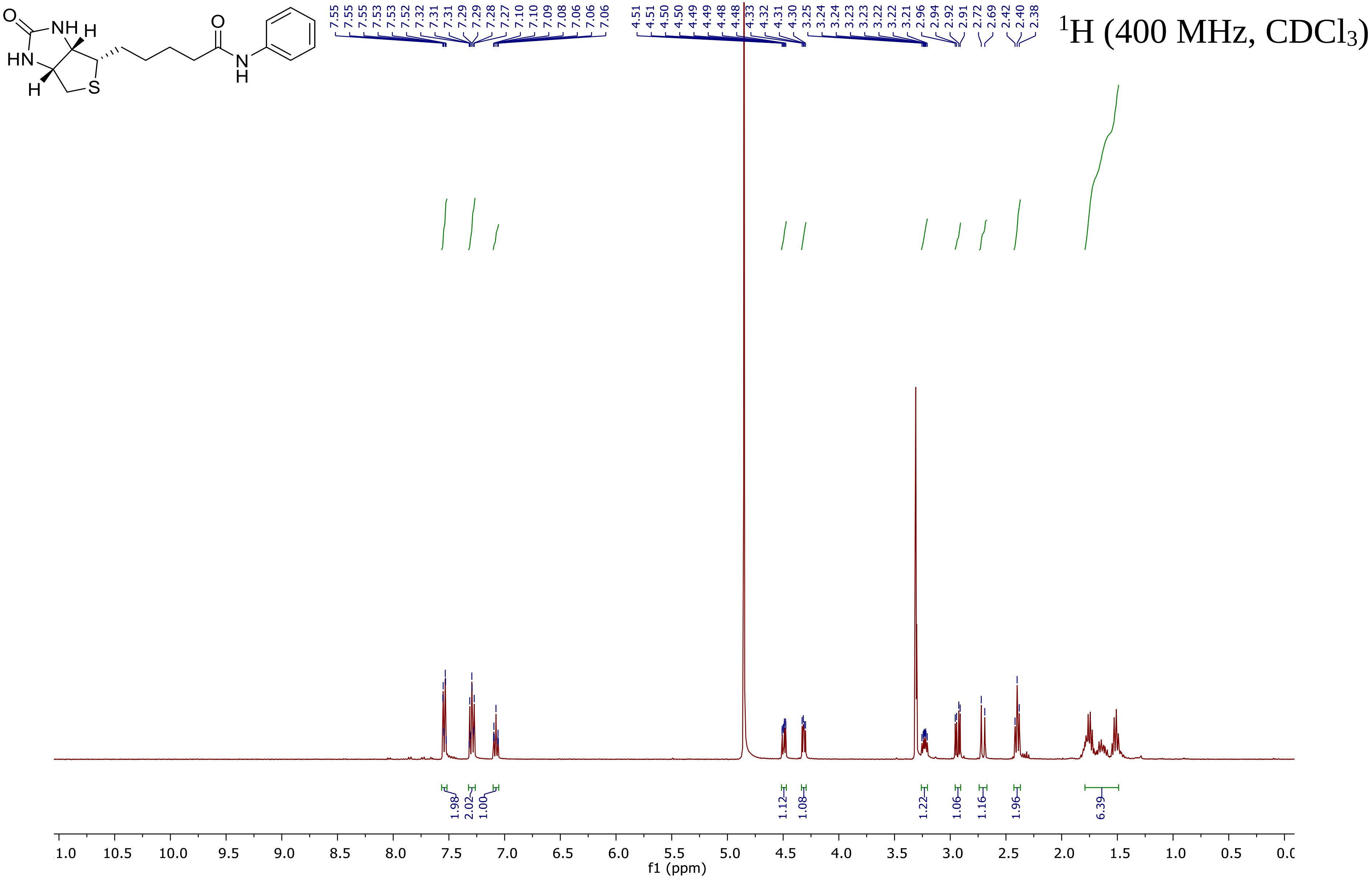

1H (400 MHz, CDCl3)
7.55 7.55 7.55 7.53 7.53 7.52 7.32 7.31 7.29 7.29 7.28 7.27 7.10 7.09 7.08 7.06 7.06 7.06
4.51 4.51 4.50 4.50 4.49 4.49 4.48 4.48 4.33 4.32 4.31 4.30 3.25 3.24 3.24 3.23 3.23 3.22 3.22 3.21 2.96 2.94 2.92 2.91 2.72 2.69 2.42 2.40 2.38
1.98 2.02 1.00 1.12 1.08 1.22 1.06 1.16 1.96 6.39
f1 (ppm)

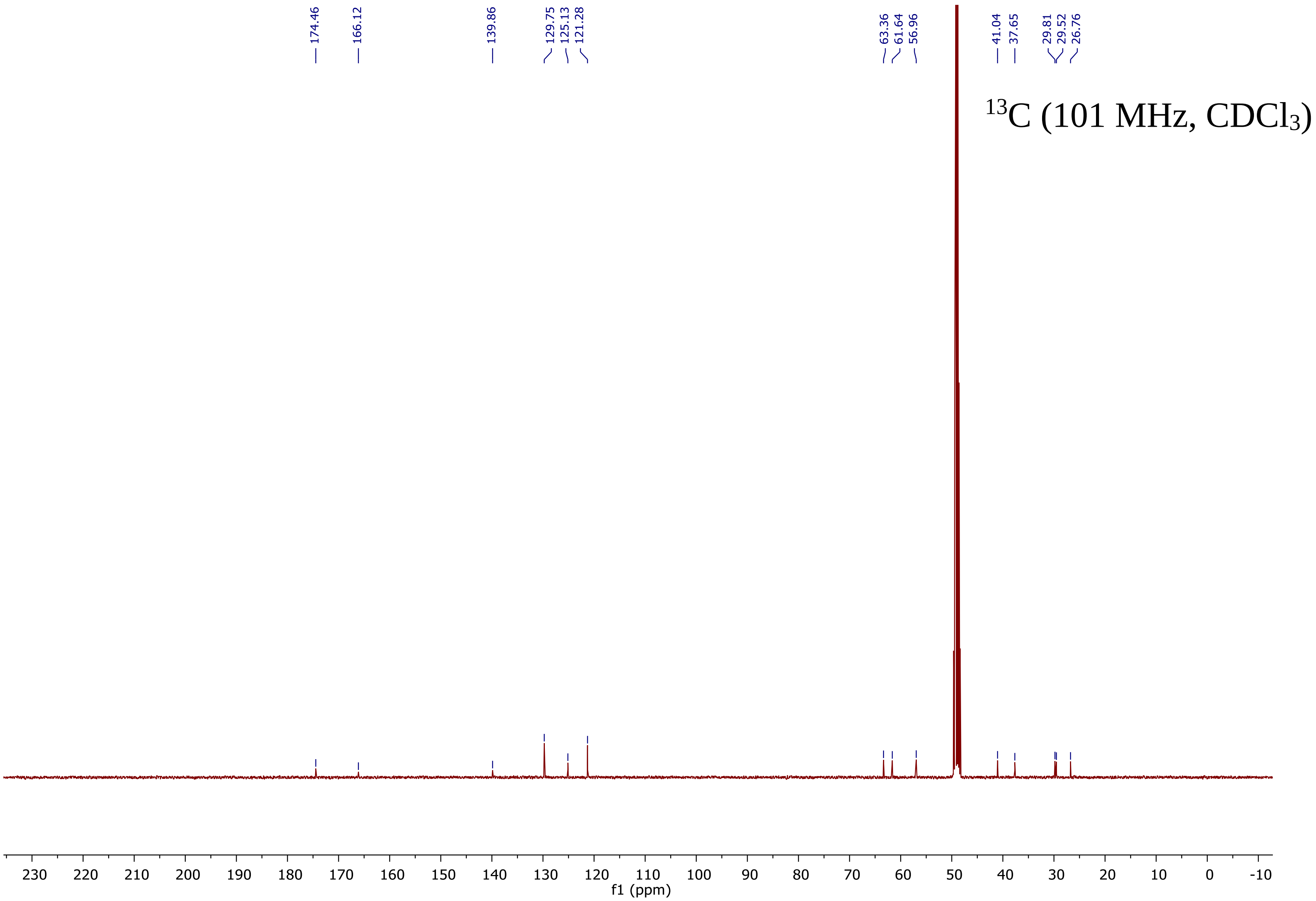
174.46
166.12
139.86
129.75
125.13
121.28
63.36
61.64
56.96
41.04
37.65
29.81
29.52
26.76
^{13}C (101 MHz, $CDCl_3$)
230 220 210 200 190 180 170 160 150 140 130 120 110 100 90 80 70 60 50 40 30 20 10 0 -10
f1 (ppm)

Crystallographic Data

(dmba)Pd(II)Cl(RuPhos)

Identification code	s20cgf1	
Empirical formula	C40 H59 Cl N O3 P Pd	
Formula weight	774.70	
Temperature	150.01(10) K	
Wavelength	1.54184 Å	
Crystal system	Monoclinic	
Space group	$P2_1/c$	
Unit cell dimensions	a = 15.21639(15) Å	α= 90°.
	b = 22.61775(15) Å	β= 113.9415(11)°.
	c = 12.35903(11) Å	γ = 90°.
Volume	3887.52(7) $Å^3$	
Z	4	
Density (calculated)	1.324 Mg/m^3	
Absorption coefficient	5.149 mm^{-1}	
F(000)	1632	
Crystal size	0.110 x 0.070 x 0.020 mm^3	
Theta range for data collection	3.178 to 73.062°.	
Index ranges	-16<=h<=18, -27<=k<=27, -15<=l<=15	
Reflections collected	34752	
Independent reflections	7724 [R(int) = 0.0318]	
Completeness to theta = 67.684°	100.0 %	
Absorption correction	Semi-empirical from equivalents	
Max. and min. transmission	1.00000 and 0.69031	
Refinement method	Full-matrix least-squares on F^2	
Data / restraints / parameters	7724 / 1 / 435	
Goodness-of-fit on F^2	1.016	
Final R indices [I>2sigma(I)]	R1 = 0.0246, wR2 = 0.0621	
R indices (all data)	R1 = 0.0269, wR2 = 0.0635	
Extinction coefficient	n/a	
Largest diff. peak and hole	0.533 and -0.618 e.$Å^{-3}$	

(dmba)Pd(II)Cl(Xantphos)

Identification code	e21cgf2	
Empirical formula	C49 H45 Cl4 N O P2 Pd	
Formula weight	974.00	
Temperature	150.0(3) K	
Wavelength	0.71073 Å	
Crystal system	Monoclinic	
Space group	$P2_1/n$	
Unit cell dimensions	a = 11.7378(3) Å	α= 90°.
	b = 23.3003(5) Å	β= 98.135(2)°.
	c = 16.2116(3) Å	γ = 90°.
Volume	4389.16(17) $Å^3$	
Z	4	
Density (calculated)	1.474 Mg/m^3	
Absorption coefficient	0.778 mm^{-1}	
F(000)	1992	
Crystal size	0.468 x 0.425 x 0.082 mm^3	
Theta range for data collection	3.004 to 27.482°.	
Index ranges	-15<=h<=15, -30<=k<=30, -21<=l<=21	
Reflections collected	40882	
Independent reflections	10037 [R(int) = 0.0345]	
Completeness to theta = 25.242°	99.8 %	
Absorption correction	Semi-empirical from equivalents	
Max. and min. transmission	1.00000 and 0.91801	
Refinement method	Full-matrix least-squares on F^2	
Data / restraints / parameters	10037 / 0 / 527	
Goodness-of-fit on F^2	1.107	
Final R indices [I>2sigma(I)]	R1 = 0.0473, wR2 = 0.1042	
R indices (all data)	R1 = 0.0581, wR2 = 0.1095	
Extinction coefficient	n/a	
Largest diff. peak and hole	1.401 and -1.011 e.$Å^{-3}$	

References

[1] G. Cai, Y. Fu, Y. Li, X. Wan, Z. Shi, *J. Am. Chem. Soc.* **2007**, *129*, 7666–7673.
[2] R. Giri, J. K. Lam, J.-Q. Yu, *J. Am. Chem. Soc.* **2010**, *132*, 686–693.
[3] Z. J. Jiang, Z. H. Li, J. B. Yu, W. K. Su, *J. Org. Chem.* **2016**, *81*, 10049–10055.
[4] S. Nakai, T. Yatabe, K. Suzuki, Y. Sasano, Y. Iwabuchi, J. Hasegawa, N. Mizuno, K. Yamaguchi, *Angew. Chem. Int. Ed.* **2019**, *58*, 16651–16659.
[5] L. Henry, N. Delsuc, C. Laugel, F. Lambert, C. Sandt, S. Hostachy, A. S. Bernard, H. C. Bertrand, L. Grimaud, A. Baillet-Guffroy, C. Policar, *Bioconjug. Chem.* **2018**, *29*, 987–991.
[6] A. Spaeth, A. Graeler, T. Maisch, K. Plaetzer, *Eur. J. Med. Chem.* **2018**, *159*, 423–440.
[7] H. Li, P. Zhang, L. P. Smaga, R. A. Hoffman, J. Chan, *J. Am. Chem. Soc.* **2015**, *137*, 15628–15631.
[8] D. Y. Ong, Z. Yen, A. Yoshii, J. Revillo Imbernon, R. Takita, S. Chiba, *Angew. Chem. Int. Ed.* **2019**, *58*, 4992–4997.
[9] D. Chamorro-Arenas, U. Osorio-Nieto, L. Quintero, L. Hernández-García, F. Sartillo-Piscil, *J. Org. Chem.* **2018**, *83*, 15333–15346.
[10] V. N. Tsarev, Y. Morioka, J. Caner, Q. Wang, R. Ushimaru, A. Kudo, H. Naka, S. Saito, *Org. Lett.* **2015**, *17*, 2530–2533.
[11] G. A. Price, A. K. Brisdon, S. Randall, E. Lewis, D. M. Whittaker, R. G. Pritchard, C. A. Muryn, K. R. Flower, P. Quayle, *J. Organomet. Chem.* **2017**, *846*, 251–262.
[12] S. A. Spring, S. Goggins, C. G. Frost, *Org. Biomol. Chem.* **2017**, *15*, 7122–7126.
[13] N. L. Bell, C. Xu, J. W. B. Fyfe, J. C. Vantourout, J. Brals, S. Chabbra, B. E. Bode, D. B. Cordes, A. M. Z. Slawin, T. M. McGuire, A. J. B. Watson, *Angew. Chem. Int. Ed.* **2021**, *60*, 7935–7940.
[14] E. V. Vinogradova, C. Zhang, A. M. Spokoyny, B. L. Pentelute, S. L. Buchwald, *Nature* **2015**, *526*, 687–691.
